# Evidence for mitochondrial Lonp1 expression in the nucleus

**DOI:** 10.1038/s41598-022-14860-0

**Published:** 2022-06-27

**Authors:** Lara Gibellini, Rebecca Borella, Anna De Gaetano, Giada Zanini, Domenico Lo Tartaro, Gianluca Carnevale, Francesca Beretti, Lorena Losi, Sara De Biasi, Milena Nasi, Mattia Forcato, Andrea Cossarizza, Marcello Pinti

**Affiliations:** 1 Department of Medical and Surgical Sciences for Children and Adults, University of Modena and Reggio Emilia, Modena, Italy; 2 Department of Life Sciences, University of Modena and Reggio Emilia, Via Campi 287, 41125 Modena, Italy; 3 Department of Surgical, Medical, Dental and Morphological Sciences, University of Modena and Reggio Emilia, Modena, Italy; 4 Department of Biomedical, Metabolic and Neural Sciences, University of Modena and Reggio Emilia, Modena, Italy

**Keywords:** Mitochondria, Cell biology, Organelles, Nucleus

## Abstract

The coordinated communication between the mitochondria and nucleus is essential for cellular activities. Nonetheless, the pathways involved in this crosstalk are scarcely understood. The protease Lonp1 was previously believed to be exclusively located in the mitochondria, with an important role in mitochondrial morphology, mtDNA maintenance, and cellular metabolism, in both normal and neoplastic cells. However, we recently detected Lonp1 in the nuclear, where as much as 22% of all cellular Lonp1 can be found. Nuclear localization is detectable under all conditions, but the amount is dependent on a response to heat shock (HS). Lonp1 in the nucleus interacts with heat shock factor 1 (HSF1) and modulates the HS response. These findings reveal a novel extramitochondrial function for Lonp1 in response to stress.

## Introduction

Recently, there has been increasing evidence that mitochondria not only communicate with the nucleus but that several proteins containing mitochondrial-targeting sequences also reside or translocate to the nucleus under certain conditions^1^. These proteins can translocate in response to several stimuli and likely perform a similar function in both mitochondrial and nuclear compartments^1^. Examples of mitochondrial protein moonlighting to the nucleus include: mammalian nuclear factor erythroid 2-related factor 2 (NRF2), Activating Transcription Factor associated with Stress 1 (ATFS-1), the monooxygenase CLK-1, the pyruvate dehydrogenase complex (PDC), and fumarase^1–4^.

Lonp1 is a mitochondrial protease which plays a crucial role in the maintenance of mitochondrial functions^5^. Three Lonp1 variants are present in humans, resulting from the alternative splicing of exon 1. Global loss of Lonp1 causes fragmentation of the mitochondrial network, associated with severe alterations of *cristae*, as well as mitochondrial membrane depolarization and reduced respiratory capacity^6,7^. On the contrary, Lonp1 overexpression is associated with unchanged mitochondrial morphology and mass^8^. Changes in Lonp1 expression have been linked to epithelial-mesenchymal transition in cancer^8–10^, and mutations in the Lonp1 sequence have been linked to cerebral, ocular, dental, auricular and skeletal syndromes^5,11^. In mitochondria, Lonp1 is mainly involved both in the degradation of misfolded, oxidatively modified and regulatory proteins, and in the preservation of mitochondrial DNA (mtDNA)^12–14^. Recently however, Lonp1 has also been reported to play a role in metabolism remodelling^7^. This indicates that additional extra-mitochondrial functions of Lonp1 may exist^15^. As previous assumptions have believed that Lonp1 is located exclusively in the mitochondria, possible nuclear roles of this protein have been paid little attention. Here we provide evidence that Lonp1 can be present in the nuclei of murine and human tissue and cultured human cells. The nuclear localization of Lonp1 is increased in response to heat shock (HS) is independent of either DNA damage or cell cycle.

## Results

### Lonp1 localization in mitochondria and nuclei

**Figure 1:**
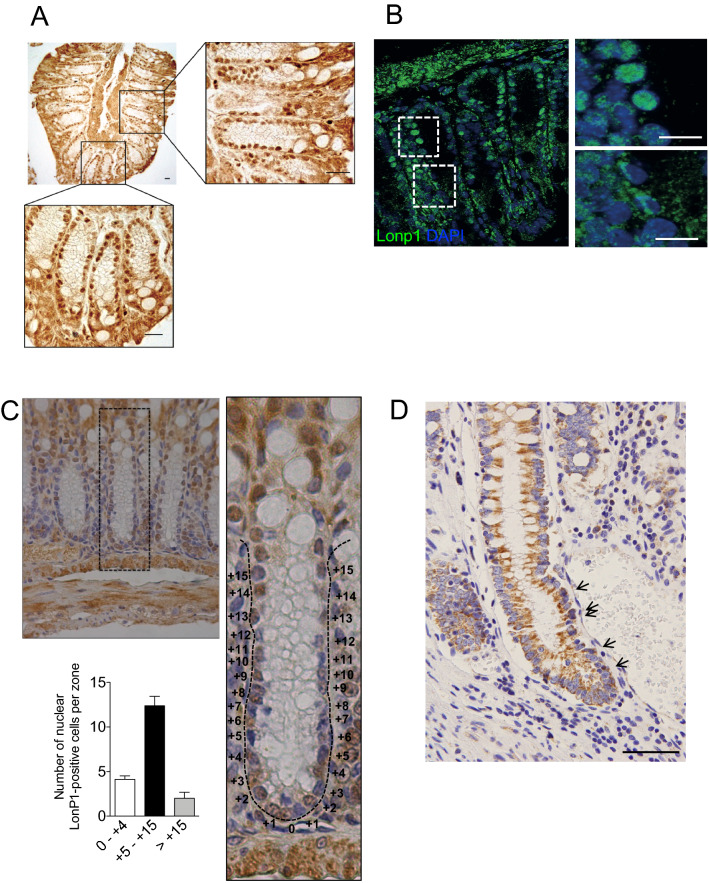
Lonp1 localizes in the nuclei of mouse colon tissue and human colon tissue. (**A**) Representative images of Lonp1 IHC on murine colon tissue, showing Lonp1 nuclear localization. (**B**) Representative images of Lonp1 IF on murine colon tissue, showing Lonp1 nuclear localization. Nuclei were counterstained with DAPI. (**C**) Representative Ki-67 IHC staining in intestinal crypt. Cells of the crypt were sectioned into three zones: the lower (0 to + 4), upper (+ 5 to + 15), and non (> + 15)-crypt zones. The histogram represents the percentage of cells with nuclear Lonp1 per crypt zone (n = 10 crypts per group). Data represent the mean ± SD. (**D**) Representative images of Lonp1 IHC on human colon tissue, showing Lonp1 nuclear localization (arrows). Bar: 200 µm.

Previous data from our lab revealed that mitochondrial Lonp1 is overexpressed in colon cancer cells and tissue, and that it is involved in energetic metabolism and epithelial-mesenchymal transition in these cells^6–8,16^. Lonp1 was assumed to localize exclusively in mitochondria. Intriguingly, while analysing the tissue-specific expression of Lonp1 in mice, we observed the nuclear localisation of Lonp1 in murine gut epithelium (Fig. 1), both by immunofluorescence and immunohistochemistry. We observed Lonp1 expression in the nuclei of healthy gut cell samples from adult wild-type mice (Fig. 1A and supplementary Fig. 1), confirmed by immunofluorescence (Fig. 1B). Ki-67 staining enabled the observation of section crypts in the lower (0 to + 4), upper (+ 5 to + 15) and non-crypt (> + 15; Fig. 1C) zones. Detailed analysis of Lonp1 nuclear expression within the crypts showed that positivity of Lonp1 was concentrated in the transient amplifying (TA) cell area (+ 5 to + 15; Fig. 1C). We therefore analysed Lonp1 subcellular localization in human colon tissue, and also found Lonp1 human nuclear localisation (Fig. 1D; indicated by arrows).

**Figure 2:**
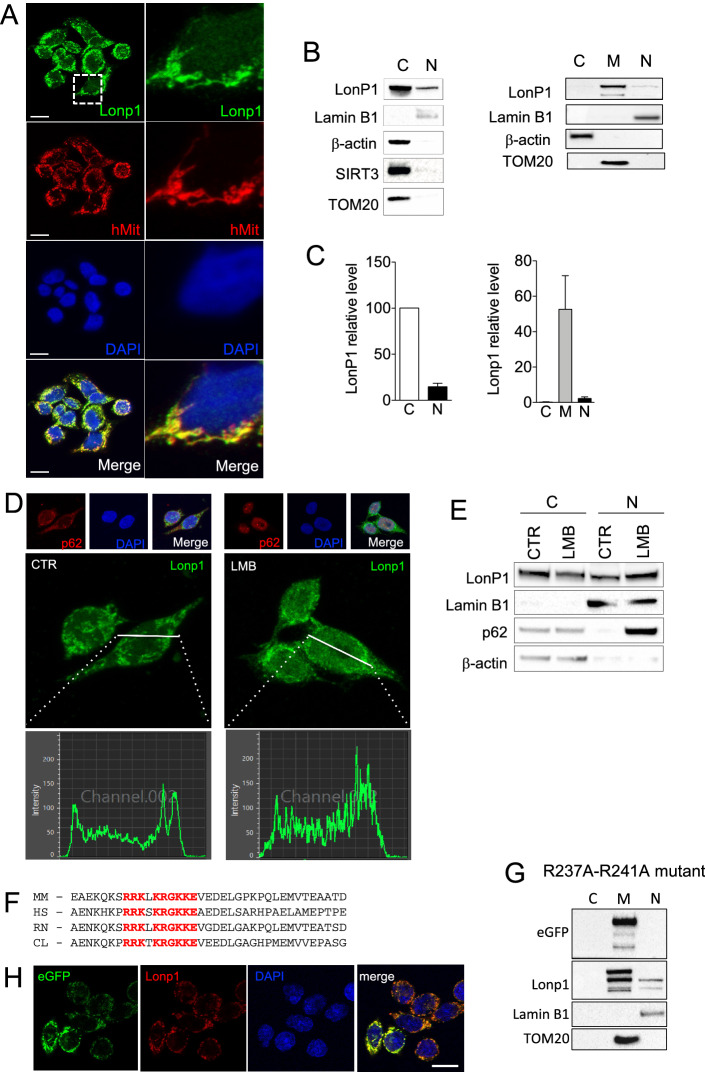
A fraction of Lonp1 localizes in the nuclei of human cells. (**A**) Representative confocal microscopy images of SW620 cells after immunostaining with anti-Lonp1 and anti-human mitochondria (hMit) Abs. Nuclei were counterstained with DAPI. Bars: 10 mm. (**B**) Left panel: representative immunoblot of cytosolic (C) and nuclear (N) fractions obtained from SW620 cells. Lamin B1 is the nuclear fraction loading control, whereas β-actin is the cytosolic fraction loading control. Immunoblots of Sirtuin-3 (SIRT3) and TOM20 are also reported to indicate that mitochondrial contamination is not present. Blots were cut prior to hybridisation with antibodies during blotting. Right panel: representative immunoblot of cytosolic (C), mitochondrial (M), nuclear (N) fractions obtained from SW620 cells. β-actin is the cytosolic fraction loading control, TOM20 is the mitochondrial fraction loading control and lamin B1 is the nuclear fraction loading control. (**C**) Left histogram: quantification of the ratio of nuclear and cytosolic Lonp1 (n = 3 independent experiments). Right histogram: quantification of the ratio of cytosolic, mitochondrial and nuclear Lonp1 (n = 3 independent experiments). (**D**) Representative confocal microscopy images of SW620 cells after immunostaining with anti-Lonp1 antibody. Cells were treated or not with leptomycin B (LMB) to inhibit nuclear export of proteins. Staining of p62 was reported as positive control for LBM treatment. Nuclei were counterstained with DAPI. (**E**) Representative immunoblot of cytosolic (cytosol) and nuclear (N) fractions obtained from SW620 cells, treated or not with LBM. Lamin B1 is the nuclear fraction loading control, whereas β-actin is the cytosolic fraction loading control. Immunoblot for p62 is also reported as positive control for LMB treatment. Blots were cut prior to hybridisation with antibodies during blotting. (**F**) Alignment of Lonp1 protein sequences (amino acids 218 to 256) from the indicated mammalian species. HS, *Homo sapiens*; CL, *Canis lupus familiaris*; MM, *Mus musculus*; RN, *Rattus norvegicus*. The nuclear targeting sequence is highlighted in red. (**G**) Representative immunoblot of cytosolic (C), mitochondrial (M), nuclear (N) fractions obtained from SW620 cells expressing the R237A-R241A mutant of Lonp1. β-actin is the cytosolic fraction loading control, TOM20 is the mitochondrial fraction loading control and lamin B1 is the nuclear fraction loading control. Blots were cut prior to hybridisation with antibodies during blotting. (**H**) Representative confocal microscopy images of SW620 cells expressing the R237A-R241A mutant of eGFP-Lonp1 after immunostaining with anti-Lonp1 antibody. Nuclei were counterstained with DAPI. Bar: 10 mm.

To verify the nuclear presence of Lonp1 in neoplastic cells, we next examined Lonp1 subcellular localization in human cell lines. As shown in Fig. 2A, we found that endogenously expressed Lonp1 is present mainly in mitochondria, but also in nuclei of SW620 cells. Thus, we looked at the nuclear fraction of SW620 cells, and confirmed the presence of Lonp1 (Fig. 2B, left panel). The quantification of Lonp1 expression in nuclear and cytosolic fractions obtained from SW620 cells also revealed that 10 to 22% of Lonp1 is present in the nucleus of these cells (Fig. 2B,C). To further confirm that Lonp1 is specifically present in the nucleus, we separated nuclear, cytosolic and mitochondrial fractions, and found Lonp1 in the mitochondria and in the nucleus, but not in the cytosol (Fig. 2B, right panel). The quantification of Lonp1 in these fractions revealed that it was present mainly in mitochondria (Fig. 2C). A small fraction of Lonp1 was also present in the nucleus. Nuclear localization was also confirmed in two other colon cancer cell lines, namely SW48 and SW480, and in HeLa cells (Supplementary Fig. 2). Subsequently, we treated cells overnight with 50 nM leptomycin B (LMB), which inhibits nuclear export. The expression and subcellular distribution of p62 was used as a positive control to confirm the effectiveness of LMB treatment. In this condition, decreased levels of Lonp1 were observed in the cytoplasm, whereas increased levels were observed in the nucleus of treated cells (Fig. 2D,E), indicating that a fraction of Lonp1 translocates into the nucleus.

We next searched for nuclear localization signal (NLS) in a murine and human Lonp1 aminoacidic sequence with cNLS Mapper (available at http://nls-mapper.iab.keio.ac.jp/cgi-bin/NLS_Mapper_form.cgi). A putative NLS sequence (RRKLKRGKKEVE) was identified in Lonp1 between amino acid 225 and 236. This sequence is conserved in human Lonp1 (RRKSKRGKKEAE), as well as in other mammals (Fig. 2F). Thus, we mutated the NLS sequence by introducing two point mutations (R237A and R241A), tagged Lonp1 at C-term with eGFP and we overexpressed this mutant form of Lonp1 in SW620 cells. When we used an anti GFP Ab, we did not detect the protein in the nuclear fraction, suggesting that this sequence is needed for nuclear targeting. Conversely, the anti Lonp1 Ab revealed a weak but detectable signal in the nucleus, corresponding to the molecular weight of the native protein (Fig. 2G). Confocal microscopy analysis confirmed this observation (Fig. 2H).

### Lonp1 localizes to the nucleus in response to heat shock

The dual localization of Lonp1 in the mitochondria and the nucleus may be part of a regulatory mechanism that links mitochondrial function to nuclear events. To identify the potential functions of the nuclear protein and considering that Lonp1 nuclear localization correlates with Ki-67 expression, at least in mice, we first investigated whether Lonp1 could be involved in cell cycle regulation. For this reason, SW620 cells were exposed to thymidine, which causes cell cycle arrest in G0/G1, and Lonp1 cell localization was assessed (Supplementary Fig. 3A). No changes were observed in the nuclear fraction of Lonp1 in thymidine treated cells compared to untreated cells. This observation ruled out the involvement of Lonp1 in cell cycle regulation.

Since Lonp1 is able to bind to DNA within the mitochondria, we next investigated whether Lonp1 was recruited to the nucleus due to DNA damage response (DDR)^1^. To examine the effects of double-strand breaks on Lonp1 expression, SW620 cells were exposed to 5 Gy of ionizing radiation (IR) and left to recover for 24 h. We first confirmed that DNA damage occurred in our model, by analysing the levels of γ-H2AX, a key player in of the DDR in irradiated cells. As shown in Supplementary Fig. 3B, the γ-H2AX protein level was significantly increased in irradiated SW620 cells compared to untreated cells, and the presence of the γ-H2AX foci was also confirmed (left panel). However, irradiated cells did not show any changes in Lonp1 subcellular distribution when compared to untreated cells, suggesting that nuclear Lonp1 is not related to a DNA damage response (Supplementary Fig. 3B, right panel). These results were confirmed using DNA damage chemicals, inducing hydroxyurea (HU) or doxorubicin (DOXO). Cells were treated with increasing concentrations of these molecules for varying exposure times, and the levels of γ-H2AX were analysed. We found that treatment with 4 mM HU or 2 μM DOXO led to γ-H2AX increase, and to the appearance of γ-H2AX foci in the nucleus (Supplementary Fig. 3C). Therefore, under these conditions the levels of Lonp1 in nuclear and cytosolic fractions from cells were measured, but no changes in Lonp1 subcellular distribution were observed (Supplementary Fig. 3C, right panel). Despite their diverse mechanisms of action, these stressors had no effect on Lonp1 subcellular localization, clearly indicating that DNA damage does not induce Lonp1 localization.

**Figure 3:**
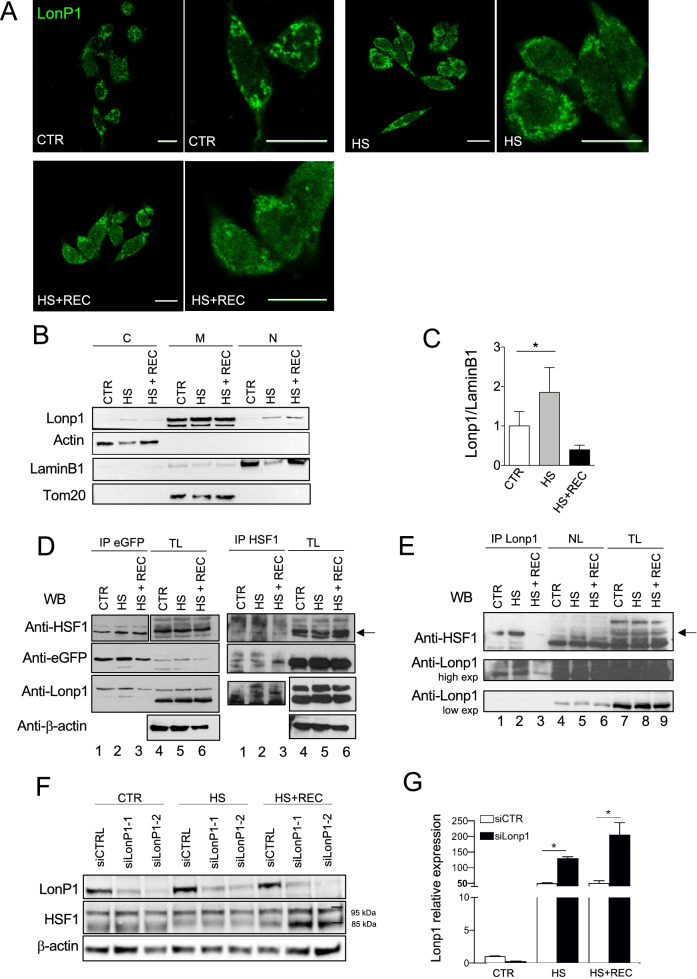
Lonp1 relocates in the nucleus in response to heat shock. (**A**) Representative confocal microscopy images of SW620 cells after immunostaining with anti-Lonp1 antibody. Cells were kept at 37 °C (CTRL), heat-shocked (HS) at 42 °C and then left to recover (HS + REC). Bars: 10 mm. (**B**) Representative immunoblot showing Lonp1 expression in cytosolic, mitochondrial and nuclear fractions from SW620 cells maintained at 37 °C (CTR), at 42 °C for 3 h to induce heat shock (HS) and kept at 42 °C for 3 h and then left for 1 h at 37 °C to recover (HS + REC). β-actin is the cytosolic fraction loading control, TOM20 is the mitochondrial fraction loading control and lamin B1 is the nuclear fraction loading control. Blots were cut prior to hybridisation with antibodies during blotting. (**C**) Histogram representing the relative levels of Lonp1 in nuclear fractions, obtained from three independent experiments. Data are shown as mean SD. *p < 0.05. (**D**) Representative immunoprecipitation experiment showing the interaction between Lonp1-eGFP and HSF1 in SW620 cells after heat-shock (HS) and then left 3 h at 37 °C to recover (HS + REC). Left panels: lysates were immunoprecipitated with anti-eGFP and immunoblotted with anti-HSF1. Western blot on total lysate (TL) is also shown. Blots were cut prior to hybridisation with antibodies during blotting. Right panels: lysates were immunoprecipitated with anti-HSF1 and immunoblotted with anti-eGFP. Blots were cut prior to hybridisation with antibodies during blotting. (**E**) Representative immunoprecipitation experiment showing the interaction between endogeneous Lonp1 and HSF1 in nuclear lysates from SW620 cells after heat-shock (HS) and then left 3 h at 37 °C to recover (HS + REC). Nuclear lysates were immunoprecipitated with anti-Lonp1 and immunoblotted with anti-HSF1. Western blots on nuclear lysates (NL) and total lysates (NL) are also shown. Blots were cut prior to hybridisation with antibodies during blotting. (**F**) Representative immunoblot showing the quantification of HSF1 in SW620 cells transfected with scramble small-interfering RNAs (siCTRL) and cells transfected with small-interfering RNAs against Lonp1 (siLonp1-1 and siLonp1-2), after HS and HS + REC. Blots were cut prior to hybridisation with antibodies during blotting. (**G**) Quantification of the mRNA levels of HSP70 in SW620 cells transfected with scramble small-interfering RNAs (siCTRL) and cells transfected with small-interfering RNAs against Lonp1 (siLonp1), after HS and HS + REC. *P < 0.05.

An alternative hypothesis was that Lonp1 translocates to the nucleus in response to proteotoxic stress. As heat shock is commonly used to induce proteotoxic stress^17^, we kept SW620 cells for up to 3 h at 42 °C and then left them to recover. We then analysed Lonp1 expression and distribution and observed that there was a clear localization of Lonp1 in the nucleus of treated cells compared to an insignificant presence in the nucleus of control cells (Fig. 3A). We then analysed the expression of Lonp1 in nuclear and cytosolic fractions under HS compared to controls. Lonp1 expression in the same fraction showed a twofold increase of the protein in the nucleus of treated cells when compared to controls, indicating that HS induces Lonp1 relocalisation to the nucleus (Fig. 3B, C). Treatment of cells under HS, or HS plus recovery, with 50 nM LMB enhanced the nuclear localization of Lonp1, further suggesting that Lonp1 translocates to the nucleus under HS (Supplementary Figs. 4A and 4B).

We hypothesized that Lonp1 in the nucleus may contribute to regulate HS response (HSR). In mammalian cells, HSR is regulated by heat shock factor-1 (HSF1)^40^. While HSF1 is mostly located in the cytosol and nucleus of unstressed cells, it accumulates in the nucleus in heat-shocked cells and promotes the transcription of HSR genes. Thus, we assumed that Lonp1 may interact with HSF-1 and modulate its function in the nucleus. We first looked for an interaction between Lonp1 and HSF1. Therefore, SW620 cells were transfected with a full-length form of Lonp1 tagged with eGFP at C-term, heat-shocked for 3 h, and left to recover. Protein extracts were pulled down with GFP Trap Agarose beads and assayed with anti-HSF1 Ab (Fig. 3D, left panels), showing that under normal conditions HSF1 physically interacts with Lonp1 (lane1) , and that HS increased the binding of Lonp1 to HSF1 (lane2). The interaction has been observed also when an anti-Lonp1 Ab has been used to pull down the endogenous protein in the absence of eGFP-tagged protein, excluding a possible artefact (Supplementary Fig. 5). When we pulled down HSF-1 on the cells expressing Lonp1-eGFP with anti HSF-1, we could reveal the presence of Lonp1 in the immunoprecipitate with anti-eGFP Ab, thus confirming the specificity of the interaction (Fig. 3D, right panels lanes 1–3). Then, we pulled down HSF-1 in SW620 cell extracts, and we immunoblotted the immunoprecipitate with anti Lonp1 Ab. We detected the presence of a band at the MW of Lonp1 protein, with a higher intensity in samples from cells under HS and HS + REC than control (Supplementary Fig. 5B). To verify if the interaction occurred specifically in the nucleus or not, SW620 cells were heat-shocked for 3 h, and left to recover, and nuclear extracts were pulled down with anti-Lonp1 and assayed with anti-HSF1 Ab. HSF1 physically interacts with Lonp1 in the nucleus, and HS increased the binding of Lonp1 for HSF1 (Fig. 3E). Knock-down of Lonp1 followed by HS and recovery determined an increase in the levels of HSF1 in the nucleus (Fig. 3F), suggesting that Lonp1 can modulate HSF1 levels, likely by degrading it. In addition, when Lonp1 was silenced by small interfering RNA (siRNA), the levels of heat shock protein-70 (HSP70) increased both during HS and recovery (Fig. 3G).

As Lonp1 interacted with HSF1, we hypothesized that this interaction could impact on the transcriptional activity of HSF1 during heat shock response and modulate the expression of HSF1 target genes. To test this hypothesis, we performed an RNAseq analysis on SW620 cells with silenced Lonp1 undergoing HS, or HS followed by recovery and compared them to SW620 cells treated with a scramble siRNA, in the same conditions. As PCA and cluster analysis using the top 10% variable genes showed a close similarity between HS and HS plus recovery samples (Supplementary Fig. 5), we focused our attention only on HS.

**Figure 4:**
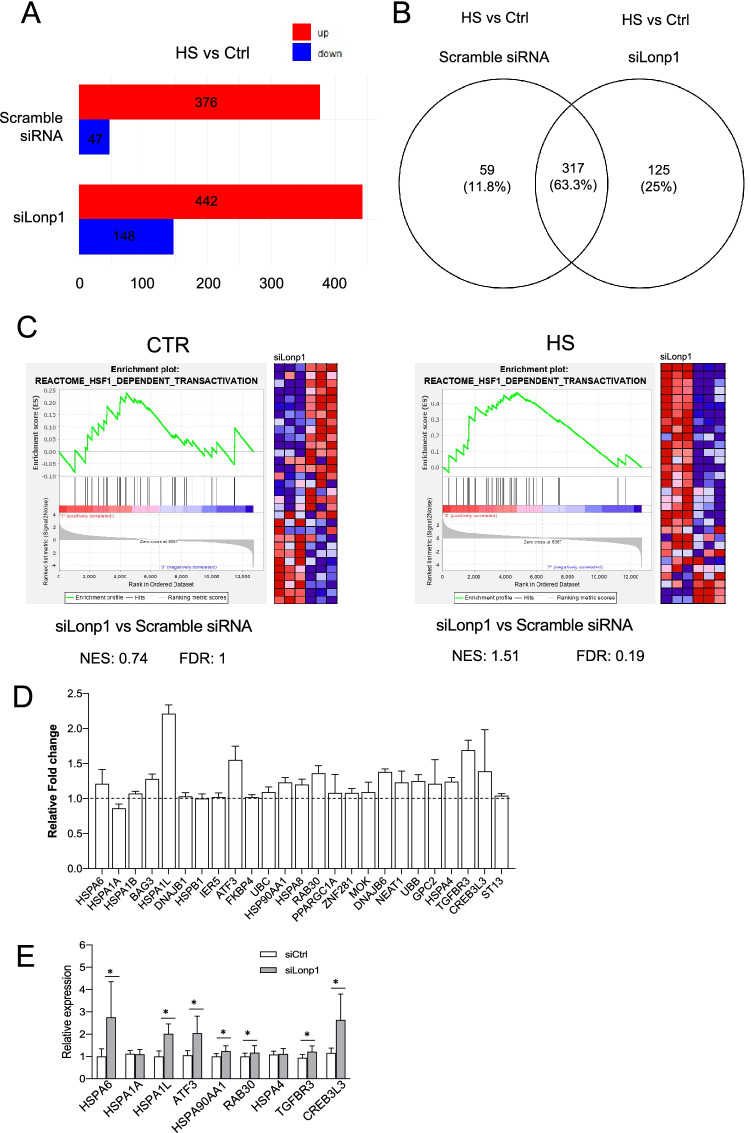
Silencing of Lonp1 enhances HSF1-mediated response to HS. (**A**) Number of genes significantly up- or down-regulated in SW620 cells transfected with small-interfering RNAs against Lonp1 (siLonp1) or with a scramble siRNA, kept at 37 °C (Ctrl) or at 42° for 1 h (HS). FDR q value ≤ 0.05 was used to identify differentially expressed genes, with fold change (FC) > 2 for up-regulated and FC < 2 for down-regulated genes. (**B**) Venn diagram showing the overlap between genes upregulated in HS, in cells where Lonp1 was silenced (siLonp1) or not (Scramble) in SW620 cells. (**C**) Left panel: Gene set enrichment analysis (GSEA) plots for the “Reactome—HSF1 dependent transactivation” signature in SW620 cells transfected with small-interfering RNA against Lonp1 (siLonp1) in comparison with a scramble siRNA, kept at 37 °C. Right panel: Gene set enrichment analysis (GSEA) plots for the “Reactome—HSF1 dependent transactivation” signature in SW620 cells transfected with small-interfering RNA against Lonp1 (siLonp1) in comparison with a scramble siRNA, kept at 42° for 1 h. The Normalized enrichment score (NES) and FDR values for both analyses are reported. (**D**) Ratio between fold change observed after HS in cells treated with siLonp1, and fold change observed after HS in cells treated with a scramble siRNA. Twenty-five HSF1 target genes are shown; data are calculated from RNAseq data set, and expressed as mean ± SD. (**E**) Relative expression of nine selected HSF-1 target genes in cells in cells treated with siLonp1 after HS. Data have been normalized to the expression observed after HS in cells treated with a scramble siRNA, set arbitrarily to one. Data are the mean ± SD of two independent experiments, each in triplicate. *p < 0.05.

We first analyzed the changes in the gene expression caused by HS. In this condition, 442 genes were upregulated and 148 downregulated in cells treated with siLonp1 when compared to cells kept at 37 °C. In cells treated with a scramble siRNA, HS determined the upregulation of 376 genes and the downregulation of 47 genes (Fig. 4A). Genes upregulated during HS in Lonp1-silenced cells were largely overlapping with those upregulated in cells treated with a scramble siRNA, but 125 of them (25%) are uniquely upregulated when Lonp1 was silenced, indicating that silencing of Lonp1 modified HS response (Fig. 4B). As expected, gene ontology analysis revealed that, when compared to control samples, genes upregulated in HS were significantly enriched in “response to unfolded protein”, “protein refolding” and “regulation of cellular response to heat” biological processes, either in cells treated with a Lonp1 siRNA or with a scrambled, control siRNA (Supplementary Table 1). Then, we focused our attention on a possible effect of Lonp1 on HSF1 downstream genes. Gene ontology revealed a significant enrichment of genes related to “regulation of HSF1 mediated HS response” “HSF1 dependent transactivation” and “HSF1 activation” pathways (Supplementary table 2) after HS. We performed a Gene Set Enrichment Analysis (GSEA) to determines whether the set of genes that are target of HSF1 showed a statistically significant, concordant difference between cells where Lonp1 was present or not (Fig. 4C and supplementary Table 3). Samples kept at 37 °C did not show any relevant enrichment of HSF1 target genes when Lonp1 is silenced if compared to samples treated with scrambled siRNA, suggesting that in normal conditions, Lonp1 does not have any relevant effect on HSF1 (Fig. 4C, left panel). Conversely, HS determined a slight, but significant enrichment of HSF1 target genes in Lonp1-knocked down cells (Fig. 4C, right panel). To confirm this observation, we compared the fold change in the expression of HSF1 target genes caused by HS between cells where Lonp1 was silenced or not. HSF1 target genes showed a slightly higher expression in samples under HS where Lonp1 was silenced, if compared to samples under HS treated with a scrambled siRNA, with the only notable exception of HSPA1A (Fig. 4D)^18^. We selected 9 HSF1 target genes, and confirmed this observation by qRT-PCR (Fig. 4E). This suggests that the absence of Lonp1 determined a stronger response to HS, mediated by HSF1. Overall, these data indicate that Lonp1 knock down leads to a further increase of HSF1 target genes at the transcriptional level in response to HS and suggest that Lonp1 in the nucleus interacts with HSF1, likely modulating the HSR.

## Discussion

Lonp1 is a nuclear-encoded mitochondrial enzyme, previously assumed to modulate several cellular functions, exclusively in the mitochondria. Inactivation of Lonp1 by small interfering RNA (siRNA) or by specific inhibitors, has been proven to lead to defective respiration, alteration of mitochondrial ultrastructure and inhibition of mitochondrial translation^6,19,20^. For the first time, this study demonstrates that Lonp1 is also expressed in the nucleus and that its relocalisation is in response to HS.

As no other mitochondrial protease has been observed in the nucleus, the localization of Lonp1 in this study was surprising but not totally unexpected, for several reasons. Firstly, an extra-mitochondrial form of Lonp1 has already been demonstrated by Polo et al., who identified mitochondrial associated membranes (MAMs) as a site where Lonp1 localizes in the presence of ER-stress, clearly indicating that Lonp1 is not exclusively mitochondrial^15^. Independent observations in other cellular and animal models have also confirmed this observation^21,22^. Secondly, several mitochondrial proteins have been shown to relocate to the nucleus in response to different stimuli, and to regulate a plethora of functions. The monooxygenase CLK-1 is a mitochondrial enzyme involved in respiration and longevity. However, a nuclear, independent form of this protein exists and mediates a retrograde signal in response to mitochondrial Reactive Oxygen Species (ROS). Similarly, ATFS-1, a transcription factor that regulates mitochondria-to-nuclear communication during the mt Unfolded protein response (UPR), can move to mitochondria and, in condition of mitochondrial stress, to the nucleus, where it regulates the transcription of OXPHOS related genes. Although the behaviour of Lonp1 is very similar to that of CLK-1 and ATFS-1, a crucial difference exists; both the nuclear and mitochondrial form of Lonp1 is identical whereas the nuclear CLK-1 and ATFS-1 (include the MTS) and therefore differ from the mitochondrial forms of the respective proteins. Therefore, Lonp1 most resembles the mitochondrial pyruvate dehydrogenase complex (PDC); a complex of three enzymes catalysing the conversion of pyruvate into Acetyl-CoA, which has been shown to translocate from the mitochondrial matrix to the nucleus during cell cycle progression, in response to growth signals, such as serum or epidermal growth factor.

We have also established that Lonp1 can localize in the nucleus in response to HS. Our proposal of a link between Lonp1 and HS is not without precedent, as several works have already demonstrated a complex interplay between them in prokaryotes. Lonp1 is highly conserved throughout evolution, and was first described in *E. coli*, where it is known as *La* protease. Its roles in this species are multiple, spanning from protein quality control, radiation resistance, filamentation, capsular polysaccharide production, cell division, and survival under starvation conditions^5^. Over the past decade, a role for bacterial Lon in proteotoxic stress response has also been described^23–25^. For example, in *Caulobacter crescentus*, the proteotoxic stress induces cell cycle arrest by triggering the degradation of the replication initiator DnaA by Lon^24^. In *Yersinia pestis,* HS protein Q (hspQ), which is induced by HS, regulates the substrate preference and catalytic efficiency of Lon^25^. Thus, our study demonstrates that this link is maintained in eukaryotes, and particularly in mammalian cells. In this study, we report the translocation of Lonp1 to the nucleus when cells are exposed to HS, but not to other stress stimuli, such as genotoxic stress or cell cycle block. Previous reports have indicated that Lonp1 is stress-responsive and is upregulated in the presence of HS^26^; our observation further expands the importance of Lonp1 as a stress response protein, and its range of actions in the human cell.

It is theoretically possible that the nuclear increase of Lonp1 in the presence of HS could be caused by the direct import of the neo-synthesized protein from the cytoplasm into the nucleus. However, we tended to exclude these possibilities, for several reasons. As HS does not modify the transcription of the plasmid-encoded protein, the relative increase in the nuclear fraction form can only be due to a relocation of the mitochondrial form. Furthermore, the MW of the protein present in the nuclear fraction is identical to that present in the mitochondria, suggesting that MTS is cut away from the enzyme when it relocates into the nucleus. The way Lonp1 is exported from mitochondria to the nucleus is still unknown. An ongoing open question is the nature of the interaction between Lonp1 and HSF1. As Lonp1 displays a proteolytic activity, we are tempted to speculate that Lonp1 can contact HSF1 to degrade it, thereby modulating HSR.

Mutations of LONP1 gene are associated with Cerebral Ocular Dental Auricular Skeletal Anomalies Syndrome (CODAS), a complex multisystemic and developmental disorder^5,10^. The pathogenetic link between LONP1 mutations and the signs and symptoms of the disease are still elusive. It could be of great interest to understand if the Lonp1 mutants observed in CODAS patients display an aberrant nuclear localization, and if this difference can contribute to the development of the anomalies observed in CODAS patients.

## Methods

All experiments and methods were performed in accordance with relevant guidelines and regulations. All methods are reported in accordance with Animal Research: Reporting of In Vivo Experiments (ARRIVE) guidelines.

### Mice

*Lonp1*^*wt/–*^ mice were generated with a knock-out allele encompassing exons 5 and 8 from Biogem Srl (Ariano Irpino, Italy) on a C57BL/6 background^27^. The genotyping was performed on genomic DNA extracted from ear samples and obtained using the commercial Easy-DNA™ gDNA Purification Kit (Thermo Fisher Scientific, Waltham, MA, USA). 20–50 ng of DNA were amplified in 25 μL of a reaction mix containing 1X GoTaq Flexy Buffer (Promega Corporation, Madison, WI, USA), 2 mM MgCl_2_, 0.2 mM of each dNTP (Promega), 0.03 U of GoTaq G2 Flexy DNA Polymerase and 0.2 μM of the following primers: forward 5-CAGGGAAGAAACTGAAGTCAGGC-3, reverse 5-CACTCTGGTTCATGGCCACC-3. A 925 bp PCR product was obtained only for knock-out allele. All mice were housed in ventilated cages for a 12-h day/night cycle with access to water and food ad libitum. All animal experiments and protocols were performed in accordance with ARRIVE guidelines and approved by the Italian law protecting animals used for scientific purposes (authorization n°253/2017-PR, released on March, 21st, 2017 from Italian Ministry of Health).

### Cell culture

SW620 were maintained in Glutamax RPMI 1640 supplemented with gentamycin and 10% foetal bovine serum (FBS). SW620 and SW480 cell lines were kindly gifted by Dr. Zanocco Marani. HeLa cell line was kindly gifted by Prof. Andrea Cossarizza. SW48 cell line was purchased from ATCC. SW480 and HeLa cells were maintained in Glutamax Dulbecco modified Eagle medium (DMEM) supplemented with gentamycin and 10% FBS (Life Technologies). Cells were maintained in an incubator at 37 °C, 5% CO2, in a humidified atmosphere. To induce heat shock, cells were incubated in a water bath set at 42 °C.

### Retroviral transduction

The pMSCV-Puro empty vector and the pMSCV containing the cDNA encoding for the double mutant R237A/R241A Lonp1 protease were used to transiently transfect amphotrophic Phoenix cell line, as described in^8^. Retroviral supernatants were used to stably transfect SW620 cells, and stable transfectants were selected by using 4 μg/ml puromycin, and then maintained in cell medium supplemented with 2 μg/ml puromycin.

### RNA interference

Cells were reverse transfected by using RNAiMAx (Thermo Fisher) and 100 nM of two pre-validated siRNAs (s17901 and s17902, Thermo Fisher) against Lonp1 mRNA. After 72 h of incubation, cells were collected and lysed by using RIPA buffer.

### Immunofluorescence and confocal microscopy

Cells for confocal microscopy were grown on coverslips, fixed with 4% paraformaldehyde (PFA, Sigma Aldrich) in PBS, for 10 min, and permeabilized with 0.1% triton X-100 in PBS, for 15 min. Cells were blocked in 3% bovine serum albumin (BSA) for 1 h, incubated with primary antibodies overnight, washed, incubated with Alexa Fluor-conjugated secondary antibodies (Life Technologies) in 3% BSA for 1 h, and washed again to remove unbound antibody. Cells were counterstained with DAPI. Coverslips were mounted in Fluoromount (Sigma Aldrich) and images were collected on a confocal microscope SP8-AOBS (Leica). Image analysis was performed with ImageJ and ScanR.

### Western blotting

Western blotting was performed as previously described^6^. Total cell lysates were prepared in RIPA lysis buffer plus protease inhibitors cocktail (Sigma Aldrich) and phosphatase inhibitors (Sigma Aldrich). Nuclear fractions were obtained by using NE-PER nuclear and cytoplasmic extraction kit and following manufacturer’s instructions. Nuclear, mitochondrial and cytosolic fractions were isolated by using the Cell fractionation kit (Abcam, Cambridge, UK), following provided instructions. Samples were resolved by SDS-PAGE on precast gels (12%, 4–12%) and transferred to nitrocellulose membranes (Bio-Rad Laboratories), which were then immunoblotted. Blots were cut prior to hybridisation with antibodies during blotting. The following primary antibodies were used: anti-Lamin B1 (Santa Cruz Biotechnology), anti-β-actin (Abcam), anti-TOM20 (Santa Cruz Biotechnology), anti-SIRT3 (Santa Cruz Biotechnology), anti-HSF1 (Abcam). Lonp1 was probed using a custom anti-Lonp1 polyclonal antibody (Primm, Milan, Italy) to the His-Tag recombinant protein Lonp1 (from aa 376 to aa 497; QQR- LGREVEEKIKQTHRKYLLQEQLKIIKKELGLEKDDKDAIEEKFRERLKELVVPKHVMDVVDEELSKLGLLDNHSSEFNVTRNYLDWLTSIPWGKYSNENLDLARAQAVLEEDHYGMEDV)^6,8,28^ (Supplementary Fig. 7). A second, validated anti-Lonp1 antibody has been used (Sigma Aldrich antibody n. HPA002034), when indicated. The following secondary antibodies were used: HRP-conjugated goat anti-rabbit and HRP-conjugated goat anti-mouse (Bio-Rad Laboratories). Enhanced Clarity chemiluminescent substrate (Bio-Rad laboratories) was used to detect proteins by using a Chemidoc MP (Bio-Rad Laboratories). Image analysis was performed by Image Lab software v5.2.1.

### Immunoprecipitation

Briefly, 800 ugr of total lysate (TL) or nuclear lysate (NL) from nuclear fraction in a RIPA modified Buffer (20 mM Tris–Cl, pH 7.0; 1% Nonidet P-40; 150 mM NaCl; 10% glycerol; 10 mM EDTA; 20 mM NaF; 5 mM sodium pyrophosphate; and 1 mM Na3VO4) was precleared with 10 μl of 50% (v/v) protein A/G agarose (GEHealthcare, Little Chalfont, United Kingdom). Subsequently, supernatant from precleared beads was incubated with: GFP Trap Agarose beads (Chromotek, Germany), or anti-HFS1 antibody, or anti Lonp1 antibody (ratio: 0.5μgr of antibody/500μgr of total protein) overnight at 4 °C with constant rotation. In immunoprecipitation for HSF1 or Lonp1, 30 μl of 50% (v/v) protein A/G agarose (GEHealthcare, Little Chalfont, United Kingdom) were added and incubated for 1 h with agitation. All immunoprecipitation beads were washed three times with wash buffer (20 mM Tris–Cl, pH 7.0, 1% Nonidet P-40, 150 mM NaCl, 10% glycerol, 10 mM EDTA, 20 mM NaF, 5 mM sodium pyrophosphate), and once with 10 mM Tris–Cl, pH 7.4. The target proteins bound were eluted from beads by boiling in SDS loading buffer for 5 min at 99 °C^29^.

### Histology and immunohistochemistry

Histological sections of mice colon (n = 3) were deparaffinized in xylene and rehydrated in alcohol upon distilled water. Antigen retrieval was performed by incubating slides in 10 mM sodium citrate buffer, pH 6.0, at sub-boiling temperature for 15 min. Then, endogenous peroxidase activity was blocked by incubating sections in a 5% methanol plus 1% hydrogen peroxide solution for 5 min. After blocking with PBS containing 3% BSA, sections were incubated overnight at 4° C with the primary rabbit anti-Lonp1 antibody diluted 1:100 in PBS containing 3% BSA. Then, after 3 washings, HRP conjugated secondary antibody was incubated (diluted 1:200 in PBS) for 1 h at room temperature. Finally, after 3 washings in PBS, HRP was revealed by a DAB based kit (Sigma Aldrich), according to manufacturers' instructions. Tissues were obtained from the Department of Diagnostic and Clinical Medicine, and Public Health, University of Modena and Reggio Emilia, through an institutional review board-approved protocol.

### Flow cytometry

Cell cycle was analysed by flow cytometry. In brief, cells were treated with 10 μM 5-ethynyl-2´-deoxyuridine (EdU) for 2 h. Cells were detached and immediately stained using to Click-iT™ EdU Alexa Fluor™ 488 Flow Cytometry Assay Kit (Thermo Fisher Scientific), following manufacturer protocol/standard procedures. Fixed cells were acquired by Attune NxT acoustic flow cytometer (Thermo Fisher Scientific) equipped with four lasers (405 nm, 488 nm, 561 nm, 638 nm). A minimum of 10,000 events were acquired for every sample^30^.

### RNAseq

SW620 cells exposed to heat shock for and RNA extracted using RNeasy mini kit (QIAgen) following provided instructions. Three independent biological replicates were examined. The quality of RNA samples was checked by using an Agilent 2100 Bioanalyzer (Agilent, Santa Clara, CA, USA); RNA samples were used either for RNAseq and for qPCR validation. RNAseq library was prepared from 1.5 µg of total RNA using TruSeq Stranded mRNA Library Prep Kit in paired end format (Illumina) following the manufacturer’s recommendations. RNA sequencing was carried out on Illumina Novaseq 6000 (Illumina) at BMR Genomics (Padua, Italy).

### Quantitative real time PCR

For relative expression of the genes CREB3L3, HSPA1L, TGFBR3, ATF3, RAB30, HSPA4, HSP90AA1, HSPA1A, HSPA6 has been quantified with a set of wet-lab pre-validated oligos (Bio-Rad). Real-time PCR was performed in triplicate, using the SsoAdvanced Universal SYBR Green Supermix (Biorad) following the suggested reaction protocol and thermocycling parameters, in a CFX96 Touch instrument (Bio-Rad). Transcript of ribosomal protein S18 (RPS18) has been used as reference gene. Changes in the gene expression have been calculated through ΔΔ-cycle method.

### Bioinformatics analysis

Raw reads were aligned using STAR version 2.7.3a to build version hg38 of the human genome^31^. Counts for UCSC annotated genes were calculated from the aligned reads using featureCounts function of the Rsubread R package^32^ and R (version 3.6.3). Normalization and differential analysis were carried out using edgeR R package^33^. Raw counts were normalized to obtain Counts Per Million mapped reads (CPM). Principal component analysis (PCA) was performed using the top 10% variable genes and the prcomp function of stats R package. Only genes with a CPM greater than 1 in at least three samples were retained for differential analysis. Gene expression changes were considered statistically significant with Benjamini–Hochberg FDR less than 5% and fold changes greater than 2 for upregulation and lower than − 2 for downregulation.

Functional enrichment analysis of the differentially expressed genes was performed using Enrichr (https://maayanlab.cloud/Enrichr/) and DAVID (Version 6.8, Oct 2016; https://david.ncifcrf.gov/home.jsp)^34–37^. Gene set enrichment analysis version 4.2.0^38^ was performed on the log2 TMM values calculated by edgeR and the Reactome collection of Molecular Signature Database (MSigDB) version 7.2^39^. Gene sets were considered significantly enriched at FDR ≤ 5% when using 1000 permutations of gene sets. RNA-seq data from this study have been deposited at Gene Expression Omnibus database (GEO, https://www.ncbi.nlm.nih.gov/geo/) with accession number GSE164834.

### Statistical analysis

All the measurements data are presented as mean ± standard deviation (SD) if not differently specified. Statistical analysis between different experimental conditions was performed with ANOVA followed by the Bonferroni means comparison or *t* test when appropriate. A threshold of *P* ≤ 0.05 was selected to indicate statistical significance. Statistical calculations were performed using standard functions of GraphPad Prism 8.0. Image analysis was performed by ImageJ v2.0 and ScanR.

## Supplementary Information

Supplementary Figures.

Supplementary Legends.

Supplementary Table 1.

Supplementary Table 2.

Supplementary Table 3.

## Data Availability

RNA-seq data from this study have been deposited at Gene Expression Omnibus database (GEO, https://www.ncbi.nlm.nih.gov/geo/) with accession number GSE164834.
