# Supplementary material for: Evidence for mitochondrial Lonp1 expression in the nucleus
Source: Sci Rep. 2022 Jun 27;12:10877. doi: 10.1038/s41598-022-14860-0 (PMC9237102; doi:10.1038/s41598-022-14860-0)

Supplementary material

“Evidence for mitochondrial Lonp1 expression in the nucleus”

Gibellini et al

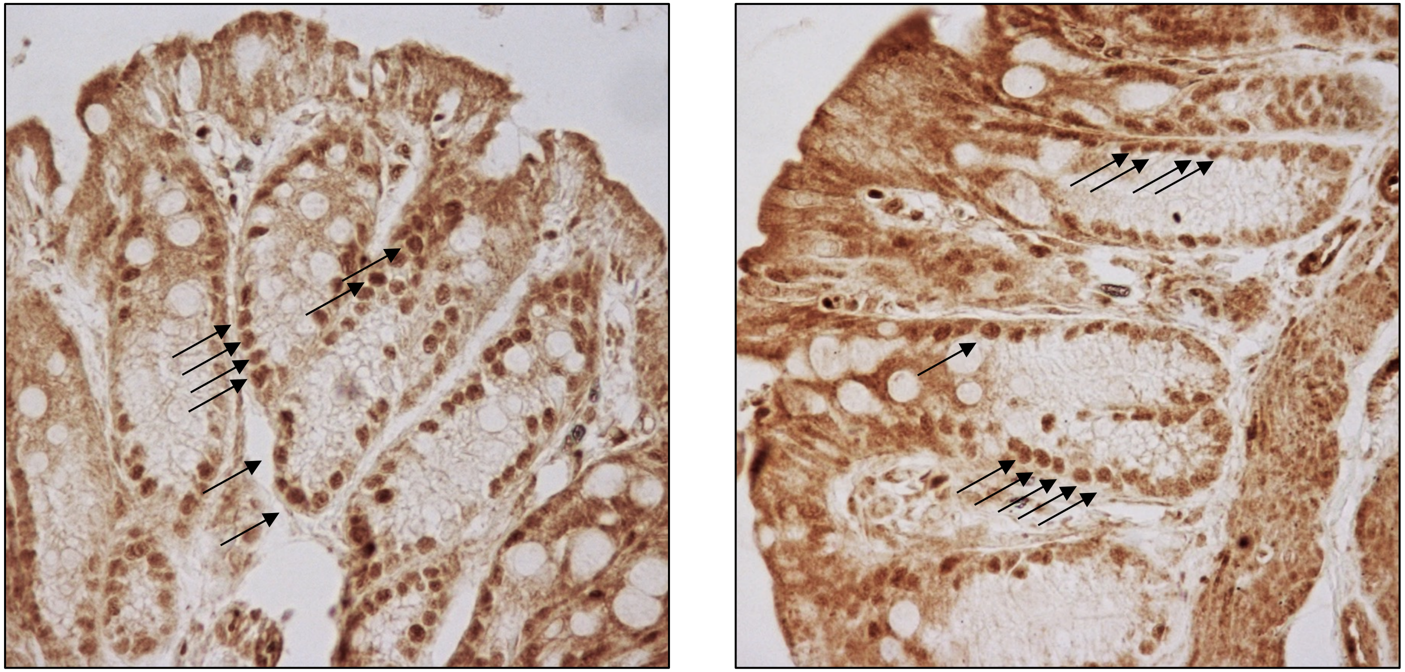

Supplementary Figure 1

Representative images of Lonp1 IHC on murine colon tissue, showing Lonp1 nuclear localization. Cells have been labelled with anti Lonp1 Ab from Sigma Aldrich (Cat. n. HPA002034) and revealed by an HRP-conjugated goat anti-rabbit Ab. Positive nuclei are indicated by arrows.

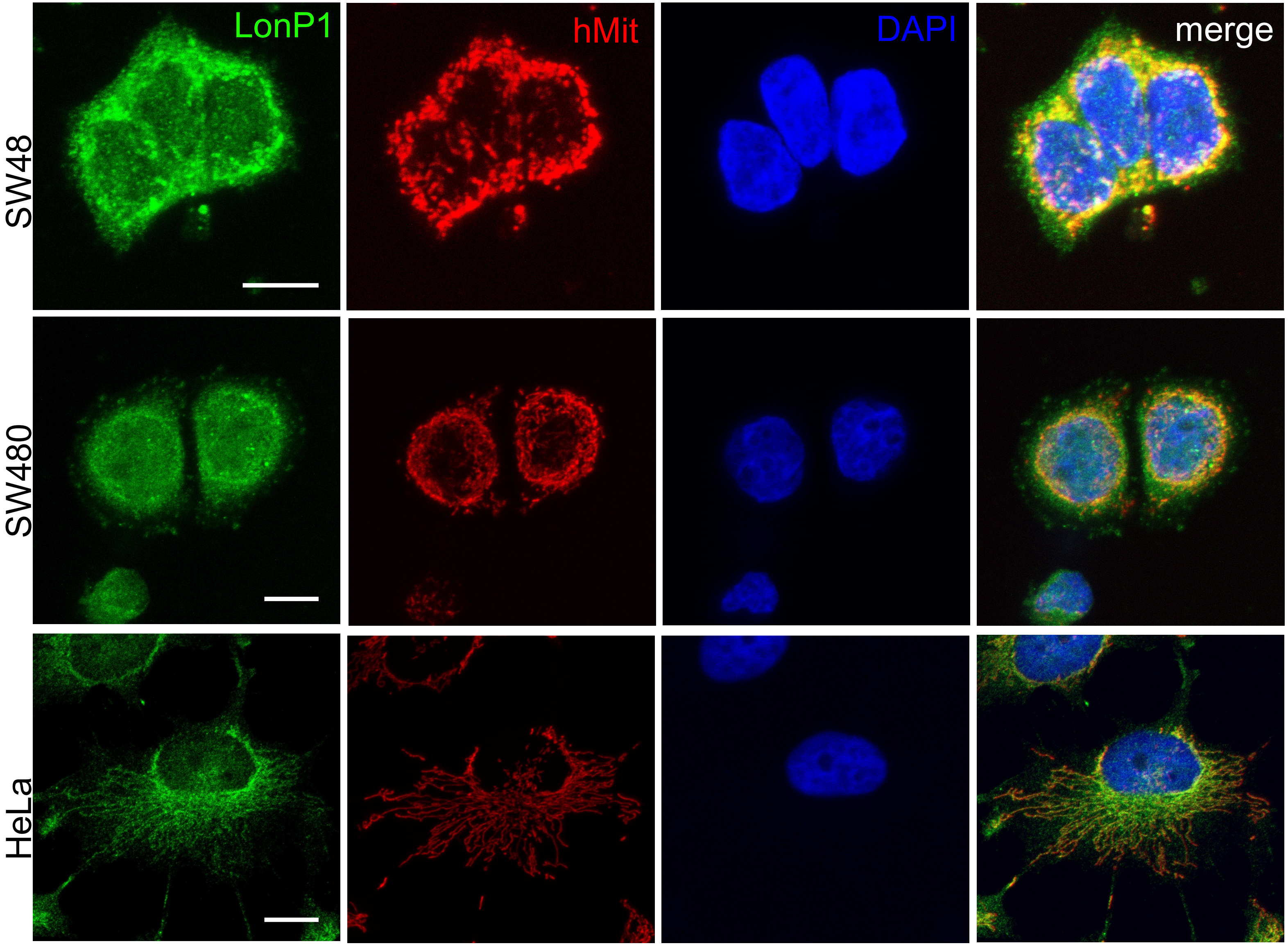

Supplementary Figure 2. Lonp1 localizes in the nucleus in SW48, SW480 and SW48 cells.
Representative confocal microscopy images of SW48, SW480 and HeLa cells after immunostaining with anti-Lonp1 and anti-human mitochondria (hMit) antibodies. Nuclei were counterstained with DAPI.

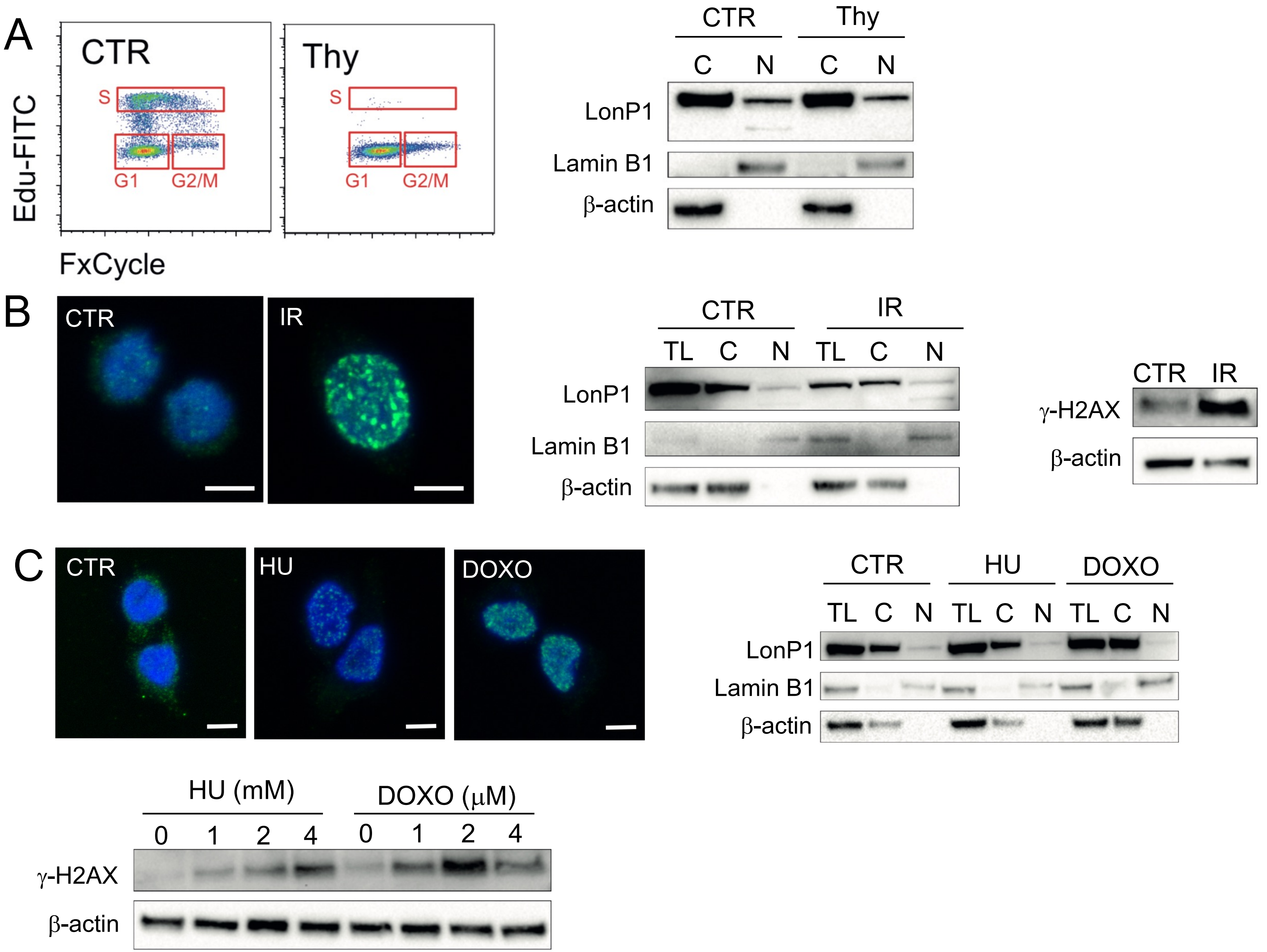

Supplementary Figure 3. DNA damage or cell cycle do not influence Lonp1 nuclear expression.
A. Cell cycle analysis of SW620 cells treated or not with thymidine, using 5-ethynyl-2´-deoxyuridine. Cell were treated with thymidine (Thy) to block cell cycle in the G1 phase. Representative immunoblot of cytosolic (C) and nuclear (N) fractions obtained from SW620 cells, treated or not with thymidine. B. Representative confocal microscopy images of SW620 control cells (CTR), and cells irradiated with 5 Gy (IR), immunostained with anti-γ-H2AX. Bars: 10 μm. Representative immunoblot reporting the expression of γ-H2AX in SW620 control cells, and cells irradiated with 5 Gy. C. Representative confocal microscopy images of SW620 control cells (CTR), and cells treated with hydroxyurea (HU) or doxorubicin (DOXO), immunostained with anti-γ-H2AX. Bars: 10 μm. Representative immunoblot of SW620 cells (CTRL), and cells treated with increasing concentrations of HU and DOXO. Right panel: Representative immunoblot of total lysate (TL), cytosolic (cyt) and nuclear (N) fractions obtained from SW620 treated or not with 4 mM HU or 2 μM DOXO.

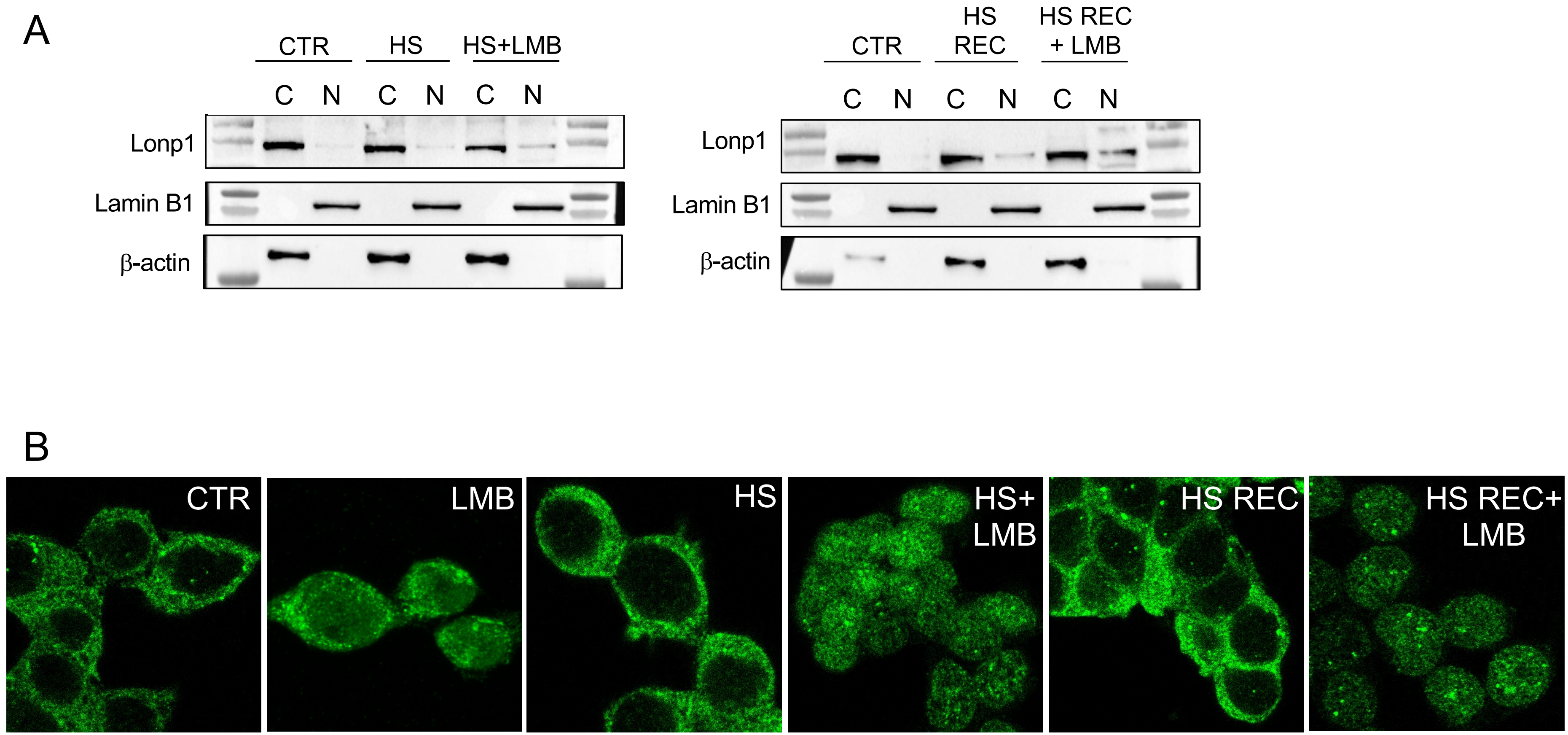

Supplementary Figure 4

A. Immunoblot showing Lonp1 expression in cytosolic (C) and nuclear (N) fractions from SW620 cells maintained at 37°C (CTR), at 42°C for 3 hrs to induce heat shock (HS) and kept at 42°C for 3 hrs and then left for 1 hour at 37°C to recover (HS+REC) in the presence or not of Leptomycin B (LMB) 50 nM. β-actin is the cytosolic fraction loading control and Lamin B1 is the nuclear fraction loading control. B. Confocal microscopy images of SW620 cells kept at 37°C (CTR), at 42°C for 3 hrs to induce heat shock (HS) and kept at 42°C for 3 hrs and then left for 1 hour at 37°C to recover (HS+REC) in the presence or not of Leptomycin B (LMB) 50 nM.

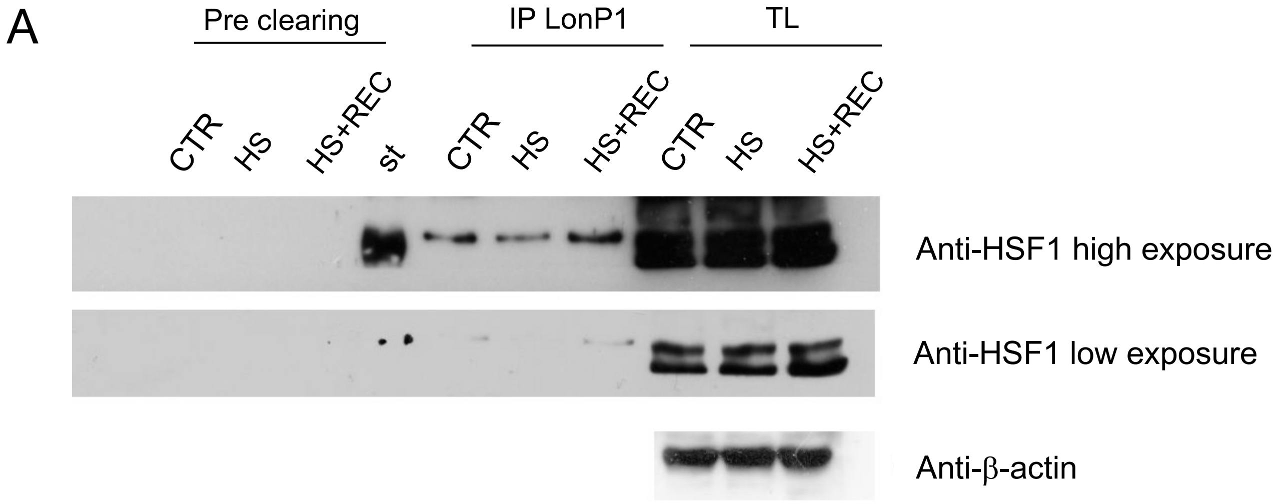

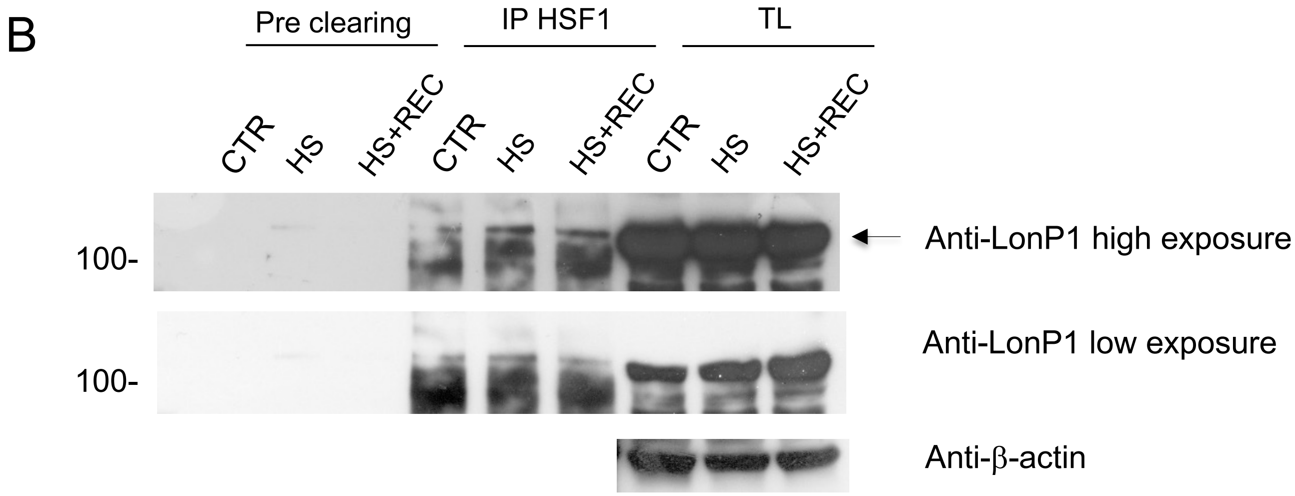

Supplementary Figure 5

A. Immunoprecipitation experiment showing the interaction between endogenous Lonp1 and HSF1 in SW620 cells after 3 hours of heat shock and recovery at 37°C to recover (HS+REC). Immunoprecipitation has been performed using anti-Lonp1 Ab (Sigma-Aldrich), and immunoblotted with anti-HSF1 Ab. Immunoblot on Pre cleared lysate and total lysate (TL) are also shown. B. Immunoprecipitation experiment showing the interaction between HSF-1 and endogenous Lonp1 in SW620 cells after heat-shock (HS) and after 3 hours of heat shock and recovery at 37°C to recover (HS+REC). Immunoprecipitation has been performed using and anti HSF-1 Ab and immunoblotted with anti-Lonp1 (Sigma Aldrich). Immunoblot on precleared lysate and total lysate (TL) are also shown. St=standard.

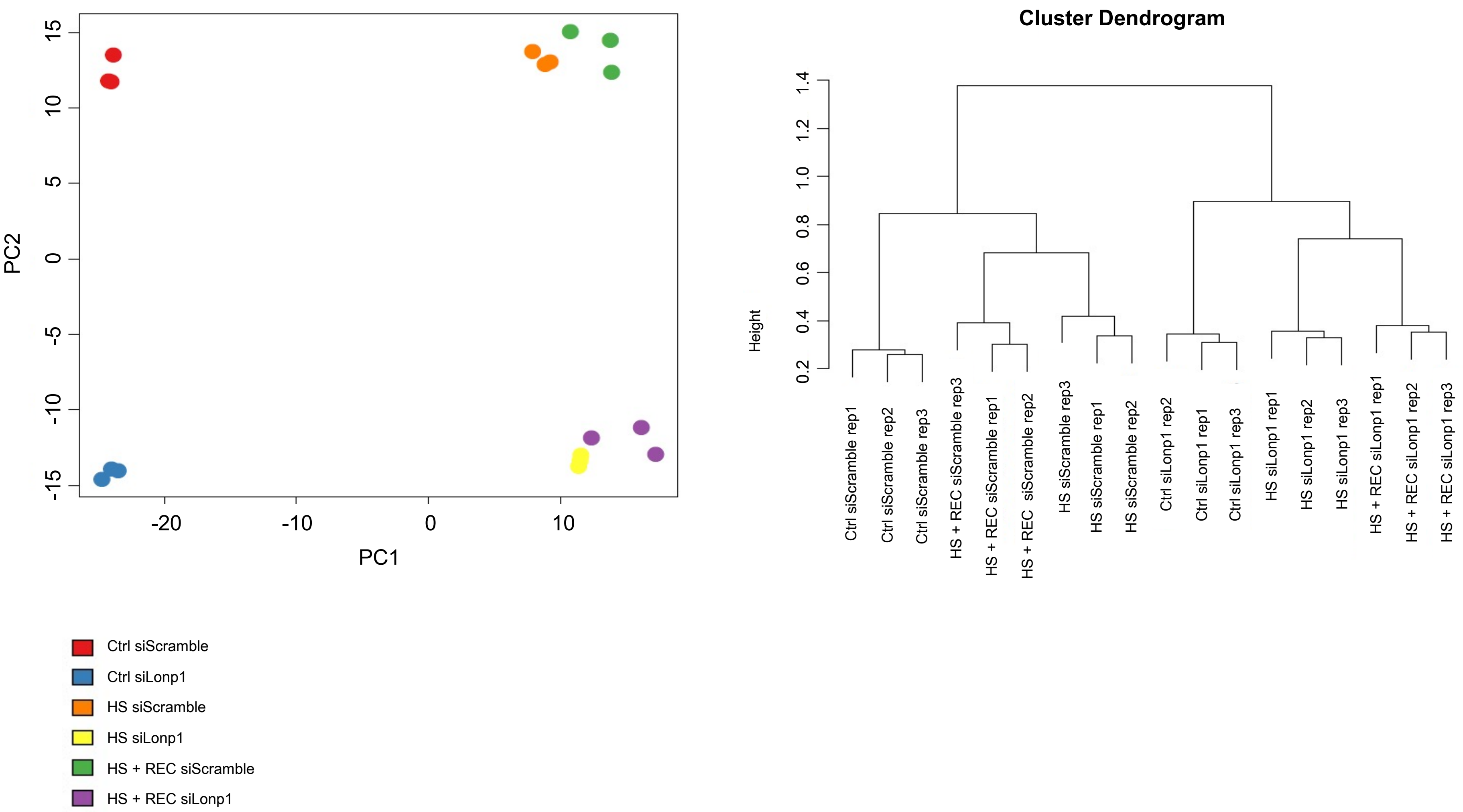

Supplementary Figure 6

Principal component analysis and unsupervised cluster analysis and of RNAseq data from SW620 cells undergoing heat shock for 1 hour, heat shock for 1h followed by 1h hour of recovery, and cells kept at 37°C, when Lonp1 was silenced or not. Ctrl siScramble: cells transfected with a scrambled siRNA, kept at 37°C. Ctrl siLonp1: cells transfected with siRNA for LONP1, kept at 37°C. HS siScramble: cells transfected with scrambled siRNA, kept at 42°C for 1 hour. HS_siLONP1: cells transfected with LONP1 siRNA, kept at 42°C for 1 hour. Hs +REC siScramble: cells transfected with scrambled siRNA, kept at 42°C for 1 hour and left at 37°C for 1 hour to recover. HS + Rec siLonp1: cells transfected with LONP1 siRNA, kept at 42°C for 1 hour and left at 37°C for 1 hour to recover.

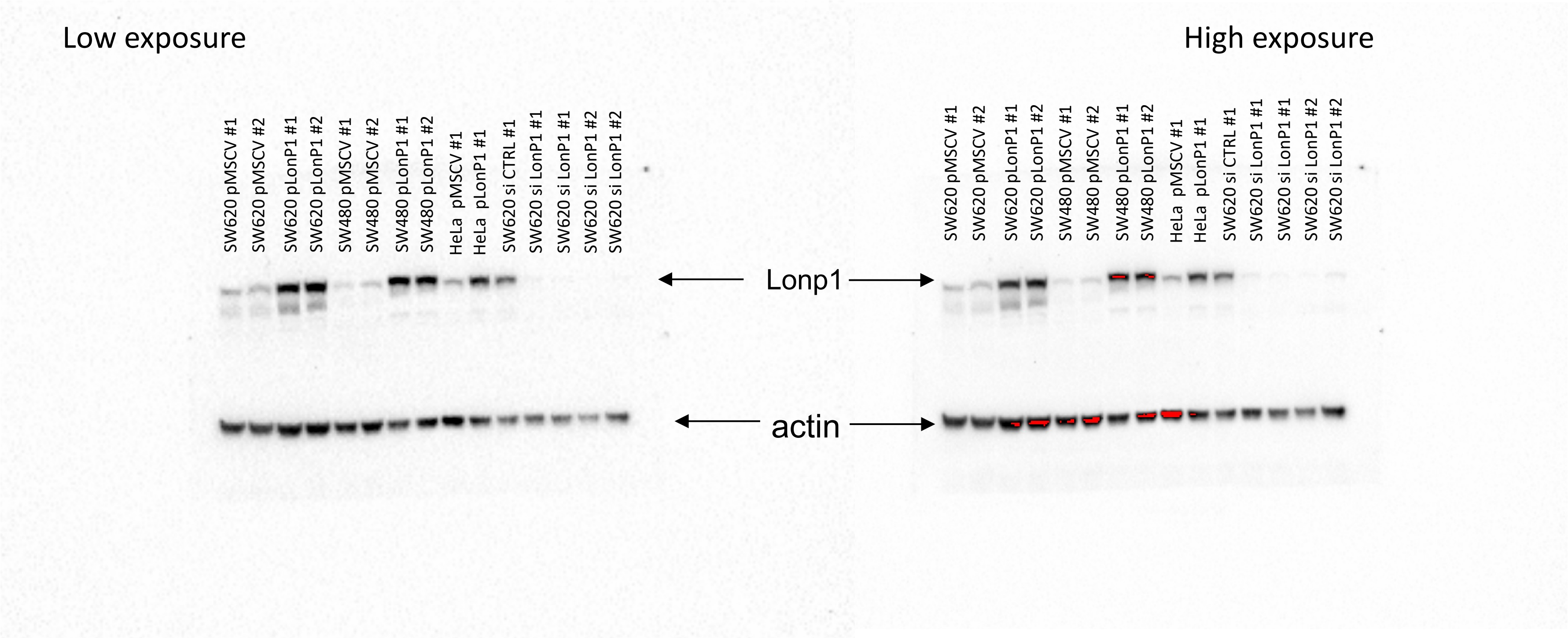

Supplementary Figure 7

Specific recognition of human Lonp1 by anti human Lonp1 antibody used in this study. Human SW620, SW480 and HeLa cells stably overexpressing Lonp1 by retroviral transduction, or control parental cells transduced with the empty vector pMSCV were probed with anti Lonp1 Ab. Cell overexpressing Lonp1 (lane 3, 4, 7, 8,10) show higher signal in WB analysis, in comparison to their control cells (lanes 1,2, 5, 6,9) show. Conversely, cells where Lonp1 was silenced by using two specific, pre validated siRNAs, show a very low signal in WB analysis, if compared to a control, scramble siRNA. The image is reported with two different exposure, to make signal in silenced cells visible. Analysis is shown in duplicate for overexpression in SW620, and SW480 cells, and for silencing with two antiLonp1 siRNA, and once for overexpression in HeLa cells.

Fig 2B

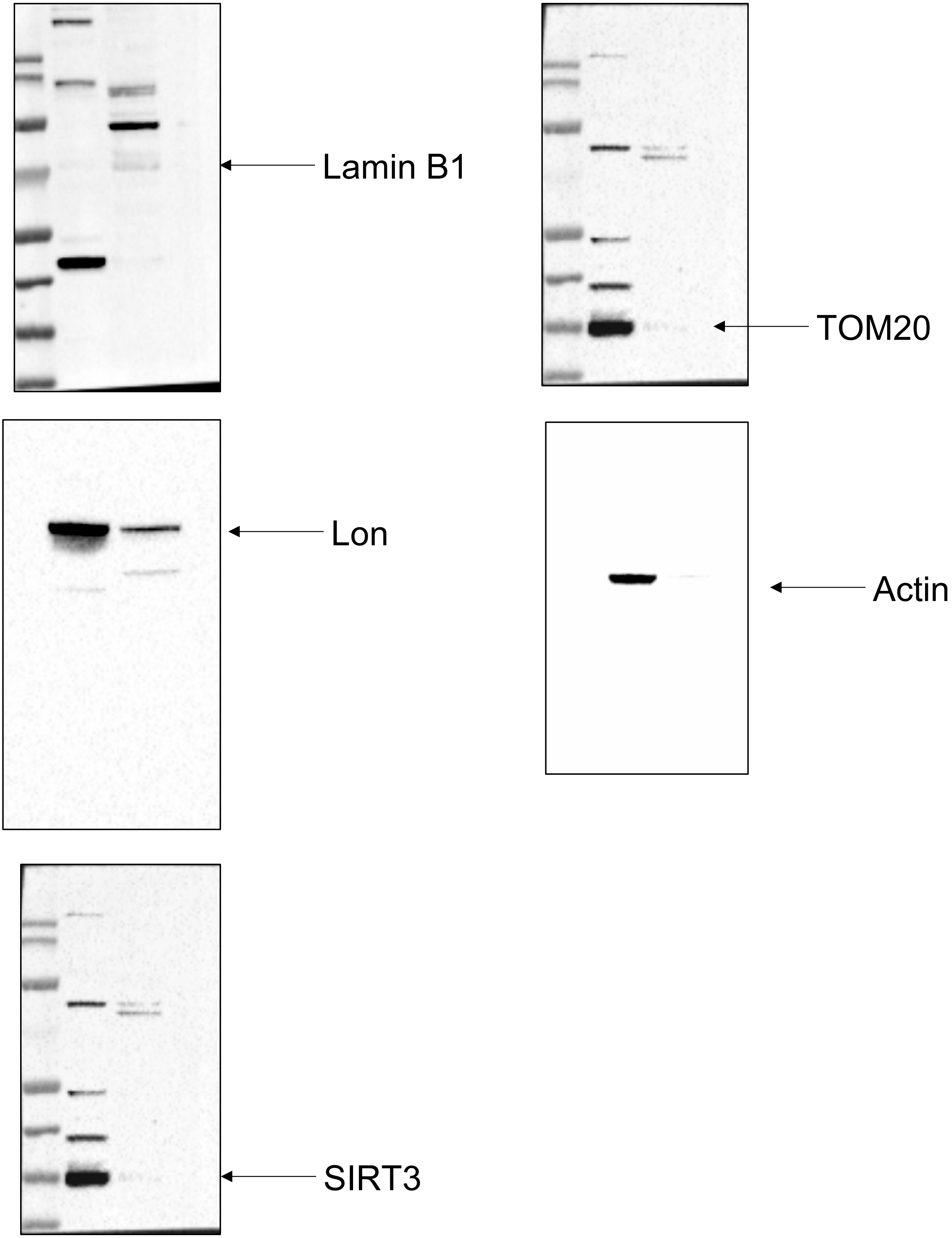

Fig 2B

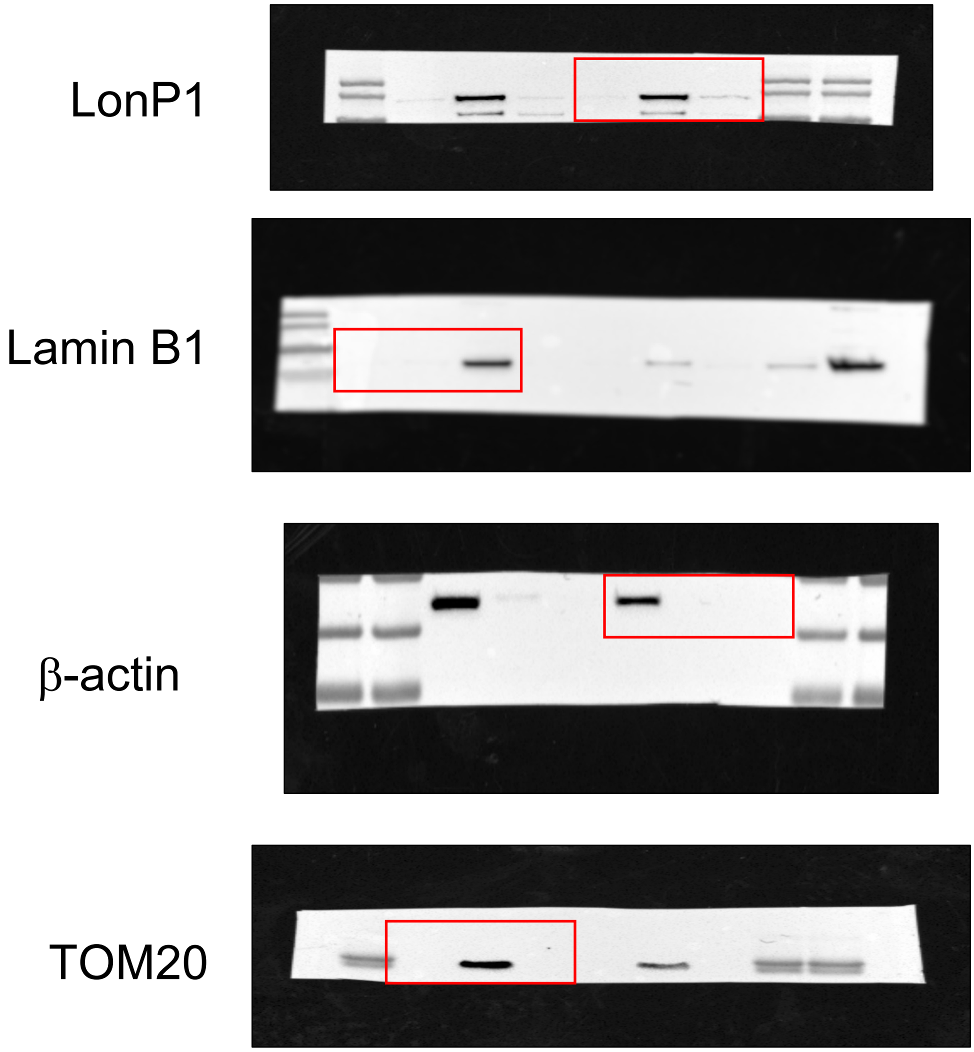

Figure 2 B different exposures of b-actin blot

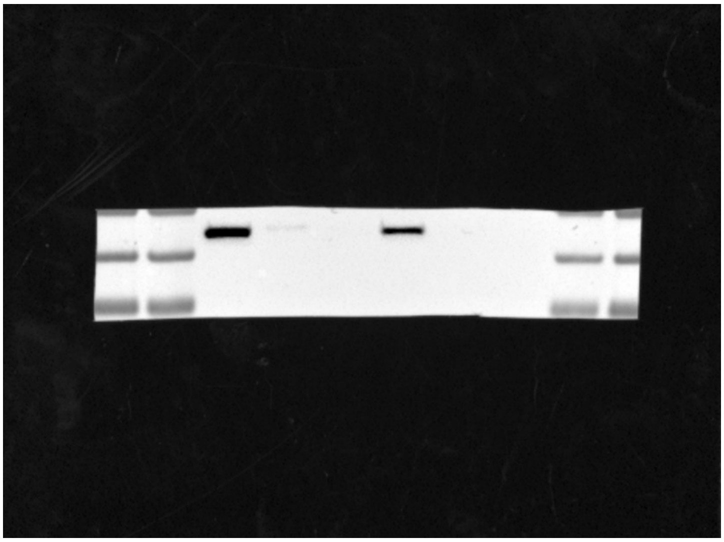

1 secs

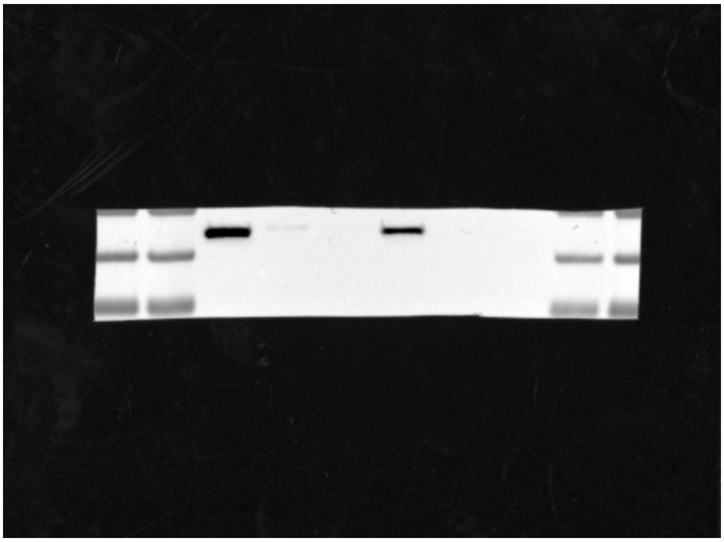

2 secs

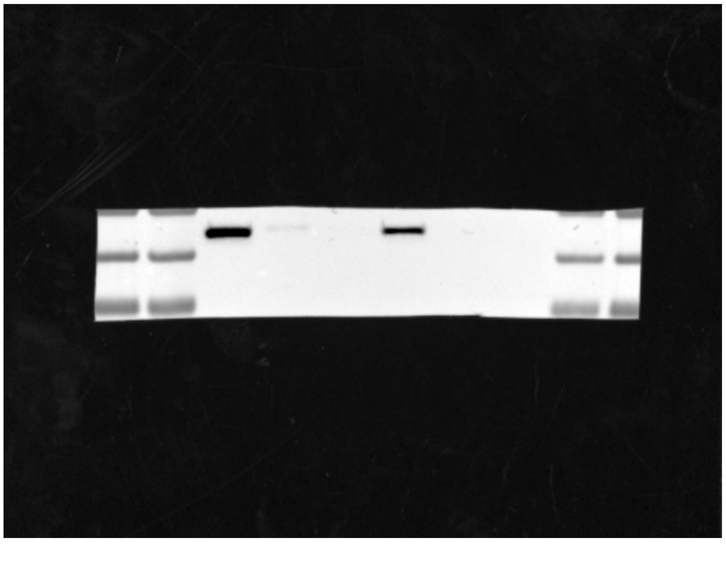

3secs

Figure 2 B different exposures of b-actin blot

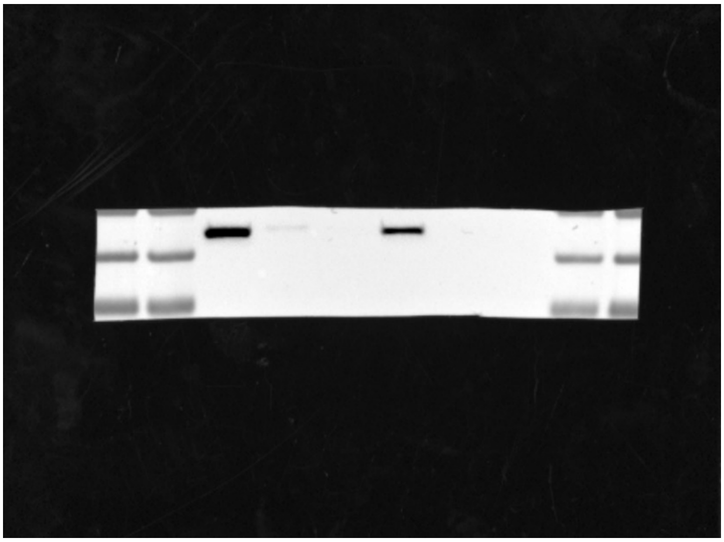

4 secs

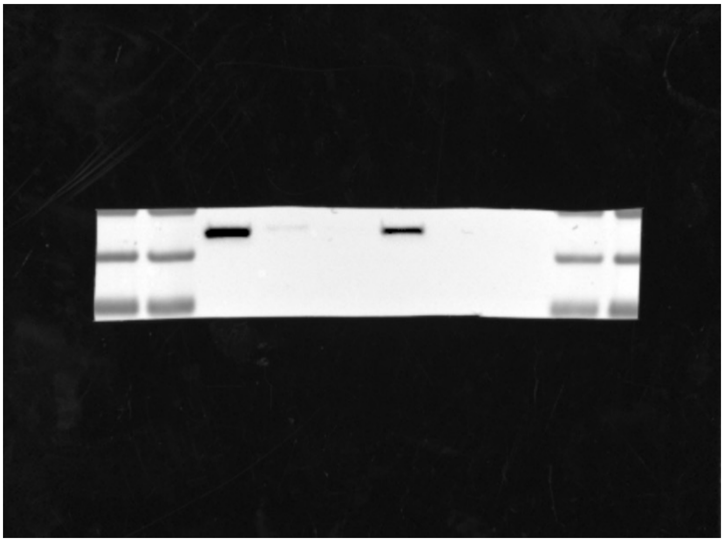

5 secs

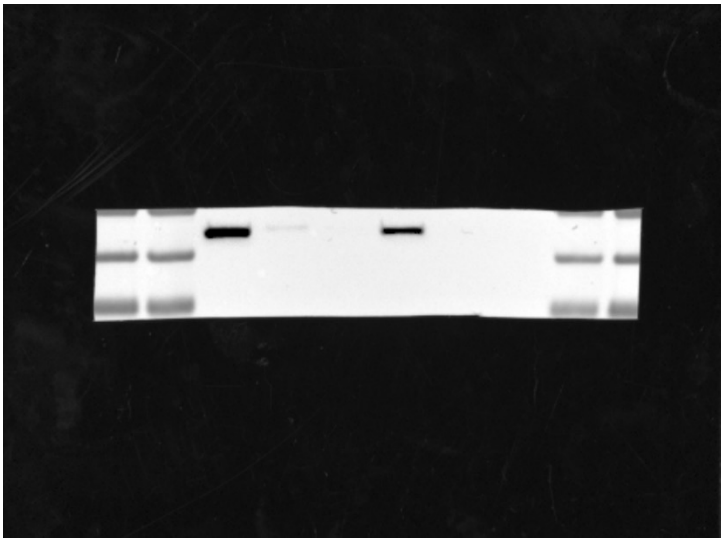

6 secs

Figure 2 B different exposures of b-actin blot

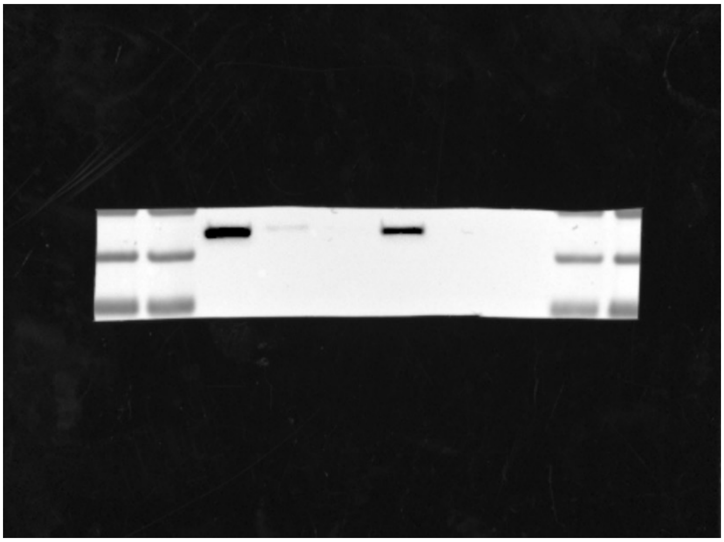

7 secs

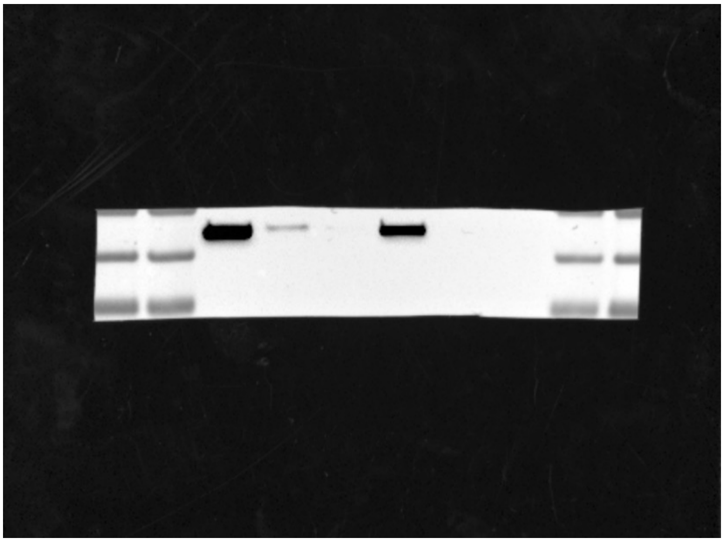

30 sec

Fig 2E

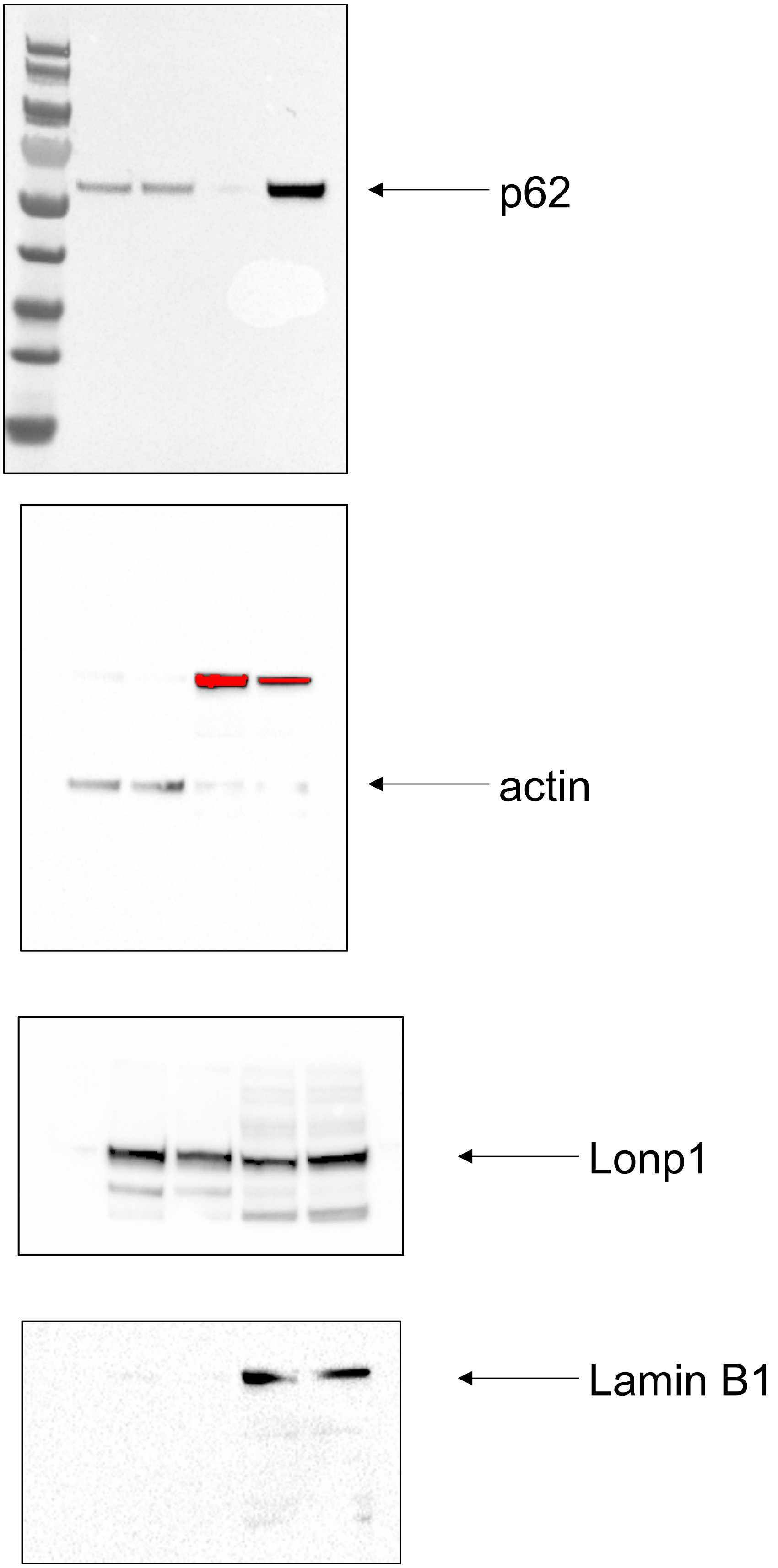

Figure 2 E actin different exposures

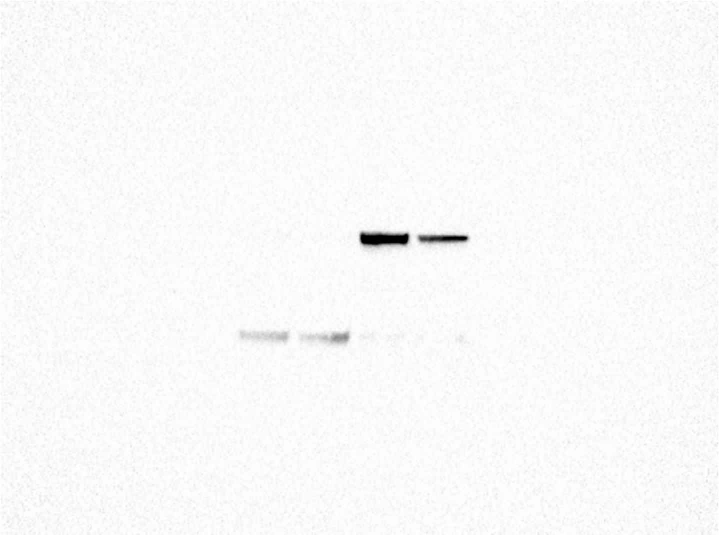

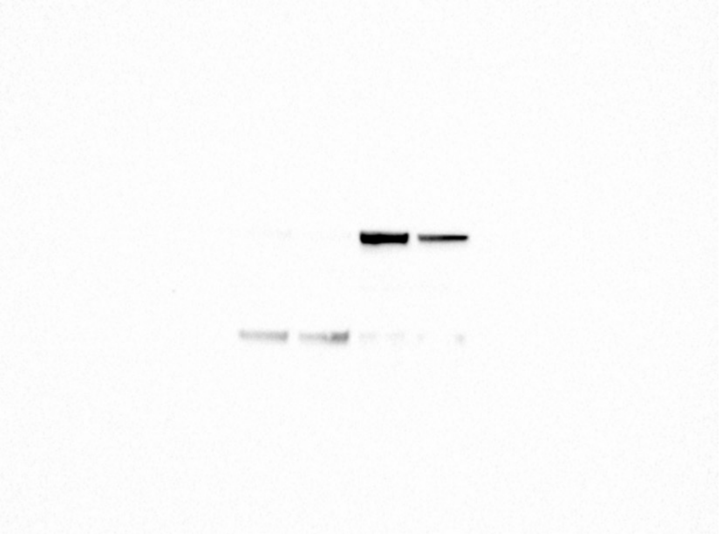

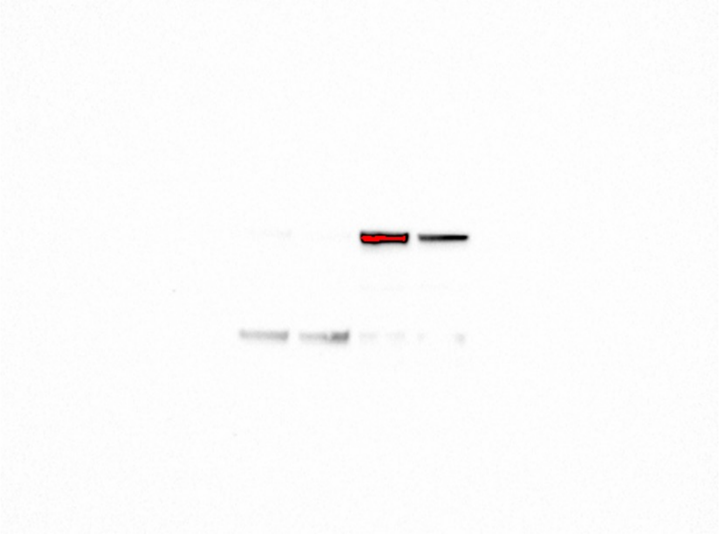

Figure 2 E actin different exposures

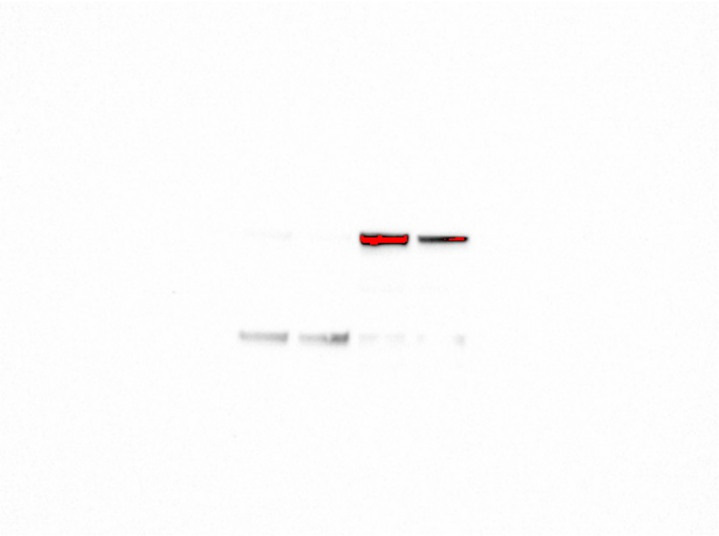

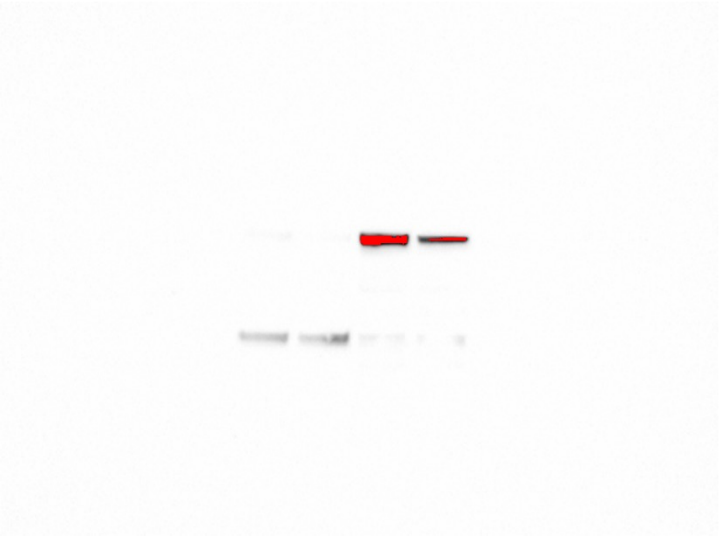

Fig 2G

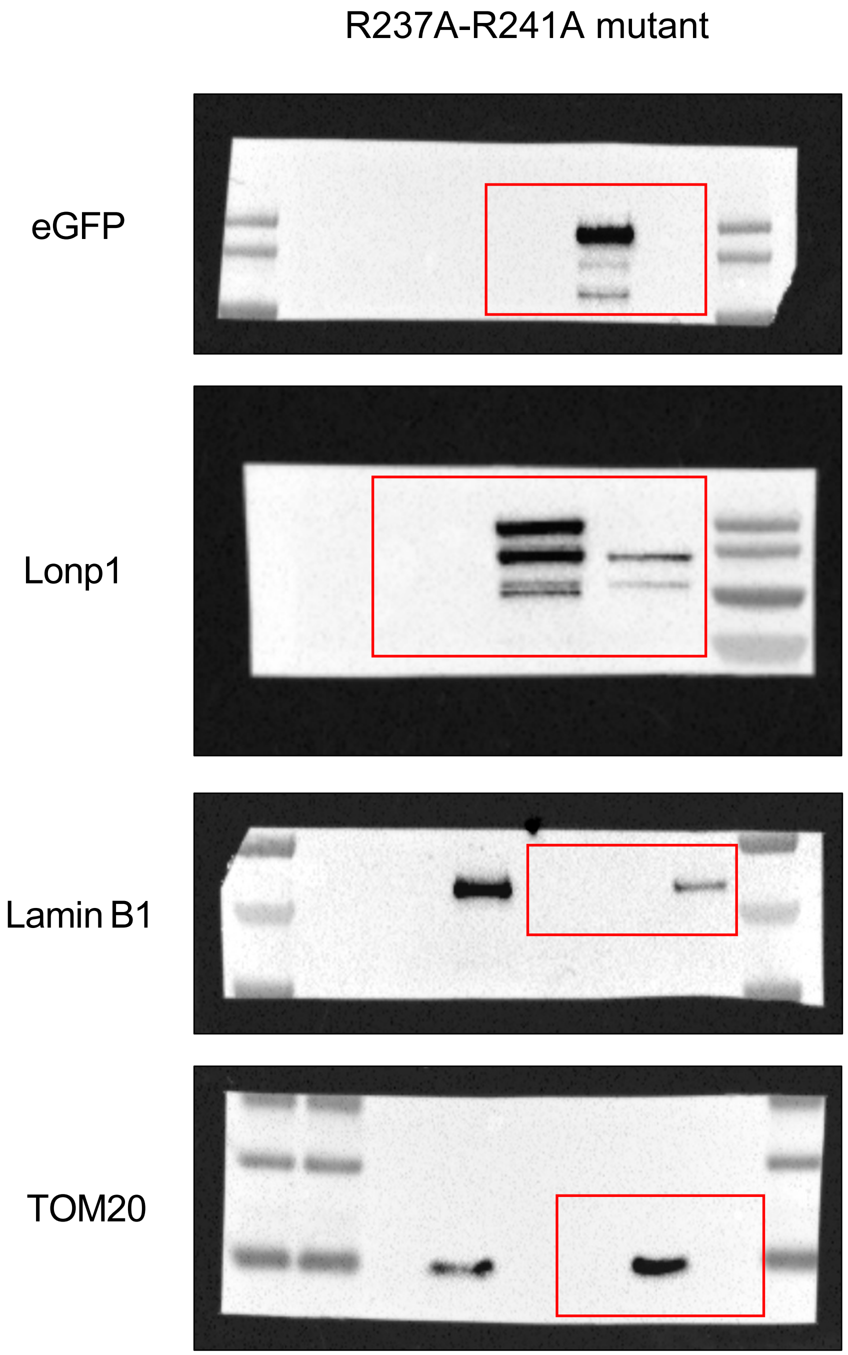

Fig. 3B

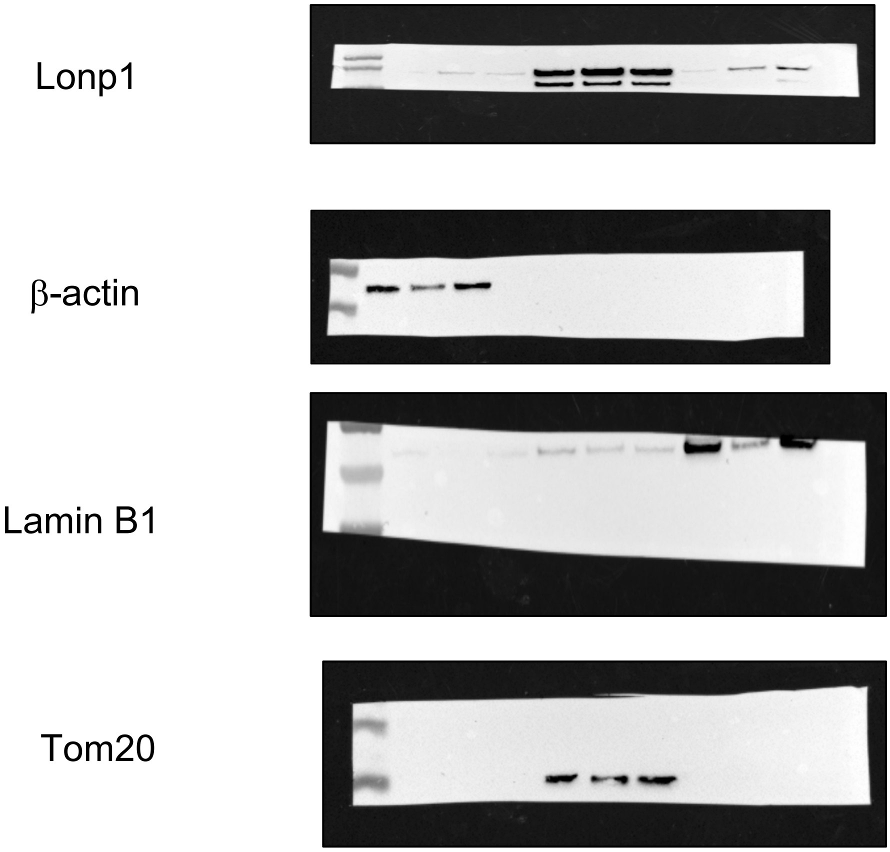

Fig 3D

IP eGFP

HSF1 IP

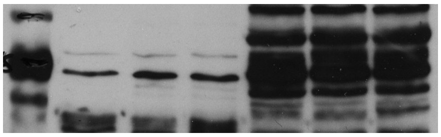

HSF1 TL

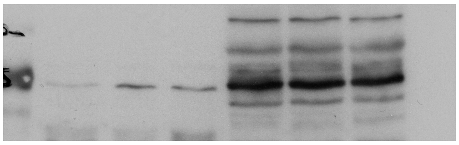

β-actin

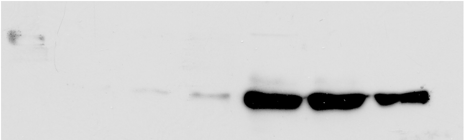

Lonp1

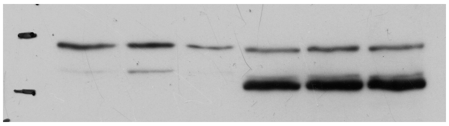

eGFP

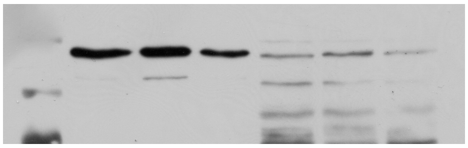

Fig 3D

IP HSF1

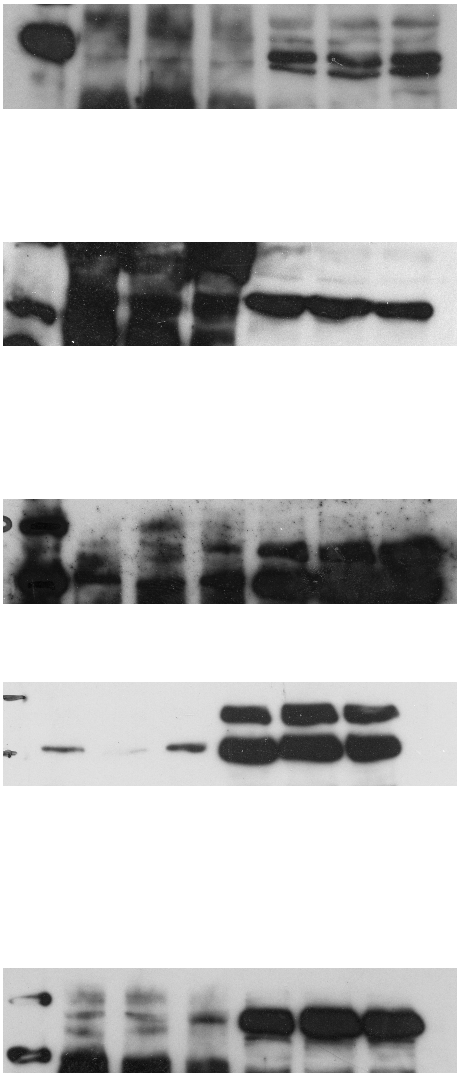

HSF1

β-actin

Lonp1 IP

Lonp1 TL

eGFP

Fig 3E

HSF1 NL

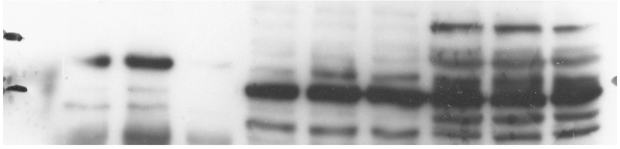

Lonp1 high exp

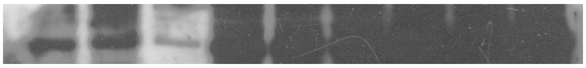

Lonp1 IP low exp

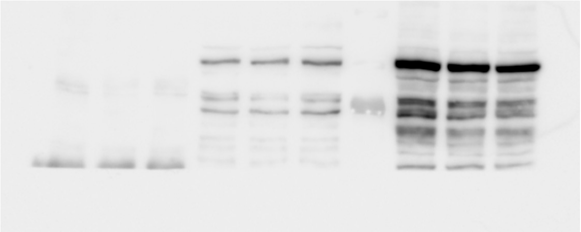

Figure 3F

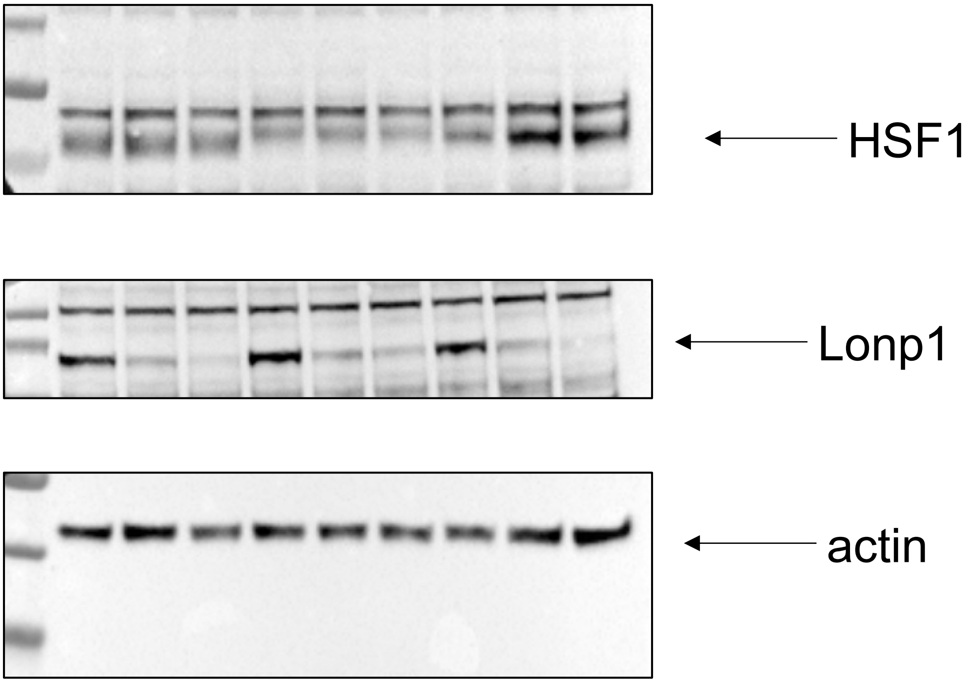

Supplementary Figure 3

Panel A   Actin – different esposures

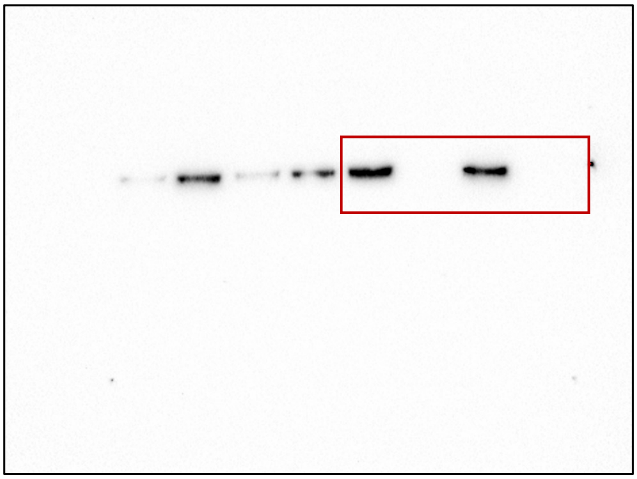

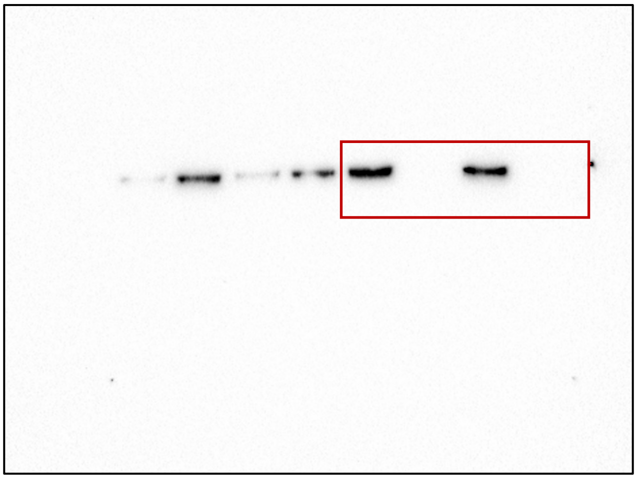

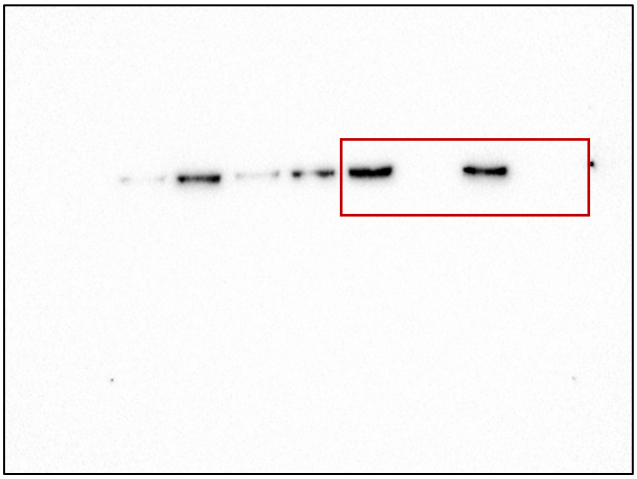

Supplementary Figure 3

Panel A   Actin – different esposures

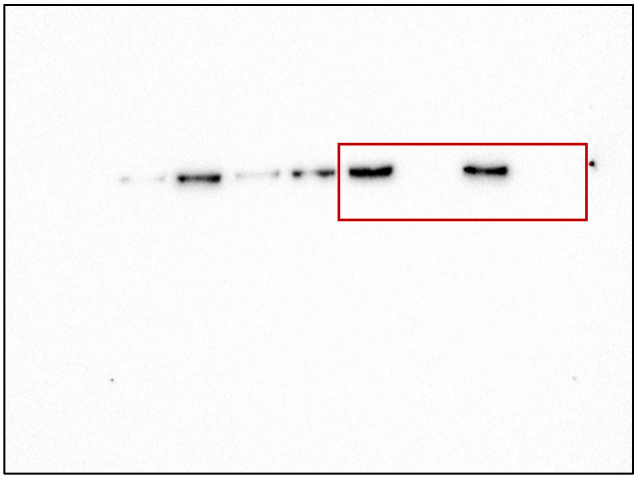

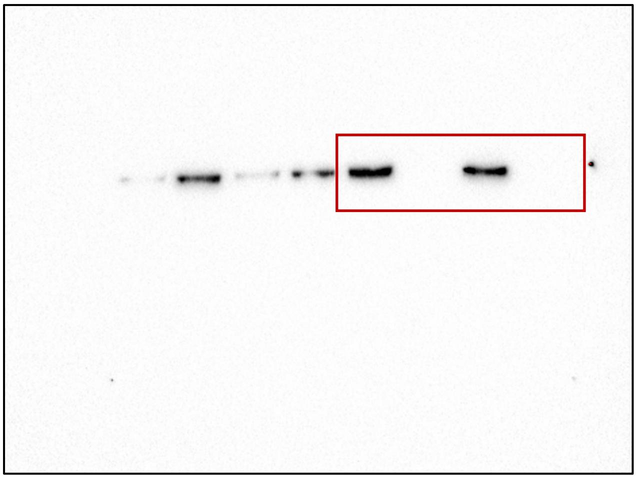

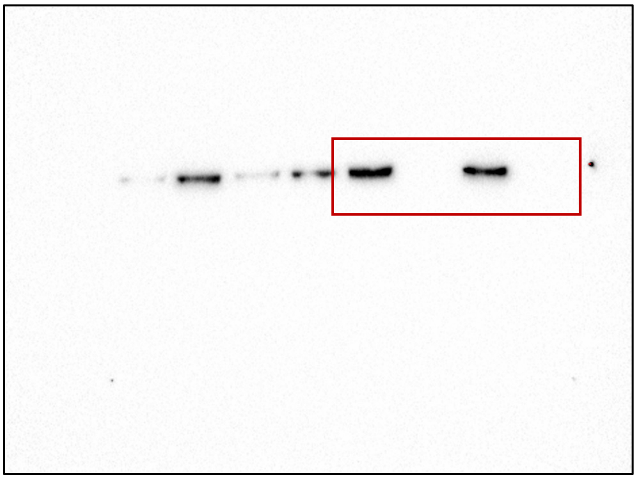

Supplementary Figure 3

Panel A   Actin – different esposures

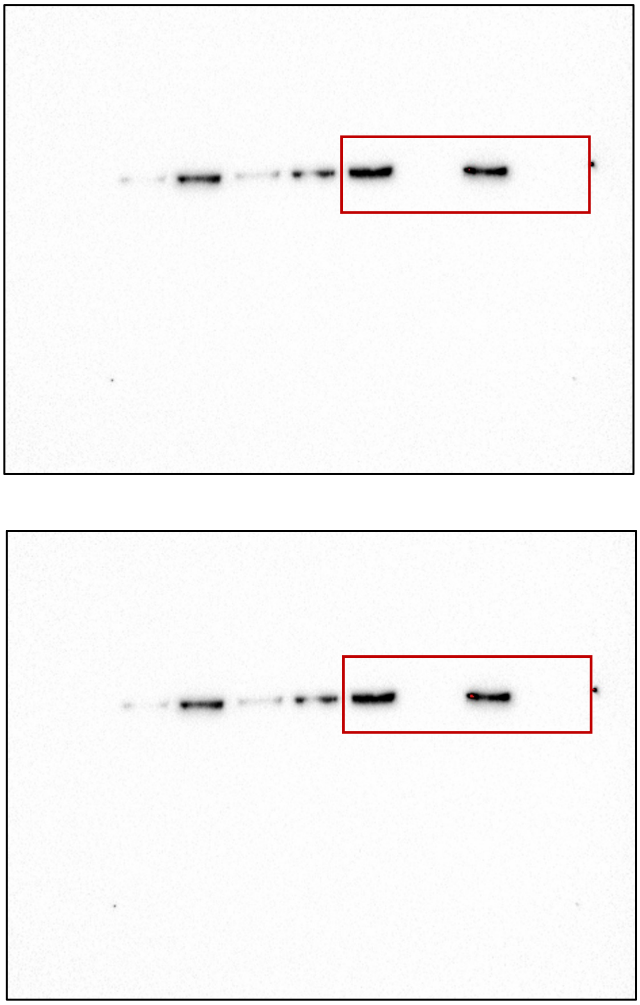

Supplementary Figure 3

Panel A Lonp1

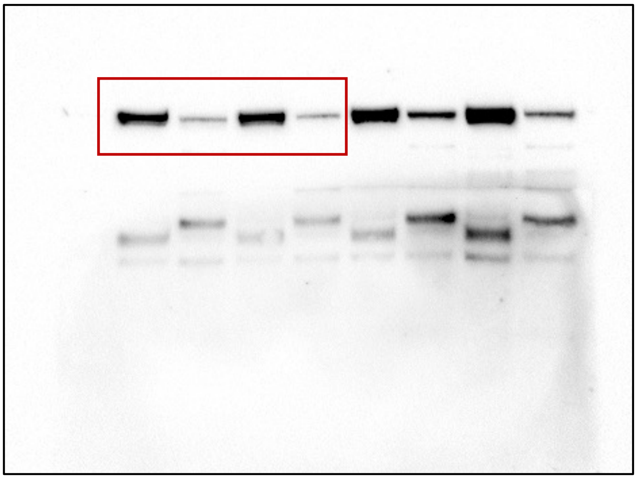

Panel A Lamin B1

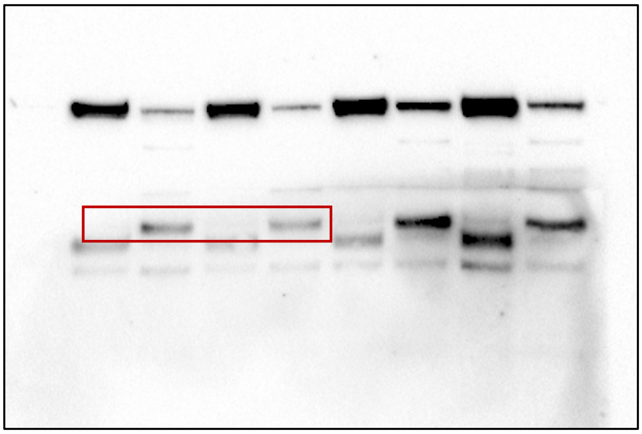

Supplementary Figure 3

Panel B Actin

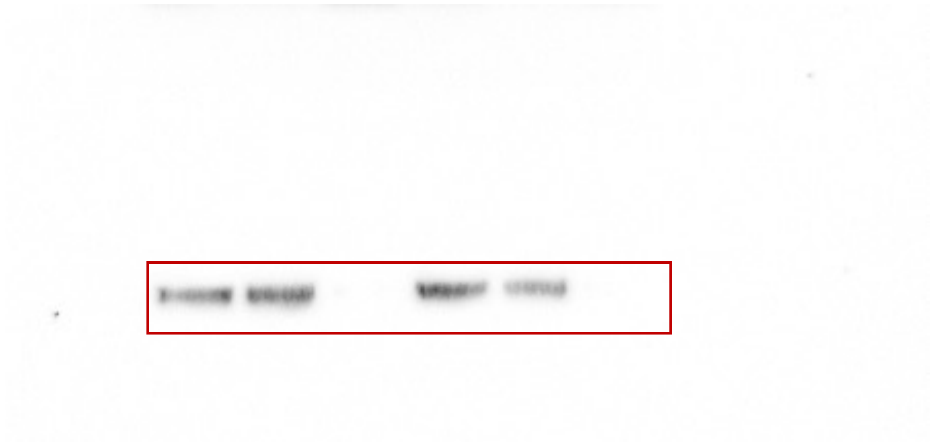

Panel B Lonp1

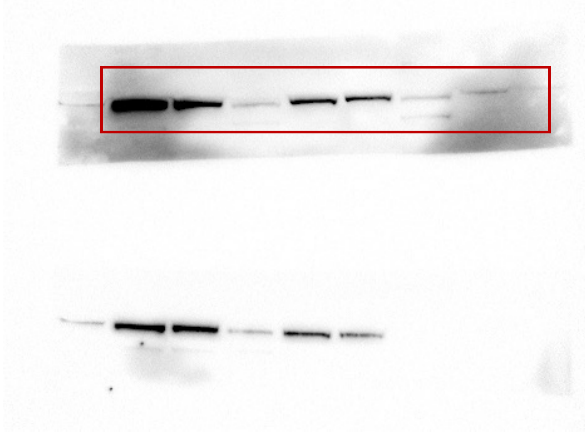

Panel B Lamin B1

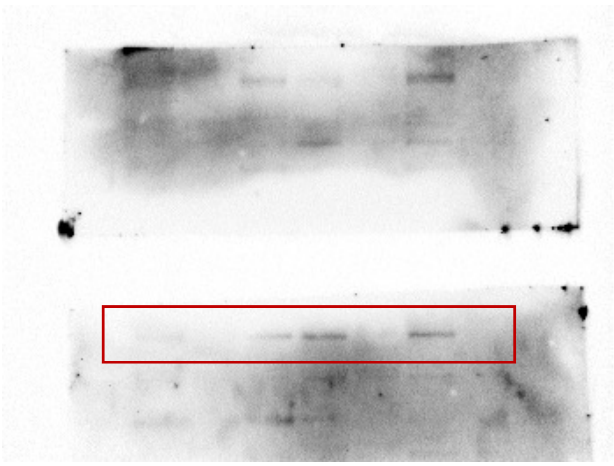

Supplementary Figure 3

Panel B right blots - Actin

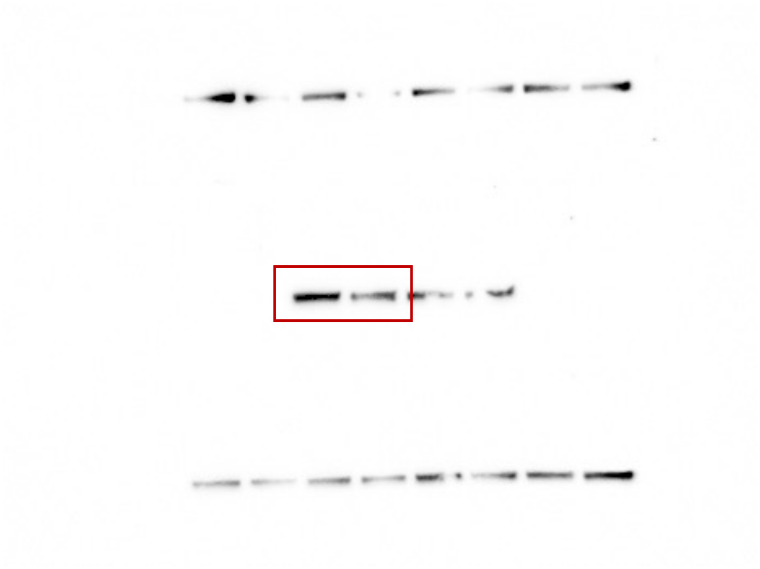

Panel B Right blots H2AX

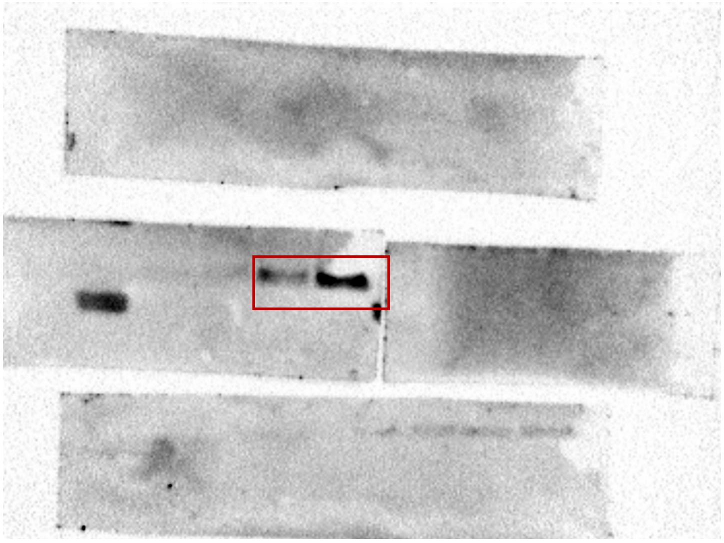

Supplementary Figure 3

Panel C Lonp1

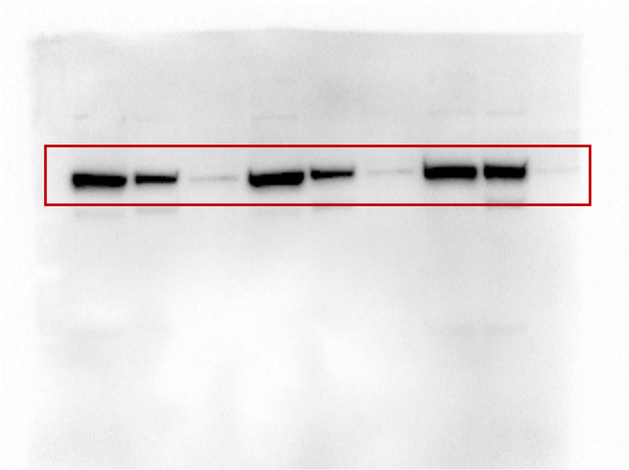

Panel C Lamin B1

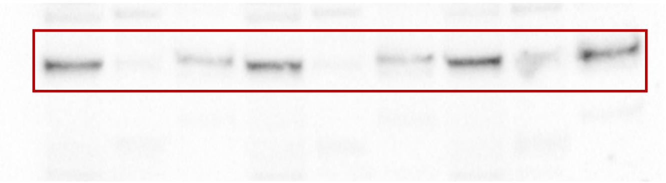

Panel C actin

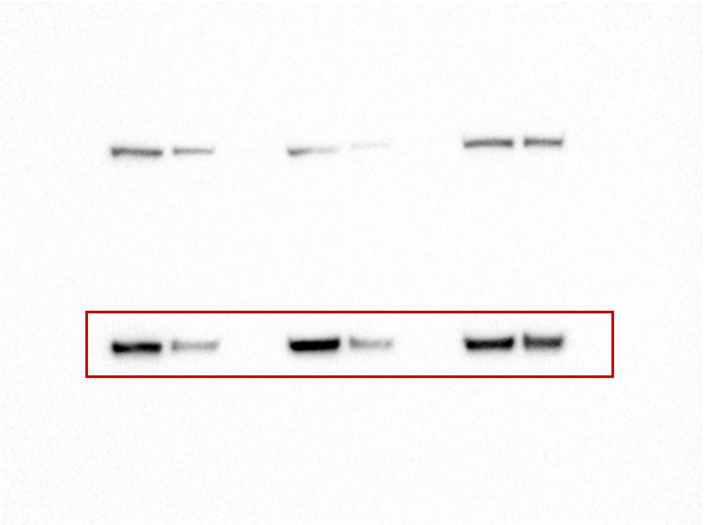

Supplementary Figure 3

Panel C lower panel H2AX

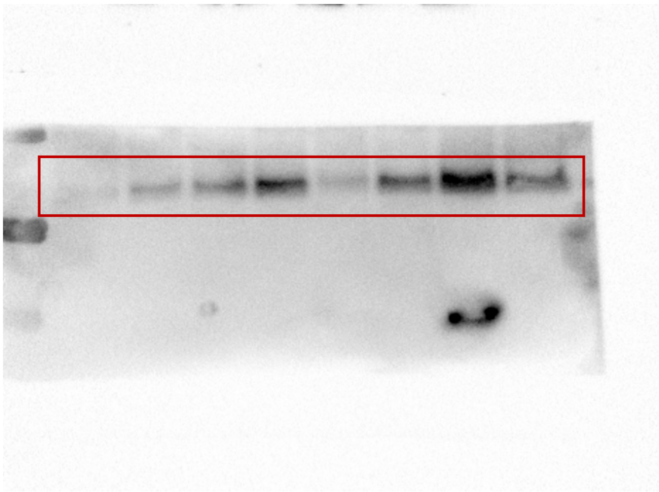

Panel C lower panel actin

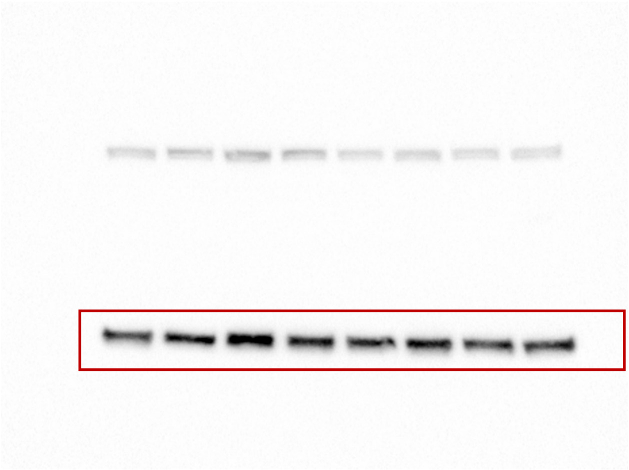

Supplementary Figure 4

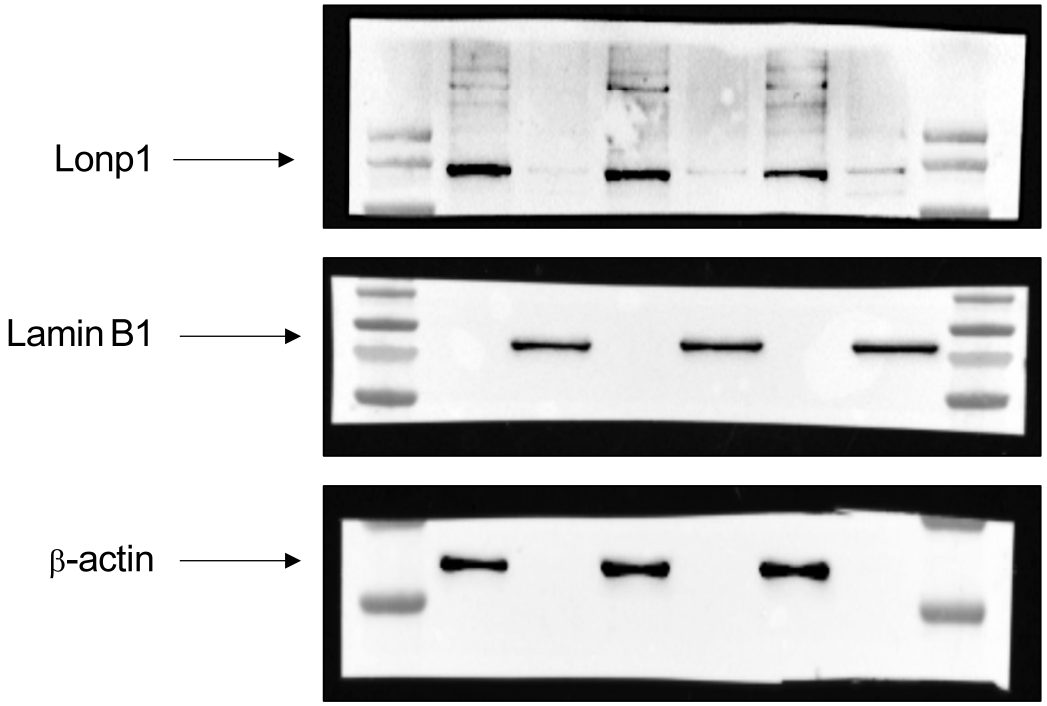

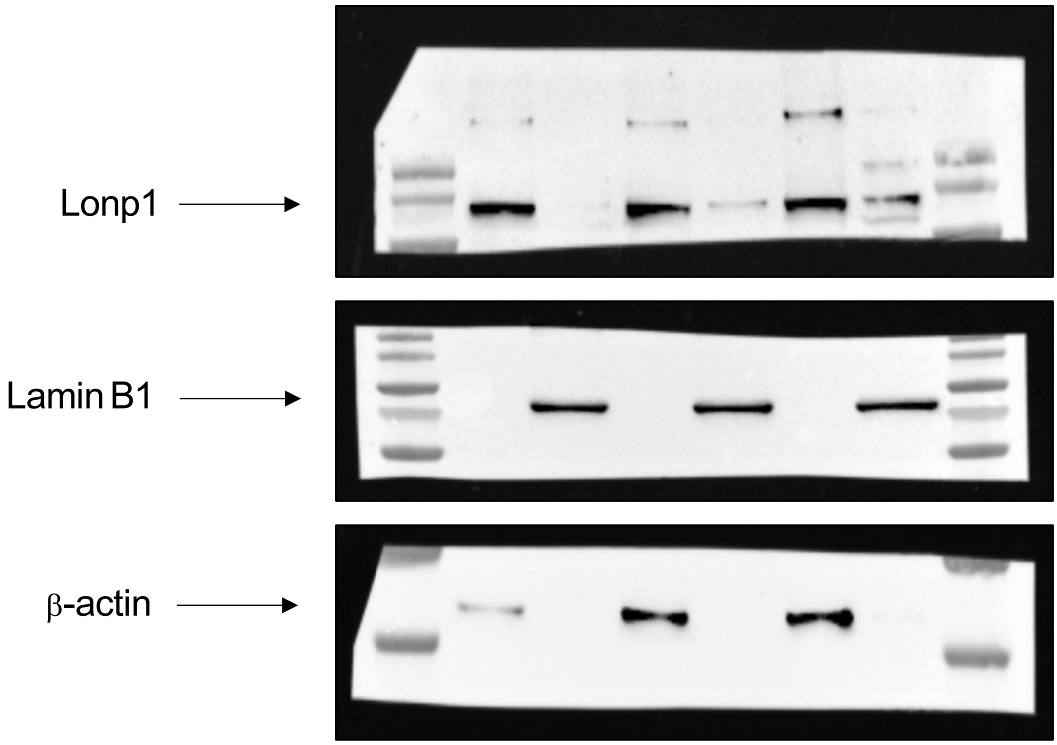

Supplementary Figure 5A

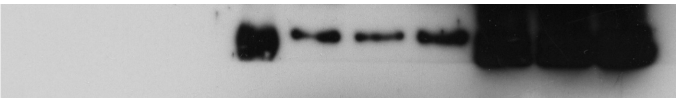

WB anti HSF-1 high exposure

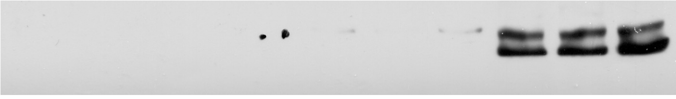

WB anti HSF-1 low exposure

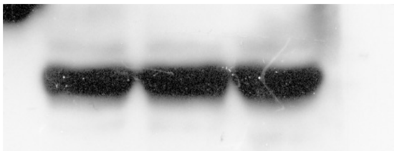

WB actin

Supplementary Figure 5B

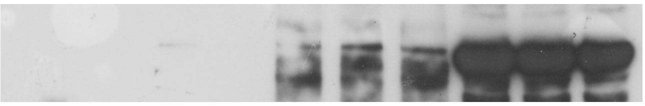

WB anti Lonp1 high exposure

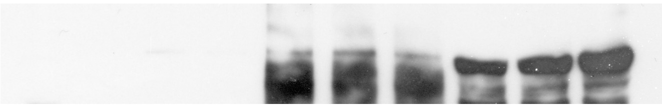

WB anti Lonp1low exposure

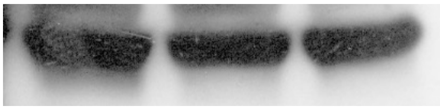

WB anti actin

Supplementary Figure 7

Low exposure

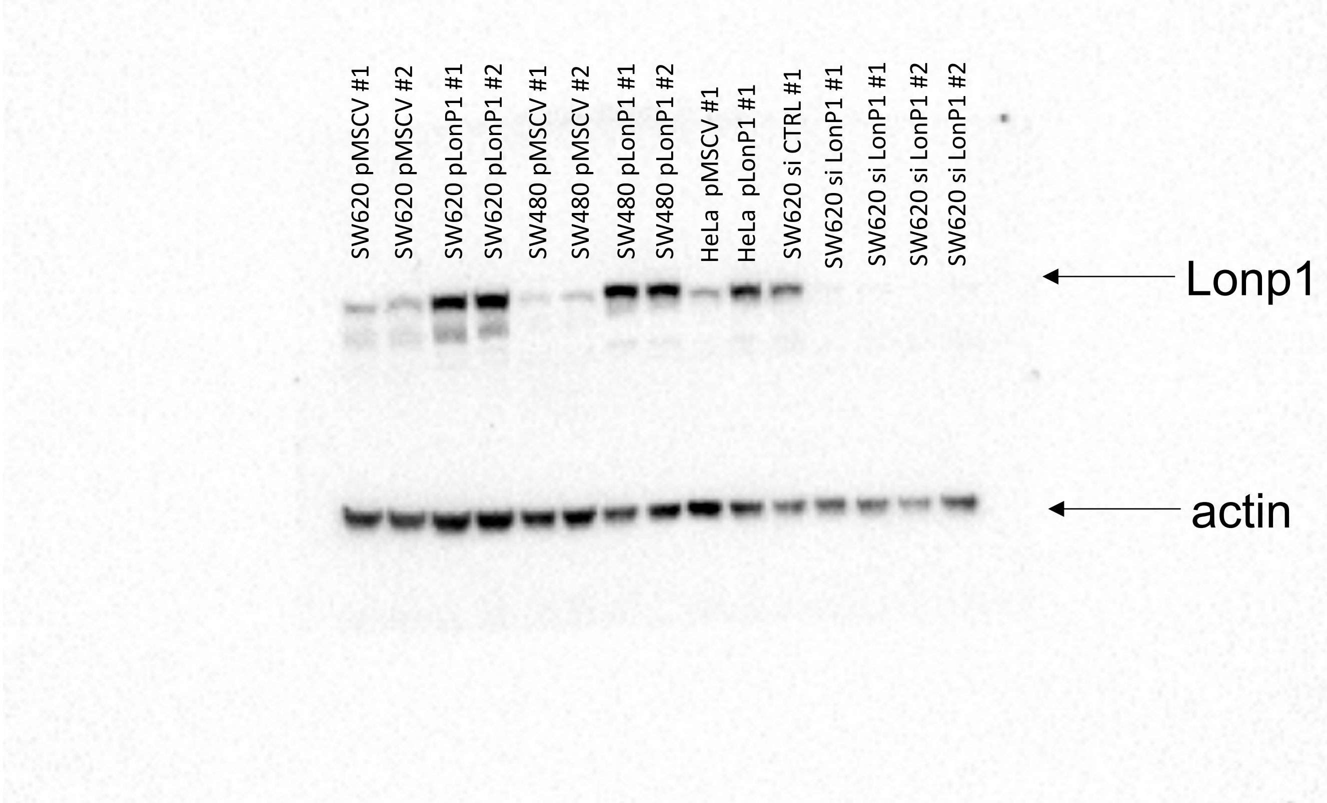

High exposure

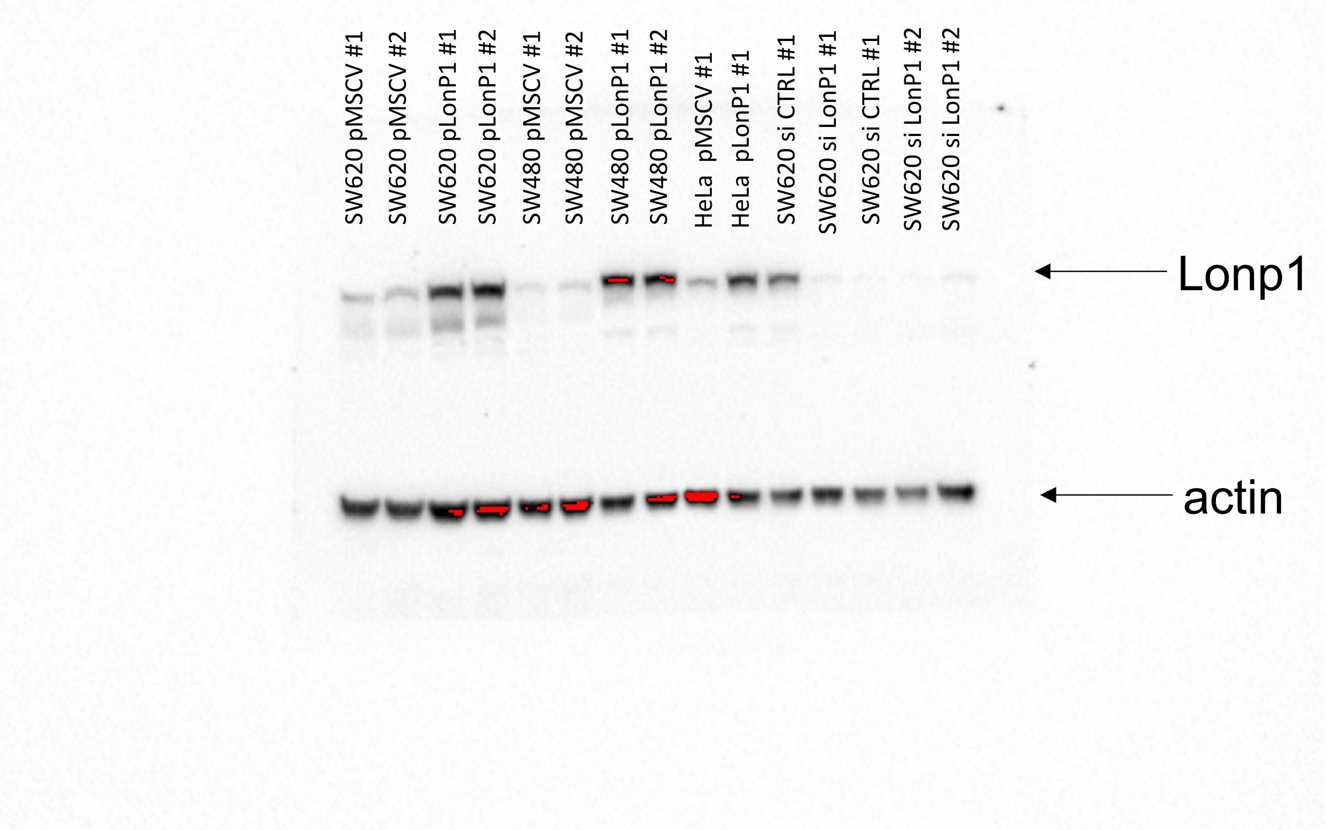

Supplement: Supplementary file 1 — Supplementary Figures. [file 41598_2022_14860_MOESM1_ESM.pdf]
