# Supplementary material for: Evidence for mitochondrial Lonp1 expression in the nucleus
Source: Sci Rep. 2022 Jun 27;12:10877. doi: 10.1038/s41598-022-14860-0 (PMC9237102; doi:10.1038/s41598-022-14860-0)
Supplement: Supplementary file 2 — Supplementary Legends. [file 41598_2022_14860_MOESM2_ESM.docx]

**Supplementary table 1.** Gene Ontology (GO) terms as determined by DAVID in cells under HS in comparison to cells kept at 37°C, either with silenced Lonp1 (siLonp1) or in cells treated with a scramble siRNA (Scramble siRNA). Significantly enriched terms (Benjamini-Hochberg adjusted p value ≤ 0.05) are in bold.

**Supplementary table 2.** Reactome pathways found to be enriched in cells under HS in comparison to cells kept at 37°C (Ctrl), either with silenced Lonp1 (siLonp1) or in cells treated with a scramble siRNA (Scramble). Significantly enriched pathways (adjusted p value ≤ 0.05) are in bold.

**Supplementary table 3.** Enriched gene sets from the Reactome collection in SW620 cells when Lonp1 is silenced, in comparison to scramble siRNA, either at 37°C (Ctrl) and at 42°C for 1 hour (HS), as detected with GSEA. Data are ranked based on Normalized enrichment score (NES).
