# Supplementary material for: Evidence for mitochondrial Lonp1 expression in the nucleus
Source: Sci Rep. 2022 Jun 27;12:10877. doi: 10.1038/s41598-022-14860-0 (PMC9237102; doi:10.1038/s41598-022-14860-0)
Supplement: Supplementary file 3 — Supplementary Table 1. [file 41598_2022_14860_MOESM3_ESM.pdf]

Supplementary Table 1

Scramble siRNA - HS vs Ctrl					
Category	Term	Count	%	P-Value	Benjamini
**GOTERM_BP_DIRECT**	**transcription. DNA-templated**	**83**	**22.3**	**8.4E-15**	**1.6E-11**
**GOTERM_BP_DIRECT**	**response to unfolded protein**	**14**	**3.8**	**1.6E-13**	**1.5E-10**
**GOTERM_BP_DIRECT**	**positive regulation of transcription from RNA polymerase II promoter**	**53**	**14.2**	**3.7E-13**	**2.4E-10**
**GOTERM_BP_DIRECT**	**regulation of transcription. DNA-templated**	**62**	**16.7**	**2.3E-10**	**9.7E-8**
**GOTERM_BP_DIRECT**	**negative regulation of transcription from RNA polymerase II promoter**	**40**	**10.8**	**2.5E-10**	**9.7E-8**
**GOTERM_BP_DIRECT**	**protein refolding**	**7**	**1.9**	**1.1E-7**	**3.6E-5**
**GOTERM_BP_DIRECT**	**regulation of cellular response to heat**	**11**	**3.0**	**6.4E-7**	**1.8E-4**
**GOTERM_BP_DIRECT**	**transcription from RNA polymerase II promoter**	**27**	**7.3**	**1.1E-6**	**2.6E-4**
**GOTERM_BP_DIRECT**	**skeletal muscle cell differentiation**	**9**	**2.4**	**1.8E-6**	**3.9E-4**
**GOTERM_BP_DIRECT**	**negative regulation of apoptotic process**	**21**	**5.6**	**1.4E-4**	**2.7E-2**
**GOTERM_BP_DIRECT**	**negative regulation of transcription. DNA-templated**	**22**	**5.9**	**1.7E-4**	**3.1E-2**
GOTERM_BP_DIRECT	reactive oxygen species metabolic process	6	1.6	3.2E-4	5.2E-2
GOTERM_BP_DIRECT	negative regulation of inclusion body assembly	4	1.1	5.6E-4	8.3E-2
GOTERM_BP_DIRECT	cellular response to vascular endothelial growth factor stimulus	5	1.3	6.0E-4	8.3E-2
GOTERM_BP_DIRECT	regulation of transcription from RNA polymerase II promoter	19	5.1	7.1E-4	8.6E-2
GOTERM_BP_DIRECT	regulation of cell death	4	1.1	7.7E-4	8.6E-2
GOTERM_BP_DIRECT	positive regulation of apoptotic process	15	4.0	7.8E-4	8.6E-2
GOTERM_BP_DIRECT	response to hypoxia	11	3.0	8.4E-4	8.6E-2
GOTERM_BP_DIRECT	thymus development	6	1.6	8.5E-4	8.6E-2
GOTERM_BP_DIRECT	endoderm formation	4	1.1	1.0E-3	9.6E-2
GOTERM_BP_DIRECT	cellular response to hormone stimulus	6	1.6	1.0E-3	9.6E-2
GOTERM_BP_DIRECT	protein folding	11	3.0	1.2E-3	1.0E-1
GOTERM_BP_DIRECT	cellular response to amino acid stimulus	6	1.6	1.3E-3	1.1E-1
GOTERM_BP_DIRECT	response to heat	6	1.6	1.4E-3	1.1E-1
GOTERM_BP_DIRECT	cellular heat acclimation	3	0.8	1.8E-3	1.4E-1
GOTERM_BP_DIRECT	response to hydrogen peroxide	6	1.6	1.8E-3	1.4E-1
GOTERM_BP_DIRECT	intrinsic apoptotic signaling pathway in response to endoplasmic reticulum stress	5	1.3	2.4E-3	1.7E-1
GOTERM_BP_DIRECT	odontogenesis of dentin-containing tooth	6	1.6	2.6E-3	1.8E-1
GOTERM_BP_DIRECT	cellular response to extracellular stimulus	4	1.1	3.5E-3	2.2E-1
GOTERM_BP_DIRECT	response to mechanical stimulus	6	1.6	3.5E-3	2.2E-1
GOTERM_BP_DIRECT	cellular response to heat	5	1.3	3.7E-3	2.2E-1
GOTERM_BP_DIRECT	positive regulation of cell differentiation	5	1.3	3.7E-3	2.2E-1

GOTERM_BP_DIRECT	negative regulation of sequence-specific DNA binding transcription factor activity	6	1.6	3.8E-3	2.2E-1
GOTERM_BP_DIRECT	positive regulation of transcription. DNA-templated	19	5.1	3.9E-3	2.2E-1
GOTERM_BP_DIRECT	positive regulation of osteoclast differentiation	4	1.1	4.1E-3	2.2E-1
GOTERM_BP_DIRECT	cellular response to corticotropin-releasing hormone stimulus	3	0.8	4.3E-3	2.3E-1
GOTERM_BP_DIRECT	cell-cell signaling	12	3.2	4.9E-3	2.5E-1
GOTERM_BP_DIRECT	response to estrogen	6	1.6	5.3E-3	2.7E-1
GOTERM_BP_DIRECT	angiogenesis	11	3.0	5.6E-3	2.8E-1
GOTERM_BP_DIRECT	response to insulin	6	1.6	6.1E-3	2.9E-1
GOTERM_BP_DIRECT	negative regulation of myoblast differentiation	4	1.1	7.1E-3	3.3E-1
GOTERM_BP_DIRECT	cellular response to interleukin-1	6	1.6	7.7E-3	3.4E-1
GOTERM_BP_DIRECT	response to lipopolysaccharide	9	2.4	7.9E-3	3.4E-1
GOTERM_BP_DIRECT	peripheral nervous system development	4	1.1	8.0E-3	3.4E-1
GOTERM_BP_DIRECT	response to cAMP	5	1.3	8.1E-3	3.4E-1
GOTERM_BP_DIRECT	negative regulation of neuron apoptotic process	8	2.2	8.2E-3	3.4E-1
GOTERM_BP_DIRECT	fat cell differentiation	6	1.6	8.7E-3	3.6E-1
GOTERM_BP_DIRECT	inactivation of MAPK activity	4	1.1	8.9E-3	3.6E-1
GOTERM_BP_DIRECT	regulation of mRNA stability	7	1.9	9.1E-3	3.6E-1
GOTERM_BP_DIRECT	protein stabilization	8	2.2	9.6E-3	3.6E-1
GOTERM_BP_DIRECT	multicellular organism development	18	4.8	9.7E-3	3.6E-1
GOTERM_BP_DIRECT	negative regulation of gene expression	8	2.2	9.9E-3	3.6E-1
GOTERM_BP_DIRECT	positive regulation of cardiac muscle contraction	3	0.8	9.9E-3	3.6E-1
GOTERM_BP_DIRECT	positive regulation of interleukin-8 production	4	1.1	1.0E-2	3.6E-1
GOTERM_BP_DIRECT	cellular response to DNA damage stimulus	10	2.7	1.0E-2	3.7E-1
GOTERM_BP_DIRECT	T cell differentiation in thymus	4	1.1	1.1E-2	3.8E-1
GOTERM_BP_DIRECT	cellular response to calcium ion	5	1.3	1.2E-2	3.9E-1
GOTERM_BP_DIRECT	embryonic hindlimb morphogenesis	4	1.1	1.2E-2	3.9E-1
GOTERM_BP_DIRECT	patterning of blood vessels	4	1.1	1.2E-2	3.9E-1
GOTERM_BP_DIRECT	cellular response to tumor necrosis factor	7	1.9	1.2E-2	3.9E-1
GOTERM_BP_DIRECT	chaperone mediated protein folding requiring cofactor	3	0.8	1.2E-2	3.9E-1
GOTERM_BP_DIRECT	anterior/posterior pattern specification	6	1.6	1.3E-2	3.9E-1
GOTERM_BP_DIRECT	cellular response to glucose starvation	4	1.1	1.3E-2	3.9E-1
GOTERM_BP_DIRECT	regulation of RNA splicing	4	1.1	1.3E-2	3.9E-1
GOTERM_BP_DIRECT	negative regulation of Notch signaling pathway	4	1.1	1.3E-2	3.9E-1
GOTERM_BP_DIRECT	ventricular septum morphogenesis	4	1.1	1.3E-2	3.9E-1
GOTERM_BP_DIRECT	cellular response to fibroblast growth factor stimulus	4	1.1	1.5E-2	4.0E-1
GOTERM_BP_DIRECT	protein localization to nucleus	4	1.1	1.5E-2	4.0E-1
GOTERM_BP_DIRECT	regulation of ossification	3	0.8	1.5E-2	4.0E-1

GOTERM_BP_DIRECT	positive regulation of nuclear-transcribed mRNA poly(A) tail shortening	3	0.8	1.5E-2	4.0E-1
GOTERM_BP_DIRECT	positive regulation of angiogenesis	7	1.9	1.5E-2	4.0E-1
GOTERM_BP_DIRECT	negative regulation of cell death	5	1.3	1.5E-2	4.0E-1
GOTERM_BP_DIRECT	determination of left/right symmetry	5	1.3	1.5E-2	4.0E-1
GOTERM_BP_DIRECT	intrinsic apoptotic signaling pathway in response to DNA damage by p53 class mediator	4	1.1	1.6E-2	4.2E-1
GOTERM_BP_DIRECT	positive regulation of transcription from RNA polymerase II promoter in response to endoplasmic reticulum stress	3	0.8	1.8E-2	4.4E-1
GOTERM_BP_DIRECT	3'-UTR-mediated mRNA destabilization	3	0.8	1.8E-2	4.4E-1
GOTERM_BP_DIRECT	positive regulation of DNA damage response. signal transduction by p53 class mediator	3	0.8	1.8E-2	4.4E-1
GOTERM_BP_DIRECT	muscle organ development	6	1.6	1.9E-2	4.8E-1
GOTERM_BP_DIRECT	mitotic cell cycle arrest	3	0.8	2.1E-2	4.9E-1
GOTERM_BP_DIRECT	trophectodermal cell differentiation	3	0.8	2.1E-2	4.9E-1
GOTERM_BP_DIRECT	left/right axis specification	3	0.8	2.1E-2	4.9E-1
GOTERM_BP_DIRECT	response to stress	5	1.3	2.1E-2	5.0E-1
GOTERM_BP_DIRECT	transforming growth factor beta receptor signaling pathway	6	1.6	2.2E-2	5.1E-1
GOTERM_BP_DIRECT	DNA damage response. signal transduction by p53 class mediator resulting in cell cycle arrest	5	1.3	2.2E-2	5.2E-1
GOTERM_BP_DIRECT	male gonad development	6	1.6	2.4E-2	5.4E-1
GOTERM_BP_DIRECT	spleen development	4	1.1	2.4E-2	5.4E-1
GOTERM_BP_DIRECT	neuron differentiation	6	1.6	2.5E-2	5.4E-1
GOTERM_BP_DIRECT	cellular response to oxidative stress	5	1.3	2.5E-2	5.4E-1
GOTERM_BP_DIRECT	positive regulation of proteasomal ubiquitin-dependent protein catabolic process	5	1.3	2.5E-2	5.4E-1
GOTERM_BP_DIRECT	cellular response to hypoxia	6	1.6	2.6E-2	5.5E-1
GOTERM_BP_DIRECT	negative regulation of T cell proliferation	4	1.1	2.6E-2	5.5E-1
GOTERM_BP_DIRECT	erythrocyte development	3	0.8	2.7E-2	5.7E-1
GOTERM_BP_DIRECT	positive regulation of NF-kappaB transcription factor activity	7	1.9	2.8E-2	5.9E-1
GOTERM_BP_DIRECT	cell death	4	1.1	3.0E-2	6.0E-1
GOTERM_BP_DIRECT	regulation of cyclin-dependent protein serine/threonine kinase activity	4	1.1	3.0E-2	6.0E-1
GOTERM_BP_DIRECT	response to glucose	5	1.3	3.0E-2	6.0E-1
GOTERM_BP_DIRECT	positive regulation of p38MAPK cascade	3	0.8	3.1E-2	6.0E-1
GOTERM_BP_DIRECT	regulation of protein ubiquitination	3	0.8	3.1E-2	6.0E-1
GOTERM_BP_DIRECT	positive regulation of endothelial cell proliferation	5	1.3	3.2E-2	6.1E-1
GOTERM_BP_DIRECT	positive regulation of cysteine-type endopeptidase activity involved in apoptotic process	4	1.1	3.2E-2	6.1E-1
GOTERM_BP_DIRECT	regulation of apoptotic process	9	2.4	3.2E-2	6.1E-1

GOTERM_BP_DIRECT	negative regulation of protein ubiquitination	4	1.1	3.4E-2	6.1E-1
GOTERM_BP_DIRECT	regulation of chloride transport	2	0.5	3.4E-2	6.1E-1
GOTERM_BP_DIRECT	positive regulation of mRNA endonucleolytic cleavage involved in unfolded protein response	2	0.5	3.4E-2	6.1E-1
GOTERM_BP_DIRECT	positive regulation of endoribonuclease activity	2	0.5	3.4E-2	6.1E-1
GOTERM_BP_DIRECT	negative regulation of mast cell cytokine production	2	0.5	3.4E-2	6.1E-1
GOTERM_BP_DIRECT	response to dsRNA	2	0.5	3.4E-2	6.1E-1
GOTERM_BP_DIRECT	negative regulation of establishment of protein localization to mitochondrion	2	0.5	3.4E-2	6.1E-1
GOTERM_BP_DIRECT	cellular response to mechanical stimulus	5	1.3	3.5E-2	6.2E-1
GOTERM_BP_DIRECT	cell cycle arrest	7	1.9	3.6E-2	6.4E-1
GOTERM_BP_DIRECT	pharyngeal system development	3	0.8	3.8E-2	6.5E-1
GOTERM_BP_DIRECT	cell fate determination	3	0.8	3.8E-2	6.5E-1
GOTERM_BP_DIRECT	response to corticosterone	3	0.8	3.8E-2	6.5E-1
GOTERM_BP_DIRECT	response to activity	4	1.1	3.8E-2	6.5E-1
GOTERM_BP_DIRECT	heart development	8	2.2	4.0E-2	6.7E-1
GOTERM_BP_DIRECT	circadian rhythm	5	1.3	4.1E-2	6.9E-1
GOTERM_BP_DIRECT	retinal ganglion cell axon guidance	3	0.8	4.2E-2	6.9E-1
GOTERM_BP_DIRECT	regulation of neurogenesis	3	0.8	4.2E-2	6.9E-1
GOTERM_BP_DIRECT	palate development	5	1.3	4.3E-2	7.0E-1
GOTERM_BP_DIRECT	positive regulation of endothelial cell migration	4	1.1	4.5E-2	7.1E-1
GOTERM_BP_DIRECT	middle ear morphogenesis	3	0.8	4.6E-2	7.1E-1
GOTERM_BP_DIRECT	cellular response to glucocorticoid stimulus	3	0.8	4.6E-2	7.1E-1
GOTERM_BP_DIRECT	embryonic placenta development	3	0.8	4.6E-2	7.1E-1
GOTERM_BP_DIRECT	negative regulation of phosphorylation	3	0.8	4.6E-2	7.1E-1
GOTERM_BP_DIRECT	brain development	8	2.2	4.7E-2	7.1E-1
GOTERM_BP_DIRECT	positive regulation of fat cell differentiation	4	1.1	4.8E-2	7.1E-1
GOTERM_BP_DIRECT	cellular response to starvation	4	1.1	4.8E-2	7.1E-1
GOTERM_BP_DIRECT	negative regulation of inflammatory response	5	1.3	4.8E-2	7.1E-1
GOTERM_BP_DIRECT	peptidyl-tyrosine phosphorylation	7	1.9	5.1E-2	7.1E-1
GOTERM_BP_DIRECT	cellular response to gamma radiation	3	0.8	5.1E-2	7.1E-1
GOTERM_BP_DIRECT	response to methionine	2	0.5	5.1E-2	7.1E-1
GOTERM_BP_DIRECT	vascular smooth muscle cell differentiation	2	0.5	5.1E-2	7.1E-1
GOTERM_BP_DIRECT	vitamin D receptor signaling pathway	2	0.5	5.1E-2	7.1E-1
GOTERM_BP_DIRECT	brain segmentation	2	0.5	5.1E-2	7.1E-1
GOTERM_BP_DIRECT	negative regulation of tyrosine phosphorylation of Stat1 protein	2	0.5	5.1E-2	7.1E-1
GOTERM_BP_DIRECT	positive regulation of mast cell cytokine production	2	0.5	5.1E-2	7.1E-1
GOTERM_BP_DIRECT	chaperone-mediated autophagy	2	0.5	5.1E-2	7.1E-1

GOTERM_BP_DIRECT	positive regulation of nucleotide-binding oligomerization domain containing 2 signaling pathway	2	0.5	5.1E-2	7.1E-1
GOTERM_BP_DIRECT	visceral motor neuron differentiation	2	0.5	5.1E-2	7.1E-1
GOTERM_BP_DIRECT	negative regulation of cell growth	6	1.6	5.9E-2	8.2E-1
GOTERM_BP_DIRECT	regulation of growth	4	1.1	6.4E-2	8.6E-1
GOTERM_BP_DIRECT	ionotropic glutamate receptor signaling pathway	3	0.8	6.4E-2	8.6E-1
GOTERM_BP_DIRECT	cellular response to nutrient	2	0.5	6.8E-2	8.6E-1
GOTERM_BP_DIRECT	positive regulation of ureteric bud formation	2	0.5	6.8E-2	8.6E-1
GOTERM_BP_DIRECT	regulation of response to reactive oxygen species	2	0.5	6.8E-2	8.6E-1
GOTERM_BP_DIRECT	regulation of odontogenesis	2	0.5	6.8E-2	8.6E-1
GOTERM_BP_DIRECT	gamma-delta T cell activation	2	0.5	6.8E-2	8.6E-1
GOTERM_BP_DIRECT	positive regulation of cellular pH reduction	2	0.5	6.8E-2	8.6E-1
GOTERM_BP_DIRECT	negative regulation of RNA polymerase II regulatory region sequence-specific DNA binding	2	0.5	6.8E-2	8.6E-1
GOTERM_BP_DIRECT	male sex differentiation	2	0.5	6.8E-2	8.6E-1
GOTERM_BP_DIRECT	positive regulation of alpha-amino-3-hydroxy-5-methyl-4-isoxazole propionate selective glutamate receptor activity	2	0.5	6.8E-2	8.6E-1
GOTERM_BP_DIRECT	T-helper 2 cell differentiation	2	0.5	6.8E-2	8.6E-1
GOTERM_BP_DIRECT	apoptotic process	16	4.3	6.9E-2	8.7E-1
GOTERM_BP_DIRECT	vasculogenesis	4	1.1	7.3E-2	9.2E-1
GOTERM_BP_DIRECT	adult behavior	3	0.8	7.4E-2	9.2E-1
GOTERM_BP_DIRECT	actin cytoskeleton organization	6	1.6	7.6E-2	9.4E-1
GOTERM_BP_DIRECT	protein autophosphorylation	7	1.9	7.8E-2	9.6E-1
GOTERM_BP_DIRECT	response to drug	10	2.7	8.3E-2	9.7E-1
GOTERM_BP_DIRECT	negative regulation of interleukin-2 biosynthetic process	2	0.5	8.4E-2	9.7E-1
GOTERM_BP_DIRECT	positive regulation of endothelial cell chemotaxis	2	0.5	8.4E-2	9.7E-1
GOTERM_BP_DIRECT	organ formation	2	0.5	8.4E-2	9.7E-1
GOTERM_BP_DIRECT	positive regulation of endothelial cell chemotaxis by VEGF-activated vascular endothelial growth factor receptor signaling pathway	2	0.5	8.4E-2	9.7E-1
GOTERM_BP_DIRECT	negative regulation of muscle cell apoptotic process	2	0.5	8.4E-2	9.7E-1
GOTERM_BP_DIRECT	negative regulation of protein homodimerization activity	2	0.5	8.4E-2	9.7E-1
GOTERM_BP_DIRECT	ventricular septum development	3	0.8	8.4E-2	9.7E-1
GOTERM_BP_DIRECT	RNA phosphodiester bond hydrolysis. exonucleolytic	3	0.8	8.4E-2	9.7E-1
GOTERM_BP_DIRECT	regulation of transcription from RNA polymerase II promoter in response to hypoxia	3	0.8	8.4E-2	9.7E-1
GOTERM_BP_DIRECT	positive regulation of smooth muscle cell proliferation	4	1.1	8.6E-2	9.9E-1
GOTERM_BP_DIRECT	heart looping	4	1.1	8.9E-2	1.0E0

GOTERM_BP_DIRECT	nuclear-transcribed mRNA poly(A) tail shortening	3	0.8	9.0E-2	1.0E0
GOTERM_BP_DIRECT	cell morphogenesis	4	1.1	9.3E-2	1.0E0
GOTERM_BP_DIRECT	negative regulation of protein kinase activity	5	1.3	9.3E-2	1.0E0
GOTERM_BP_DIRECT	cellular response to BMP stimulus	3	0.8	9.5E-2	1.0E0
GOTERM_BP_DIRECT	embryonic skeletal system development	3	0.8	9.5E-2	1.0E0
GOTERM_BP_DIRECT	intrinsic apoptotic signaling pathway	3	0.8	9.5E-2	1.0E0
GOTERM_BP_DIRECT	positive regulation of reactive oxygen species metabolic process	3	0.8	9.5E-2	1.0E0
GOTERM_BP_DIRECT	regulation of gene expression	5	1.3	9.6E-2	1.0E0
GOTERM_BP_DIRECT	embryonic process involved in female pregnancy	2	0.5	1.0E-1	1.0E0
GOTERM_BP_DIRECT	regulation of transcription from RNA polymerase II promoter in response to oxidative stress	2	0.5	1.0E-1	1.0E0
GOTERM_BP_DIRECT	positive regulation of tumor necrosis factor-mediated signaling pathway	2	0.5	1.0E-1	1.0E0
GOTERM_BP_DIRECT	positive regulation of cell migration	7	1.9	1.0E-1	1.0E0

**siLonp1 - HS vs Ctrl**					
Category	Term	Count	%	P-Value	Benjamini
**GOTERM_BP_DIRECT**	**response to unfolded protein**	**16**	**3.6**	**1.3E-15**	**2.6E-12**
**GOTERM_BP_DIRECT**	**transcription. DNA-templated**	**88**	**20.0**	**1.8E-13**	**1.8E-10**
**GOTERM_BP_DIRECT**	**regulation of transcription. DNA-templated**	**71**	**16.1**	**1.1E-11**	**7.1E-9**
**GOTERM_BP_DIRECT**	**negative regulation of transcription from RNA polymerase II promoter**	**43**	**9.8**	**3.9E-10**	**1.9E-7**
**GOTERM_BP_DIRECT**	**positive regulation of transcription from RNA polymerase II promoter**	**46**	**10.5**	**1.4E-7**	**5.5E-5**
**GOTERM_BP_DIRECT**	**protein refolding**	**7**	**1.6**	**2.5E-7**	**7.3E-5**
**GOTERM_BP_DIRECT**	**regulation of cellular response to heat**	**12**	**2.7**	**2.6E-7**	**7.3E-5**
**GOTERM_BP_DIRECT**	**negative regulation of inclusion body assembly**	**5**	**1.1**	**2.9E-5**	**7.3E-3**
**GOTERM_BP_DIRECT**	**transcription from RNA polymerase II promoter**	**26**	**5.9**	**3.7E-5**	**8.1E-3**
**GOTERM_BP_DIRECT**	**skeletal muscle cell differentiation**	**8**	**1.8**	**4.8E-5**	**9.6E-3**
**GOTERM_BP_DIRECT**	**cellular response to vascular endothelial growth factor stimulus**	**6**	**1.4**	**7.6E-5**	**1.4E-2**
**GOTERM_BP_DIRECT**	**regulation of protein ubiquitination**	**5**	**1.1**	**2.3E-4**	**3.8E-2**
**GOTERM_BP_DIRECT**	**protein folding**	**13**	**3.0**	**2.6E-4**	**4.0E-2**
**GOTERM_BP_DIRECT**	**response to heat**	**7**	**1.6**	**3.6E-4**	**5.1E-2**
GOTERM_BP_DIRECT	negative regulation of transcription. DNA-templated	23	5.2	4.4E-4	5.8E-2
GOTERM_BP_DIRECT	regulation of cell death	4	0.9	1.1E-3	1.4E-1
GOTERM_BP_DIRECT	response to methionine	3	0.7	1.2E-3	1.4E-1
GOTERM_BP_DIRECT	PERK-mediated unfolded protein response	4	0.9	1.5E-3	1.5E-1
GOTERM_BP_DIRECT	positive regulation of transcription from RNA polymerase II promoter in response to endoplasmic reticulum stress	4	0.9	1.5E-3	1.5E-1

GOTERM_BP_DIRECT	endoderm formation	4	0.9	1.5E-3	1.5E-1
GOTERM_BP_DIRECT	positive regulation of transcription. DNA-templated	22	5.0	1.6E-3	1.5E-1
GOTERM_BP_DIRECT	multicellular organism development	22	5.0	1.8E-3	1.6E-1
GOTERM_BP_DIRECT	negative regulation of apoptotic process	20	4.5	2.0E-3	1.6E-1
GOTERM_BP_DIRECT	positive regulation of angiogenesis	9	2.0	2.1E-3	1.6E-1
GOTERM_BP_DIRECT	response to insulin	7	1.6	2.1E-3	1.6E-1
GOTERM_BP_DIRECT	patterning of blood vessels	5	1.1	2.2E-3	1.6E-1
GOTERM_BP_DIRECT	cellular heat acclimation	3	0.7	2.3E-3	1.7E-1
GOTERM_BP_DIRECT	protein localization to nucleus	5	1.1	2.8E-3	1.9E-1
GOTERM_BP_DIRECT	cellular response to fibroblast growth factor stimulus	5	1.1	2.8E-3	1.9E-1
GOTERM_BP_DIRECT	cellular response to interleukin-1	7	1.6	2.9E-3	1.9E-1
GOTERM_BP_DIRECT	negative regulation of cell growth	9	2.0	2.9E-3	1.9E-1
GOTERM_BP_DIRECT	positive regulation of endothelial cell chemotaxis by VEGF-activated vascular endothelial growth factor receptor signaling pathway	3	0.7	3.8E-3	2.4E-1
GOTERM_BP_DIRECT	odontogenesis of dentin-containing tooth	6	1.4	4.7E-3	2.8E-1
GOTERM_BP_DIRECT	response to cold	5	1.1	5.5E-3	3.2E-1
GOTERM_BP_DIRECT	response to lipopolysaccharide	10	2.3	5.6E-3	3.2E-1
GOTERM_BP_DIRECT	protein stabilization	9	2.0	5.9E-3	3.2E-1
GOTERM_BP_DIRECT	positive regulation of osteoclast differentiation	4	0.9	6.0E-3	3.2E-1
GOTERM_BP_DIRECT	cellular response to tumor necrosis factor	8	1.8	6.4E-3	3.4E-1
GOTERM_BP_DIRECT	regulation of transcription from RNA polymerase II promoter	18	4.1	7.4E-3	3.8E-1
GOTERM_BP_DIRECT	transforming growth factor beta receptor signaling pathway	7	1.6	1.0E-2	5.0E-1
GOTERM_BP_DIRECT	negative regulation of myoblast differentiation	4	0.9	1.0E-2	5.0E-1
GOTERM_BP_DIRECT	regulation of apoptotic process	11	2.5	1.0E-2	5.0E-1
GOTERM_BP_DIRECT	protein ubiquitination involved in ubiquitin-dependent protein catabolic process	9	2.0	1.2E-2	5.1E-1
GOTERM_BP_DIRECT	neuron differentiation	7	1.6	1.2E-2	5.1E-1
GOTERM_BP_DIRECT	positive regulation of endothelial cell proliferation	6	1.4	1.2E-2	5.1E-1
GOTERM_BP_DIRECT	negative regulation of cysteine-type endopeptidase activity involved in apoptotic process	6	1.4	1.2E-2	5.1E-1
GOTERM_BP_DIRECT	cellular response to hormone stimulus	5	1.1	1.2E-2	5.1E-1
GOTERM_BP_DIRECT	endothelial cell chemotaxis	3	0.7	1.3E-2	5.2E-1
GOTERM_BP_DIRECT	inactivation of MAPK activity	4	0.9	1.3E-2	5.2E-1
GOTERM_BP_DIRECT	forebrain development	5	1.1	1.3E-2	5.2E-1
GOTERM_BP_DIRECT	organ regeneration	5	1.1	1.4E-2	5.3E-1
GOTERM_BP_DIRECT	cellular response to amino acid stimulus	5	1.1	1.4E-2	5.3E-1
GOTERM_BP_DIRECT	positive regulation of interleukin-8 production	4	0.9	1.4E-2	5.3E-1
GOTERM_BP_DIRECT	actin cytoskeleton organization	8	1.8	1.5E-2	5.3E-1

GOTERM_BP_DIRECT	chaperone mediated protein folding requiring cofactor	3	0.7	1.6E-2	5.3E-1
GOTERM_BP_DIRECT	T cell differentiation in thymus	4	0.9	1.6E-2	5.3E-1
GOTERM_BP_DIRECT	JNK cascade	5	1.1	1.6E-2	5.3E-1
GOTERM_BP_DIRECT	negative regulation of neuron apoptotic process	8	1.8	1.6E-2	5.3E-1
GOTERM_BP_DIRECT	circadian rhythm	6	1.4	1.7E-2	5.3E-1
GOTERM_BP_DIRECT	response to endoplasmic reticulum stress	6	1.4	1.7E-2	5.3E-1
GOTERM_BP_DIRECT	regulation of mRNA stability	7	1.6	1.7E-2	5.3E-1
GOTERM_BP_DIRECT	positive regulation of NF-kappaB transcription factor activity	8	1.8	1.7E-2	5.3E-1
GOTERM_BP_DIRECT	positive regulation of apoptotic process	13	3.0	1.7E-2	5.3E-1
GOTERM_BP_DIRECT	palate development	6	1.4	1.8E-2	5.3E-1
GOTERM_BP_DIRECT	regulation of transcription from RNA polymerase II promoter in response to hypoxia	4	0.9	1.8E-2	5.3E-1
GOTERM_BP_DIRECT	embryonic hindlimb morphogenesis	4	0.9	1.8E-2	5.3E-1
GOTERM_BP_DIRECT	response to hydrogen peroxide	5	1.1	1.8E-2	5.4E-1
GOTERM_BP_DIRECT	regulation of ossification	3	0.7	1.9E-2	5.4E-1
GOTERM_BP_DIRECT	negative regulation of Notch signaling pathway	4	0.9	1.9E-2	5.4E-1
GOTERM_BP_DIRECT	regulation of RNA splicing	4	0.9	1.9E-2	5.4E-1
GOTERM_BP_DIRECT	ventricular septum morphogenesis	4	0.9	1.9E-2	5.4E-1
GOTERM_BP_DIRECT	anterior/posterior pattern specification	6	1.4	2.2E-2	6.0E-1
GOTERM_BP_DIRECT	response to hypoxia	9	2.0	2.2E-2	6.0E-1
GOTERM_BP_DIRECT	positive regulation of ATPase activity	4	0.9	2.3E-2	6.2E-1
GOTERM_BP_DIRECT	cellular response to DNA damage stimulus	10	2.3	2.4E-2	6.3E-1
GOTERM_BP_DIRECT	brown fat cell differentiation	4	0.9	2.5E-2	6.6E-1
GOTERM_BP_DIRECT	cellular response to lipopolysaccharide	7	1.6	2.5E-2	6.6E-1
GOTERM_BP_DIRECT	MyD88-independent toll-like receptor signaling pathway	3	0.7	2.7E-2	6.7E-1
GOTERM_BP_DIRECT	chaperone-mediated protein complex assembly	3	0.7	2.7E-2	6.7E-1
GOTERM_BP_DIRECT	intrinsic apoptotic signaling pathway in response to endoplasmic reticulum stress	4	0.9	2.7E-2	6.8E-1
GOTERM_BP_DIRECT	I-kappaB kinase/NF-kappaB signaling	5	1.1	3.1E-2	7.7E-1
GOTERM_BP_DIRECT	response to starvation	4	0.9	3.2E-2	7.7E-1
GOTERM_BP_DIRECT	muscle organ development	6	1.4	3.2E-2	7.7E-1
GOTERM_BP_DIRECT	response to stress	5	1.1	3.3E-2	7.8E-1
GOTERM_BP_DIRECT	DNA damage response. signal transduction by p53 class mediator resulting in cell cycle arrest	5	1.1	3.5E-2	7.9E-1
GOTERM_BP_DIRECT	angiogenesis	10	2.3	3.5E-2	7.9E-1
GOTERM_BP_DIRECT	induction of positive chemotaxis	3	0.7	3.5E-2	7.9E-1
GOTERM_BP_DIRECT	cell migration involved in sprouting angiogenesis	3	0.7	3.5E-2	7.9E-1
GOTERM_BP_DIRECT	negative regulation of T cell proliferation	4	0.9	3.7E-2	8.0E-1

GOTERM_BP_DIRECT	cellular response to heat	4	0.9	3.7E-2	8.0E-1
GOTERM_BP_DIRECT	negative regulation of transforming growth factor beta receptor signaling pathway	5	1.1	3.8E-2	8.0E-1
GOTERM_BP_DIRECT	cellular response to oxidative stress	5	1.1	3.8E-2	8.0E-1
GOTERM_BP_DIRECT	negative regulation of establishment of protein localization to mitochondrion	2	0.5	3.9E-2	8.0E-1
GOTERM_BP_DIRECT	positive regulation of mRNA endonucleolytic cleavage involved in unfolded protein response	2	0.5	3.9E-2	8.0E-1
GOTERM_BP_DIRECT	positive regulation of endoribonuclease activity	2	0.5	3.9E-2	8.0E-1
GOTERM_BP_DIRECT	positive regulation of p38MAPK cascade	3	0.7	3.9E-2	8.0E-1
GOTERM_BP_DIRECT	intermediate filament organization	3	0.7	3.9E-2	8.0E-1
GOTERM_BP_DIRECT	ERBB2 signaling pathway	4	0.9	3.9E-2	8.0E-1
GOTERM_BP_DIRECT	response to progesterone	4	0.9	4.2E-2	8.4E-1
GOTERM_BP_DIRECT	blood vessel morphogenesis	3	0.7	4.4E-2	8.8E-1
GOTERM_BP_DIRECT	response to glucose	5	1.1	4.6E-2	9.1E-1
GOTERM_BP_DIRECT	negative regulation of protein ubiquitination	4	0.9	4.8E-2	9.3E-1
GOTERM_BP_DIRECT	response to corticosterone	3	0.7	4.9E-2	9.4E-1
GOTERM_BP_DIRECT	regulation of gene expression	6	1.4	4.9E-2	9.4E-1
GOTERM_BP_DIRECT	thymus development	4	0.9	5.4E-2	9.5E-1
GOTERM_BP_DIRECT	response to activity	4	0.9	5.4E-2	9.5E-1
GOTERM_BP_DIRECT	negative regulation of endoplasmic reticulum stress-induced intrinsic apoptotic signaling pathway	3	0.7	5.4E-2	9.5E-1
GOTERM_BP_DIRECT	positive regulation of blood vessel endothelial cell migration	3	0.7	5.4E-2	9.5E-1
GOTERM_BP_DIRECT	retinal ganglion cell axon guidance	3	0.7	5.4E-2	9.5E-1
GOTERM_BP_DIRECT	regulation of neurogenesis	3	0.7	5.4E-2	9.5E-1
GOTERM_BP_DIRECT	vascular endothelial growth factor receptor signaling pathway	5	1.1	5.5E-2	9.5E-1
GOTERM_BP_DIRECT	protein autophosphorylation	8	1.8	5.7E-2	9.5E-1
GOTERM_BP_DIRECT	fat cell differentiation	5	1.1	5.7E-2	9.5E-1
GOTERM_BP_DIRECT	small GTPase mediated signal transduction	10	2.3	5.8E-2	9.5E-1
GOTERM_BP_DIRECT	visceral motor neuron differentiation	2	0.5	5.8E-2	9.5E-1
GOTERM_BP_DIRECT	cellular response to ionomycin	2	0.5	5.8E-2	9.5E-1
GOTERM_BP_DIRECT	brain segmentation	2	0.5	5.8E-2	9.5E-1
GOTERM_BP_DIRECT	chaperone-mediated autophagy	2	0.5	5.8E-2	9.5E-1
GOTERM_BP_DIRECT	positive regulation of mast cell cytokine production	2	0.5	5.8E-2	9.5E-1
GOTERM_BP_DIRECT	positive regulation of nucleotide-binding oligomerization domain containing 2 signaling pathway	2	0.5	5.8E-2	9.5E-1
GOTERM_BP_DIRECT	positive regulation of intrinsic apoptotic signaling pathway by p53 class mediator	2	0.5	5.8E-2	9.5E-1
GOTERM_BP_DIRECT	neuron migration	6	1.4	5.9E-2	9.5E-1
GOTERM_BP_DIRECT	middle ear morphogenesis	3	0.7	5.9E-2	9.5E-1

GOTERM_BP_DIRECT	cellular response to glucocorticoid stimulus	3	0.7	5.9E-2	9.5E-1
GOTERM_BP_DIRECT	positive regulation of interferon-gamma production	4	0.9	6.3E-2	9.9E-1
GOTERM_BP_DIRECT	outflow tract morphogenesis	4	0.9	6.3E-2	9.9E-1
GOTERM_BP_DIRECT	positive regulation of endothelial cell migration	4	0.9	6.3E-2	9.9E-1
GOTERM_BP_DIRECT	stress-activated MAPK cascade	3	0.7	6.5E-2	1.0E0
GOTERM_BP_DIRECT	cellular response to gamma radiation	3	0.7	6.5E-2	1.0E0
GOTERM_BP_DIRECT	cellular response to starvation	4	0.9	6.7E-2	1.0E0
GOTERM_BP_DIRECT	positive regulation of fat cell differentiation	4	0.9	6.7E-2	1.0E0
GOTERM_BP_DIRECT	xenobiotic metabolic process	5	1.1	7.0E-2	1.0E0
GOTERM_BP_DIRECT	negative regulation of cyclin-dependent protein serine/threonine kinase activity	3	0.7	7.0E-2	1.0E0
GOTERM_BP_DIRECT	inflammatory response	13	3.0	7.5E-2	1.0E0
GOTERM_BP_DIRECT	negative regulation of peptidyl-serine phosphorylation	3	0.7	7.6E-2	1.0E0
GOTERM_BP_DIRECT	regulation of odontogenesis	2	0.5	7.7E-2	1.0E0
GOTERM_BP_DIRECT	male sex differentiation	2	0.5	7.7E-2	1.0E0
GOTERM_BP_DIRECT	negative regulation of RNA polymerase II regulatory region sequence-specific DNA binding	2	0.5	7.7E-2	1.0E0
GOTERM_BP_DIRECT	positive regulation of alpha-amino-3-hydroxy-5-methyl-4-isoxazole propionate selective glutamate receptor activity	2	0.5	7.7E-2	1.0E0
GOTERM_BP_DIRECT	gamma-delta T cell activation	2	0.5	7.7E-2	1.0E0
GOTERM_BP_DIRECT	regulation of response to reactive oxygen species	2	0.5	7.7E-2	1.0E0
GOTERM_BP_DIRECT	in utero embryonic development	8	1.8	8.1E-2	1.0E0
GOTERM_BP_DIRECT	cellular response to calcium ion	4	0.9	8.1E-2	1.0E0
GOTERM_BP_DIRECT	ionotropic glutamate receptor signaling pathway	3	0.7	8.2E-2	1.0E0
GOTERM_BP_DIRECT	peripheral nervous system development	3	0.7	8.2E-2	1.0E0
GOTERM_BP_DIRECT	peptidyl-tyrosine phosphorylation	7	1.6	8.5E-2	1.0E0
GOTERM_BP_DIRECT	brain development	8	1.8	8.6E-2	1.0E0
GOTERM_BP_DIRECT	ventricular cardiac muscle tissue morphogenesis	3	0.7	8.8E-2	1.0E0
GOTERM_BP_DIRECT	adult behavior	3	0.7	9.4E-2	1.0E0
GOTERM_BP_DIRECT	glycogen biosynthetic process	3	0.7	9.4E-2	1.0E0
GOTERM_BP_DIRECT	apoptotic nuclear changes	2	0.5	9.6E-2	1.0E0
GOTERM_BP_DIRECT	protein import into mitochondrial outer membrane	2	0.5	9.6E-2	1.0E0
GOTERM_BP_DIRECT	positive regulation of endothelial cell chemotaxis	2	0.5	9.6E-2	1.0E0
GOTERM_BP_DIRECT	positive regulation of histone deacetylase activity	2	0.5	9.6E-2	1.0E0
GOTERM_BP_DIRECT	negative regulation of protein homodimerization activity	2	0.5	9.6E-2	1.0E0
GOTERM_BP_DIRECT	organ formation	2	0.5	9.6E-2	1.0E0
GOTERM_BP_DIRECT	cellular response to fructose stimulus	2	0.5	9.6E-2	1.0E0

GOTERM_BP_DIRECT	negative regulation of interleukin-2 biosynthetic process	2	0.5	9.6E-2	1.0E0
