# Supplementary material for: Evidence for mitochondrial Lonp1 expression in the nucleus
Source: Sci Rep. 2022 Jun 27;12:10877. doi: 10.1038/s41598-022-14860-0 (PMC9237102; doi:10.1038/s41598-022-14860-0)
Supplement: Supplementary file 4 — Supplementary Table 2. [file 41598_2022_14860_MOESM4_ESM.pdf]

Supplementary Table 2

HS vs Ctrl Scramble							
Term	Overlap	P-value	Adjusted P-value	Old P-value	Old Adjusted P-value	Odds Ratio	Combined Score
**Attenuation phase Homo sapiens R-HSA-3371568**	**12/26**	**1.25E-14**	**8.13E-12**	**0**	**0**	**46.17739**	**1478.384**
**HSF1-dependent transactivation Homo sapiens R-HSA-3371571**	**12/34**	**6.18E-13**	**2.02E-10**	**0**	**0**	**29.37363**	**825.7373**
**HSF1 activation Homo sapiens R-HSA-3371511**	**11/29**	**2.29E-12**	**4.98E-10**	**0**	**0**	**32.82588**	**879.8154**
**Regulation of HSF1-mediated heat shock response Homo sapiens R-HSA-3371453**	**14/80**	**2.65E-10**	**4.20E-08**	**0**	**0**	**11.46041**	**252.6976**
**Cellular response to heat stress Homo sapiens R-HSA-3371556**	**15/96**	**3.22E-10**	**4.20E-08**	**0**	**0**	**10.02514**	**219.1085**
**Generic Transcription Pathway Homo sapiens R-HSA-212436**	**43/812**	**9.73E-10**	**1.06E-07**	**0**	**0**	**3.166098**	**65.69711**
**Cellular responses to stress Homo sapiens R-HSA-2262752**	**24/367**	**1.37E-07**	**1.28E-05**	**0**	**0**	**3.832693**	**60.56399**
**Gene Expression Homo sapiens R-HSA-74160**	**54/1631**	**3.22E-05**	**0.002628**	**0**	**0**	**1.91916**	**19.8481**
RAF-independent MAPK1/3 activation Homo sapiens R-HSA-112409	4/23	8.20E-04	0.057112	0	0	11.09508	78.84095
Negative regulation of MAPK pathway Homo sapiens R-HSA-5675221	5/40	8.76E-04	0.057112	0	0	7.542934	53.10378
Developmental Biology Homo sapiens R-HSA-1266738	27/786	0.002001	0.111746	0	0	1.922885	11.9491
Receptor-ligand binding initiates the second proteolytic cleavage of Notch receptor Homo sapiens R-HSA-156988	3/14	0.002057	0.111746	0	0	14.34048	88.71977
Constitutive Signaling by NOTCH1 HD Domain Mutants Homo sapiens R-HSA-2691232	3/15	0.002535	0.11807	0	0	13.14477	78.57242
Signaling by NOTCH1 HD Domain Mutants in Cancer Homo sapiens R-HSA-2691230	3/15	0.002535	0.11807	0	0	13.14477	78.57242
SMAD2/SMAD3:SMAD4 heterotrimer regulates transcription Homo sapiens R-HSA-2173796	4/32	0.002916	0.126748	0	0	7.525346	43.92956

Senescence-Associated Secretory Phenotype (SASP) Homo sapiens R-HSA-2559582	6/77	0.003276	0.133479	0	0	4.465855	25.55033
Scavenging by Class F Receptors Homo sapiens R-HSA-3000484	2/6	0.005029	0.182178	0	0	26.22995	138.8205
Uptake and function of diphtheria toxin Homo sapiens R-HSA-5336415	2/6	0.005029	0.182178	0	0	26.22995	138.8205
NOTCH2 Activation and Transmission of Signal to the Nucleus Homo sapiens R-HSA-2979096	3/21	0.006818	0.215906	0	0	8.7605	43.6992
Signaling by NOTCH1 t(7;9)(NOTCH1:M1580 K2555) Translocation Mutant Homo sapiens R-HSA-2660825	2/7	0.006954	0.215906	0	0	20.98289	104.2521
Constitutive Signaling by NOTCH1 t(7;9)(NOTCH1:M1580 K2555) Translocation Mutant Homo sapiens R-HSA-2660826	2/7	0.006954	0.215906	0	0	20.98289	104.2521
MAPK family signaling cascades Homo sapiens R-HSA-5683057	12/284	0.007782	0.230619	0	0	2.345507	11.38976
Transcriptional activity of SMAD2/SMAD3:SMAD4 heterotrimer Homo sapiens R-HSA-2173793	4/44	0.00923	0.261656	0	0	5.264516	24.6657
Signaling by TGF-beta Receptor Complex Homo sapiens R-HSA-170834	5/73	0.012098	0.290601	0	0	3.875852	17.11064
Downregulation of TGF-beta receptor signaling Homo sapiens R-HSA-2173788	3/26	0.012438	0.290601	0	0	6.854295	30.06971
Regulation of Hypoxia-inducible Factor (HIF) by oxygen Homo sapiens R-HSA-1234174	3/26	0.012438	0.290601	0	0	6.854295	30.06971
Cellular response to hypoxia Homo sapiens R-HSA-2262749	3/26	0.012438	0.290601	0	0	6.854295	30.06971
TP53 Regulates Transcription of Cell Cycle Genes Homo sapiens R-HSA-6791312	4/48	0.01248	0.290601	0	0	4.784946	20.9755
POU5F1 (OCT4). SOX2. NANOG repress genes related to differentiation Homo sapiens R-HSA-2892245	2/10	0.014356	0.312006	0	0	13.1123	55.64309
Regulation of gene expression by Hypoxia-inducible Factor	2/10	0.014356	0.312006	0	0	13.1123	55.64309

Homo sapiens R-HSA-1234158							
Transcriptional Regulation by TP53 Homo sapiens R-HSA-3700989	13/348	0.015013	0.313871	0	0	2.062062	8.658324
Signaling by Leptin Homo sapiens R-HSA-2586552	10/243	0.017062	0.313871	0	0	2.273857	9.256648
Signaling by NOTCH3 Homo sapiens R-HSA-1980148	2/11	0.01733	0.313871	0	0	11.65478	47.26361
Signaling by NOTCH4 Homo sapiens R-HSA-1980150	2/11	0.01733	0.313871	0	0	11.65478	47.26361
Membrane binding and targetting of GAG proteins Homo sapiens R-HSA-174490	2/11	0.01733	0.313871	0	0	11.65478	47.26361
Synthesis And Processing Of GAG. GAGPOL Polyproteins Homo sapiens R-HSA-174495	2/11	0.01733	0.313871	0	0	11.65478	47.26361
Activated NOTCH1 Transmits Signal to the Nucleus Homo sapiens R-HSA-2122948	3/30	0.018382	0.318935	0	0	5.837653	23.32934
AUF1 (hnRNP D0) binds and destabilizes mRNA Homo sapiens R-HSA-450408	4/54	0.018588	0.318935	0	0	4.209462	16.77566
Reversible hydration of carbon dioxide Homo sapiens R-HSA-1475029	2/12	0.020541	0.325244	0	0	10.48877	40.75249
Signaling by SCF-KIT Homo sapiens R-HSA-1433557	12/325	0.020609	0.325244	0	0	2.03395	7.895881
TGF-beta receptor signaling activates SMADs Homo sapiens R-HSA-2173789	3/32	0.02185	0.325244	0	0	5.434501	20.7792
Signaling by NOTCH2 Homo sapiens R-HSA-1980145	3/32	0.02185	0.325244	0	0	5.434501	20.7792
Oncogene Induced Senescence Homo sapiens R-HSA-2559585	3/32	0.02185	0.325244	0	0	5.434501	20.7792
Signaling by VEGF Homo sapiens R-HSA-194138	12/328	0.021949	0.325244	0	0	2.014327	7.692787
Regulation of mRNA stability by proteins that bind AU-rich elements Homo sapiens R-HSA-450531	5/86	0.023069	0.334243	0	0	3.251639	12.2563
Assembly Of The HIV Virion Homo sapiens R-HSA-175474	2/13	0.023977	0.339855	0	0	9.534759	35.57075
Regulation of IFNG signaling Homo sapiens R-HSA-877312	2/14	0.027631	0.378425	0	0	8.73975	31.36537
NCAM signaling for neurite out-growth Homo sapiens R-HSA-375165	10/266	0.029548	0.378425	0	0	2.067111	7.27984
Cellular Senescence Homo sapiens R-HSA-2559583	7/161	0.032818	0.378425	0	0	2.398374	8.194713

GRB2 events in EGFR signaling Homo sapiens R-HSA-179812	9/235	0.034216	0.378425	0	0	2.104868	7.104062
SHC1 events in EGFR signaling Homo sapiens R-HSA-180336	9/235	0.034216	0.378425	0	0	2.104868	7.104062
SOS-mediated signalling Homo sapiens R-HSA-112412	9/235	0.034216	0.378425	0	0	2.104868	7.104062
SHC1 events in ERBB4 signaling Homo sapiens R-HSA-1250347	9/235	0.034216	0.378425	0	0	2.104868	7.104062
RAF/MAP kinase cascade Homo sapiens R-HSA-5673001	9/235	0.034216	0.378425	0	0	2.104868	7.104062
G1 Phase Homo sapiens R-HSA-69236	3/38	0.034237	0.378425	0	0	4.501494	15.19001
Cyclin D associated events in G1 Homo sapiens R-HSA-69231	3/38	0.034237	0.378425	0	0	4.501494	15.19001
FRS-mediated FGFR2 signaling Homo sapiens R-HSA-5654700	9/236	0.03501	0.378425	0	0	2.095488	7.024327
FRS-mediated FGFR4 signaling Homo sapiens R-HSA-5654712	9/236	0.03501	0.378425	0	0	2.095488	7.024327
FRS-mediated FGFR3 signaling Homo sapiens R-HSA-5654706	9/236	0.03501	0.378425	0	0	2.095488	7.024327
FRS-mediated FGFR1 signaling Homo sapiens R-HSA-5654693	9/236	0.03501	0.378425	0	0	2.095488	7.024327
Fc epsilon receptor (FCERI) signaling Homo sapiens R-HSA-2454202	13/395	0.036958	0.378425	0	0	1.803946	5.94936
Signaling by PTK6 Homo sapiens R-HSA-8848021	4/67	0.037338	0.378425	0	0	3.338624	10.97653
ARMS-mediated activation Homo sapiens R-HSA-170984	9/239	0.037468	0.378425	0	0	2.067836	6.79132
Signalling to p38 via RIT and RIN Homo sapiens R-HSA-187706	9/239	0.037468	0.378425	0	0	2.067836	6.79132
Frs2-mediated activation Homo sapiens R-HSA-170968	9/240	0.038313	0.378425	0	0	2.058778	6.715661
MAPK1/MAPK3 signaling Homo sapiens R-HSA-5684996	9/241	0.039171	0.378425	0	0	2.049798	6.640981
Unblocking of NMDA receptor. glutamate binding and activation Homo sapiens R-HSA-438066	2/17	0.0398	0.378425	0	0	6.990731	22.53734
Prolonged ERK activation events Homo sapiens R-HSA-169893	9/242	0.040042	0.378425	0	0	2.040895	6.567265

VEGFA-VEGFR2 Pathway Homo sapiens R-HSA-4420097	11/320	0.040404	0.378425	0	0	1.883805	6.044797
Interleukin receptor SHC signaling Homo sapiens R-HSA-912526	9/245	0.042733	0.378425	0	0	2.01464	6.351746
Signalling to RAS Homo sapiens R-HSA-167044	9/246	0.043656	0.378425	0	0	2.006036	6.281734
Transcriptional regulation of pluripotent stem cells Homo sapiens R-HSA-452723	3/42	0.044126	0.378425	0	0	4.038977	12.60444
Oxygen-dependent proline hydroxylation of Hypoxia-inducible Factor Alpha Homo sapiens R-HSA-1234176	2/18	0.04423	0.378425	0	0	6.553476	20.43607
VEGFR2 mediated cell proliferation Homo sapiens R-HSA-5218921	9/248	0.045543	0.378425	0	0	1.989044	6.144369
FCERI mediated MAPK activation Homo sapiens R-HSA-2871796	10/289	0.047444	0.378425	0	0	1.894451	5.774678
Downstream signaling of activated FGFR2 Homo sapiens R-HSA-5654696	11/329	0.047627	0.378425	0	0	1.829637	5.570058
Downstream signaling of activated FGFR4 Homo sapiens R-HSA-5654716	11/329	0.047627	0.378425	0	0	1.829637	5.570058
Downstream signaling of activated FGFR3 Homo sapiens R-HSA-5654708	11/329	0.047627	0.378425	0	0	1.829637	5.570058
Signaling by ERBB4 Homo sapiens R-HSA-1236394	11/330	0.048481	0.378425	0	0	1.823807	5.519918
Regulation of TP53 Activity through Methylation Homo sapiens R-HSA-6804760	2/19	0.048833	0.378425	0	0	6.167663	18.62238
Constitutive Signaling by Ligand-Responsive EGFR Cancer Variants Homo sapiens R-HSA-1236382	2/19	0.048833	0.378425	0	0	6.167663	18.62238
Signaling by Ligand-Responsive EGFR Variants in Cancer Homo sapiens R-HSA-5637815	2/19	0.048833	0.378425	0	0	6.167663	18.62238
Signaling by EGFR in Cancer Homo sapiens R-HSA-1643713	2/19	0.048833	0.378425	0	0	6.167663	18.62238
Interleukin-2 signaling Homo sapiens R-HSA-451927	9/252	0.049478	0.378425	0	0	1.955899	5.879891
Signaling by FGFR4 Homo sapiens R-HSA-5654743	11/332	0.050219	0.378425	0	0	1.812256	5.42113

Downstream signaling of activated FGFR1 Homo sapiens R-HSA-5654687	11/332	0.050219	0.378425	0	0	1.812256	5.42113
Signalling to ERKs Homo sapiens R-HSA-187687	9/253	0.050495	0.378425	0	0	1.947782	5.815832
Signaling by FGFR3 Homo sapiens R-HSA-5654741	11/333	0.051103	0.378628	0	0	1.806535	5.37247
Signaling by ERBB2 Homo sapiens R-HSA-1227986	3/45	0.052372	0.383667	0	0	3.749904	11.05992
Regulation of gene expression in beta cells Homo sapiens R-HSA-210745	2/20	0.0536	0.38561	0	0	5.824718	17.04432
Signaling by FGFR1 Homo sapiens R-HSA-5654736	11/336	0.05382	0.38561	0	0	1.789581	5.229359
TP53 regulates transcription of additional cell cycle genes whose exact role in the p53 pathway remain uncertain Homo sapiens R-HSA-6804115	2/21	0.058524	0.405848	0	0	5.517872	15.66143
Aflatoxin activation and detoxification Homo sapiens R-HSA-5423646	2/21	0.058524	0.405848	0	0	5.517872	15.66143
Downstream signal transduction Homo sapiens R-HSA-186763	11/341	0.058559	0.405848	0	0	1.762009	5.000076
Interleukin-3. 5 and GM-CSF signaling Homo sapiens R-HSA-512988	9/261	0.059134	0.405848	0	0	1.885169	5.331152
DAP12 signaling Homo sapiens R-HSA-2424491	11/344	0.061532	0.414359	0	0	1.745864	4.867816
Transcriptional regulation of white adipocyte differentiation Homo sapiens R-HSA-381340	4/79	0.061645	0.414359	0	0	2.802724	7.809386
Axon guidance Homo sapiens R-HSA-422475	15/515	0.063552	0.41885	0	0	1.589252	4.379807
SALM protein interactions at the synapse Homo sapiens R-HSA-8849932	2/22	0.063598	0.41885	0	0	5.241711	14.44179
Signaling by Interleukins Homo sapiens R-HSA-449147	12/392	0.067994	0.443321	0	0	1.66952	4.48823
Signaling by BMP Homo sapiens R-HSA-201451	2/23	0.068814	0.444227	0	0	4.991851	13.35991
Nuclear Receptor transcription pathway Homo sapiens R-HSA-383280	3/51	0.070891	0.447543	0	0	3.280161	8.681303
Signaling by EGFR Homo sapiens R-HSA-177929	11/355	0.07327	0.447543	0	0	1.689073	4.414566
Growth hormone receptor signaling Homo sapiens R-HSA-982772	2/24	0.074165	0.447543	0	0	4.764706	12.39519

DAP12 interactions Homo sapiens R-HSA-2172127	11/359	0.077869	0.447543	0	0	1.669312	4.261291
Cytokine Signaling in Immune system Homo sapiens R-HSA-1280215	17/620	0.078441	0.447543	0	0	1.493725	3.802144
PI3K/AKT Signaling in Cancer Homo sapiens R-HSA-2219528	4/86	0.078883	0.447543	0	0	2.562549	6.508343
ATF4 activates genes Homo sapiens R-HSA-380994	2/25	0.079644	0.447543	0	0	4.557312	11.53084
Constitutive Signaling by AKT1 E17K in Cancer Homo sapiens R-HSA-5674400	2/25	0.079644	0.447543	0	0	4.557312	11.53084
Regulation of IFNA signaling Homo sapiens R-HSA-912694	2/25	0.079644	0.447543	0	0	4.557312	11.53084
Budding and maturation of HIV virion Homo sapiens R-HSA-162588	2/25	0.079644	0.447543	0	0	4.557312	11.53084
Signaling by FGFR2 Homo sapiens R-HSA-5654738	11/361	0.080236	0.447543	0	0	1.659601	4.18682
Signaling by PDGF Homo sapiens R-HSA-186797	11/364	0.083869	0.447543	0	0	1.64524	4.077733
Signaling by FGFR Homo sapiens R-HSA-190236	11/366	0.086347	0.447543	0	0	1.635802	4.00671
IRS-mediated signalling Homo sapiens R-HSA-112399	9/284	0.089015	0.447543	0	0	1.72545	4.173773
Abacavir metabolism Homo sapiens R-HSA-2161541	1/5	0.09054	0.447543	0	0	13.08	31.41771
Na+-dependent glucose transporters Homo sapiens R-HSA-428808	1/5	0.09054	0.447543	0	0	13.08	31.41771
The fatty acid cycling model Homo sapiens R-HSA-167826	1/5	0.09054	0.447543	0	0	13.08	31.41771
The proton buffering model Homo sapiens R-HSA-167827	1/5	0.09054	0.447543	0	0	13.08	31.41771
Mitochondrial Uncoupling Proteins Homo sapiens R-HSA-166187	1/5	0.09054	0.447543	0	0	13.08	31.41771
PTK6 Expression Homo sapiens R-HSA-8849473	1/5	0.09054	0.447543	0	0	13.08	31.41771
Uptake and actions of bacterial toxins Homo sapiens R-HSA-5339562	2/27	0.09096	0.447543	0	0	4.192299	10.05033
Constitutive Signaling by NOTCH1 HD+PEST Domain Mutants Homo sapiens R-HSA-2894862	3/57	0.091919	0.447543	0	0	2.914805	6.95718
Signaling by NOTCH1 in Cancer Homo sapiens R-HSA-2644603	3/57	0.091919	0.447543	0	0	2.914805	6.95718
Signaling by NOTCH1 PEST Domain Mutants in Cancer	3/57	0.091919	0.447543	0	0	2.914805	6.95718

Homo sapiens R-HSA-2644602							
Constitutive Signaling by NOTCH1 PEST Domain Mutants Homo sapiens R-HSA-2644606	3/57	0.091919	0.447543	0	0	2.914805	6.95718
Signaling by NOTCH1 HD+PEST Domain Mutants in Cancer Homo sapiens R-HSA-2894858	3/57	0.091919	0.447543	0	0	2.914805	6.95718
Oxidative Stress Induced Senescence Homo sapiens R-HSA-2559580	4/91	0.092509	0.447543	0	0	2.414658	5.747983
Insulin receptor signalling cascade Homo sapiens R-HSA-74751	9/287	0.093471	0.447543	0	0	1.706565	4.044737
Diseases of signal transduction Homo sapiens R-HSA-5663202	9/288	0.094985	0.447543	0	0	1.70036	4.002713
IGF1R signaling cascade Homo sapiens R-HSA-2428924	9/288	0.094985	0.447543	0	0	1.70036	4.002713
Signaling by Type 1 Insulin-like Growth Factor 1 Receptor (IGF1R) Homo sapiens R-HSA-2404192	9/288	0.094985	0.447543	0	0	1.70036	4.002713
IRS-related events triggered by IGF1R Homo sapiens R-HSA-2428928	9/288	0.094985	0.447543	0	0	1.70036	4.002713
MAPK6/MAPK4 signaling Homo sapiens R-HSA-5687128	4/92	0.09536	0.447543	0	0	2.387097	5.609908
MyD88:Mal cascade initiated on plasma membrane Homo sapiens R-HSA-166058	4/92	0.09536	0.447543	0	0	2.387097	5.609908
Toll Like Receptor TLR1:TLR2 Cascade Homo sapiens R-HSA-168179	4/92	0.09536	0.447543	0	0	2.387097	5.609908
Toll Like Receptor TLR6:TLR2 Cascade Homo sapiens R-HSA-168188	4/92	0.09536	0.447543	0	0	2.387097	5.609908
Toll Like Receptor 2 (TLR2) Cascade Homo sapiens R-HSA-181438	4/92	0.09536	0.447543	0	0	2.387097	5.609908
NGF signalling via TRKA from the plasma membrane Homo sapiens R-HSA-187037	11/374	0.096705	0.447543	0	0	1.599087	3.735612
PERK regulates gene expression Homo sapiens R-HSA-381042	2/28	0.096785	0.447543	0	0	4.030852	9.413117
Endosomal Sorting Complex Required For Transport	2/28	0.096785	0.447543	0	0	4.030852	9.413117

(ESCRT) Homo sapiens R-HSA-917729							
The role of GTSE1 in G2/M progression after G2 checkpoint Homo sapiens R-HSA-8852276	3/59	0.099441	0.456587	0	0	2.810417	6.486985
Negative regulation of FGFR3 signaling Homo sapiens R-HSA-5654732	2/29	0.102711	0.468306	0	0	3.881363	8.833332
Vitamins Homo sapiens R-HSA-211916	1/6	0.107642	0.484018	0	0	10.46347	23.32249
Heme degradation Homo sapiens R-HSA-189483	1/6	0.107642	0.484018	0	0	10.46347	23.32249
Negative regulation of FGFR4 signaling Homo sapiens R-HSA-5654733	2/31	0.114849	0.512889	0	0	3.613314	7.819695
Gastrin-CREB signalling pathway via PKC and MAPK Homo sapiens R-HSA-881907	12/432	0.116453	0.516513	0	0	1.507378	3.241267
Regulation of beta-cell development Homo sapiens R-HSA-186712	2/32	0.121049	0.529692	0	0	3.492692	7.375018
Negative regulation of FGFR1 signaling Homo sapiens R-HSA-5654726	2/32	0.121049	0.529692	0	0	3.492692	7.375018
Inositol transporters Homo sapiens R-HSA-429593	1/7	0.124423	0.53371	0	0	8.719111	18.1712
Vitamin D (calciferol) metabolism Homo sapiens R-HSA-196791	1/7	0.124423	0.53371	0	0	8.719111	18.1712
PTK6 promotes HIF1A stabilization Homo sapiens R-HSA-8857538	1/7	0.124423	0.53371	0	0	8.719111	18.1712
Semaphorin interactions Homo sapiens R-HSA-373755	3/67	0.131802	0.546787	0	0	2.45811	4.981257
Signaling by Insulin receptor Homo sapiens R-HSA-74752	9/311	0.133679	0.546787	0	0	1.568995	3.157307
Negative regulation of FGFR2 signaling Homo sapiens R-HSA-5654727	2/34	0.133685	0.546787	0	0	3.274064	6.58831
Interferon alpha/beta signaling Homo sapiens R-HSA-909733	3/68	0.13608	0.546787	0	0	2.420169	4.827061
Lysosome Vesicle Biogenesis Homo sapiens R-HSA-432720	2/35	0.140109	0.546787	0	0	3.174688	6.239313
Fanconi Anemia Pathway Homo sapiens R-HSA-6783310	2/35	0.140109	0.546787	0	0	3.174688	6.239313
VEGF ligand-receptor interactions Homo sapiens R-HSA-194313	1/8	0.14089	0.546787	0	0	7.473143	14.64569

VEGF binds to VEGFR leading to receptor dimerization Homo sapiens R-HSA-195399	1/8	0.14089	0.546787	0	0	7.473143	14.64569
Erythrocytes take up oxygen and release carbon dioxide Homo sapiens R-HSA-1247673	1/8	0.14089	0.546787	0	0	7.473143	14.64569
Sema4D mediated inhibition of cell attachment and migration Homo sapiens R-HSA-416550	1/8	0.14089	0.546787	0	0	7.473143	14.64569
Terminal pathway of complement Homo sapiens R-HSA-166665	1/8	0.14089	0.546787	0	0	7.473143	14.64569
Release of Hh-Np from the secreting cell Homo sapiens R-HSA-5362798	1/8	0.14089	0.546787	0	0	7.473143	14.64569
Ligand-receptor interactions Homo sapiens R-HSA-5632681	1/8	0.14089	0.546787	0	0	7.473143	14.64569
WNT mediated activation of DVL Homo sapiens R-HSA-201688	1/8	0.14089	0.546787	0	0	7.473143	14.64569
BH3-only proteins associate with and inactivate anti-apoptotic BCL-2 members Homo sapiens R-HSA-111453	1/8	0.14089	0.546787	0	0	7.473143	14.64569
Downregulation of ERBB4 signaling Homo sapiens R-HSA-1253288	1/8	0.14089	0.546787	0	0	7.473143	14.64569
NCAM1 interactions Homo sapiens R-HSA-419037	2/37	0.15315	0.572245	0	0	2.992972	5.615825
Signaling by NOTCH1 Homo sapiens R-HSA-1980143	3/72	0.15365	0.572245	0	0	2.279403	4.269494
Downstream signaling events of B Cell Receptor (BCR) Homo sapiens R-HSA-1168372	6/192	0.154428	0.572245	0	0	1.694682	3.16571
CHL1 interactions Homo sapiens R-HSA-447041	1/9	0.157048	0.572245	0	0	6.538667	12.10442
Ca2+ activated K+ channels Homo sapiens R-HSA-1296052	1/9	0.157048	0.572245	0	0	6.538667	12.10442
Prostanoid ligand receptors Homo sapiens R-HSA-391908	1/9	0.157048	0.572245	0	0	6.538667	12.10442
AKT phosphorylates targets in the nucleus Homo sapiens R-HSA-198693	1/9	0.157048	0.572245	0	0	6.538667	12.10442
Highly calcium permeable nicotinic acetylcholine receptors Homo sapiens R-HSA-629597	1/9	0.157048	0.572245	0	0	6.538667	12.10442

PTK6 Regulates RTKs and Their Effectors AKT1 and DOK1 Homo sapiens R-HSA-8849469	1/9	0.157048	0.572245	0	0	6.538667	12.10442
EGFR Transactivation by Gastrin Homo sapiens R-HSA-2179392	1/9	0.157048	0.572245	0	0	6.538667	12.10442
Activated TLR4 signalling Homo sapiens R-HSA-166054	4/112	0.160306	0.572245	0	0	1.943051	3.557081
Interferon Signaling Homo sapiens R-HSA-913531	6/196	0.165023	0.572245	0	0	1.658663	2.988368
Collagen degradation Homo sapiens R-HSA-1442490	2/39	0.166414	0.572245	0	0	2.8309	5.076589
Activation of NMDA receptor upon glutamate binding and postsynaptic events Homo sapiens R-HSA-442755	2/39	0.166414	0.572245	0	0	2.8309	5.076589
Abacavir transport and metabolism Homo sapiens R-HSA-2161522	1/10	0.172902	0.572245	0	0	5.811852	10.19997
eNOS activation Homo sapiens R-HSA-203615	1/10	0.172902	0.572245	0	0	5.811852	10.19997
Tetrahydrobiopterin (BH4) synthesis. recycling. salvage and regulation Homo sapiens R-HSA-1474151	1/10	0.172902	0.572245	0	0	5.811852	10.19997
ATF6-alpha activates chaperone genes Homo sapiens R-HSA-381183	1/10	0.172902	0.572245	0	0	5.811852	10.19997
Regulation of signaling by NODAL Homo sapiens R-HSA-1433617	1/10	0.172902	0.572245	0	0	5.811852	10.19997
SLBP independent Processing of Histone Pre-mRNAs Homo sapiens R-HSA-111367	1/10	0.172902	0.572245	0	0	5.811852	10.19997
IRAK2 mediated activation of TAK1 complex upon TLR7/8 or 9 stimulation Homo sapiens R-HSA-975163	1/10	0.172902	0.572245	0	0	5.811852	10.19997
IRAK2 mediated activation of TAK1 complex Homo sapiens R-HSA-937042	1/10	0.172902	0.572245	0	0	5.811852	10.19997
Diseases of carbohydrate metabolism Homo sapiens R-HSA-5663084	1/10	0.172902	0.572245	0	0	5.811852	10.19997
Myoclonic epilepsy of Lafora Homo sapiens R-HSA-3785653	1/10	0.172902	0.572245	0	0	5.811852	10.19997
Glycogen storage diseases Homo sapiens R-HSA-3229121	1/10	0.172902	0.572245	0	0	5.811852	10.19997

Signaling by FGFR3 fusions in cancer Homo sapiens R-HSA-8853334	1/10	0.172902	0.572245	0	0	5.811852	10.19997
Activation of the AP-1 family of transcription factors Homo sapiens R-HSA-450341	1/10	0.172902	0.572245	0	0	5.811852	10.19997
IRAK1 recruits IKK complex Homo sapiens R-HSA-937039	1/10	0.172902	0.572245	0	0	5.811852	10.19997
IRAK1 recruits IKK complex upon TLR7/8 or 9 stimulation Homo sapiens R-HSA-975144	1/10	0.172902	0.572245	0	0	5.811852	10.19997
Glucose metabolism Homo sapiens R-HSA-70326	3/79	0.185945	0.598327	0	0	2.068717	3.480208
EPHB-mediated forward signaling Homo sapiens R-HSA-3928662	2/42	0.186654	0.598327	0	0	2.618182	4.394618
SLBP Dependent Processing of Replication-Dependent Histone Pre-mRNAs Homo sapiens R-HSA-77588	1/11	0.188459	0.598327	0	0	5.2304	8.728872
Highly calcium permeable postsynaptic nicotinic acetylcholine receptors Homo sapiens R-HSA-629594	1/11	0.188459	0.598327	0	0	5.2304	8.728872
Response to metal ions Homo sapiens R-HSA-5660526	1/11	0.188459	0.598327	0	0	5.2304	8.728872
Metallothioneins bind metals Homo sapiens R-HSA-5661231	1/11	0.188459	0.598327	0	0	5.2304	8.728872
Interleukin-6 signaling Homo sapiens R-HSA-1059683	1/11	0.188459	0.598327	0	0	5.2304	8.728872
Signaling by FGFR4 in disease Homo sapiens R-HSA-5655291	1/11	0.188459	0.598327	0	0	5.2304	8.728872
TP53 Regulates Transcription of Cell Death Genes Homo sapiens R-HSA-5633008	2/43	0.193476	0.598327	0	0	2.554193	4.195522
PI-3K cascade:FGFR1 Homo sapiens R-HSA-5654689	4/122	0.197525	0.598327	0	0	1.777474	2.882869
PI-3K cascade:FGFR3 Homo sapiens R-HSA-5654710	4/122	0.197525	0.598327	0	0	1.777474	2.882869
PI3K events in ERBB4 signaling Homo sapiens R-HSA-1250342	4/122	0.197525	0.598327	0	0	1.777474	2.882869
PIP3 activates AKT signaling Homo sapiens R-HSA-1257604	4/122	0.197525	0.598327	0	0	1.777474	2.882869
PI-3K cascade:FGFR4 Homo sapiens R-HSA-5654720	4/122	0.197525	0.598327	0	0	1.777474	2.882869
PI-3K cascade:FGFR2 Homo sapiens R-HSA-5654695	4/122	0.197525	0.598327	0	0	1.777474	2.882869

Toll Like Receptor 4 (TLR4) Cascade Homo sapiens R-HSA-166016	4/122	0.197525	0.598327	0	0	1.777474	2.882869
Toll Like Receptor 10 (TLR10) Cascade Homo sapiens R-HSA-168142	3/82	0.200292	0.598327	0	0	1.989853	3.199638
Toll Like Receptor 5 (TLR5) Cascade Homo sapiens R-HSA-168176	3/82	0.200292	0.598327	0	0	1.989853	3.199638
MyD88 cascade initiated on plasma membrane Homo sapiens R-HSA-975871	3/82	0.200292	0.598327	0	0	1.989853	3.199638
Interleukin-1 signaling Homo sapiens R-HSA-446652	2/44	0.20033	0.598327	0	0	2.493252	4.008625
Erythrocytes take up carbon dioxide and release oxygen Homo sapiens R-HSA-1237044	1/12	0.203725	0.598327	0	0	4.754667	7.564606
O2/CO2 exchange in erythrocytes Homo sapiens R-HSA-1480926	1/12	0.203725	0.598327	0	0	4.754667	7.564606
ATF6-alpha activates chaperones Homo sapiens R-HSA-381033	1/12	0.203725	0.598327	0	0	4.754667	7.564606
The NLRP3 inflammasome Homo sapiens R-HSA-844456	1/12	0.203725	0.598327	0	0	4.754667	7.564606
Presynaptic nicotinic acetylcholine receptors Homo sapiens R-HSA-622323	1/12	0.203725	0.598327	0	0	4.754667	7.564606
TRAF6 mediated induction of NFkB and MAP kinases upon TLR7/8 or 9 activation Homo sapiens R-HSA-975138	3/83	0.205132	0.599759	0	0	1.964879	3.112565
GAB1 signalosome Homo sapiens R-HSA-180292	4/125	0.209171	0.601666	0	0	1.733138	2.711673
PI3K/AKT activation Homo sapiens R-HSA-198203	4/125	0.209171	0.601666	0	0	1.733138	2.711673
TP53 Regulates Metabolic Genes Homo sapiens R-HSA-5628897	3/84	0.209999	0.601666	0	0	1.940522	3.028482
MyD88 dependent cascade initiated on endosome Homo sapiens R-HSA-975155	3/85	0.214891	0.601666	0	0	1.916759	2.947257
Toll Like Receptor 7/8 (TLR7/8) Cascade Homo sapiens R-HSA-168181	3/85	0.214891	0.601666	0	0	1.916759	2.947257
Facilitative Na+-independent glucose transporters Homo sapiens R-HSA-428790	1/13	0.218704	0.601666	0	0	4.358222	6.624662
Synthesis. secretion. and inactivation of Glucose-dependent Insulinotropic	1/13	0.218704	0.601666	0	0	4.358222	6.624662

Polypeptide (GIP) Homo sapiens R-HSA-400511							
TP53 Regulates Transcription of Genes Involved in G1 Cell Cycle Arrest Homo sapiens R-HSA-6804116	1/13	0.218704	0.601666	0	0	4.3582 22	6.624662
Signaling by Activin Homo sapiens R-HSA-1502540	1/13	0.218704	0.601666	0	0	4.3582 22	6.624662
AKT phosphorylates targets in the cytosol Homo sapiens R-HSA-198323	1/13	0.218704	0.601666	0	0	4.3582 22	6.624662
p75NTR recruits signalling complexes Homo sapiens R-HSA-209543	1/13	0.218704	0.601666	0	0	4.3582 22	6.624662
NF-kB is activated and signals survival Homo sapiens R-HSA-209560	1/13	0.218704	0.601666	0	0	4.3582 22	6.624662
ERBB2 Activates PTK6 Signaling Homo sapiens R-HSA-8847993	1/13	0.218704	0.601666	0	0	4.3582 22	6.624662
Downregulation of ERBB2:ERBB3 signaling Homo sapiens R-HSA-1358803	1/13	0.218704	0.601666	0	0	4.3582 22	6.624662
Regulation of PLK1 Activity at G2/M Transition Homo sapiens R-HSA-2565942	3/87	0.224746	0.607204	0	0	1.8709 31	2.7929
Hexose transport Homo sapiens R-HSA-189200	2/48	0.227991	0.607204	0	0	2.2759 82	3.364927
Signalling by NGF Homo sapiens R-HSA-166520	11/450	0.228658	0.607204	0	0	1.3170 34	1.943321
Toll Like Receptor 9 (TLR9) Cascade Homo sapiens R-HSA-168138	3/88	0.229706	0.607204	0	0	1.8488 25	2.719541
SEMA3A-Plexin repulsion signaling by inhibiting Integrin adhesion Homo sapiens R-HSA-399955	1/14	0.233402	0.607204	0	0	4.0227 69	5.853109
Early Phase of HIV Life Cycle Homo sapiens R-HSA-162594	1/14	0.233402	0.607204	0	0	4.0227 69	5.853109
Regulation of TP53 Activity through Association with Co-factors Homo sapiens R-HSA-6804759	1/14	0.233402	0.607204	0	0	4.0227 69	5.853109
TP53 regulates transcription of several additional cell death genes whose specific roles in p53-dependent apoptosis remain uncertain Homo sapiens R-HSA-6803205	1/14	0.233402	0.607204	0	0	4.0227 69	5.853109

Glutathione synthesis and recycling Homo sapiens R-HSA-174403	1/14	0.233402	0.607204	0	0	4.022769	5.853109
Regulation of pyruvate dehydrogenase (PDH) complex Homo sapiens R-HSA-204174	1/14	0.233402	0.607204	0	0	4.022769	5.853109
Activation of Nicotinic Acetylcholine Receptors Homo sapiens R-HSA-629602	1/14	0.233402	0.607204	0	0	4.022769	5.853109
Postsynaptic nicotinic acetylcholine receptors Homo sapiens R-HSA-622327	1/14	0.233402	0.607204	0	0	4.022769	5.853109
Acetylcholine Binding And Downstream Events Homo sapiens R-HSA-181431	1/14	0.233402	0.607204	0	0	4.022769	5.853109
Activation of anterior HOX genes in hindbrain development during early embryogenesis Homo sapiens R-HSA-5617472	3/89	0.234686	0.607204	0	0	1.827234	2.648585
Activation of HOX genes during differentiation Homo sapiens R-HSA-5619507	3/89	0.234686	0.607204	0	0	1.827234	2.648585
CREB phosphorylation through the activation of CaMKII Homo sapiens R-HSA-442729	1/15	0.247824	0.616722	0	0	3.735238	5.210798
Eicosanoid ligand-binding receptors Homo sapiens R-HSA-391903	1/15	0.247824	0.616722	0	0	3.735238	5.210798
Regulation of innate immune responses to cytosolic DNA Homo sapiens R-HSA-3134975	1/15	0.247824	0.616722	0	0	3.735238	5.210798
Association of licensing factors with the pre-replicative complex Homo sapiens R-HSA-69298	1/15	0.247824	0.616722	0	0	3.735238	5.210798
NRIF signals cell death from the nucleus Homo sapiens R-HSA-205043	1/15	0.247824	0.616722	0	0	3.735238	5.210798
Glycogen synthesis Homo sapiens R-HSA-3322077	1/15	0.247824	0.616722	0	0	3.735238	5.210798
Glycogen breakdown (glycogenolysis) Homo sapiens R-HSA-70221	1/15	0.247824	0.616722	0	0	3.735238	5.210798
ERBB2 Regulates Cell Motility Homo sapiens R-HSA-6785631	1/15	0.247824	0.616722	0	0	3.735238	5.210798

Signaling by EGFRvIII in Cancer Homo sapiens R-HSA-5637812	1/15	0.247824	0.616722	0	0	3.735238	5.210798
Constitutive Signaling by EGFRvIII Homo sapiens R-HSA-5637810	1/15	0.247824	0.616722	0	0	3.735238	5.210798
Metabolism of fat-soluble vitamins Homo sapiens R-HSA-6806667	2/51	0.248904	0.617054	0	0	2.136309	2.970942
Mitotic G1-G1/S phases Homo sapiens R-HSA-453279	4/136	0.253366	0.618869	0	0	1.587814	2.179942
Interferon gamma signaling Homo sapiens R-HSA-877300	3/93	0.254787	0.618869	0	0	1.745666	2.386898
Innate Immune System Homo sapiens R-HSA-168249	18/807	0.260976	0.618869	0	0	1.200268	1.612354
Regulation of KIT signaling Homo sapiens R-HSA-1433559	1/16	0.261975	0.618869	0	0	3.486044	4.669576
Sema3A PAK dependent Axon repulsion Homo sapiens R-HSA-399954	1/16	0.261975	0.618869	0	0	3.486044	4.669576
TGF-beta receptor signaling in EMT (epithelial to mesenchymal transition) Homo sapiens R-HSA-2173791	1/16	0.261975	0.618869	0	0	3.486044	4.669576
Translesion synthesis by REV1 Homo sapiens R-HSA-110312	1/16	0.261975	0.618869	0	0	3.486044	4.669576
p75NTR signals via NF-kB Homo sapiens R-HSA-193639	1/16	0.261975	0.618869	0	0	3.486044	4.669576
TRAF6 mediated induction of TAK1 complex Homo sapiens R-HSA-937072	1/16	0.261975	0.618869	0	0	3.486044	4.669576
Spry regulation of FGF signaling Homo sapiens R-HSA-1295596	1/16	0.261975	0.618869	0	0	3.486044	4.669576
PI3K events in ERBB2 signaling Homo sapiens R-HSA-1963642	1/16	0.261975	0.618869	0	0	3.486044	4.669576
GRB2 events in ERBB2 signaling Homo sapiens R-HSA-1963640	1/16	0.261975	0.618869	0	0	3.486044	4.669576
MAP3K8 (TPL2)-dependent MAPK1/3 activation Homo sapiens R-HSA-5684264	1/16	0.261975	0.618869	0	0	3.486044	4.669576
EPH-Ephrin signaling Homo sapiens R-HSA-2682334	3/95	0.264928	0.620212	0	0	1.707542	2.268126
Golgi Associated Vesicle Biogenesis Homo sapiens R-HSA-432722	2/54	0.269872	0.620212	0	0	2.012752	2.636315
Toll-Like Receptors Cascades Homo sapiens R-HSA-168898	4/140	0.269901	0.620212	0	0	1.540797	2.017981

Visual phototransduction Homo sapiens R-HSA-2187338	3/97	0.275116	0.620212	0	0	1.67104	2.15658
Signaling by the B Cell Receptor (BCR) Homo sapiens R-HSA-983705	6/233	0.275147	0.620212	0	0	1.385665	1.78813
Ras activation uopn Ca2+ infux through NMDA receptor Homo sapiens R-HSA-442982	1/17	0.275861	0.620212	0	0	3.268	4.208719
TNF receptor superfamily (TNFSF) members mediating non-canonical NF-kB pathway Homo sapiens R-HSA-5676594	1/17	0.275861	0.620212	0	0	3.268	4.208719
Trafficking of GluR2-containing AMPA receptors Homo sapiens R-HSA-416993	1/17	0.275861	0.620212	0	0	3.268	4.208719
Tristetraprolin (TTP. ZFP36) binds and destabilizes mRNA Homo sapiens R-HSA-450513	1/17	0.275861	0.620212	0	0	3.268	4.208719
Inflammasomes Homo sapiens R-HSA-622312	1/17	0.275861	0.620212	0	0	3.268	4.208719
Formation of Senescence-Associated Heterochromatin Foci (SAHF) Homo sapiens R-HSA-2559584	1/17	0.275861	0.620212	0	0	3.268	4.208719
Metabolism of porphyrins Homo sapiens R-HSA-189445	1/17	0.275861	0.620212	0	0	3.268	4.208719
Translesion synthesis by POLK Homo sapiens R-HSA-5655862	1/17	0.275861	0.620212	0	0	3.268	4.208719
Translesion synthesis by POLI Homo sapiens R-HSA-5656121	1/17	0.275861	0.620212	0	0	3.268	4.208719
Hh mutants that don't undergo autocatalytic processing are degraded by ERAD Homo sapiens R-HSA-5362768	2/55	0.276862	0.620323	0	0	1.974675	2.535948
Neurotransmitter Receptor Binding And Downstream Transmission In The Postsynaptic Cell Homo sapiens R-HSA-112314	4/142	0.27824	0.621276	0	0	1.518311	1.942331
Chemokine receptors bind chemokines Homo sapiens R-HSA-380108	2/56	0.283848	0.631634	0	0	1.938008	2.440567
Activation of SMO Homo sapiens R-HSA-5635838	1/18	0.289487	0.635506	0	0	3.075608	3.812667
TP53 Regulates Transcription of Genes Involved in G2 Cell	1/18	0.289487	0.635506	0	0	3.075608	3.812667

Cycle Arrest Homo sapiens R-HSA-6804114							
SHC1 events in ERBB2 signaling Homo sapiens R-HSA-1250196	1/18	0.289487	0.635506	0	0	3.075608	3.812667
Activation of IRF3/IRF7 mediated by TBK1/IKK epsilon Homo sapiens R-HSA-936964	1/18	0.289487	0.635506	0	0	3.075608	3.812667
Hh mutants abrogate ligand secretion Homo sapiens R-HSA-5387390	2/57	0.290827	0.636306	0	0	1.902674	2.349854
Recruitment and ATM-mediated phosphorylation of repair and signaling proteins at DNA double strand breaks Homo sapiens R-HSA-5693565	2/58	0.297797	0.643048	0	0	1.868602	2.263516
GABA synthesis. release. reuptake and degradation Homo sapiens R-HSA-888590	1/19	0.302856	0.643048	0	0	2.904593	3.469528
Other semaphorin interactions Homo sapiens R-HSA-416700	1/19	0.302856	0.643048	0	0	2.904593	3.469528
Ephrin signaling Homo sapiens R-HSA-3928664	1/19	0.302856	0.643048	0	0	2.904593	3.469528
TP53 Regulates Transcription of Genes Involved in Cytochrome C Release Homo sapiens R-HSA-6803204	1/19	0.302856	0.643048	0	0	2.904593	3.469528
Signaling by NODAL Homo sapiens R-HSA-1181150	1/19	0.302856	0.643048	0	0	2.904593	3.469528
Phase 4 - resting membrane potential Homo sapiens R-HSA-5576886	1/19	0.302856	0.643048	0	0	2.904593	3.469528
Translesion Synthesis by POLH Homo sapiens R-HSA-110320	1/19	0.302856	0.643048	0	0	2.904593	3.469528
DNA Damage/Telomere Stress Induced Senescence Homo sapiens R-HSA-2559586	2/59	0.304757	0.643048	0	0	1.835726	2.181282
DNA Double Strand Break Response Homo sapiens R-HSA-5693606	2/59	0.304757	0.643048	0	0	1.835726	2.181282
SCF(Skp2)-mediated degradation of p27/p21 Homo sapiens R-HSA-187577	2/59	0.304757	0.643048	0	0	1.835726	2.181282
MAP kinase activation in TLR cascade Homo sapiens R-HSA-450294	2/60	0.311704	0.655585	0	0	1.803983	2.102903

eNOS activation and regulation Homo sapiens R-HSA-203765	1/20	0.315975	0.658619	0	0	2.751579	3.170073
Metabolism of nitric oxide Homo sapiens R-HSA-202131	1/20	0.315975	0.658619	0	0	2.751579	3.170073
DNA Replication Homo sapiens R-HSA-69306	3/105	0.316178	0.658619	0	0	1.539347	1.772484
Constitutive Signaling by Aberrant PI3K in Cancer Homo sapiens R-HSA-2219530	2/61	0.318637	0.661628	0	0	1.773316	2.028148
Biological oxidations Homo sapiens R-HSA-211859	5/199	0.319989	0.662326	0	0	1.349793	1.538047
Degradation of the extracellular matrix Homo sapiens R-HSA-1474228	3/106	0.321328	0.662992	0	0	1.524324	1.730556
HATs acetylate histones Homo sapiens R-HSA-3214847	3/107	0.326478	0.671075	0	0	1.50959	1.689822
Regulation of FZD by ubiquitination Homo sapiens R-HSA-4641263	1/21	0.328847	0.671075	0	0	2.613867	2.907041
Collagen biosynthesis and modifying enzymes Homo sapiens R-HSA-1650814	2/63	0.33245	0.671075	0	0	1.715	1.888672
Circadian Clock Homo sapiens R-HSA-400253	2/63	0.33245	0.671075	0	0	1.715	1.888672
p53-Dependent G1/S DNA damage checkpoint Homo sapiens R-HSA-69580	2/63	0.33245	0.671075	0	0	1.715	1.888672
p53-Dependent G1 DNA Damage Response Homo sapiens R-HSA-69563	2/63	0.33245	0.671075	0	0	1.715	1.888672
Asymmetric localization of PCP proteins Homo sapiens R-HSA-4608870	2/63	0.33245	0.671075	0	0	1.715	1.888672
Hedgehog ligand biogenesis Homo sapiens R-HSA-5358346	2/64	0.339327	0.678792	0	0	1.687252	1.823569
Pink/Parkin Mediated Mitophagy Homo sapiens R-HSA-5205685	1/22	0.341478	0.678792	0	0	2.48927	2.674648
Mitophagy Homo sapiens R-HSA-5205647	1/22	0.341478	0.678792	0	0	2.48927	2.674648
Signaling by FGFR3 point mutants in cancer Homo sapiens R-HSA-8853338	1/22	0.341478	0.678792	0	0	2.48927	2.674648
Signaling by FGFR3 in disease Homo sapiens R-HSA-5655332	1/22	0.341478	0.678792	0	0	2.48927	2.674648

G1/S DNA Damage Checkpoints Homo sapiens R-HSA-69615	2/65	0.346182	0.680604	0	0	1.660385	1.761323
G1/S Transition Homo sapiens R-HSA-69206	3/112	0.35221	0.680604	0	0	1.439973	1.502654
Activation of NF-kappaB in B cells Homo sapiens R-HSA-1169091	2/66	0.353013	0.680604	0	0	1.634358	1.701775
The canonical retinoid cycle in rods (twilight vision) Homo sapiens R-HSA-2453902	1/23	0.353872	0.680604	0	0	2.376	2.468235
Incretin synthesis. secretion. and inactivation Homo sapiens R-HSA-400508	1/23	0.353872	0.680604	0	0	2.376	2.468235
Deadenylation of mRNA Homo sapiens R-HSA-429947	1/23	0.353872	0.680604	0	0	2.376	2.468235
Branched-chain amino acid catabolism Homo sapiens R-HSA-70895	1/23	0.353872	0.680604	0	0	2.376	2.468235
Gap-filling DNA repair synthesis and ligation in GG-NER Homo sapiens R-HSA-5696397	1/23	0.353872	0.680604	0	0	2.376	2.468235
Downregulation of SMAD2/3:SMAD4 transcriptional activity Homo sapiens R-HSA-2173795	1/23	0.353872	0.680604	0	0	2.376	2.468235
SHC-mediated cascade:FGFR4 Homo sapiens R-HSA-5654719	1/23	0.353872	0.680604	0	0	2.376	2.468235
APC/C:Cdc20 mediated degradation of Cyclin B Homo sapiens R-HSA-174048	1/23	0.353872	0.680604	0	0	2.376	2.468235
PPARA activates gene expression Homo sapiens R-HSA-1989781	3/113	0.357345	0.684554	0	0	1.42681	1.468263
Role of LAT2/NTAL/LAB on calcium mobilization Homo sapiens R-HSA-2730905	4/162	0.363096	0.684554	0	0	1.324758	1.342095
Transmission across Chemical Synapses Homo sapiens R-HSA-112315	5/211	0.364552	0.684554	0	0	1.270379	1.281922
Sema4D induced cell migration and growth-cone collapse Homo sapiens R-HSA-416572	1/24	0.366033	0.684554	0	0	2.27258	2.284013
TNFs bind their physiological receptors Homo sapiens R-HSA-5669034	1/24	0.366033	0.684554	0	0	2.27258	2.284013
Post-Elongation Processing of Intronless pre-mRNA Homo sapiens R-HSA-112297	1/24	0.366033	0.684554	0	0	2.27258	2.284013

Processing of Capped Intronless Pre-mRNA Homo sapiens R-HSA-75067	1/24	0.366033	0.684554	0	0	2.27258	2.284013
IKK complex recruitment mediated by RIP1 Homo sapiens R-HSA-937041	1/24	0.366033	0.684554	0	0	2.27258	2.284013
Amyloid fiber formation Homo sapiens R-HSA-977225	2/68	0.3666	0.684554	0	0	1.58467	1.590192
Cyclin E associated events during G1/S transition Homo sapiens R-HSA-69202	2/68	0.3666	0.684554	0	0	1.58467	1.590192
Regulation of lipid metabolism by Peroxisome proliferator-activated receptor alpha (PPARalpha) Homo sapiens R-HSA-400206	3/116	0.372715	0.684554	0	0	1.388716	1.37058
Cyclin A:Cdk2-associated events at S phase entry Homo sapiens R-HSA-69656	2/69	0.373352	0.684554	0	0	1.560939	1.53789
Loss of Nlp from mitotic centrosomes Homo sapiens R-HSA-380259	2/69	0.373352	0.684554	0	0	1.560939	1.53789
Loss of proteins required for interphase microtubule organization?from the centrosome Homo sapiens R-HSA-380284	2/69	0.373352	0.684554	0	0	1.560939	1.53789
Activation of G protein gated Potassium channels Homo sapiens R-HSA-1296041	1/25	0.377966	0.684554	0	0	2.177778	2.11887
Inhibition of voltage gated Ca2+ channels via Gbeta/gamma subunits Homo sapiens R-HSA-997272	1/25	0.377966	0.684554	0	0	2.177778	2.11887
G protein gated Potassium channels Homo sapiens R-HSA-1296059	1/25	0.377966	0.684554	0	0	2.177778	2.11887
HDMs demethylate histones Homo sapiens R-HSA-3214842	1/25	0.377966	0.684554	0	0	2.177778	2.11887
APC-Cdc20 mediated degradation of Nek2A Homo sapiens R-HSA-179409	1/25	0.377966	0.684554	0	0	2.177778	2.11887
trans-Golgi Network Vesicle Budding Homo sapiens R-HSA-199992	2/70	0.380075	0.684554	0	0	1.537905	1.487751
Clathrin derived vesicle budding Homo sapiens R-HSA-421837	2/70	0.380075	0.684554	0	0	1.537905	1.487751
Switching of origins to a post-replicative state Homo sapiens R-HSA-69052	2/70	0.380075	0.684554	0	0	1.537905	1.487751

Orc1 removal from chromatin Homo sapiens R-HSA-68949	2/70	0.380075	0.684554	0	0	1.537905	1.487751
SHC-mediated cascade:FGFR2 Homo sapiens R-HSA-5654699	1/26	0.389675	0.697051	0	0	2.09056	1.970232
ISG15 antiviral mechanism Homo sapiens R-HSA-1169408	2/72	0.393427	0.697051	0	0	1.493812	1.393516
Antiviral mechanism by IFN-stimulated genes Homo sapiens R-HSA-1169410	2/72	0.393427	0.697051	0	0	1.493812	1.393516
Removal of licensing factors from origins Homo sapiens R-HSA-69300	2/72	0.393427	0.697051	0	0	1.493812	1.393516
TRAF6 Mediated Induction of proinflammatory cytokines Homo sapiens R-HSA-168180	2/72	0.393427	0.697051	0	0	1.493812	1.393516
AURKA Activation by TPX2 Homo sapiens R-HSA-8854518	2/72	0.393427	0.697051	0	0	1.493812	1.393516
Sema4D in semaphorin signaling Homo sapiens R-HSA-400685	1/27	0.401164	0.699356	0	0	2.010051	1.83595
CREB phosphorylation through the activation of Ras Homo sapiens R-HSA-442742	1/27	0.401164	0.699356	0	0	2.010051	1.83595
EGFR downregulation Homo sapiens R-HSA-182971	1/27	0.401164	0.699356	0	0	2.010051	1.83595
VEGFR2 mediated vascular permeability Homo sapiens R-HSA-5218920	1/27	0.401164	0.699356	0	0	2.010051	1.83595
Pyruvate metabolism Homo sapiens R-HSA-70268	1/27	0.401164	0.699356	0	0	2.010051	1.83595
Interleukin-6 family signaling Homo sapiens R-HSA-6783589	1/27	0.401164	0.699356	0	0	2.010051	1.83595
Signaling by NOTCH Homo sapiens R-HSA-157118	3/122	0.403223	0.701071	0	0	1.318291	1.197358
Binding and Uptake of Ligands by Scavenger Receptors Homo sapiens R-HSA-2173782	2/74	0.406648	0.705145	0	0	1.452169	1.306671
G2/M Transition Homo sapiens R-HSA-69275	4/173	0.409878	0.70886	0	0	1.237832	1.104018
Recognition of DNA damage by PCNA-containing replication complex Homo sapiens R-HSA-110314	1/28	0.412437	0.710846	0	0	1.935506	1.714221
Regulation of DNA replication Homo sapiens R-HSA-69304	2/75	0.413207	0.710846	0	0	1.432203	1.265792
Mitotic G2-G2/M phases Homo sapiens R-HSA-453274	4/175	0.418321	0.717751	0	0	1.223228	1.066051

Surfactant metabolism Homo sapiens R-HSA-5683826	1/29	0.423499	0.719066	0	0	1.866286	1.60352
Activation of BH3-only proteins Homo sapiens R-HSA-114452	1/29	0.423499	0.719066	0	0	1.866286	1.60352
YAP1- and WWTR1 (TAZ)-stimulated gene expression Homo sapiens R-HSA-2032785	1/29	0.423499	0.719066	0	0	1.866286	1.60352
PIWI-interacting RNA (piRNA) biogenesis Homo sapiens R-HSA-5601884	1/29	0.423499	0.719066	0	0	1.866286	1.60352
Tight junction interactions Homo sapiens R-HSA-420029	1/30	0.434353	0.725355	0	0	1.801839	1.50255
RNA Polymerase I Promoter Opening Homo sapiens R-HSA-73728	1/30	0.434353	0.725355	0	0	1.801839	1.50255
Termination of translesion DNA synthesis Homo sapiens R-HSA-5656169	1/30	0.434353	0.725355	0	0	1.801839	1.50255
Activation of the pre-replicative complex Homo sapiens R-HSA-68962	1/30	0.434353	0.725355	0	0	1.801839	1.50255
MAPK targets/ Nuclear events mediated by MAP kinases Homo sapiens R-HSA-450282	1/30	0.434353	0.725355	0	0	1.801839	1.50255
Metabolism of carbohydrates Homo sapiens R-HSA-71387	6/282	0.43765	0.725355	0	0	1.13678	0.939362
RNA Polymerase I Transcription Homo sapiens R-HSA-73864	2/79	0.439069	0.725355	0	0	1.357525	1.117377
Centrosome maturation Homo sapiens R-HSA-380287	2/79	0.439069	0.725355	0	0	1.357525	1.117377
Recruitment of mitotic centrosome proteins and complexes Homo sapiens R-HSA-380270	2/79	0.439069	0.725355	0	0	1.357525	1.117377
HS-GAG biosynthesis Homo sapiens R-HSA-2022928	1/31	0.445003	0.725355	0	0	1.741689	1.4102
Inwardly rectifying K+ channels Homo sapiens R-HSA-1296065	1/31	0.445003	0.725355	0	0	1.741689	1.4102
Trafficking of AMPA receptors Homo sapiens R-HSA-399719	1/31	0.445003	0.725355	0	0	1.741689	1.4102
Glutamate Binding. Activation of AMPA Receptors and Synaptic Plasticity Homo sapiens R-HSA-399721	1/31	0.445003	0.725355	0	0	1.741689	1.4102
Export of Viral Ribonucleoproteins from Nucleus Homo sapiens R-HSA-168274	1/31	0.445003	0.725355	0	0	1.741689	1.4102

Packaging Of Telomere Ends Homo sapiens R-HSA-171306	1/31	0.445003	0.725355	0	0	1.7416 89	1.4102
Regulation of TNFR1 signaling Homo sapiens R-HSA-5357905	1/31	0.445003	0.725355	0	0	1.7416 89	1.4102
Processing of DNA double-strand break ends Homo sapiens R-HSA-5693607	2/81	0.451766	0.733223	0	0	1.3230 22	1.051262
Activation of Matrix Metalloproteinases Homo sapiens R-HSA-1592389	1/32	0.455453	0.733223	0	0	1.6854 19	1.325519
DNA methylation Homo sapiens R-HSA-5334118	1/32	0.455453	0.733223	0	0	1.6854 19	1.325519
RNA Polymerase I Transcription Termination Homo sapiens R-HSA-73863	1/32	0.455453	0.733223	0	0	1.6854 19	1.325519
Gluconeogenesis Homo sapiens R-HSA-70263	1/32	0.455453	0.733223	0	0	1.6854 19	1.325519
DNA Replication Pre-Initiation Homo sapiens R-HSA-69002	2/82	0.458052	0.733784	0	0	1.3064 17	1.020013
M/G1 Transition Homo sapiens R-HSA-68874	2/82	0.458052	0.733784	0	0	1.3064 17	1.020013
PI5P. PP2A and IER3 Regulate PI3K/AKT Signaling Homo sapiens R-HSA-6811558	2/83	0.464297	0.741965	0	0	1.2902 22	0.989897
Inactivation. recovery and regulation of the phototransduction cascade Homo sapiens R-HSA-2514859	1/33	0.465707	0.742399	0	0	1.6326 67	1.247681
Class B/2 (Secretin family receptors) Homo sapiens R-HSA-373080	2/84	0.4705	0.747071	0	0	1.2744 23	0.960864
EPHA-mediated growth cone collapse Homo sapiens R-HSA-3928663	1/34	0.475768	0.747071	0	0	1.5831 11	1.175973
The phototransduction cascade Homo sapiens R-HSA-2514856	1/34	0.475768	0.747071	0	0	1.5831 11	1.175973
Activated PKN1 stimulates transcription of AR (androgen receptor) regulated genes KLK2 and KLK3 Homo sapiens R-HSA-5625886	1/34	0.475768	0.747071	0	0	1.5831 11	1.175973
Negative regulators of RIG-I/MDA5 signaling Homo sapiens R-HSA-936440	1/34	0.475768	0.747071	0	0	1.5831 11	1.175973
Collagen formation Homo sapiens R-HSA-1474290	2/85	0.476659	0.747071	0	0	1.2590 04	0.932864
Hedgehog 'on' state Homo sapiens R-HSA-5632684	2/85	0.476659	0.747071	0	0	1.2590 04	0.932864

Infectious disease Homo sapiens R-HSA-5663205	7/348	0.481676	0.752109	0	0	1.072734	0.783615
Unfolded Protein Response (UPR) Homo sapiens R-HSA-381119	2/86	0.482775	0.752109	0	0	1.243952	0.905853
Post NMDA receptor activation events Homo sapiens R-HSA-438064	1/35	0.485641	0.752109	0	0	1.536471	1.109772
SIRT1 negatively regulates rRNA Expression Homo sapiens R-HSA-427359	1/35	0.485641	0.752109	0	0	1.536471	1.109772
Regulation of TP53 Degradation Homo sapiens R-HSA-6804757	1/35	0.485641	0.752109	0	0	1.536471	1.109772
Regulation of TP53 Expression and Degradation Homo sapiens R-HSA-6806003	1/36	0.495328	0.763237	0	0	1.492495	1.048532
Factors involved in megakaryocyte development and platelet production Homo sapiens R-HSA-983231	3/141	0.496338	0.763237	0	0	1.13568	0.795541
HIV Life Cycle Homo sapiens R-HSA-162587	3/141	0.496338	0.763237	0	0	1.13568	0.795541
Neuronal System Homo sapiens R-HSA-112316	6/301	0.499965	0.767004	0	0	1.062519	0.736558
Steroid hormones Homo sapiens R-HSA-209943	1/37	0.504833	0.770845	0	0	1.450963	0.991775
Translesion synthesis by Y family DNA polymerases bypasses lesions on DNA template Homo sapiens R-HSA-110313	1/37	0.504833	0.770845	0	0	1.450963	0.991775
Negative regulation of the PI3K/AKT network Homo sapiens R-HSA-199418	2/90	0.506789	0.772025	0	0	1.187166	0.806869
PCP/CE pathway Homo sapiens R-HSA-4086400	2/91	0.512679	0.775999	0	0	1.173767	0.7842
Glutathione conjugation Homo sapiens R-HSA-156590	1/38	0.514159	0.775999	0	0	1.411676	0.939079
DNA Damage Recognition in GG-NER Homo sapiens R-HSA-5696394	1/38	0.514159	0.775999	0	0	1.411676	0.939079
Signaling by FGFR1 in disease Homo sapiens R-HSA-5655302	1/38	0.514159	0.775999	0	0	1.411676	0.939079
Phase 1 - Functionalization of compounds Homo sapiens R-HSA-211945	2/92	0.518522	0.776825	0	0	1.160665	0.762294
GABA B receptor activation Homo sapiens R-HSA-977444	1/39	0.52331	0.776825	0	0	1.374456	0.890072

Activation of GABAB receptors Homo sapiens R-HSA-991365	1/39	0.52331	0.776825	0	0	1.374456	0.890072
NS1 Mediated Effects on Host Pathways Homo sapiens R-HSA-168276	1/39	0.52331	0.776825	0	0	1.374456	0.890072
tRNA modification in the nucleus and cytosol Homo sapiens R-HSA-6782315	1/39	0.52331	0.776825	0	0	1.374456	0.890072
Dual Incision in GG-NER Homo sapiens R-HSA-5696400	1/39	0.52331	0.776825	0	0	1.374456	0.890072
Nuclear signaling by ERBB4 Homo sapiens R-HSA-1251985	1/39	0.52331	0.776825	0	0	1.374456	0.890072
Influenza Infection Homo sapiens R-HSA-168254	3/147	0.524238	0.776825	0	0	1.088025	0.702658
PRC2 methylates histones and DNA Homo sapiens R-HSA-212300	1/40	0.532289	0.784319	0	0	1.339145	0.844422
TNFR2 non-canonical NF-kB pathway Homo sapiens R-HSA-5668541	2/95	0.53577	0.784319	0	0	1.123052	0.700842
Host Interactions with Influenza Factors Homo sapiens R-HSA-168253	1/41	0.5411	0.784319	0	0	1.3056	0.801836
Condensation of Prophase Chromosomes Homo sapiens R-HSA-2299718	1/41	0.5411	0.784319	0	0	1.3056	0.801836
TNF signaling Homo sapiens R-HSA-75893	1/41	0.5411	0.784319	0	0	1.3056	0.801836
Regulation of TP53 Activity Homo sapiens R-HSA-5633007	3/151	0.542359	0.784319	0	0	1.058402	0.647558
Synthesis of DNA Homo sapiens R-HSA-69239	2/97	0.54703	0.784319	0	0	1.099296	0.663152
MyD88-independent TLR3/TLR4 cascade Homo sapiens R-HSA-166166	2/97	0.54703	0.784319	0	0	1.099296	0.663152
Toll Like Receptor 3 (TLR3) Cascade Homo sapiens R-HSA-168164	2/97	0.54703	0.784319	0	0	1.099296	0.663152
TRIF-mediated TLR3/TLR4 signaling Homo sapiens R-HSA-937061	2/97	0.54703	0.784319	0	0	1.099296	0.663152
Anchoring of the basal body to the plasma membrane Homo sapiens R-HSA-5620912	2/97	0.54703	0.784319	0	0	1.099296	0.663152
Signaling by Retinoic Acid Homo sapiens R-HSA-5362517	1/42	0.549745	0.784319	0	0	1.273691	0.762051

Retinoid metabolism and transport Homo sapiens R-HSA-975634	1/42	0.549745	0.784319	0	0	1.273691	0.762051
Intrinsic Pathway for Apoptosis Homo sapiens R-HSA-109606	1/42	0.549745	0.784319	0	0	1.273691	0.762051
Glucose transport Homo sapiens R-HSA-70153	1/42	0.549745	0.784319	0	0	1.273691	0.762051
Activation of gene expression by SREBF (SREBP) Homo sapiens R-HSA-2426168	1/42	0.549745	0.784319	0	0	1.273691	0.762051
Deactivation of the beta-catenin transactivating complex Homo sapiens R-HSA-3769402	1/42	0.549745	0.784319	0	0	1.273691	0.762051
Potassium Channels Homo sapiens R-HSA-1296071	2/99	0.558099	0.784406	0	0	1.07652	0.627847
CLEC7A (Dectin-1) signaling Homo sapiens R-HSA-5607764	2/99	0.558099	0.784406	0	0	1.07652	0.627847
Downstream TCR signaling Homo sapiens R-HSA-202424	2/99	0.558099	0.784406	0	0	1.07652	0.627847
Iron uptake and transport Homo sapiens R-HSA-917937	1/43	0.558227	0.784406	0	0	1.243302	0.724831
ERCC6 (CSB) and EHMT2 (G9a) positively regulate rRNA expression Homo sapiens R-HSA-427389	1/43	0.558227	0.784406	0	0	1.243302	0.724831
Formation of Incision Complex in GG-NER Homo sapiens R-HSA-5696395	1/43	0.558227	0.784406	0	0	1.243302	0.724831
Signaling by FGFR2 in disease Homo sapiens R-HSA-5655253	1/43	0.558227	0.784406	0	0	1.243302	0.724831
Intra-Golgi traffic Homo sapiens R-HSA-6811438	1/44	0.566551	0.794371	0	0	1.214326	0.689966
Transport of glucose and other sugars. bile salts and organic acids. metal ions and amine compounds Homo sapiens R-HSA-425366	2/101	0.568974	0.794371	0	0	1.054664	0.594747
Gene Silencing by RNA Homo sapiens R-HSA-211000	2/101	0.568974	0.794371	0	0	1.054664	0.594747
Diseases of metabolism Homo sapiens R-HSA-5668914	1/45	0.574717	0.800675	0	0	1.186667	0.657267
tRNA processing Homo sapiens R-HSA-72306	2/103	0.579655	0.805831	0	0	1.033674	0.563686
DNA Damage Bypass Homo sapiens R-HSA-73893	1/46	0.582731	0.808384	0	0	1.160237	0.626563
NOTCH1 Intracellular Domain Regulates Transcription	1/47	0.590594	0.81582	0	0	1.134957	0.597699

Homo sapiens R-HSA-2122947							
Nucleotide-binding domain. leucine rich repeat containing receptor (NLR) signaling pathways Homo sapiens R-HSA-168643	1/47	0.590594	0.81582	0	0	1.134957	0.597699
EPH-ephrin mediated repulsion of cells Homo sapiens R-HSA-3928665	1/48	0.598308	0.821257	0	0	1.110752	0.570536
Pyruvate metabolism and Citric Acid (TCA) cycle Homo sapiens R-HSA-71406	1/48	0.598308	0.821257	0	0	1.110752	0.570536
Death Receptor Signalling Homo sapiens R-HSA-73887	1/48	0.598308	0.821257	0	0	1.110752	0.570536
Regulation of activated PAK-2p34 by proteasome mediated degradation Homo sapiens R-HSA-211733	1/49	0.605878	0.826848	0	0	1.087556	0.544948
Disease Homo sapiens R-HSA-1643685	13/725	0.608912	0.826848	0	0	0.951249	0.471897
Deadenylation-dependent mRNA decay Homo sapiens R-HSA-429914	1/50	0.613306	0.826848	0	0	1.065306	0.520819
CDK-mediated phosphorylation and removal of Cdc6 Homo sapiens R-HSA-69017	1/50	0.613306	0.826848	0	0	1.065306	0.520819
Ubiquitin-dependent degradation of Cyclin D1 Homo sapiens R-HSA-69229	1/50	0.613306	0.826848	0	0	1.065306	0.520819
Ubiquitin-dependent degradation of Cyclin D Homo sapiens R-HSA-75815	1/50	0.613306	0.826848	0	0	1.065306	0.520819
Regulation of Apoptosis Homo sapiens R-HSA-169911	1/50	0.613306	0.826848	0	0	1.065306	0.520819
Chromatin organization Homo sapiens R-HSA-4839726	4/226	0.617021	0.826848	0	0	0.939746	0.453759
Chromatin modifying enzymes Homo sapiens R-HSA-3247509	4/226	0.617021	0.826848	0	0	0.939746	0.453759
FCERI mediated NF-kB activation Homo sapiens R-HSA-2871837	2/111	0.620424	0.826848	0	0	0.957415	0.457024
Neurotransmitter Release Cycle Homo sapiens R-HSA-112310	1/51	0.620594	0.826848	0	0	1.043947	0.498044
Vpu mediated degradation of CD4 Homo sapiens R-HSA-180534	1/51	0.620594	0.826848	0	0	1.043947	0.498044

Autodegradation of the E3 ubiquitin ligase COP1 Homo sapiens R-HSA-349425	1/51	0.620594	0.826848	0	0	1.043947	0.498044
HDR through Homologous Recombination (HR) or Single Strand Annealing (SSA) Homo sapiens R-HSA-5693567	2/112	0.625301	0.826848	0	0	0.948663	0.445419
Deposition of new CENPA-containing nucleosomes at the centromere Homo sapiens R-HSA-606279	1/52	0.627745	0.826848	0	0	1.023425	0.476528
Nucleosome assembly Homo sapiens R-HSA-774815	1/52	0.627745	0.826848	0	0	1.023425	0.476528
Nonhomologous End-Joining (NHEJ) Homo sapiens R-HSA-5693571	1/52	0.627745	0.826848	0	0	1.023425	0.476528
p53-Independent DNA Damage Response Homo sapiens R-HSA-69610	1/52	0.627745	0.826848	0	0	1.023425	0.476528
p53-Independent G1/S DNA damage checkpoint Homo sapiens R-HSA-69613	1/52	0.627745	0.826848	0	0	1.023425	0.476528
Ubiquitin Mediated Degradation of Phosphorylated Cdc25A Homo sapiens R-HSA-69601	1/52	0.627745	0.826848	0	0	1.023425	0.476528
Formation of TC-NER Pre-Incision Complex Homo sapiens R-HSA-6781823	1/53	0.634762	0.832726	0	0	1.003692	0.456184
Vif-mediated degradation of APOBEC3G Homo sapiens R-HSA-180585	1/53	0.634762	0.832726	0	0	1.003692	0.456184
Heparan sulfate/heparin (HS-GAG) metabolism Homo sapiens R-HSA-1638091	1/54	0.641646	0.833373	0	0	0.984704	0.436931
Meiotic recombination Homo sapiens R-HSA-912446	1/54	0.641646	0.833373	0	0	0.984704	0.436931
SCF-beta-TrCP mediated degradation of Emi1 Homo sapiens R-HSA-174113	1/54	0.641646	0.833373	0	0	0.984704	0.436931
Stabilization of p53 Homo sapiens R-HSA-69541	1/54	0.641646	0.833373	0	0	0.984704	0.436931
Degradation of AXIN Homo sapiens R-HSA-4641257	1/54	0.641646	0.833373	0	0	0.984704	0.436931
RNA Polymerase I. RNA Polymerase III. and Mitochondrial Transcription Homo sapiens R-HSA-504046	2/116	0.644322	0.835185	0	0	0.915189	0.402277
GABA receptor activation Homo sapiens R-HSA-977443	1/55	0.648402	0.837144	0	0	0.96642	0.418696
Regulation of cholesterol biosynthesis by SREBP	1/55	0.648402	0.837144	0	0	0.96642	0.418696

(SREBF) Homo sapiens R-HSA-1655829							
Homology Directed Repair Homo sapiens R-HSA-5693538	2/118	0.653544	0.839585	0	0	0.899318	0.382521
TCR signaling Homo sapiens R-HSA-202403	2/118	0.653544	0.839585	0	0	0.899318	0.382521
Degradation of DVL Homo sapiens R-HSA-4641258	1/56	0.65503	0.839585	0	0	0.9488	0.401413
tRNA processing in the nucleus Homo sapiens R-HSA-6784531	1/57	0.661534	0.839585	0	0	0.93181	0.385019
Meiotic synapsis Homo sapiens R-HSA-1221632	1/57	0.661534	0.839585	0	0	0.93181	0.385019
RNA Polymerase I Chain Elongation Homo sapiens R-HSA-73777	1/57	0.661534	0.839585	0	0	0.93181	0.385019
rRNA modification in the nucleus Homo sapiens R-HSA-6790901	1/58	0.667915	0.839585	0	0	0.915415	0.369457
Formation of the beta-catenin:TCF transactivating complex Homo sapiens R-HSA-201722	1/58	0.667915	0.839585	0	0	0.915415	0.369457
B-WICH complex positively regulates rRNA expression Homo sapiens R-HSA-5250924	1/58	0.667915	0.839585	0	0	0.915415	0.369457
NIK-->noncanonical NF-kB signaling Homo sapiens R-HSA-5676590	1/58	0.667915	0.839585	0	0	0.915415	0.369457
CDT1 association with the CDC6:ORC:origin complex Homo sapiens R-HSA-68827	1/58	0.667915	0.839585	0	0	0.915415	0.369457
Cell Cycle Checkpoints Homo sapiens R-HSA-69620	3/182	0.668221	0.839585	0	0	0.87371	0.352224
Telomere Maintenance Homo sapiens R-HSA-157579	1/59	0.674176	0.839585	0	0	0.899586	0.354674
Degradation of GLI1 by the proteasome Homo sapiens R-HSA-5610780	1/59	0.674176	0.839585	0	0	0.899586	0.354674
GLI3 is processed to GLI3R by the proteasome Homo sapiens R-HSA-5610785	1/59	0.674176	0.839585	0	0	0.899586	0.354674
Degradation of GLI2 by the proteasome Homo sapiens R-HSA-5610783	1/59	0.674176	0.839585	0	0	0.899586	0.354674
C-type lectin receptors (CLRs) Homo sapiens R-HSA-5621481	2/123	0.675764	0.839585	0	0	0.861935	0.337802
S Phase Homo sapiens R-HSA-69242	2/124	0.680067	0.839585	0	0	0.854826	0.32959

Cleavage of Growing Transcript in the Termination Region Homo sapiens R-HSA-109688	1/60	0.68032	0.839585	0	0	0.884294	0.340623
RNA Polymerase II Transcription Termination Homo sapiens R-HSA-73856	1/60	0.68032	0.839585	0	0	0.884294	0.340623
Post-Elongation Processing of the Transcript Homo sapiens R-HSA-76044	1/60	0.68032	0.839585	0	0	0.884294	0.340623
RHO GTPases activate PKNs Homo sapiens R-HSA-5625740	1/60	0.68032	0.839585	0	0	0.884294	0.340623
HDACs deacetylate histones Homo sapiens R-HSA-3214815	1/60	0.68032	0.839585	0	0	0.884294	0.340623
Immune System Homo sapiens R-HSA-168256	27/1547	0.685598	0.839585	0	0	0.921445	0.347812
Cell-cell junction organization Homo sapiens R-HSA-421270	1/61	0.686348	0.839585	0	0	0.869511	0.327258
Cell death signalling via NRAGE. NRIF and NADE Homo sapiens R-HSA-204998	1/61	0.686348	0.839585	0	0	0.869511	0.327258
TP53 Regulates Transcription of DNA Repair Genes Homo sapiens R-HSA-6796648	1/61	0.686348	0.839585	0	0	0.869511	0.327258
Dectin-1 mediated noncanonical NF-kB signaling Homo sapiens R-HSA-5607761	1/61	0.686348	0.839585	0	0	0.869511	0.327258
Cytochrome P450 - arranged by substrate type Homo sapiens R-HSA-211897	1/62	0.692263	0.842081	0	0	0.855213	0.314539
Gap-filling DNA repair synthesis and ligation in TC-NER Homo sapiens R-HSA-6782210	1/62	0.692263	0.842081	0	0	0.855213	0.314539
Autodegradation of Cdh1 by Cdh1:APC/C Homo sapiens R-HSA-174084	1/62	0.692263	0.842081	0	0	0.855213	0.314539
Late Phase of HIV Life Cycle Homo sapiens R-HSA-162599	2/128	0.696813	0.844414	0	0	0.827519	0.298931
Dual incision in TC-NER Homo sapiens R-HSA-6782135	1/63	0.698066	0.844414	0	0	0.841376	0.302426
Signaling by FGFR in disease Homo sapiens R-HSA-1226099	1/63	0.698066	0.844414	0	0	0.841376	0.302426
G alpha (q) signalling events Homo sapiens R-HSA-416476	3/191	0.699652	0.844765	0	0	0.831498	0.296988
HDR through Homologous Recombination (HRR) Homo sapiens R-HSA-5685942	1/64	0.70376	0.848155	0	0	0.827979	0.290883

Peptide ligand-binding receptors Homo sapiens R-HSA-375276	3/193	0.706322	0.849671	0	0	0.822661	0.286026
ER-Phagosome pathway Homo sapiens R-HSA-1236974	1/65	0.709347	0.851739	0	0	0.815	0.279879
APC/C:Cdc20 mediated degradation of Securin Homo sapiens R-HSA-174154	1/66	0.714829	0.855172	0	0	0.802421	0.269382
Cytosolic sensors of pathogen-associated DNA Homo sapiens R-HSA-1834949	1/66	0.714829	0.855172	0	0	0.802421	0.269382
Regulation of RAS by GAPs Homo sapiens R-HSA-5658442	1/67	0.720208	0.857318	0	0	0.790222	0.259363
Assembly of the pre-replicative complex Homo sapiens R-HSA-68867	1/67	0.720208	0.857318	0	0	0.790222	0.259363
mRNA Splicing - Major Pathway Homo sapiens R-HSA-72163	2/134	0.720568	0.857318	0	0	0.789661	0.258784
Antigen processing: Ubiquitination & Proteasome degradation Homo sapiens R-HSA-983168	4/260	0.723329	0.859036	0	0	0.813508	0.263488
TCF dependent signaling in response to WNT Homo sapiens R-HSA-201681	3/199	0.725651	0.859839	0	0	0.797231	0.255661
Influenza Life Cycle Homo sapiens R-HSA-168255	2/136	0.728131	0.859839	0	0	0.777796	0.246774
Signaling by Hedgehog Homo sapiens R-HSA-5358351	2/136	0.728131	0.859839	0	0	0.777796	0.246774
Class A/1 (Rhodopsin-like receptors) Homo sapiens R-HSA-373076	5/323	0.729281	0.859839	0	0	0.818203	0.258304
GPCR ligand binding Homo sapiens R-HSA-500792	7/447	0.738871	0.868627	0	0	0.8271	0.250307
APC/C:Cdh1 mediated degradation of Cdc20 and other APC/C:Cdh1 targeted proteins in late mitosis/early G1 Homo sapiens R-HSA-174178	1/71	0.740731	0.868627	0	0	0.744914	0.223562
Cdc20:Phospho-APC/C mediated degradation of Cyclin A Homo sapiens R-HSA-174184	1/71	0.740731	0.868627	0	0	0.744914	0.223562
Transcriptional regulation by small RNAs Homo sapiens R-HSA-5578749	1/72	0.745623	0.871229	0	0	0.734385	0.215568

APC:Cdc20 mediated degradation of cell cycle proteins prior to satisfation of the cell cycle checkpoint Homo sapiens R-HSA-179419	1/72	0.745623	0.871229	0	0	0.734385	0.215568
G alpha (s) signalling events Homo sapiens R-HSA-418555	2/142	0.749787	0.872148	0	0	0.744232	0.214314
Positive epigenetic regulation of rRNA expression Homo sapiens R-HSA-5250913	1/73	0.750422	0.872148	0	0	0.724148	0.207917
NoRC negatively regulates rRNA expression Homo sapiens R-HSA-427413	1/73	0.750422	0.872148	0	0	0.724148	0.207917
APC/C:Cdc20 mediated degradation of mitotic proteins Homo sapiens R-HSA-176409	1/74	0.755131	0.874731	0	0	0.714192	0.20059
mRNA Splicing Homo sapiens R-HSA-72172	2/144	0.756669	0.874731	0	0	0.733675	0.20457
Beta-catenin independent WNT signaling Homo sapiens R-HSA-3858494	2/144	0.756669	0.874731	0	0	0.733675	0.20457
Activation of APC/C and APC/C:Cdc20 mediated degradation of mitotic proteins Homo sapiens R-HSA-176814	1/75	0.759752	0.875533	0	0	0.704505	0.193572
DNA Double-Strand Break Repair Homo sapiens R-HSA-5693532	2/145	0.760048	0.875533	0	0	0.728507	0.199883
Negative epigenetic regulation of rRNA expression Homo sapiens R-HSA-5250941	1/76	0.764286	0.875772	0	0	0.695076	0.186846
RNA Polymerase I Promoter Clearance Homo sapiens R-HSA-73854	1/76	0.764286	0.875772	0	0	0.695076	0.186846
Transcription-Coupled Nucleotide Excision Repair (TC-NER) Homo sapiens R-HSA-6781827	1/76	0.764286	0.875772	0	0	0.695076	0.186846
Cell Cycle. Mitotic Homo sapiens R-HSA-69278	7/462	0.768782	0.877253	0	0	0.799208	0.21015
Metabolism of vitamins and cofactors Homo sapiens R-HSA-196854	2/148	0.769945	0.877253	0	0	0.713428	0.186516
G2/M DNA damage checkpoint Homo sapiens R-HSA-69473	1/78	0.773098	0.877253	0	0	0.676952	0.174213
Signal Transduction Homo sapiens R-HSA-162582	42/2465	0.776066	0.877253	0	0	0.892695	0.226313

G2/M Checkpoints Homo sapiens R-HSA-69481	2/150	0.776343	0.877253	0	0	0.703714	0.178153
PI3K Cascade Homo sapiens R-HSA-109704	1/79	0.777381	0.877253	0	0	0.668239	0.168279
Regulation of APC/C activators between G1/S and early anaphase Homo sapiens R-HSA-176408	1/79	0.777381	0.877253	0	0	0.668239	0.168279
RIG-I/MDA5 mediated induction of IFN-alpha/beta pathways Homo sapiens R-HSA-168928	1/79	0.777381	0.877253	0	0	0.668239	0.168279
Fatty acid. triacylglycerol. and ketone body metabolism Homo sapiens R-HSA-535734	3/217	0.777688	0.877253	0	0	0.729498	0.183418
Complement cascade Homo sapiens R-HSA-166658	1/80	0.781583	0.878876	0	0	0.659747	0.162584
Extracellular matrix organization Homo sapiens R-HSA-1474244	4/283	0.781822	0.878876	0	0	0.745558	0.183503
The citric acid (TCA) cycle and respiratory electron transport Homo sapiens R-HSA-1428517	2/153	0.78565	0.879667	0	0	0.689627	0.166368
Peptide hormone metabolism Homo sapiens R-HSA-2980736	1/81	0.785705	0.879667	0	0	0.651467	0.157116
Global Genome Nucleotide Excision Repair (GG-NER) Homo sapiens R-HSA-5696399	1/82	0.789751	0.879667	0	0	0.643391	0.151865
Degradation of beta-catenin by the destruction complex Homo sapiens R-HSA-195253	1/82	0.789751	0.879667	0	0	0.643391	0.151865
Antigen processing-Cross presentation Homo sapiens R-HSA-1236975	1/82	0.789751	0.879667	0	0	0.643391	0.151865
HIV Infection Homo sapiens R-HSA-162906	3/222	0.790621	0.879667	0	0	0.712659	0.16743
p75 NTR receptor-mediated signalling Homo sapiens R-HSA-193704	1/83	0.793719	0.879689	0	0	0.635512	0.146819
Immunoregulatory interactions between a Lymphoid and a non-Lymphoid cell Homo sapiens R-HSA-198933	2/157	0.797529	0.879689	0	0	0.671692	0.151962
Mitochondrial translation initiation Homo sapiens R-HSA-5368286	1/84	0.797614	0.879689	0	0	0.627823	0.14197

Mitochondrial translation termination Homo sapiens R-HSA-5419276	1/84	0.797614	0.879689	0	0	0.627823	0.14197
Mitochondrial translation elongation Homo sapiens R-HSA-5389840	1/84	0.797614	0.879689	0	0	0.627823	0.14197
Meiosis Homo sapiens R-HSA-1500620	1/85	0.801435	0.879689	0	0	0.620317	0.137309
APC/C-mediated degradation of cell cycle proteins Homo sapiens R-HSA-174143	1/85	0.801435	0.879689	0	0	0.620317	0.137309
Regulation of mitotic cell cycle Homo sapiens R-HSA-453276	1/85	0.801435	0.879689	0	0	0.620317	0.137309
Cell junction organization Homo sapiens R-HSA-446728	1/86	0.805183	0.880838	0	0	0.612988	0.132825
Chromosome Maintenance Homo sapiens R-HSA-73886	1/86	0.805183	0.880838	0	0	0.612988	0.132825
Signaling by Wnt Homo sapiens R-HSA-195721	4/295	0.808218	0.882677	0	0	0.71437	0.152106
Apoptosis Homo sapiens R-HSA-109581	2/163	0.814256	0.887784	0	0	0.646461	0.132835
Regulation of TP53 Activity through Phosphorylation Homo sapiens R-HSA-6804756	1/89	0.816012	0.888213	0	0	0.592	0.120369
Mitochondrial translation Homo sapiens R-HSA-5368287	1/90	0.819486	0.888957	0	0	0.585318	0.116524
Vesicle-mediated transport Homo sapiens R-HSA-5653656	7/492	0.820953	0.888957	0	0	0.748599	0.14769
Major pathway of rRNA processing in the nucleolus Homo sapiens R-HSA-6791226	2/166	0.822149	0.888957	0	0	0.634538	0.124264
Programmed Cell Death Homo sapiens R-HSA-5357801	2/166	0.822149	0.888957	0	0	0.634538	0.124264
Class I MHC mediated antigen processing & presentation Homo sapiens R-HSA-983169	4/305	0.828181	0.893996	0	0	0.69028	0.130134
L1CAM interactions Homo sapiens R-HSA-373760	1/96	0.839001	0.904179	0	0	0.548182	0.09623
Regulation of actin dynamics for phagocytic cup formation Homo sapiens R-HSA-2029482	1/97	0.842042	0.90596	0	0	0.542444	0.09326
Hedgehog 'off' state Homo sapiens R-HSA-5610787	1/99	0.847954	0.910817	0	0	0.53132	0.08763

Stimuli-sensing channels Homo sapiens R-HSA-2672351	1/100	0.850827	0.910901	0	0	0.525926	0.084962
Phase II conjugation Homo sapiens R-HSA-156580	1/100	0.850827	0.910901	0	0	0.525926	0.084962
Adaptive Immune System Homo sapiens R-HSA-1280218	11/762	0.852384	0.910927	0	0	0.757357	0.120964
Cell surface interactions at the vascular wall Homo sapiens R-HSA-202733	1/101	0.853645	0.910927	0	0	0.52064	0.082386
rRNA processing Homo sapiens R-HSA-72312	2/180	0.855138	0.91103	0	0	0.58421	0.091424
Platelet degranulation Homo sapiens R-HSA-114608	1/105	0.864398	0.919392	0	0	0.500513	0.072936
Assembly of the primary cilium Homo sapiens R-HSA-5617833	2/187	0.869453	0.921785	0	0	0.561902	0.078605
Mitotic Prophase Homo sapiens R-HSA-68875	1/107	0.869475	0.921785	0	0	0.491019	0.068677
Nucleotide Excision Repair Homo sapiens R-HSA-5696398	1/108	0.871942	0.9229	0	0	0.486405	0.066653
Respiratory electron transport. ATP synthesis by chemiosmotic coupling. and heat production by uncoupling proteins. Homo sapiens R-HSA-163200	1/109	0.874363	0.923962	0	0	0.481877	0.064697
Response to elevated platelet cytosolic Ca2+ Homo sapiens R-HSA-76005	1/110	0.876738	0.924972	0	0	0.477431	0.062805
Processing of Capped Intron-Containing Pre-mRNA Homo sapiens R-HSA-72203	2/193	0.880677	0.927628	0	0	0.544083	0.069133
Epigenetic regulation of gene expression Homo sapiens R-HSA-212165	1/115	0.887957	0.933787	0	0	0.456374	0.054232
Fcgamma receptor (FCGR) dependent phagocytosis Homo sapiens R-HSA-2029480	1/120	0.898158	0.941982	0	0	0.437087	0.046947
Membrane Trafficking Homo sapiens R-HSA-199991	5/420	0.898925	0.941982	0	0	0.623811	0.066471
Glycosaminoglycan metabolism Homo sapiens R-HSA-1630316	1/121	0.900084	0.941982	0	0	0.433422	0.045625
Rho GTPase cycle Homo sapiens R-HSA-194840	1/122	0.901974	0.942447	0	0	0.429818	0.044344
RNA Polymerase II Transcription Homo sapiens R-HSA-73857	1/124	0.905647	0.943819	0	0	0.422786	0.0419

DNA Repair Homo sapiens R-HSA-73894	3/285	0.906182	0.943819	0	0	0.551651	0.054346
Cell Cycle Homo sapiens R-HSA-1640170	7/566	0.91063	0.946939	0	0	0.646989	0.06057
Influenza Viral RNA Transcription and Replication Homo sapiens R-HSA-168273	1/128	0.912587	0.947463	0	0	0.409386	0.037447
Host Interactions of HIV factors Homo sapiens R-HSA-162909	1/129	0.914241	0.947671	0	0	0.406167	0.036417
Cell-Cell communication Homo sapiens R-HSA-1500931	1/131	0.917456	0.949494	0	0	0.399877	0.03445
Cardiac conduction Homo sapiens R-HSA-5576891	1/135	0.923529	0.954265	0	0	0.387861	0.030855
Signaling by GPCR Homo sapiens R-HSA-372790	18/1293	0.930553	0.960001	0	0	0.723589	0.052081
G alpha (i) signalling events Homo sapiens R-HSA-418594	2/240	0.942159	0.970438	0	0	0.435582	0.025953
Organelle biogenesis and maintenance Homo sapiens R-HSA-1852241	3/326	0.946479	0.97335	0	0	0.480607	0.026437
Separation of Sister Chromatids Homo sapiens R-HSA-2467813	1/162	0.954367	0.979917	0	0	0.322369	0.015057
SLC-mediated transmembrane transport Homo sapiens R-HSA-425407	2/268	0.962947	0.983337	0	0	0.389168	0.014694
M Phase Homo sapiens R-HSA-68886	2/268	0.962947	0.983337	0	0	0.389168	0.014694
Mitotic Anaphase Homo sapiens R-HSA-68882	1/173	0.96303	0.983337	0	0	0.301581	0.011361
Mitotic Metaphase and Anaphase Homo sapiens R-HSA-2555396	1/174	0.963731	0.983337	0	0	0.299823	0.011076
Intra-Golgi and retrograde Golgi-to-ER traffic Homo sapiens R-HSA-6811442	1/179	0.967042	0.985174	0	0	0.291326	0.009763
Muscle contraction Homo sapiens R-HSA-397014	1/196	0.976204	0.992956	0	0	0.265696	0.006399
Ion channel transport Homo sapiens R-HSA-983712	1/203	0.979192	0.993014	0	0	0.256396	0.005391
Hemostasis Homo sapiens R-HSA-109582	5/552	0.979307	0.993014	0	0	0.470023	0.009828
Platelet activation. signaling and aggregation Homo sapiens R-HSA-76002	1/253	0.992031	0.999956	0	0	0.204995	0.00164
RHO GTPase Effectors Homo sapiens R-HSA-195258	1/255	0.992331	0.999956	0	0	0.20336	0.001565
Signaling by Rho GTPases Homo sapiens R-HSA-194315	2/367	0.992774	0.999956	0	0	0.282162	0.002046

Metabolism Homo sapiens R-HSA-1430728	23/1908	0.993784	0.999956	0	0	0.613156	0.003823
Transmembrane transport of small molecules Homo sapiens R-HSA-382551	4/594	0.996284	0.999956	0	0	0.346893	0.001292
Metabolism of amino acids and derivatives Homo sapiens R-HSA-71291	1/335	0.998356	0.999956	0	0	0.154012	2.53E-04
GPCR downstream signaling Homo sapiens R-HSA-388396	7/983	0.999447	0.999956	0	0	0.362455	2.01E-04
Metabolism of lipids and lipoproteins Homo sapiens R-HSA-556833	3/659	0.9997	0.999956	0	0	0.232557	6.98E-05
Metabolism of proteins Homo sapiens R-HSA-392499	6/1074	0.999956	0.999956	0	0	0.281749	1.24E-05

siLonp_HS vs Ctrl							
Term	Overlap	P-value	Adjusted P-value	Old P-value	Old Adjusted P-value	Odds Ratio	Combined Score
Attenuation phase Homo sapiens R-HSA-3371568	14/26	4.10E-17	2.94E-14	0	0	53.2796	2010.43
HSF1-dependent transactivation Homo sapiens R-HSA-3371571	14/34	5.03E-15	1.80E-12	0	0	31.95467	1052.079
HSF1 activation Homo sapiens R-HSA-3371511	13/29	1.24E-14	2.96E-12	0	0	37.01136	1185.178
Regulation of HSF1-mediated heat shock response Homo sapiens R-HSA-3371453	17/80	1.49E-12	2.68E-10	0	0	12.37778	337.0594
Cellular response to heat stress Homo sapiens R-HSA-3371556	18/96	3.15E-12	4.52E-10	0	0	10.60232	280.7873
Cellular responses to stress Homo sapiens R-HSA-2262752	30/367	8.46E-10	1.01E-07	0	0	4.153078	86.75814
Generic Transcription Pathway Homo sapiens R-HSA-212436	44/812	4.28E-08	4.39E-06	0	0	2.7048	45.89384
Senescence-Associated Secretory Phenotype (SASP) Homo sapiens R-HSA-2559582	9/77	4.95E-05	0.00444	0	0	5.957411	59.06242
Developmental Biology Homo sapiens R-HSA-1266738	34/786	1.60E-04	0.012779	0	0	2.083998	18.21254
Receptor-ligand binding initiates the second proteolytic cleavage of Notch receptor Homo sapiens R-HSA-156988	4/14	1.98E-04	0.014184	0	0	17.85205	152.2695

Negative regulation of MAPK pathway Homo sapiens R-HSA-5675221	6/40	2.29E-04	0.01462	0	0	7.902321	66.24864
Constitutive Signaling by NOTCH1 HD Domain Mutants Homo sapiens R-HSA-2691232	4/15	2.65E-04	0.01462	0	0	16.22831	133.6712
Signaling by NOTCH1 HD Domain Mutants in Cancer Homo sapiens R-HSA-2691230	4/15	2.65E-04	0.01462	0	0	16.22831	133.6712
NOTCH2 Activation and Transmission of Signal to the Nucleus Homo sapiens R-HSA-2979096	4/21	0.001045	0.052789	0	0	10.49745	72.05277
Gene Expression Homo sapiens R-HSA-74160	55/1631	0.001103	0.052789	0	0	1.621562	11.04263
RAF-independent MAPK1/3 activation Homo sapiens R-HSA-112409	4/23	0.001493	0.066995	0	0	9.391492	61.11056
ATF4 activates genes Homo sapiens R-HSA-380994	4/25	0.00206	0.086993	0	0	8.496195	52.55055
Downregulation of TGF-beta receptor signaling Homo sapiens R-HSA-2173788	4/26	0.002392	0.095423	0	0	8.109589	48.94572
Cellular Senescence Homo sapiens R-HSA-2559583	10/161	0.003152	0.1136	0	0	2.975074	17.13573
PERK regulates gene expression Homo sapiens R-HSA-381042	4/28	0.003164	0.1136	0	0	7.433029	42.7831
Amyloid fiber formation Homo sapiens R-HSA-977225	6/68	0.003882	0.13273	0	0	4.327316	24.02261
Activated NOTCH1 Transmits Signal to the Nucleus Homo sapiens R-HSA-2122948	4/30	0.004091	0.133511	0	0	6.860555	37.7262
TRAF6 mediated induction of TAK1 complex Homo sapiens R-HSA-937072	3/16	0.004846	0.148915	0	0	10.27422	54.75814
TGF-beta receptor signaling activates SMADs Homo sapiens R-HSA-2173789	4/32	0.005185	0.148915	0	0	6.369863	33.51806
Signaling by NOTCH2 Homo sapiens R-HSA-1980145	4/32	0.005185	0.148915	0	0	6.369863	33.51806
AUF1 (hnRNP D0) binds and destabilizes mRNA Homo sapiens R-HSA-450408	5/54	0.006701	0.170622	0	0	4.55541	22.80221
Activation of IRF3/IRF7 mediated by TBK1/IKK epsilon Homo sapiens R-HSA-936964	3/18	0.006834	0.170622	0	0	8.903417	44.39144
Scavenging by Class F Receptors Homo sapiens R-HSA-3000484	2/6	0.006891	0.170622	0	0	22.22045	110.6018

Uptake and function of diphtheria toxin Homo sapiens R-HSA-5336415	2/6	0.006891	0.170622	0	0	22.22045	110.6018
Constitutive Signaling by Ligand-Responsive EGFR Cancer Variants Homo sapiens R-HSA-1236382	3/19	0.007984	0.175295	0	0	8.346526	40.31674
Signaling by Ligand-Responsive EGFR Variants in Cancer Homo sapiens R-HSA-5637815	3/19	0.007984	0.175295	0	0	8.346526	40.31674
Signaling by EGFR in Cancer Homo sapiens R-HSA-1643713	3/19	0.007984	0.175295	0	0	8.346526	40.31674
Transcriptional regulation of white adipocyte differentiation Homo sapiens R-HSA-381340	6/79	0.008057	0.175295	0	0	3.673181	17.70931
Signaling by NOTCH1 t(7;9)(NOTCH1:M1580 K2555) Translocation Mutant Homo sapiens R-HSA-2660825	2/7	0.009508	0.19504	0	0	17.77545	82.75666
Constitutive Signaling by NOTCH1 t(7;9)(NOTCH1:M1580 K2555) Translocation Mutant Homo sapiens R-HSA-2660826	2/7	0.009508	0.19504	0	0	17.77545	82.75666
MAPK family signaling cascades Homo sapiens R-HSA-5683057	13/284	0.010919	0.217765	0	0	2.156659	9.74225
Regulation of mRNA stability by proteins that bind AU-rich elements Homo sapiens R-HSA-450531	6/86	0.011992	0.22424	0	0	3.350573	14.82144
VEGF ligand-receptor interactions Homo sapiens R-HSA-194313	2/8	0.012492	0.22424	0	0	14.81212	64.91601
VEGF binds to VEGFR leading to receptor dimerization Homo sapiens R-HSA-195399	2/8	0.012492	0.22424	0	0	14.81212	64.91601
Downregulation of ERBB4 signaling Homo sapiens R-HSA-1253288	2/8	0.012492	0.22424	0	0	14.81212	64.91601
Signaling by SCF-KIT Homo sapiens R-HSA-1433557	14/325	0.013816	0.237299	0	0	2.024356	8.668212
Signaling by VEGF Homo sapiens R-HSA-194138	14/328	0.014854	0.237299	0	0	2.004703	8.438748
IKK complex recruitment mediated by RIP1 Homo sapiens R-HSA-937041	3/24	0.015373	0.237299	0	0	6.357631	26.54391

Oxidative Stress Induced Senescence Homo sapiens R-HSA-2559580	6/91	0.015521	0.237299	0	0	3.152671	13.1326
PTK6 Regulates RTKs and Their Effectors AKT1 and DOK1 Homo sapiens R-HSA-8849469	2/9	0.015829	0.237299	0	0	12.69545	52.6344
MyD88:Mal cascade initiated on plasma membrane Homo sapiens R-HSA-166058	6/92	0.016305	0.237299	0	0	3.115852	12.82575
Toll Like Receptor TLR1:TLR2 Cascade Homo sapiens R-HSA-168179	6/92	0.016305	0.237299	0	0	3.115852	12.82575
Toll Like Receptor TLR6:TLR2 Cascade Homo sapiens R-HSA-168188	6/92	0.016305	0.237299	0	0	3.115852	12.82575
Toll Like Receptor 2 (TLR2) Cascade Homo sapiens R-HSA-181438	6/92	0.016305	0.237299	0	0	3.115852	12.82575
Signaling by ERBB2 Homo sapiens R-HSA-1227986	4/45	0.017174	0.237299	0	0	4.347255	17.6688
Regulation of Hypoxia-inducible Factor (HIF) by oxygen Homo sapiens R-HSA-1234174	3/26	0.019119	0.237299	0	0	5.804199	22.96772
Cellular response to hypoxia Homo sapiens R-HSA-2262749	3/26	0.019119	0.237299	0	0	5.804199	22.96772
Activation of PPARGC1A (PGC-1alpha) by phosphorylation Homo sapiens R-HSA-2151209	2/10	0.0195	0.237299	0	0	11.10795	43.73608
POU5F1 (OCT4). SOX2. NANOG repress genes related to differentiation Homo sapiens R-HSA-2892245	2/10	0.0195	0.237299	0	0	11.10795	43.73608
IRAK2 mediated activation of TAK1 complex upon TLR7/8 or 9 stimulation Homo sapiens R-HSA-975163	2/10	0.0195	0.237299	0	0	11.10795	43.73608
IRAK2 mediated activation of TAK1 complex Homo sapiens R-HSA-937042	2/10	0.0195	0.237299	0	0	11.10795	43.73608
Diseases of carbohydrate metabolism Homo sapiens R-HSA-5663084	2/10	0.0195	0.237299	0	0	11.10795	43.73608
Myoclonic epilepsy of Lafora Homo sapiens R-HSA-3785653	2/10	0.0195	0.237299	0	0	11.10795	43.73608
Glycogen storage diseases Homo sapiens R-HSA-3229121	2/10	0.0195	0.237299	0	0	11.10795	43.73608

Axon guidance Homo sapiens R-HSA-422475	19/515	0.021094	0.251711	0	0	1.726235	6.66115
Signaling by NOTCH1 Homo sapiens R-HSA-1980143	5/72	0.021521	0.251711	0	0	3.328495	12.77719
VEGFR2 mediated cell proliferation Homo sapiens R-HSA-5218921	11/248	0.022549	0.251711	0	0	2.080639	7.889938
Transcriptional Regulation by TP53 Homo sapiens R-HSA-3700989	14/348	0.023387	0.251711	0	0	1.882702	7.070663
Signaling by NOTCH3 Homo sapiens R-HSA-1980148	2/11	0.023488	0.251711	0	0	9.873232	37.03698
Signaling by NOTCH4 Homo sapiens R-HSA-1980150	2/11	0.023488	0.251711	0	0	9.873232	37.03698
Membrane binding and targetting of GAG proteins Homo sapiens R-HSA-174490	2/11	0.023488	0.251711	0	0	9.873232	37.03698
Synthesis And Processing Of GAG. GAGPOL Polyproteins Homo sapiens R-HSA-174495	2/11	0.023488	0.251711	0	0	9.873232	37.03698
Negative regulation of FGFR3 signaling Homo sapiens R-HSA-5654732	3/29	0.025598	0.264466	0	0	5.133695	18.81614
VEGFA-VEGFR2 Pathway Homo sapiens R-HSA-4420097	13/320	0.026378	0.264466	0	0	1.900207	6.907711
RNA Polymerase I Promoter Opening Homo sapiens R-HSA-73728	3/30	0.027988	0.264466	0	0	4.943305	17.67712
Fc epsilon receptor (FCERI) signaling Homo sapiens R-HSA-2454202	15/395	0.030333	0.264466	0	0	1.772895	6.197202
Packaging Of Telomere Ends Homo sapiens R-HSA-171306	3/31	0.030492	0.264466	0	0	4.766515	16.63647
Negative regulation of FGFR4 signaling Homo sapiens R-HSA-5654733	3/31	0.030492	0.264466	0	0	4.766515	16.63647
Interleukin-3. 5 and GM-CSF signaling Homo sapiens R-HSA-512988	11/261	0.031178	0.264466	0	0	1.971118	6.835943
p75NTR recruits signalling complexes Homo sapiens R-HSA-209543	2/13	0.032358	0.264466	0	0	8.077273	27.71236
NF-kB is activated and signals survival Homo sapiens R-HSA-209560	2/13	0.032358	0.264466	0	0	8.077273	27.71236
Assembly Of The HIV Virion Homo sapiens R-HSA-175474	2/13	0.032358	0.264466	0	0	8.077273	27.71236
Downregulation of ERBB2:ERBB3 signaling Homo sapiens R-HSA-1358803	2/13	0.032358	0.264466	0	0	8.077273	27.71236

DNA methylation Homo sapiens R-HSA-5334118	3/32	0.03311	0.264 466	0	0	4.601917	15.682 91
Oncogene Induced Senescence Homo sapiens R-HSA-2559585	3/32	0.03311	0.264 466	0	0	4.601917	15.682 91
SMAD2/SMAD3:SMAD4 heterotrimer regulates transcription Homo sapiens R-HSA-2173796	3/32	0.03311	0.264 466	0	0	4.601917	15.682 91
Negative regulation of FGFR1 signaling Homo sapiens R-HSA-5654726	3/32	0.03311	0.264 466	0	0	4.601917	15.682 91
NCAM signaling for neurite out-growth Homo sapiens R-HSA-375165	11/266	0.035052	0.264 466	0	0	1.931969	6.4738 85
GRB2 events in EGFR signaling Homo sapiens R-HSA-179812	10/235	0.036703	0.264 466	0	0	1.988992	6.5734 16
SHC1 events in EGFR signaling Homo sapiens R-HSA-180336	10/235	0.036703	0.264 466	0	0	1.988992	6.5734 16
SOS-mediated signalling Homo sapiens R-HSA-112412	10/235	0.036703	0.264 466	0	0	1.988992	6.5734 16
SHC1 events in ERBB4 signaling Homo sapiens R-HSA-1250347	10/235	0.036703	0.264 466	0	0	1.988992	6.5734 16
RAF/MAP kinase cascade Homo sapiens R-HSA-5673001	10/235	0.036703	0.264 466	0	0	1.988992	6.5734 16
Constitutive Signaling by NOTCH1 HD+PEST Domain Mutants Homo sapiens R-HSA-2894862	4/57	0.037112	0.264 466	0	0	3.360903	11.070 21
Signaling by NOTCH1 in Cancer Homo sapiens R-HSA-2644603	4/57	0.037112	0.264 466	0	0	3.360903	11.070 21
Signaling by NOTCH1 PEST Domain Mutants in Cancer Homo sapiens R-HSA-2644602	4/57	0.037112	0.264 466	0	0	3.360903	11.070 21
Constitutive Signaling by NOTCH1 PEST Domain Mutants Homo sapiens R-HSA-2644606	4/57	0.037112	0.264 466	0	0	3.360903	11.070 21
Signaling by NOTCH1 HD+PEST Domain Mutants in Cancer Homo sapiens R-HSA-2894858	4/57	0.037112	0.264 466	0	0	3.360903	11.070 21
FRS-mediated FGFR2 signaling Homo sapiens R-HSA-5654700	10/236	0.037607	0.264 466	0	0	1.980088	6.4958 1

FRS-mediated FGFR4 signaling Homo sapiens R-HSA-5654712	10/236	0.037607	0.264466		0	0	1.980088	6.49581
FRS-mediated FGFR3 signaling Homo sapiens R-HSA-5654706	10/236	0.037607	0.264466		0	0	1.980088	6.49581
FRS-mediated FGFR1 signaling Homo sapiens R-HSA-5654693	10/236	0.037607	0.264466		0	0	1.980088	6.49581
Activated TLR4 signalling Homo sapiens R-HSA-166054	6/112	0.038102	0.264466		0	0	2.525359	8.251554
Activated PKN1 stimulates transcription of AR (androgen receptor) regulated genes KLK2 and KLK3 Homo sapiens R-HSA-5625886	3/34	0.038685	0.264466		0	0	4.304578	13.9998
Negative regulation of FGFR2 signaling Homo sapiens R-HSA-5654727	3/34	0.038685	0.264466		0	0	4.304578	13.9998
Formation of the beta-catenin:TCF transactivating complex Homo sapiens R-HSA-201722	4/58	0.039191	0.264466		0	0	3.298495	10.68482
Recruitment and ATM-mediated phosphorylation of repair and signaling proteins at DNA double strand breaks Homo sapiens R-HSA-5693565	4/58	0.039191	0.264466		0	0	3.298495	10.68482
ARMS-mediated activation Homo sapiens R-HSA-170984	10/239	0.040411	0.264466		0	0	1.953845	6.269193
Signalling to p38 via RIT and RIN Homo sapiens R-HSA-187706	10/239	0.040411	0.264466		0	0	1.953845	6.269193
DNA Damage/Telomere Stress Induced Senescence Homo sapiens R-HSA-2559586	4/59	0.041336	0.264466		0	0	3.238356	10.31747
DNA Double Strand Break Response Homo sapiens R-HSA-5693606	4/59	0.041336	0.264466		0	0	3.238356	10.31747
The role of GTSE1 in G2/M progression after G2 checkpoint Homo sapiens R-HSA-8852276	4/59	0.041336	0.264466		0	0	3.238356	10.31747
Frs2-mediated activation Homo sapiens R-HSA-170968	10/240	0.041377	0.264466		0	0	1.94525	6.195666
SIRT1 negatively regulates rRNA Expression Homo sapiens R-HSA-427359	3/35	0.04164	0.264466		0	0	4.169846	13.2547

Fanconi Anemia Pathway Homo sapiens R-HSA-6783310	3/35	0.04164	0.264466	0	0	4.169846	13.2547
Regulation of innate immune responses to cytosolic DNA Homo sapiens R-HSA-3134975	2/15	0.042317	0.264466	0	0	6.833916	21.61276
Association of licensing factors with the pre-replicative complex Homo sapiens R-HSA-69298	2/15	0.042317	0.264466	0	0	6.833916	21.61276
NRIF signals cell death from the nucleus Homo sapiens R-HSA-205043	2/15	0.042317	0.264466	0	0	6.833916	21.61276
Glycogen synthesis Homo sapiens R-HSA-3322077	2/15	0.042317	0.264466	0	0	6.833916	21.61276
MAPK1/MAPK3 signaling Homo sapiens R-HSA-5684996	10/241	0.042359	0.264466	0	0	1.936728	6.123119
Prolonged ERK activation events Homo sapiens R-HSA-169893	10/242	0.043356	0.267231	0	0	1.928281	6.051536
HDACs deacetylate histones Homo sapiens R-HSA-3214815	4/60	0.043546	0.267231	0	0	3.180365	9.967065
Signaling by Leptin Homo sapiens R-HSA-2586552	10/243	0.044369	0.269503	0	0	1.919905	5.980901
Interleukin receptor SHC signaling Homo sapiens R-HSA-912526	10/245	0.046444	0.269503	0	0	1.903369	5.842415
Activation of anterior HOX genes in hindbrain development during early embryogenesis Homo sapiens R-HSA-5617472	5/89	0.047345	0.269503	0	0	2.652555	8.091067
Activation of HOX genes during differentiation Homo sapiens R-HSA-5619507	5/89	0.047345	0.269503	0	0	2.652555	8.091067
Signalling to RAS Homo sapiens R-HSA-167044	10/246	0.047505	0.269503	0	0	1.895206	5.774535
TGF-beta receptor signaling in EMT (epithelial to mesenchymal transition) Homo sapiens R-HSA-2173791	2/16	0.04767	0.269503	0	0	6.345455	19.31213
Translesion synthesis by REV1 Homo sapiens R-HSA-110312	2/16	0.04767	0.269503	0	0	6.345455	19.31213
p75NTR signals via NF-kB Homo sapiens R-HSA-193639	2/16	0.04767	0.269503	0	0	6.345455	19.31213
Spry regulation of FGF signaling Homo sapiens R-HSA-1295596	2/16	0.04767	0.269503	0	0	6.345455	19.31213

MAP3K8 (TPL2)-dependent MAPK1/3 activation Homo sapiens R-HSA-5684264	2/16	0.04767	0.269503		0	0	6.345455	19.31213
G1 Phase Homo sapiens R-HSA-69236	3/38	0.05116	0.284749		0	0	3.811845	11.33187
Cyclin D associated events in G1 Homo sapiens R-HSA-69231	3/38	0.05116	0.284749		0	0	3.811845	11.33187
IRS-mediated signalling Homo sapiens R-HSA-112399	11/284	0.051789	0.285346		0	0	1.802903	5.337646
MAPK6/MAPK4 signaling Homo sapiens R-HSA-5687128	5/92	0.05323	0.285346		0	0	2.560693	7.510849
Unblocking of NMDA receptor. glutamate binding and activation Homo sapiens R-HSA-438066	2/17	0.053254	0.285346		0	0	5.922121	17.36771
Translesion synthesis by POLK Homo sapiens R-HSA-5655862	2/17	0.053254	0.285346		0	0	5.922121	17.36771
Translesion synthesis by POLI Homo sapiens R-HSA-5656121	2/17	0.053254	0.285346		0	0	5.922121	17.36771
Toll Like Receptor 4 (TLR4) Cascade Homo sapiens R-HSA-166016	6/122	0.053808	0.285837		0	0	2.306469	6.740253
Interleukin-2 signaling Homo sapiens R-HSA-451927	10/252	0.054216	0.285837		0	0	1.847643	5.385457
Insulin receptor signalling cascade Homo sapiens R-HSA-74751	11/287	0.055024	0.285837		0	0	1.783029	5.170746
Signalling to ERKs Homo sapiens R-HSA-187687	10/253	0.055393	0.285837		0	0	1.839944	5.323521
IGF1R signaling cascade Homo sapiens R-HSA-2428924	11/288	0.056132	0.285837		0	0	1.7765	5.116398
Signaling by Type 1 Insulin-like Growth Factor 1 Receptor (IGF1R) Homo sapiens R-HSA-2404192	11/288	0.056132	0.285837		0	0	1.7765	5.116398
IRS-related events triggered by IGF1R Homo sapiens R-HSA-2428928	11/288	0.056132	0.285837		0	0	1.7765	5.116398
FCERI mediated MAPK activation Homo sapiens R-HSA-2871796	11/289	0.057255	0.2895		0	0	1.770018	5.062678
PRC2 methylates histones and DNA Homo sapiens R-HSA-212300	3/40	0.058039	0.291414		0	0	3.60543	10.26334
Oxygen-dependent proline hydroxylation of Hypoxia-	2/18	0.059056	0.294462		0	0	5.551705	15.70723

inducible Factor Alpha Homo sapiens R-HSA-1234176								
Condensation of Prophase Chromosomes Homo sapiens R-HSA-2299718	3/41	0.061634	0.304548		0	0	3.51037	9.781775
Downstream signaling of activated FGFR2 Homo sapiens R-HSA-5654696	12/329	0.062776	0.304548		0	0	1.693874	4.688955
Downstream signaling of activated FGFR4 Homo sapiens R-HSA-5654716	12/329	0.062776	0.304548		0	0	1.693874	4.688955
Downstream signaling of activated FGFR3 Homo sapiens R-HSA-5654708	12/329	0.062776	0.304548		0	0	1.693874	4.688955
Signaling by ERBB4 Homo sapiens R-HSA-1236394	12/330	0.063912	0.30798		0	0	1.68846	4.643676
Regulation of TP53 Activity through Methylation Homo sapiens R-HSA-6804760	2/19	0.065065	0.308601		0	0	5.224866	14.27626
Translesion Synthesis by POLH Homo sapiens R-HSA-110320	2/19	0.065065	0.308601		0	0	5.224866	14.27626
Deactivation of the beta-catenin transactivating complex Homo sapiens R-HSA-3769402	3/42	0.065331	0.308601		0	0	3.420186	9.331275
Signaling by FGFR4 Homo sapiens R-HSA-5654743	12/332	0.066225	0.308766		0	0	1.677733	4.554524
Downstream signaling of activated FGFR1 Homo sapiens R-HSA-5654687	12/332	0.066225	0.308766		0	0	1.677733	4.554524
Signaling by FGFR3 Homo sapiens R-HSA-5654741	12/333	0.067402	0.312225		0	0	1.672419	4.510639
TP53 Regulates Transcription of Cell Death Genes Homo sapiens R-HSA-5633008	3/43	0.069126	0.316132		0	0	3.33451	8.909211
ERCC6 (CSB) and EHMT2 (G9a) positively regulate rRNA expression Homo sapiens R-HSA-427389	3/43	0.069126	0.316132		0	0	3.33451	8.909211
Signaling by FGFR1 Homo sapiens R-HSA-5654736	12/336	0.071015	0.321825		0	0	1.656675	4.381684
Regulation of gene expression in beta cells Homo sapiens R-HSA-210745	2/20	0.071268	0.321825		0	0	4.934343	13.03314
Transcriptional activity of SMAD2/SMAD3:SMAD4 heterotrimer Homo sapiens R-HSA-2173793	3/44	0.07302	0.32564		0	0	3.253014	8.513226
Interleukin-1 signaling Homo sapiens R-HSA-446652	3/44	0.07302	0.32564		0	0	3.253014	8.513226

Downstream signal transduction Homo sapiens R-HSA-186763	12/341	0.07731	0.337909	0	0	1.631074	4.175443
Regulation of FZD by ubiquitination Homo sapiens R-HSA-4641263	2/21	0.077653	0.337909	0	0	4.674402	11.94545
TP53 regulates transcription of additional cell cycle genes whose exact role in the p53 pathway remain uncertain Homo sapiens R-HSA-6804115	2/21	0.077653	0.337909	0	0	4.674402	11.94545
Aflatoxin activation and detoxification Homo sapiens R-HSA-5423646	2/21	0.077653	0.337909	0	0	4.674402	11.94545
Signaling by TGF-beta Receptor Complex Homo sapiens R-HSA-170834	4/73	0.07813	0.337934	0	0	2.579445	6.576004
DAP12 signaling Homo sapiens R-HSA-2424491	12/344	0.081252	0.349336	0	0	1.616083	4.056689
SALM protein interactions at the synapse Homo sapiens R-HSA-8849932	2/22	0.08421	0.355664	0	0	4.440455	10.98764
Pink/Parkin Mediated Mitophagy Homo sapiens R-HSA-5205685	2/22	0.08421	0.355664	0	0	4.440455	10.98764
Mitophagy Homo sapiens R-HSA-5205647	2/22	0.08421	0.355664	0	0	4.440455	10.98764
NOTCH1 Intracellular Domain Regulates Transcription Homo sapiens R-HSA-2122947	3/47	0.085266	0.358016	0	0	3.030752	7.461663
Signaling by Insulin receptor Homo sapiens R-HSA-74752	11/311	0.085773	0.358054	0	0	1.638345	4.023852
TP53 Regulates Transcription of Cell Cycle Genes Homo sapiens R-HSA-6791312	3/48	0.08953	0.36885	0	0	2.96325	7.150875
Toll-Like Receptors Cascades Homo sapiens R-HSA-168898	6/140	0.090682	0.36885	0	0	1.994797	4.788293
Gap-filling DNA repair synthesis and ligation in GG-NER Homo sapiens R-HSA-5696397	2/23	0.090928	0.36885	0	0	4.228788	10.13931
Downregulation of SMAD2/3:SMAD4 transcriptional activity Homo sapiens R-HSA-2173795	2/23	0.090928	0.36885	0	0	4.228788	10.13931
APC/C:Cdc20 mediated degradation of Cyclin B Homo sapiens R-HSA-174048	2/23	0.090928	0.36885	0	0	4.228788	10.13931
Signaling by EGFR Homo sapiens R-HSA-177929	12/355	0.096776	0.387945	0	0	1.56336	3.650999

RNA Polymerase I Transcription Homo sapiens R-HSA-73864	4/79	0.097602	0.387945	0	0	2.372359	5.52014
Glucose metabolism Homo sapiens R-HSA-70326	4/79	0.097602	0.387945	0	0	2.372359	5.52014
Endogenous sterols Homo sapiens R-HSA-211976	2/24	0.097797	0.387945	0	0	4.036364	9.383998
HDR through Homologous Recombination (HR) or Single Strand Annealing (SSA) Homo sapiens R-HSA-5693567	5/112	0.10271	0.390061	0	0	2.07992	4.73358
DAP12 interactions Homo sapiens R-HSA-2172127	12/359	0.102838	0.390061	0	0	1.545017	3.514289
Processing of DNA double-strand break ends Homo sapiens R-HSA-5693607	4/81	0.104552	0.390061	0	0	2.310502	5.217287
Constitutive Signaling by AKT1 E17K in Cancer Homo sapiens R-HSA-5674400	2/25	0.104806	0.390061	0	0	3.860672	8.708299
Budding and maturation of HIV virion Homo sapiens R-HSA-162588	2/25	0.104806	0.390061	0	0	3.860672	8.708299
APC-Cdc20 mediated degradation of Nek2A Homo sapiens R-HSA-179409	2/25	0.104806	0.390061	0	0	3.860672	8.708299
Abacavir metabolism Homo sapiens R-HSA-2161541	1/5	0.105732	0.390061	0	0	11.08503	24.90634
Na+-dependent glucose transporters Homo sapiens R-HSA-428808	1/5	0.105732	0.390061	0	0	11.08503	24.90634
The fatty acid cycling model Homo sapiens R-HSA-167826	1/5	0.105732	0.390061	0	0	11.08503	24.90634
The proton buffering model Homo sapiens R-HSA-167827	1/5	0.105732	0.390061	0	0	11.08503	24.90634
Mitochondrial Uncoupling Proteins Homo sapiens R-HSA-166187	1/5	0.105732	0.390061	0	0	11.08503	24.90634
Signaling by FGFR2 Homo sapiens R-HSA-5654738	12/361	0.105953	0.390061	0	0	1.536003	3.447961
Diseases of signal transduction Homo sapiens R-HSA-5663202	10/288	0.107359	0.390061	0	0	1.605382	3.582539
Deposition of new CENPA-containing nucleosomes at the centromere Homo sapiens R-HSA-606279	3/52	0.107439	0.390061	0	0	2.720794	6.069644
Nucleosome assembly Homo sapiens R-HSA-774815	3/52	0.107439	0.390061	0	0	2.720794	6.069644
Toll Like Receptor 10 (TLR10) Cascade Homo sapiens R-HSA-168142	4/82	0.108109	0.390061	0	0	2.280763	5.073824

Toll Like Receptor 5 (TLR5) Cascade Homo sapiens R-HSA-168176	4/82	0.108109	0.390061	0	0	2.280763	5.073824
MyD88 cascade initiated on plasma membrane Homo sapiens R-HSA-975871	4/82	0.108109	0.390061	0	0	2.280763	5.073824
Signaling by PDGF Homo sapiens R-HSA-186797	12/364	0.110728	0.397514	0	0	1.522674	3.350917
TRAF6 mediated induction of NFkB and MAP kinases upon TLR7/8 or 9 activation Homo sapiens R-HSA-975138	4/83	0.11172	0.399079	0	0	2.251777	4.935355
Signaling by FGFR Homo sapiens R-HSA-190236	12/366	0.11398	0.405138	0	0	1.513914	3.287811
TP53 Regulates Metabolic Genes Homo sapiens R-HSA-5628897	4/84	0.115384	0.408108	0	0	2.223516	4.801656
Meiotic recombination Homo sapiens R-HSA-912446	3/54	0.116874	0.409533	0	0	2.613828	5.610989
MyD88 dependent cascade initiated on endosome Homo sapiens R-HSA-975155	4/85	0.119101	0.409533	0	0	2.195952	4.67252
Toll Like Receptor 7/8 (TLR7/8) Cascade Homo sapiens R-HSA-168181	4/85	0.119101	0.409533	0	0	2.195952	4.67252
Uptake and actions of bacterial toxins Homo sapiens R-HSA-5339562	2/27	0.119209	0.409533	0	0	3.551455	7.553496
EGFR downregulation Homo sapiens R-HSA-182971	2/27	0.119209	0.409533	0	0	3.551455	7.553496
Synthesis of bile acids and bile salts Homo sapiens R-HSA-192105	2/27	0.119209	0.409533	0	0	3.551455	7.553496
Homology Directed Repair Homo sapiens R-HSA-5693538	5/118	0.120864	0.413241	0	0	1.968875	4.1604
Hh mutants that don't undergo autocatalytic processing are degraded by ERAD Homo sapiens R-HSA-5362768	3/55	0.121705	0.414142	0	0	2.563431	5.398989
Unfolded Protein Response (UPR) Homo sapiens R-HSA-381119	4/86	0.122868	0.414175	0	0	2.169061	4.547749
PI3K/AKT Signaling in Cancer Homo sapiens R-HSA-2219528	4/86	0.122868	0.414175	0	0	2.169061	4.547749
Recognition of DNA damage by PCNA-containing replication complex Homo sapiens R-HSA-110314	2/28	0.126585	0.419173	0	0	3.414685	7.057607

Endosomal Sorting Complex Required For Transport (ESCRT) Homo sapiens R-HSA-917729	2/28	0.126585	0.419173	0	0	3.414685	7.057607
Chemokine receptors bind chemokines Homo sapiens R-HSA-380108	3/56	0.126607	0.419173	0	0	2.514935	5.197534
Regulation of PLK1 Activity at G2/M Transition Homo sapiens R-HSA-2565942	4/87	0.126686	0.419173	0	0	2.142818	4.427156
NGF signalling via TRKA from the plasma membrane Homo sapiens R-HSA-187037	12/374	0.127537	0.420052	0	0	1.479841	3.047511
Toll Like Receptor 9 (TLR9) Cascade Homo sapiens R-HSA-168138	4/88	0.130553	0.425557	0	0	2.117199	4.310564
Meiotic synapsis Homo sapiens R-HSA-1221632	3/57	0.131579	0.425557	0	0	2.468236	5.005947
RNA Polymerase I Chain Elongation Homo sapiens R-HSA-73777	3/57	0.131579	0.425557	0	0	2.468236	5.005947
Hh mutants abrogate ligand secretion Homo sapiens R-HSA-5387390	3/57	0.131579	0.425557	0	0	2.468236	5.005947
Signaling by NOTCH Homo sapiens R-HSA-157118	5/122	0.133744	0.430232	0	0	1.901172	3.824827
Downstream signaling events of B Cell Receptor (BCR) Homo sapiens R-HSA-1168372	7/192	0.134223	0.430232	0	0	1.685132	3.384173
B-WICH complex positively regulates rRNA expression Homo sapiens R-HSA-5250924	3/58	0.136618	0.435964	0	0	2.423235	4.823604
Termination of translesion DNA synthesis Homo sapiens R-HSA-5656169	2/30	0.141643	0.446302	0	0	3.170455	6.196487
Telomere Maintenance Homo sapiens R-HSA-157579	3/59	0.141723	0.446302	0	0	2.379841	4.649929
SCF(Skp2)-mediated degradation of p27/p21 Homo sapiens R-HSA-187577	3/59	0.141723	0.446302	0	0	2.379841	4.649929
Inositol transporters Homo sapiens R-HSA-429593	1/7	0.144833	0.45213	0	0	7.389267	14.27736
PTK6 promotes HIF1A stabilization Homo sapiens R-HSA-8857538	1/7	0.144833	0.45213	0	0	7.389267	14.27736
Phase 1 - Functionalization of compounds Homo sapiens R-HSA-211945	4/92	0.146497	0.452648	0	0	2.020548	3.880973

RHO GTPases activate PKNs Homo sapiens R-HSA-5625740	3/60	0.14689	0.452648	0	0	2.337969	4.484392
MAP kinase activation in TLR cascade Homo sapiens R-HSA-450294	3/60	0.14689	0.452648	0	0	2.337969	4.484392
HS-GAG biosynthesis Homo sapiens R-HSA-2022928	2/31	0.149308	0.456184	0	0	3.060972	5.82118
Regulation of TNFR1 signaling Homo sapiens R-HSA-5357905	2/31	0.149308	0.456184	0	0	3.060972	5.82118
Biological oxidations Homo sapiens R-HSA-211859	7/199	0.15286	0.465058	0	0	1.623108	3.048572
Regulation of beta-cell development Homo sapiens R-HSA-186712	2/32	0.157055	0.466525	0	0	2.958788	5.477192
EPH-Ephrin signaling Homo sapiens R-HSA-2682334	4/95	0.158925	0.466525	0	0	1.953635	3.593372
Signaling by Interleukins Homo sapiens R-HSA-449147	12/392	0.16115	0.466525	0	0	1.408421	2.57096
Circadian Clock Homo sapiens R-HSA-400253	3/63	0.162745	0.466525	0	0	2.220729	4.031885
p53-Dependent G1/S DNA damage checkpoint Homo sapiens R-HSA-69580	3/63	0.162745	0.466525	0	0	2.220729	4.031885
p53-Dependent G1 DNA Damage Response Homo sapiens R-HSA-69563	3/63	0.162745	0.466525	0	0	2.220729	4.031885
Asymmetric localization of PCP proteins Homo sapiens R-HSA-4608870	3/63	0.162745	0.466525	0	0	2.220729	4.031885
Gastrin-CREB signalling pathway via PKC and MAPK Homo sapiens R-HSA-881907	13/432	0.162904	0.466525	0	0	1.384176	2.511714
Erythrocytes take up oxygen and release carbon dioxide Homo sapiens R-HSA-1247673	1/8	0.163739	0.466525	0	0	6.333333	11.46006
Sema4D mediated inhibition of cell attachment and migration Homo sapiens R-HSA-416550	1/8	0.163739	0.466525	0	0	6.333333	11.46006
Terminal pathway of complement Homo sapiens R-HSA-166665	1/8	0.163739	0.466525	0	0	6.333333	11.46006
Release of Hh-Np from the secreting cell Homo sapiens R-HSA-5362798	1/8	0.163739	0.466525	0	0	6.333333	11.46006
Ligand-receptor interactions Homo sapiens R-HSA-5632681	1/8	0.163739	0.466525	0	0	6.333333	11.46006

AMPK inhibits chREBP transcriptional activation activity Homo sapiens R-HSA-163680	1/8	0.163739	0.466525		0	0	6.333333	11.46006
NR1D1 (REV-ERBA) represses gene expression Homo sapiens R-HSA-1368071	1/8	0.163739	0.466525		0	0	6.333333	11.46006
Synthesis of epoxy (EET) and dihydroxyeicosatrienoic acids (DHET) Homo sapiens R-HSA-2142670	1/8	0.163739	0.466525		0	0	6.333333	11.46006
Visual phototransduction Homo sapiens R-HSA-2187338	4/97	0.167418	0.467927		0	0	1.911425	3.416212
MyD88-independent TLR3/TLR4 cascade Homo sapiens R-HSA-166166	4/97	0.167418	0.467927		0	0	1.911425	3.416212
Toll Like Receptor 3 (TLR3) Cascade Homo sapiens R-HSA-168164	4/97	0.167418	0.467927		0	0	1.911425	3.416212
TRIF-mediated TLR3/TLR4 signaling Homo sapiens R-HSA-937061	4/97	0.167418	0.467927		0	0	1.911425	3.416212
HDR through Homologous Recombination (HRR) Homo sapiens R-HSA-5685942	3/64	0.168141	0.467927		0	0	2.184212	3.894347
Hedgehog ligand biogenesis Homo sapiens R-HSA-5358346	3/64	0.168141	0.467927		0	0	2.184212	3.894347
EPHA-mediated growth cone collapse Homo sapiens R-HSA-3928663	2/34	0.172762	0.477089		0	0	2.77358	4.869965
Negative regulators of RIG-I/MDA5 signaling Homo sapiens R-HSA-936440	2/34	0.172762	0.477089		0	0	2.77358	4.869965
G1/S DNA Damage Checkpoints Homo sapiens R-HSA-69615	3/65	0.173588	0.477533		0	0	2.148872	3.762829
Activation of NF-kappaB in B cells Homo sapiens R-HSA-1169091	3/66	0.179084	0.479265		0	0	2.114655	3.636997
Lysosome Vesicle Biogenesis Homo sapiens R-HSA-432720	2/35	0.180709	0.479265		0	0	2.689394	4.601201
Bile acid and bile salt metabolism Homo sapiens R-HSA-194068	2/35	0.180709	0.479265		0	0	2.689394	4.601201
Regulation of TP53 Degradation Homo sapiens R-HSA-6804757	2/35	0.180709	0.479265		0	0	2.689394	4.601201
Sperm Motility And Taxes Homo sapiens R-HSA-1300642	1/9	0.182227	0.479265		0	0	5.541383	9.434205

CHL1 interactions Homo sapiens R-HSA-447041	1/9	0.182227	0.479265	0	0	5.541383	9.434205
Ca2+ activated K+ channels Homo sapiens R-HSA-1296052	1/9	0.182227	0.479265	0	0	5.541383	9.434205
Prostanoid ligand receptors Homo sapiens R-HSA-391908	1/9	0.182227	0.479265	0	0	5.541383	9.434205
AKT phosphorylates targets in the nucleus Homo sapiens R-HSA-198693	1/9	0.182227	0.479265	0	0	5.541383	9.434205
Synthesis of (16-20)-hydroxyeicosatetraenoic acids (HETE) Homo sapiens R-HSA-2142816	1/9	0.182227	0.479265	0	0	5.541383	9.434205
Highly calcium permeable nicotinic acetylcholine receptors Homo sapiens R-HSA-629597	1/9	0.182227	0.479265	0	0	5.541383	9.434205
EGFR Transactivation by Gastrin Homo sapiens R-HSA-2179392	1/9	0.182227	0.479265	0	0	5.541383	9.434205
Semaphorin interactions Homo sapiens R-HSA-373755	3/67	0.184627	0.480928	0	0	2.081506	3.516535
Signaling by PTK6 Homo sapiens R-HSA-8848021	3/67	0.184627	0.480928	0	0	2.081506	3.516535
Gene Silencing by RNA Homo sapiens R-HSA-211000	4/101	0.184869	0.480928	0	0	1.832227	3.092996
Regulation of TP53 Expression and Degradation Homo sapiens R-HSA-6806003	2/36	0.188709	0.488811	0	0	2.61016	4.352572
Cyclin E associated events during G1/S transition Homo sapiens R-HSA-69202	3/68	0.190215	0.488811	0	0	2.049378	3.401153
Cyclin A:Cdk2-associated events at S phase entry Homo sapiens R-HSA-69656	3/69	0.195845	0.488811	0	0	2.018223	3.290577
NCAM1 interactions Homo sapiens R-HSA-419037	2/37	0.196756	0.488811	0	0	2.535455	4.122114
Translesion synthesis by Y family DNA polymerases bypasses lesions on DNA template Homo sapiens R-HSA-110313	2/37	0.196756	0.488811	0	0	2.535455	4.122114
Abacavir transport and metabolism Homo sapiens R-HSA-2161522	1/10	0.200308	0.488811	0	0	4.925422	7.919575
eNOS activation Homo sapiens R-HSA-203615	1/10	0.200308	0.488811	0	0	4.925422	7.919575
Tetrahydrobiopterin (BH4) synthesis. recycling. salvage and regulation Homo sapiens R-HSA-1474151	1/10	0.200308	0.488811	0	0	4.925422	7.919575

ATF6-alpha activates chaperone genes Homo sapiens R-HSA-381183	1/10	0.200308	0.488811	0	0	4.925422	7.919575
Regulation of signaling by NODAL Homo sapiens R-HSA-1433617	1/10	0.200308	0.488811	0	0	4.925422	7.919575
Glycoprotein hormones Homo sapiens R-HSA-209822	1/10	0.200308	0.488811	0	0	4.925422	7.919575
SLBP independent Processing of Histone Pre-mRNAs Homo sapiens R-HSA-111367	1/10	0.200308	0.488811	0	0	4.925422	7.919575
Regulation of gene expression by Hypoxia-inducible Factor Homo sapiens R-HSA-1234158	1/10	0.200308	0.488811	0	0	4.925422	7.919575
Signaling by FGFR3 fusions in cancer Homo sapiens R-HSA-8853334	1/10	0.200308	0.488811	0	0	4.925422	7.919575
Activation of the AP-1 family of transcription factors Homo sapiens R-HSA-450341	1/10	0.200308	0.488811	0	0	4.925422	7.919575
IRAK1 recruits IKK complex Homo sapiens R-HSA-937039	1/10	0.200308	0.488811	0	0	4.925422	7.919575
IRAK1 recruits IKK complex upon TLR7/8 or 9 stimulation Homo sapiens R-HSA-975144	1/10	0.200308	0.488811	0	0	4.925422	7.919575
TRIF-mediated programmed cell death Homo sapiens R-HSA-2562578	1/10	0.200308	0.488811	0	0	4.925422	7.919575
Switching of origins to a post-replicative state Homo sapiens R-HSA-69052	3/70	0.201515	0.488811	0	0	1.987999	3.184554
Orc1 removal from chromatin Homo sapiens R-HSA-68949	3/70	0.201515	0.488811	0	0	1.987999	3.184554
DNA Replication Homo sapiens R-HSA-69306	4/105	0.20288	0.490464	0	0	1.759302	2.806334
DNA Damage Recognition in GG-NER Homo sapiens R-HSA-5696394	2/38	0.204845	0.493553	0	0	2.464899	3.9081
Transcriptional regulation by small RNAs Homo sapiens R-HSA-5578749	3/72	0.212969	0.495461	0	0	1.930177	2.985228
ISG15 antiviral mechanism Homo sapiens R-HSA-1169408	3/72	0.212969	0.495461	0	0	1.930177	2.985228
Antiviral mechanism by IFN-stimulated genes Homo sapiens R-HSA-1169410	3/72	0.212969	0.495461	0	0	1.930177	2.985228
Removal of licensing factors from origins Homo sapiens R-HSA-69300	3/72	0.212969	0.495461	0	0	1.930177	2.985228

TRAF6 Mediated Induction of proinflammatory cytokines Homo sapiens R-HSA-168180	3/72	0.212969	0.495461	0	0	1.930177	2.985228
Activation of NMDA receptor upon glutamate binding and postsynaptic events Homo sapiens R-HSA-442755	2/39	0.212969	0.495461	0	0	2.398157	3.709007
Dual Incision in GG-NER Homo sapiens R-HSA-5696400	2/39	0.212969	0.495461	0	0	2.398157	3.709007
Cytokine Signaling in Immune system Homo sapiens R-HSA-1280215	17/620	0.213667	0.495461	0	0	1.25738	1.940558
Import of palmitoyl-CoA into the mitochondrial matrix Homo sapiens R-HSA-200425	1/11	0.21799	0.495461	0	0	4.432653	6.752282
SLBP Dependent Processing of Replication-Dependent Histone Pre-mRNAs Homo sapiens R-HSA-77588	1/11	0.21799	0.495461	0	0	4.432653	6.752282
Highly calcium permeable postsynaptic nicotinic acetylcholine receptors Homo sapiens R-HSA-629594	1/11	0.21799	0.495461	0	0	4.432653	6.752282
Response to metal ions Homo sapiens R-HSA-5660526	1/11	0.21799	0.495461	0	0	4.432653	6.752282
Metallothioneins bind metals Homo sapiens R-HSA-5661231	1/11	0.21799	0.495461	0	0	4.432653	6.752282
Signaling by FGFR4 in disease Homo sapiens R-HSA-5655291	1/11	0.21799	0.495461	0	0	4.432653	6.752282
MyD88 deficiency (TLR2/4) Homo sapiens R-HSA-5602498	1/11	0.21799	0.495461	0	0	4.432653	6.752282
IRAK4 deficiency (TLR2/4) Homo sapiens R-HSA-5603041	1/11	0.21799	0.495461	0	0	4.432653	6.752282
DNA Double-Strand Break Repair Homo sapiens R-HSA-5693532	5/145	0.218051	0.495461	0	0	1.586957	2.416975
Positive epigenetic regulation of rRNA expression Homo sapiens R-HSA-5250913	3/73	0.218748	0.495461	0	0	1.902506	2.891495
NoRC negatively regulates rRNA expression Homo sapiens R-HSA-427413	3/73	0.218748	0.495461	0	0	1.902506	2.891495
TNF signaling Homo sapiens R-HSA-75893	2/41	0.229302	0.507517	0	0	2.274942	3.350337
Regulation of DNA replication Homo sapiens R-HSA-69304	3/75	0.230399	0.507517	0	0	1.849468	2.714912

Erythrocytes take up carbon dioxide and release oxygen Homo sapiens R-HSA-1237044	1/12	0.235282	0.507517		0	0	4.029478	5.830533
O2/CO2 exchange in erythrocytes Homo sapiens R-HSA-1480926	1/12	0.235282	0.507517		0	0	4.029478	5.830533
Reversible hydration of carbon dioxide Homo sapiens R-HSA-1475029	1/12	0.235282	0.507517		0	0	4.029478	5.830533
Defective EXT2 causes exostoses 2 Homo sapiens R-HSA-3656237	1/12	0.235282	0.507517		0	0	4.029478	5.830533
Defective EXT1 causes exostoses 1. TRPS2 and CHDS Homo sapiens R-HSA-3656253	1/12	0.235282	0.507517		0	0	4.029478	5.830533
ATF6-alpha activates chaperones Homo sapiens R-HSA-381033	1/12	0.235282	0.507517		0	0	4.029478	5.830533
TP53 Regulates Transcription of Death Receptors and Ligands Homo sapiens R-HSA-6803211	1/12	0.235282	0.507517		0	0	4.029478	5.830533
The NLRP3 inflammasome Homo sapiens R-HSA-844456	1/12	0.235282	0.507517		0	0	4.029478	5.830533
Peptide hormone biosynthesis Homo sapiens R-HSA-209952	1/12	0.235282	0.507517		0	0	4.029478	5.830533
Presynaptic nicotinic acetylcholine receptors Homo sapiens R-HSA-622323	1/12	0.235282	0.507517		0	0	4.029478	5.830533
G1/S Transition Homo sapiens R-HSA-69206	4/112	0.23554	0.507517		0	0	1.644681	2.378
Negative epigenetic regulation of rRNA expression Homo sapiens R-HSA-5250941	3/76	0.236268	0.507517		0	0	1.82404	2.631707
RNA Polymerase I Promoter Clearance Homo sapiens R-HSA-73854	3/76	0.236268	0.507517		0	0	1.82404	2.631707
Netrin-1 signaling Homo sapiens R-HSA-373752	2/42	0.237501	0.507517		0	0	2.217955	3.188494
EPHB-mediated forward signaling Homo sapiens R-HSA-3928662	2/42	0.237501	0.507517		0	0	2.217955	3.188494
Retinoid metabolism and transport Homo sapiens R-HSA-975634	2/42	0.237501	0.507517		0	0	2.217955	3.188494
Transcriptional regulation of pluripotent stem cells Homo sapiens R-HSA-452723	2/42	0.237501	0.507517		0	0	2.217955	3.188494

Formation of Incision Complex in GG-NER Homo sapiens R-HSA-5696395	2/43	0.245714	0.523 51	0	0	2.163747	3.0370 04
Innate Immune System Homo sapiens R-HSA-168249	21/807	0.250714	0.524 853	0	0	1.191311	1.6481 08
Facilitative Na+-independent glucose transporters Homo sapiens R-HSA-428790	1/13	0.252193	0.524 853	0	0	3.6935	5.0880 26
Cytosolic iron-sulfur cluster assembly Homo sapiens R-HSA-2564830	1/13	0.252193	0.524 853	0	0	3.6935	5.0880 26
Synthesis. secretion. and inactivation of Glucose-dependent Insulinotropic Polypeptide (GIP) Homo sapiens R-HSA-400511	1/13	0.252193	0.524 853	0	0	3.6935	5.0880 26
TP53 Regulates Transcription of Genes Involved in G1 Cell Cycle Arrest Homo sapiens R-HSA-6804116	1/13	0.252193	0.524 853	0	0	3.6935	5.0880 26
Signaling by Activin Homo sapiens R-HSA-1502540	1/13	0.252193	0.524 853	0	0	3.6935	5.0880 26
AKT phosphorylates targets in the cytosol Homo sapiens R-HSA-198323	1/13	0.252193	0.524 853	0	0	3.6935	5.0880 26
ERBB2 Activates PTK6 Signaling Homo sapiens R-HSA-8847993	1/13	0.252193	0.524 853	0	0	3.6935	5.0880 26
Intra-Golgi traffic Homo sapiens R-HSA-6811438	2/44	0.253938	0.526 958	0	0	2.112121	2.8950 11
RNA Polymerase I. RNA Polymerase III. and Mitochondrial Transcription Homo sapiens R-HSA-504046	4/116	0.254738	0.527 094	0	0	1.585616	2.1683 63
Signaling by the B Cell Receptor (BCR) Homo sapiens R-HSA-983705	7/233	0.257735	0.531 763	0	0	1.376503	1.8662 95
Diseases of metabolism Homo sapiens R-HSA-5668914	2/45	0.262167	0.533 006	0	0	2.062896	2.7617 49
Interferon Signaling Homo sapiens R-HSA-913531	6/196	0.266765	0.533 006	0	0	1.402801	1.8536 45
SEMA3A-Plexin repulsion signaling by inhibiting Integrin adhesion Homo sapiens R-HSA-399955	1/14	0.26873	0.533 006	0	0	3.40921	4.4798 68
Repression of WNT target genes Homo sapiens R-HSA-4641265	1/14	0.26873	0.533 006	0	0	3.40921	4.4798 68
Early Phase of HIV Life Cycle Homo sapiens R-HSA-162594	1/14	0.26873	0.533 006	0	0	3.40921	4.4798 68

Regulation of TP53 Activity through Association with Co-factors Homo sapiens R-HSA-6804759	1/14	0.26873	0.533006	0	0	3.40921	4.479868
TP53 regulates transcription of several additional cell death genes whose specific roles in p53-dependent apoptosis remain uncertain Homo sapiens R-HSA-6803205	1/14	0.26873	0.533006	0	0	3.40921	4.479868
Synthesis of bile acids and bile salts via 24-hydroxycholesterol Homo sapiens R-HSA-193775	1/14	0.26873	0.533006	0	0	3.40921	4.479868
Glutathione synthesis and recycling Homo sapiens R-HSA-174403	1/14	0.26873	0.533006	0	0	3.40921	4.479868
Regulation of pyruvate dehydrogenase (PDH) complex Homo sapiens R-HSA-204174	1/14	0.26873	0.533006	0	0	3.40921	4.479868
Activation of Nicotinic Acetylcholine Receptors Homo sapiens R-HSA-629602	1/14	0.26873	0.533006	0	0	3.40921	4.479868
Postsynaptic nicotinic acetylcholine receptors Homo sapiens R-HSA-622327	1/14	0.26873	0.533006	0	0	3.40921	4.479868
Acetylcholine Binding And Downstream Events Homo sapiens R-HSA-181431	1/14	0.26873	0.533006	0	0	3.40921	4.479868
Regulation of IFNG signaling Homo sapiens R-HSA-877312	1/14	0.26873	0.533006	0	0	3.40921	4.479868
DNA Damage Bypass Homo sapiens R-HSA-73893	2/46	0.270398	0.533501	0	0	2.015909	2.636527
Degradation of beta-catenin by the destruction complex Homo sapiens R-HSA-195253	3/82	0.271952	0.533501	0	0	1.684986	2.194072
DNA Replication Pre-Initiation Homo sapiens R-HSA-69002	3/82	0.271952	0.533501	0	0	1.684986	2.194072
M/G1 Transition Homo sapiens R-HSA-68874	3/82	0.271952	0.533501	0	0	1.684986	2.194072
TCF dependent signaling in response to WNT Homo sapiens R-HSA-201681	6/199	0.27805	0.537746	0	0	1.380781	1.767335
PI-3K cascade:FGFR1 Homo sapiens R-HSA-5654689	4/122	0.284084	0.537746	0	0	1.504528	1.893426
PI-3K cascade:FGFR3 Homo sapiens R-HSA-5654710	4/122	0.284084	0.537746	0	0	1.504528	1.893426
PI3K events in ERBB4 signaling Homo sapiens R-HSA-1250342	4/122	0.284084	0.537746	0	0	1.504528	1.893426

PIP3 activates AKT signaling Homo sapiens R-HSA-1257604	4/122	0.284084	0.537746	0	0	1.504528	1.893426
PI-3K cascade:FGFR4 Homo sapiens R-HSA-5654720	4/122	0.284084	0.537746	0	0	1.504528	1.893426
PI-3K cascade:FGFR2 Homo sapiens R-HSA-5654695	4/122	0.284084	0.537746	0	0	1.504528	1.893426
CREB phosphorylation through the activation of CaMKII Homo sapiens R-HSA-442729	1/15	0.284902	0.537746	0	0	3.165533	3.974672
Eicosanoid ligand-binding receptors Homo sapiens R-HSA-391903	1/15	0.284902	0.537746	0	0	3.165533	3.974672
Recycling of bile acids and salts Homo sapiens R-HSA-159418	1/15	0.284902	0.537746	0	0	3.165533	3.974672
Glycogen breakdown (glycogenolysis) Homo sapiens R-HSA-70221	1/15	0.284902	0.537746	0	0	3.165533	3.974672
ERBB2 Regulates Cell Motility Homo sapiens R-HSA-6785631	1/15	0.284902	0.537746	0	0	3.165533	3.974672
Signaling by EGFRvIII in Cancer Homo sapiens R-HSA-5637812	1/15	0.284902	0.537746	0	0	3.165533	3.974672
Constitutive Signaling by EGFRvIII Homo sapiens R-HSA-5637810	1/15	0.284902	0.537746	0	0	3.165533	3.974672
Metabolism of carbohydrates Homo sapiens R-HSA-71387	8/282	0.286513	0.537746	0	0	1.297319	1.621614
Hexose transport Homo sapiens R-HSA-189200	2/48	0.286848	0.537746	0	0	1.928063	2.407773
Death Receptor Signalling Homo sapiens R-HSA-73887	2/48	0.286848	0.537746	0	0	1.928063	2.407773
Meiosis Homo sapiens R-HSA-1500620	3/85	0.290014	0.540857	0	0	1.62309	2.009103
Hedgehog 'on' state Homo sapiens R-HSA-5632684	3/85	0.290014	0.540857	0	0	1.62309	2.009103
Signalling by NGF Homo sapiens R-HSA-166520	12/450	0.294229	0.54571	0	0	1.218222	1.490371
Mitochondrial biogenesis Homo sapiens R-HSA-1592230	2/49	0.295059	0.54571	0	0	1.886944	2.303166
Regulation of activated PAK-2p34 by proteasome mediated degradation Homo sapiens R-HSA-211733	2/49	0.295059	0.54571	0	0	1.886944	2.303166
Chromosome Maintenance Homo sapiens R-HSA-73886	3/86	0.296055	0.54571	0	0	1.603453	1.951736
GAB1 signalosome Homo sapiens R-HSA-180292	4/125	0.298945	0.54571	0	0	1.466999	1.771395

PI3K/AKT activation Homo sapiens R-HSA-198203	4/125	0.298945	0.545 71		0	0	1.466999	1.7713 95
Regulation of KIT signaling Homo sapiens R-HSA-1433559	1/16	0.300718	0.545 71		0	0	2.954346	3.5498 93
Sema3A PAK dependent Axon repulsion Homo sapiens R-HSA-399954	1/16	0.300718	0.545 71		0	0	2.954346	3.5498 93
PI3K events in ERBB2 signaling Homo sapiens R-HSA-1963642	1/16	0.300718	0.545 71		0	0	2.954346	3.5498 93
GRB2 events in ERBB2 signaling Homo sapiens R-HSA-1963640	1/16	0.300718	0.545 71		0	0	2.954346	3.5498 93
CDK-mediated phosphorylation and removal of Cdc6 Homo sapiens R-HSA-69017	2/50	0.303257	0.545 71		0	0	1.847538	2.2044 38
Ubiquitin-dependent degradation of Cyclin D1 Homo sapiens R-HSA-69229	2/50	0.303257	0.545 71		0	0	1.847538	2.2044 38
Ubiquitin-dependent degradation of Cyclin D Homo sapiens R-HSA-75815	2/50	0.303257	0.545 71		0	0	1.847538	2.2044 38
Regulation of Apoptosis Homo sapiens R-HSA-169911	2/50	0.303257	0.545 71		0	0	1.847538	2.2044 38
Diseases of glycosylation Homo sapiens R-HSA-3781865	3/88	0.308159	0.548 358		0	0	1.565563	1.8428 86
Neurotransmitter Release Cycle Homo sapiens R-HSA-112310	2/51	0.311437	0.548 358		0	0	1.80974	2.1111 68
Metabolism of fat-soluble vitamins Homo sapiens R-HSA-6806667	2/51	0.311437	0.548 358		0	0	1.80974	2.1111 68
Nuclear Receptor transcription pathway Homo sapiens R-HSA-383280	2/51	0.311437	0.548 358		0	0	1.80974	2.1111 68
Vpu mediated degradation of CD4 Homo sapiens R-HSA-180534	2/51	0.311437	0.548 358		0	0	1.80974	2.1111 68
Autodegradation of the E3 ubiquitin ligase COP1 Homo sapiens R-HSA-349425	2/51	0.311437	0.548 358		0	0	1.80974	2.1111 68
Regulation of TP53 Activity through Phosphorylation Homo sapiens R-HSA-6804756	3/89	0.314218	0.548 358		0	0	1.54728	1.7912 36
Acetylcholine Neurotransmitter Release Cycle Homo sapiens R-HSA-264642	1/17	0.316184	0.548 358		0	0	2.769558	3.1889 53

Ras activation uopn Ca2+ infux through NMDA receptor Homo sapiens R-HSA-442982	1/17	0.316184	0.548358	0	0	2.769558	3.188953
TNF receptor superfamily (TNFSF) members mediating non-canonical NF-kB pathway Homo sapiens R-HSA-5676594	1/17	0.316184	0.548358	0	0	2.769558	3.188953
Trafficking of GluR2-containing AMPA receptors Homo sapiens R-HSA-416993	1/17	0.316184	0.548358	0	0	2.769558	3.188953
Tristetraprolin (TTP. ZFP36) binds and destabilizes mRNA Homo sapiens R-HSA-450513	1/17	0.316184	0.548358	0	0	2.769558	3.188953
Inflammasomes Homo sapiens R-HSA-622312	1/17	0.316184	0.548358	0	0	2.769558	3.188953
Formation of Senescence-Associated Heterochromatin Foci (SAHF) Homo sapiens R-HSA-2559584	1/17	0.316184	0.548358	0	0	2.769558	3.188953
Ligand-dependent caspase activation Homo sapiens R-HSA-140534	1/17	0.316184	0.548358	0	0	2.769558	3.188953
Nonhomologous End-Joining (NHEJ) Homo sapiens R-HSA-5693571	2/52	0.319597	0.548973	0	0	1.773455	2.02297
p53-Independent DNA Damage Response Homo sapiens R-HSA-69610	2/52	0.319597	0.548973	0	0	1.773455	2.02297
p53-Independent G1/S DNA damage checkpoint Homo sapiens R-HSA-69613	2/52	0.319597	0.548973	0	0	1.773455	2.02297
Ubiquitin Mediated Degradation of Phosphorylated Cdc25A Homo sapiens R-HSA-69601	2/52	0.319597	0.548973	0	0	1.773455	2.02297
Transmission across Chemical Synapses Homo sapiens R-HSA-112315	6/211	0.324154	0.554499	0	0	1.29915	1.46354
PCP/CE pathway Homo sapiens R-HSA-4086400	3/91	0.326342	0.554499	0	0	1.511959	1.693104
Formation of TC-NER Pre-Incision Complex Homo sapiens R-HSA-6781823	2/53	0.327733	0.554499	0	0	1.738592	1.939495
Vif-mediated degradation of APOBEC3G Homo sapiens R-HSA-180585	2/53	0.327733	0.554499	0	0	1.738592	1.939495
Signaling by Wnt Homo sapiens R-HSA-195721	8/295	0.32874	0.554499	0	0	1.237721	1.376948
Serotonin Neurotransmitter Release Cycle Homo sapiens R-HSA-181429	1/18	0.331309	0.554499	0	0	2.606509	2.879418

Norepinephrine Neurotransmitter Release Cycle Homo sapiens R-HSA-181430	1/18	0.331309	0.554499	0	0	2.606509	2.879418
Defective GALNT12 causes colorectal cancer 1 (CRCS1) Homo sapiens R-HSA-5083636	1/18	0.331309	0.554499	0	0	2.606509	2.879418
Defective GALNT3 causes familial hyperphosphatemic tumoral calcinosis (HFTC) Homo sapiens R-HSA-5083625	1/18	0.331309	0.554499	0	0	2.606509	2.879418
Activation of SMO Homo sapiens R-HSA-5635838	1/18	0.331309	0.554499	0	0	2.606509	2.879418
SHC1 events in ERBB2 signaling Homo sapiens R-HSA-1250196	1/18	0.331309	0.554499	0	0	2.606509	2.879418
Heparan sulfate/heparin (HS-GAG) metabolism Homo sapiens R-HSA-1638091	2/54	0.335843	0.554792	0	0	1.70507	1.86042
Golgi Associated Vesicle Biogenesis Homo sapiens R-HSA-432722	2/54	0.335843	0.554792	0	0	1.70507	1.86042
SCF-beta-TrCP mediated degradation of Emi1 Homo sapiens R-HSA-174113	2/54	0.335843	0.554792	0	0	1.70507	1.86042
Stabilization of p53 Homo sapiens R-HSA-69541	2/54	0.335843	0.554792	0	0	1.70507	1.86042
Degradation of AXIN Homo sapiens R-HSA-4641257	2/54	0.335843	0.554792	0	0	1.70507	1.86042
G2/M Transition Homo sapiens R-HSA-69275	5/173	0.33612	0.554792	0	0	1.320557	1.439785
Mitotic G2-G2/M phases Homo sapiens R-HSA-453274	5/175	0.344861	0.558428	0	0	1.304886	1.3892
GABA synthesis. release. reuptake and degradation Homo sapiens R-HSA-888590	1/19	0.346101	0.558428	0	0	2.461577	2.611796
Other semaphorin interactions Homo sapiens R-HSA-416700	1/19	0.346101	0.558428	0	0	2.461577	2.611796
Ephrin signaling Homo sapiens R-HSA-3928664	1/19	0.346101	0.558428	0	0	2.461577	2.611796
Defective B3GALT6 causes EDSP2 and SEMDJL1 Homo sapiens R-HSA-4420332	1/19	0.346101	0.558428	0	0	2.461577	2.611796
Defective B4GALT7 causes EDS. progeroid type Homo sapiens R-HSA-3560783	1/19	0.346101	0.558428	0	0	2.461577	2.611796
Defective B3GAT3 causes JDSSDHD Homo sapiens R-HSA-3560801	1/19	0.346101	0.558428	0	0	2.461577	2.611796

TP53 Regulates Transcription of Genes Involved in Cytochrome C Release Homo sapiens R-HSA-6803204	1/19	0.346101	0.558428	0	0	2.461577	2.611796
Signaling by NODAL Homo sapiens R-HSA-1181150	1/19	0.346101	0.558428	0	0	2.461577	2.611796
Phase 4 - resting membrane potential Homo sapiens R-HSA-5576886	1/19	0.346101	0.558428	0	0	2.461577	2.611796
TNFR2 non-canonical NF-kB pathway Homo sapiens R-HSA-5668541	3/95	0.350574	0.564377	0	0	1.445925	1.515595
Degradation of DVL Homo sapiens R-HSA-4641258	2/56	0.351973	0.565361	0	0	1.641751	1.714319
Mitotic G1-G1/S phases Homo sapiens R-HSA-453279	4/136	0.354013	0.567369	0	0	1.343988	1.395626
Defective C1GALT1C1 causes Tn polyagglutination syndrome (TNPS) Homo sapiens R-HSA-5083632	1/20	0.360566	0.574027	0	0	2.331901	2.378729
eNOS activation and regulation Homo sapiens R-HSA-203765	1/20	0.360566	0.574027	0	0	2.331901	2.378729
Metabolism of nitric oxide Homo sapiens R-HSA-202131	1/20	0.360566	0.574027	0	0	2.331901	2.378729
Synthesis of DNA Homo sapiens R-HSA-69239	3/97	0.362658	0.576081	0	0	1.415015	1.435242
NIK-->noncanonical NF-kB signaling Homo sapiens R-HSA-5676590	2/58	0.367965	0.581935	0	0	1.582955	1.582588
CDT1 association with the CDC6:ORC:origin complex Homo sapiens R-HSA-68827	2/58	0.367965	0.581935	0	0	1.582955	1.582588
CLEC7A (Dectin-1) signaling Homo sapiens R-HSA-5607764	3/99	0.374707	0.585463	0	0	1.385393	1.359917
Downstream TCR signaling Homo sapiens R-HSA-202424	3/99	0.374707	0.585463	0	0	1.385393	1.359917
HS-GAG degradation Homo sapiens R-HSA-2024096	1/21	0.374711	0.585463	0	0	2.215193	2.174432
Cell Cycle Checkpoints Homo sapiens R-HSA-69620	5/182	0.375538	0.585463	0	0	1.252828	1.227013
Degradation of GLI1 by the proteasome Homo sapiens R-HSA-5610780	2/59	0.375903	0.585463	0	0	1.555104	1.521552
GLI3 is processed to GLI3R by the proteasome Homo sapiens R-HSA-5610785	2/59	0.375903	0.585463	0	0	1.555104	1.521552
Degradation of GLI2 by the proteasome Homo sapiens R-HSA-5610783	2/59	0.375903	0.585463	0	0	1.555104	1.521552

Neurotransmitter Receptor Binding And Downstream Transmission In The Postsynaptic Cell Homo sapiens R-HSA-112314	4/142	0.384128	0.596978	0	0	1.285157	1.229611
Signaling by FGFR3 point mutants in cancer Homo sapiens R-HSA-8853338	1/22	0.388545	0.601239	0	0	2.109599	1.994304
Signaling by FGFR3 in disease Homo sapiens R-HSA-5655332	1/22	0.388545	0.601239	0	0	2.109599	1.994304
Cell death signalling via NRAGE. NRIF and NADE Homo sapiens R-HSA-204998	2/61	0.391653	0.602157	0	0	1.502234	1.408161
Constitutive Signaling by Aberrant PI3K in Cancer Homo sapiens R-HSA-2219530	2/61	0.391653	0.602157	0	0	1.502234	1.408161
Dectin-1 mediated noncanonical NF-kB signaling Homo sapiens R-HSA-5607761	2/61	0.391653	0.602157	0	0	1.502234	1.408161
Diseases associated with O-glycosylation of proteins Homo sapiens R-HSA-3906995	2/62	0.399462	0.60395	0	0	1.477121	1.355461
Cytochrome P450 - arranged by substrate type Homo sapiens R-HSA-211897	2/62	0.399462	0.60395	0	0	1.477121	1.355461
Gap-filling DNA repair synthesis and ligation in TC-NER Homo sapiens R-HSA-6782210	2/62	0.399462	0.60395	0	0	1.477121	1.355461
Autodegradation of Cdh1 by Cdh1:APC/C Homo sapiens R-HSA-174084	2/62	0.399462	0.60395	0	0	1.477121	1.355461
Dopamine Neurotransmitter Release Cycle Homo sapiens R-HSA-212676	1/23	0.402073	0.60395	0	0	2.013605	1.834641
The canonical retinoid cycle in rods (twilight vision) Homo sapiens R-HSA-2453902	1/23	0.402073	0.60395	0	0	2.013605	1.834641
Incretin synthesis. secretion. and inactivation Homo sapiens R-HSA-400508	1/23	0.402073	0.60395	0	0	2.013605	1.834641
Deadenylation of mRNA Homo sapiens R-HSA-429947	1/23	0.402073	0.60395	0	0	2.013605	1.834641
Signaling by BMP Homo sapiens R-HSA-201451	1/23	0.402073	0.60395	0	0	2.013605	1.834641
Branched-chain amino acid catabolism Homo sapiens R-HSA-70895	1/23	0.402073	0.60395	0	0	2.013605	1.834641

SHC-mediated cascade:FGFR4 Homo sapiens R-HSA-5654719	1/23	0.402073	0.60395	0	0	2.013605	1.834641
Dual incision in TC-NER Homo sapiens R-HSA-6782135	2/63	0.407223	0.610409	0	0	1.452832	1.305216
Glutamate Neurotransmitter Release Cycle Homo sapiens R-HSA-210500	1/24	0.415302	0.611039	0	0	1.925959	1.692435
Sema4D induced cell migration and growth-cone collapse Homo sapiens R-HSA-416572	1/24	0.415302	0.611039	0	0	1.925959	1.692435
TNFs bind their physiological receptors Homo sapiens R-HSA-5669034	1/24	0.415302	0.611039	0	0	1.925959	1.692435
Post-Elongation Processing of Intronless pre-mRNA Homo sapiens R-HSA-112297	1/24	0.415302	0.611039	0	0	1.925959	1.692435
Processing of Capped Intronless Pre-mRNA Homo sapiens R-HSA-75067	1/24	0.415302	0.611039	0	0	1.925959	1.692435
Synthesis of bile acids and bile salts via 7alpha-hydroxycholesterol Homo sapiens R-HSA-193368	1/24	0.415302	0.611039	0	0	1.925959	1.692435
Growth hormone receptor signaling Homo sapiens R-HSA-982772	1/24	0.415302	0.611039	0	0	1.925959	1.692435
Diseases associated with the TLR signaling cascade Homo sapiens R-HSA-5602358	1/24	0.415302	0.611039	0	0	1.925959	1.692435
Diseases of Immune System Homo sapiens R-HSA-5260271	1/24	0.415302	0.611039	0	0	1.925959	1.692435
Mitotic Prophase Homo sapiens R-HSA-68875	3/107	0.422352	0.617777	0	0	1.278299	1.101786
HATs acetylate histones Homo sapiens R-HSA-3214847	3/107	0.422352	0.617777	0	0	1.278299	1.101786
ER-Phagosome pathway Homo sapiens R-HSA-1236974	2/65	0.422597	0.617777	0	0	1.406566	1.211526
G2/M Checkpoints Homo sapiens R-HSA-69481	4/150	0.42399	0.617777	0	0	1.214237	1.04187
Activation of G protein gated Potassium channels Homo sapiens R-HSA-1296041	1/25	0.428239	0.617777	0	0	1.845616	1.565217
Inhibition of voltage gated Ca2+ channels via Gbeta/gamma subunits Homo sapiens R-HSA-997272	1/25	0.428239	0.617777	0	0	1.845616	1.565217

G protein gated Potassium channels Homo sapiens R-HSA-1296059	1/25	0.428239	0.617777	0	0	1.845616	1.565217
HDMs demethylate histones Homo sapiens R-HSA-3214842	1/25	0.428239	0.617777	0	0	1.845616	1.565217
Regulation of IFNA signaling Homo sapiens R-HSA-912694	1/25	0.428239	0.617777	0	0	1.845616	1.565217
Regulation of TP53 Activity Homo sapiens R-HSA-5633007	4/151	0.428933	0.617777	0	0	1.205914	1.020752
APC/C:Cdc20 mediated degradation of Securin Homo sapiens R-HSA-174154	2/66	0.430207	0.617777	0	0	1.384517	1.167825
Cytosolic sensors of pathogen-associated DNA Homo sapiens R-HSA-1834949	2/66	0.430207	0.617777	0	0	1.384517	1.167825
Disease Homo sapiens R-HSA-1643685	17/725	0.436279	0.618281	0	0	1.064972	0.883366
Macroautophagy Homo sapiens R-HSA-1632852	2/67	0.437763	0.618281	0	0	1.363147	1.126064
Regulation of RAS by GAPs Homo sapiens R-HSA-5658442	2/67	0.437763	0.618281	0	0	1.363147	1.126064
Assembly of the pre-replicative complex Homo sapiens R-HSA-68867	2/67	0.437763	0.618281	0	0	1.363147	1.126064
WNT ligand biogenesis and trafficking Homo sapiens R-HSA-3238698	1/26	0.440891	0.618281	0	0	1.771701	1.450948
Fertilization Homo sapiens R-HSA-1187000	1/26	0.440891	0.618281	0	0	1.771701	1.450948
Reproduction Homo sapiens R-HSA-1474165	1/26	0.440891	0.618281	0	0	1.771701	1.450948
Diseases associated with glycosaminoglycan metabolism Homo sapiens R-HSA-3560782	1/26	0.440891	0.618281	0	0	1.771701	1.450948
A tetrasaccharide linker sequence is required for GAG synthesis Homo sapiens R-HSA-1971475	1/26	0.440891	0.618281	0	0	1.771701	1.450948
Termination of O-glycan biosynthesis Homo sapiens R-HSA-977068	1/26	0.440891	0.618281	0	0	1.771701	1.450948
Resolution of D-loop Structures through Synthesis-Dependent Strand Annealing (SDSA) Homo sapiens R-HSA-5693554	1/26	0.440891	0.618281	0	0	1.771701	1.450948

SHC-mediated cascade:FGFR2 Homo sapiens R-HSA-5654699	1/26	0.440891	0.618281	0	0	1.771701	1.450948
Interferon alpha/beta signaling Homo sapiens R-HSA-909733	2/68	0.445265	0.622624	0	0	1.342424	1.086137
FCERI mediated NF-kB activation Homo sapiens R-HSA-2871837	3/111	0.445722	0.622624	0	0	1.230701	0.99448
Loss of Nlp from mitotic centrosomes Homo sapiens R-HSA-380259	2/69	0.45271	0.623454	0	0	1.32232	1.047943
Loss of proteins required for interphase microtubule organization?from the centrosome Homo sapiens R-HSA-380284	2/69	0.45271	0.623454	0	0	1.32232	1.047943
Sema4D in semaphorin signaling Homo sapiens R-HSA-400685	1/27	0.453263	0.623454	0	0	1.703471	1.347926
CREB phosphorylation through the activation of Ras Homo sapiens R-HSA-442742	1/27	0.453263	0.623454	0	0	1.703471	1.347926
VEGFR2 mediated vascular permeability Homo sapiens R-HSA-5218920	1/27	0.453263	0.623454	0	0	1.703471	1.347926
RORA activates gene expression Homo sapiens R-HSA-1368082	1/27	0.453263	0.623454	0	0	1.703471	1.347926
Pyruvate metabolism Homo sapiens R-HSA-70268	1/27	0.453263	0.623454	0	0	1.703471	1.347926
Caspase activation via extrinsic apoptotic signalig pathway Homo sapiens R-HSA-5357769	1/27	0.453263	0.623454	0	0	1.703471	1.347926
PPARA activates gene expression Homo sapiens R-HSA-1989781	3/113	0.457267	0.627759	0	0	1.2082	0.945402
trans-Golgi Network Vesicle Budding Homo sapiens R-HSA-199992	2/70	0.460098	0.629239	0	0	1.302807	1.01139
Clathrin derived vesicle budding Homo sapiens R-HSA-421837	2/70	0.460098	0.629239	0	0	1.302807	1.01139
Energy dependent regulation of mTOR by LKB1-AMPK Homo sapiens R-HSA-380972	1/28	0.465363	0.635229	0	0	1.640296	1.254725
APC/C:Cdh1 mediated degradation of Cdc20 and other APC/C:Cdh1 targeted proteins in late mitosis/early	2/71	0.467428	0.635631	0	0	1.28386	0.976389

G1 Homo sapiens R-HSA-174178							
Cdc20:Phospho-APC/C mediated degradation of Cyclin A Homo sapiens R-HSA-174184	2/71	0.467428	0.635631	0	0	1.28386	0.976389
Epigenetic regulation of gene expression Homo sapiens R-HSA-212165	3/115	0.468709	0.636169	0	0	1.186503	0.8991
Regulation of lipid metabolism by Peroxisome proliferator-activated receptor alpha (PPARalpha) Homo sapiens R-HSA-400206	3/116	0.47439	0.639227	0	0	1.175943	0.876931
APC:Cdc20 mediated degradation of cell cycle proteins prior to satisfation of the cell cycle checkpoint Homo sapiens R-HSA-179419	2/72	0.474698	0.639227	0	0	1.265455	0.94286
AURKA Activation by TPX2 Homo sapiens R-HSA-8854518	2/72	0.474698	0.639227	0	0	1.265455	0.94286
Surfactant metabolism Homo sapiens R-HSA-5683826	1/29	0.477195	0.639227	0	0	1.581633	1.170141
COPI-independent Golgi-to-ER retrograde traffic Homo sapiens R-HSA-6811436	1/29	0.477195	0.639227	0	0	1.581633	1.170141
Activation of BH3-only proteins Homo sapiens R-HSA-114452	1/29	0.477195	0.639227	0	0	1.581633	1.170141
PIWI-interacting RNA (piRNA) biogenesis Homo sapiens R-HSA-5601884	1/29	0.477195	0.639227	0	0	1.581633	1.170141
Role of LAT2/NTAL/LAB on calcium mobilization Homo sapiens R-HSA-2730905	4/162	0.482439	0.645049	0	0	1.121322	0.817332
TCR signaling Homo sapiens R-HSA-202403	3/118	0.485667	0.645483	0	0	1.155373	0.834448
Apoptosis Homo sapiens R-HSA-109581	4/163	0.48721	0.645483	0	0	1.114213	0.801185
Tight junction interactions Homo sapiens R-HSA-420029	1/30	0.488765	0.645483	0	0	1.527015	1.093149
Activation of the pre-replicative complex Homo sapiens R-HSA-68962	1/30	0.488765	0.645483	0	0	1.527015	1.093149
MAPK targets/ Nuclear events mediated by MAP kinases Homo sapiens R-HSA-450282	1/30	0.488765	0.645483	0	0	1.527015	1.093149
Binding and Uptake of Ligands by Scavenger Receptors Homo sapiens R-HSA-2173782	2/74	0.489057	0.645483	0	0	1.230177	0.879917

APC/C:Cdc20 mediated degradation of mitotic proteins Homo sapiens R-HSA-176409	2/74	0.489057	0.645483	0	0	1.230177	0.879917
Activation of APC/C and APC/C:Cdc20 mediated degradation of mitotic proteins Homo sapiens R-HSA-176814	2/75	0.496143	0.652832	0	0	1.213263	0.850365
Neuronal System Homo sapiens R-HSA-112316	7/301	0.499159	0.652832	0	0	1.054406	0.732634
Inwardly rectifying K+ channels Homo sapiens R-HSA-1296065	1/31	0.500081	0.652832	0	0	1.476039	1.022875
Trafficking of AMPA receptors Homo sapiens R-HSA-399719	1/31	0.500081	0.652832	0	0	1.476039	1.022875
Glutamate Binding. Activation of AMPA Receptors and Synaptic Plasticity Homo sapiens R-HSA-399721	1/31	0.500081	0.652832	0	0	1.476039	1.022875
Export of Viral Ribonucleoproteins from Nucleus Homo sapiens R-HSA-168274	1/31	0.500081	0.652832	0	0	1.476039	1.022875
Programmed Cell Death Homo sapiens R-HSA-5357801	4/166	0.501413	0.653384	0	0	1.09341	0.754808
Transcription-Coupled Nucleotide Excision Repair (TC-NER) Homo sapiens R-HSA-6781827	2/76	0.503167	0.654481	0	0	1.196806	0.822007
Activation of Matrix Metalloproteinases Homo sapiens R-HSA-1592389	1/32	0.511146	0.661266	0	0	1.428352	0.958567
RNA Polymerase I Transcription Termination Homo sapiens R-HSA-73863	1/32	0.511146	0.661266	0	0	1.428352	0.958567
Gluconeogenesis Homo sapiens R-HSA-70263	1/32	0.511146	0.661266	0	0	1.428352	0.958567
C-type lectin receptors (CLRs) Homo sapiens R-HSA-5621481	3/123	0.513334	0.662902	0	0	1.106948	0.738145
G2/M DNA damage checkpoint Homo sapiens R-HSA-69473	2/78	0.517023	0.665712	0	0	1.165191	0.76864
S Phase Homo sapiens R-HSA-69242	3/124	0.518772	0.665712	0	0	1.097743	0.720438
Inactivation. recovery and regulation of the phototransduction cascade Homo sapiens R-HSA-2514859	1/33	0.521967	0.665712	0	0	1.383645	0.899578

Resolution of D-Loop Structures Homo sapiens R-HSA-5693537	1/33	0.521967	0.665712	0	0	1.383645	0.899578
PI3K Cascade Homo sapiens R-HSA-109704	2/79	0.523854	0.665712	0	0	1.15	0.743524
Regulation of APC/C activators between G1/S and early anaphase Homo sapiens R-HSA-176408	2/79	0.523854	0.665712	0	0	1.15	0.743524
RIG-I/MDA5 mediated induction of IFN-alpha/beta pathways Homo sapiens R-HSA-168928	2/79	0.523854	0.665712	0	0	1.15	0.743524
Centrosome maturation Homo sapiens R-HSA-380287	2/79	0.523854	0.665712	0	0	1.15	0.743524
Recruitment of mitotic centrosome proteins and complexes Homo sapiens R-HSA-380270	2/79	0.523854	0.665712	0	0	1.15	0.743524
Vesicle-mediated transport Homo sapiens R-HSA-5653656	11/492	0.527362	0.668986	0	0	1.012233	0.647695
The phototransduction cascade Homo sapiens R-HSA-2514856	1/34	0.532549	0.674374	0	0	1.341648	0.845346
Peptide hormone metabolism Homo sapiens R-HSA-2980736	2/81	0.53732	0.679218	0	0	1.120771	0.696179
Post NMDA receptor activation events Homo sapiens R-HSA-438064	1/35	0.542897	0.683992	0	0	1.302121	0.795382
Global Genome Nucleotide Excision Repair (GG-NER) Homo sapiens R-HSA-5696399	2/82	0.543955	0.683992	0	0	1.106705	0.673861
Antigen processing-Cross presentation Homo sapiens R-HSA-1236975	2/82	0.543955	0.683992	0	0	1.106705	0.673861
p75 NTR receptor-mediated signalling Homo sapiens R-HSA-193704	2/83	0.550522	0.689834	0	0	1.092985	0.65239
PI5P. PP2A and IER3 Regulate PI3K/AKT Signaling Homo sapiens R-HSA-6811558	2/83	0.550522	0.689834	0	0	1.092985	0.65239
Class B/2 (Secretin family receptors) Homo sapiens R-HSA-373080	2/84	0.557023	0.696764	0	0	1.079601	0.631727
Intra-Golgi and retrograde Golgi-to-ER traffic Homo sapiens R-HSA-6811442	4/179	0.560816	0.697521	0	0	1.011507	0.585018

Chromatin organization Homo sapiens R-HSA-4839726	5/226	0.561494	0.697521	0	0	1.001118	0.5778
Chromatin modifying enzymes Homo sapiens R-HSA-3247509	5/226	0.561494	0.697521	0	0	1.001118	0.5778
Defective B3GALTL causes Peters-plus syndrome (PpS) Homo sapiens R-HSA-5083635	1/37	0.562913	0.697521	0	0	1.229655	0.706597
APC/C-mediated degradation of cell cycle proteins Homo sapiens R-HSA-174143	2/85	0.563457	0.697521	0	0	1.066539	0.611835
Regulation of mitotic cell cycle Homo sapiens R-HSA-453276	2/85	0.563457	0.697521	0	0	1.066539	0.611835
O-glycosylation of TSR domain-containing proteins Homo sapiens R-HSA-5173214	1/38	0.57259	0.70518	0	0	1.19636	0.667072
Glutathione conjugation Homo sapiens R-HSA-156590	1/38	0.57259	0.70518	0	0	1.19636	0.667072
Signaling by FGFR1 in disease Homo sapiens R-HSA-5655302	1/38	0.57259	0.70518	0	0	1.19636	0.667072
Signaling by Hedgehog Homo sapiens R-HSA-5358351	3/136	0.581335	0.709533	0	0	0.998082	0.541387
GABA B receptor activation Homo sapiens R-HSA-977444	1/39	0.582054	0.709533	0	0	1.164817	0.630389
Activation of GABAB receptors Homo sapiens R-HSA-991365	1/39	0.582054	0.709533	0	0	1.164817	0.630389
mTOR signalling Homo sapiens R-HSA-165159	1/39	0.582054	0.709533	0	0	1.164817	0.630389
NS1 Mediated Effects on Host Pathways Homo sapiens R-HSA-168276	1/39	0.582054	0.709533	0	0	1.164817	0.630389
Nuclear signaling by ERBB4 Homo sapiens R-HSA-1251985	1/39	0.582054	0.709533	0	0	1.164817	0.630389
Membrane Trafficking Homo sapiens R-HSA-199991	9/420	0.584425	0.711216	0	0	0.968308	0.520103
PKB-mediated events Homo sapiens R-HSA-109703	1/40	0.591309	0.717162	0	0	1.134892	0.596291
Transcriptional activation of mitochondrial biogenesis Homo sapiens R-HSA-2151201	1/40	0.591309	0.717162	0	0	1.134892	0.596291
Negative regulation of the PI3K/AKT network Homo sapiens R-HSA-199418	2/90	0.594615	0.719956	0	0	1.005682	0.522794

Intraflagellar transport Homo sapiens R-HSA-5620924	1/41	0.600359	0.724192	0	0	1.106463	0.564548
Host Interactions with Influenza Factors Homo sapiens R-HSA-168253	1/41	0.600359	0.724192		0	0	1.106463	0.564548
DNA Repair Homo sapiens R-HSA-73894	6/285	0.604614	0.724192		0	0	0.950922	0.478472
Factors involved in megakaryocyte development and platelet production Homo sapiens R-HSA-983231	3/141	0.605838	0.724192		0	0	0.961672	0.481934
HIV Life Cycle Homo sapiens R-HSA-162587	3/141	0.605838	0.724192		0	0	0.961672	0.481934
Signaling by Retinoic Acid Homo sapiens R-HSA-5362517	1/42	0.609209	0.724192		0	0	1.07942	0.534954
Non-integrin membrane-ECM interactions Homo sapiens R-HSA-3000171	1/42	0.609209	0.724192		0	0	1.07942	0.534954
Intrinsic Pathway for Apoptosis Homo sapiens R-HSA-109606	1/42	0.609209	0.724192		0	0	1.07942	0.534954
Glucose transport Homo sapiens R-HSA-70153	1/42	0.609209	0.724192		0	0	1.07942	0.534954
BMAL1:CLOCK.NPAS2 activates circadian gene expression Homo sapiens R-HSA-1368108	1/42	0.609209	0.724192		0	0	1.07942	0.534954
Activation of gene expression by SREBF (SREBP) Homo sapiens R-HSA-2426168	1/42	0.609209	0.724192		0	0	1.07942	0.534954
Interferon gamma signaling Homo sapiens R-HSA-877300	2/93	0.612498	0.726898		0	0	0.972378	0.476669
Signaling by FGFR2 in disease Homo sapiens R-HSA-5655253	1/43	0.617864	0.732056		0	0	1.053666	0.507327
Beta-catenin independent WNT signaling Homo sapiens R-HSA-3858494	3/144	0.620077	0.732467		0	0	0.941065	0.449747
Peptide ligand-binding receptors Homo sapiens R-HSA-375276	4/193	0.620251	0.732467		0	0	0.935904	0.447017
Influenza Infection Homo sapiens R-HSA-168254	3/147	0.633962	0.746668		0	0	0.921317	0.419906
RMTs methylate histone arginines Homo sapiens R-HSA-3214858	1/45	0.634603	0.746668		0	0	1.005669	0.457333
Anchoring of the basal body to the plasma membrane Homo sapiens R-HSA-5620912	2/97	0.635396	0.746668		0	0	0.931244	0.422326

Potassium Channels Homo sapiens R-HSA-1296071	2/99	0.646442	0.75717	0	0	0.911949	0.397858
Hedgehog 'off' state Homo sapiens R-HSA-5610787	2/99	0.646442	0.75717	0	0	0.911949	0.397858
Nucleotide-binding domain. leucine rich repeat containing receptor (NLR) signaling pathways Homo sapiens R-HSA-168643	1/47	0.650611	0.759803	0	0	0.961846	0.413442
Infectious disease Homo sapiens R-HSA-5663205	7/348	0.651851	0.759803	0	0	0.906859	0.388081
Stimuli-sensing channels Homo sapiens R-HSA-2672351	2/100	0.651865	0.759803	0	0	0.902597	0.386238
Transport of glucose and other sugars. bile salts and organic acids. metal ions and amine compounds Homo sapiens R-HSA-425366	2/101	0.657221	0.763645	0	0	0.893434	0.375005
EPH-ephrin mediated repulsion of cells Homo sapiens R-HSA-3928665	1/48	0.658351	0.763645	0	0	0.941333	0.393493
Pyruvate metabolism and Citric Acid (TCA) cycle Homo sapiens R-HSA-71406	1/48	0.658351	0.763645	0	0	0.941333	0.393493
Retrograde transport at the Trans-Golgi-Network Homo sapiens R-HSA-6811440	1/49	0.66592	0.771178	0	0	0.921674	0.37474
Chondroitin sulfate/dermatan sulfate metabolism Homo sapiens R-HSA-1793185	1/50	0.673321	0.777242	0	0	0.902818	0.357095
Deadenylation-dependent mRNA decay Homo sapiens R-HSA-429914	1/50	0.673321	0.777242	0	0	0.902818	0.357095
Platelet degranulation Homo sapiens R-HSA-114608	2/105	0.67799	0.781376	0	0	0.858561	0.333656
Degradation of the extracellular matrix Homo sapiens R-HSA-1474228	2/106	0.68302	0.78577	0	0	0.850262	0.324147
Antigen processing: Ubiquitination & Proteasome degradation Homo sapiens R-HSA-983168	5/260	0.683992	0.78577	0	0	0.86611	0.328957
Nucleotide Excision Repair Homo sapiens R-HSA-5696398	2/108	0.692886	0.794716	0	0	0.834134	0.306035
Arachidonic acid metabolism Homo sapiens R-HSA-2142753	1/53	0.694557	0.795362	0	0	0.850602	0.310028
Cell Cycle. Mitotic Homo sapiens R-HSA-69278	9/462	0.695789	0.795504	0	0	0.876604	0.317952

Response to elevated platelet cytosolic Ca2+ Homo sapiens R-HSA-76005	2/110	0.702498	0.800625	0	0	0.818603	0.289059
O-linked glycosylation Homo sapiens R-HSA-5173105	2/110	0.702498	0.800625	0	0	0.818603	0.289059
GABA receptor activation Homo sapiens R-HSA-977443	1/55	0.707944	0.803667	0	0	0.819014	0.282879
Regulation of cholesterol biosynthesis by SREBP (SREBF) Homo sapiens R-HSA-1655829	1/55	0.707944	0.803667	0	0	0.819014	0.282879
M Phase Homo sapiens R-HSA-68886	5/268	0.7092	0.803667	0	0	0.839417	0.288439
Fatty acid. triacylglycerol. and ketone body metabolism Homo sapiens R-HSA-535734	4/217	0.709645	0.803667	0	0	0.829421	0.284484
tRNA processing in the nucleus Homo sapiens R-HSA-6784531	1/57	0.720745	0.814708	0	0	0.789683	0.258597
Class A/1 (Rhodopsin-like receptors) Homo sapiens R-HSA-373076	6/323	0.721663	0.814708	0	0	0.835282	0.272466
rRNA modification in the nucleus Homo sapiens R-HSA-6790901	1/58	0.726935	0.819371	0	0	0.775789	0.247414
Translocation of GLUT4 to the plasma membrane Homo sapiens R-HSA-1445148	1/60	0.738905	0.827666	0	0	0.749414	0.226762
Cleavage of Growing Transcript in the Termination Region Homo sapiens R-HSA-109688	1/60	0.738905	0.827666	0	0	0.749414	0.226762
RNA Polymerase II Transcription Termination Homo sapiens R-HSA-73856	1/60	0.738905	0.827666	0	0	0.749414	0.226762
Post-Elongation Processing of the Transcript Homo sapiens R-HSA-76044	1/60	0.738905	0.827666	0	0	0.749414	0.226762
Cell-cell junction organization Homo sapiens R-HSA-421270	1/61	0.744693	0.831554	0	0	0.736886	0.217222
TP53 Regulates Transcription of DNA Repair Genes Homo sapiens R-HSA-6796648	1/61	0.744693	0.831554	0	0	0.736886	0.217222
FCERI mediated Ca+2 mobilization Homo sapiens R-HSA-2871809	1/62	0.750352	0.835966	0	0	0.724769	0.208163
Glycosaminoglycan metabolism Homo sapiens R-HSA-1630316	2/121	0.750972	0.835966	0	0	0.742513	0.212646
Rho GTPase cycle Homo sapiens R-HSA-194840	2/122	0.755024	0.837541	0	0	0.736288	0.206901

Collagen biosynthesis and modifying enzymes Homo sapiens R-HSA-1650814	1/63	0.755887	0.837541	0	0	0.713042	0.199555
Signaling by FGFR in disease Homo sapiens R-HSA-1226099	1/63	0.755887	0.837541	0	0	0.713042	0.199555
O-linked glycosylation of mucins Homo sapiens R-HSA-913709	1/66	0.771766	0.853818	0	0	0.680028	0.176178
GPCR ligand binding Homo sapiens R-HSA-500792	8/447	0.77468	0.855724	0	0	0.802788	0.204956
Late Phase of HIV Life Cycle Homo sapiens R-HSA-162599	2/128	0.778158	0.858245	0	0	0.70101	0.175832
Host Interactions of HIV factors Homo sapiens R-HSA-162909	2/129	0.781822	0.860964	0	0	0.695455	0.171171
G alpha (q) signalling events Homo sapiens R-HSA-416476	3/191	0.797157	0.876506	0	0	0.704091	0.15962
Immune System Homo sapiens R-HSA-168256	30/1547	0.798841	0.877015	0	0	0.865962	0.19449
Influenza Life Cycle Homo sapiens R-HSA-168255	2/136	0.806017	0.883517	0	0	0.658887	0.14209
Class I MHC mediated antigen processing & presentation Homo sapiens R-HSA-983169	5/305	0.807224	0.883517	0	0	0.734477	0.157291
G alpha (s) signalling events Homo sapiens R-HSA-418555	2/142	0.824827	0.900906	0	0	0.630455	0.121414
COPI-mediated anterograde transport Homo sapiens R-HSA-6807878	1/78	0.825622	0.900906	0	0	0.573696	0.109931
Complement cascade Homo sapiens R-HSA-166658	1/80	0.833274	0.907876	0	0	0.559115	0.101979
Metabolism of vitamins and cofactors Homo sapiens R-HSA-196854	2/148	0.841983	0.915976	0	0	0.604359	0.103947
Signal Transduction Homo sapiens R-HSA-162582	48/2465	0.846621	0.916517	0	0	0.863982	0.143854
Mitochondrial translation initiation Homo sapiens R-HSA-5368286	1/84	0.847587	0.916517	0	0	0.53206	0.087982
Mitochondrial translation termination Homo sapiens R-HSA-5419276	1/84	0.847587	0.916517	0	0	0.53206	0.087982
Mitochondrial translation elongation Homo sapiens R-HSA-5389840	1/84	0.847587	0.916517	0	0	0.53206	0.087982
Organelle biogenesis and maintenance Homo sapiens R-HSA-1852241	5/326	0.850018	0.917412	0	0	0.685679	0.111421
Collagen formation Homo sapiens R-HSA-1474290	1/85	0.85097	0.917412	0	0	0.525699	0.084837

Cell junction organization Homo sapiens R-HSA-446728	1/86	0.854277	0.919107	0	0	0.519488	0.081819
The citric acid (TCA) cycle and respiratory electron transport Homo sapiens R-HSA-1428517	2/153	0.855102	0.919107	0	0	0.584196	0.091447
Adaptive Immune System Homo sapiens R-HSA-1280218	13/762	0.86448	0.926835	0	0	0.760974	0.110819
Immunoregulatory interactions between a Lymphoid and a non-Lymphoid cell Homo sapiens R-HSA-198933	2/157	0.864874	0.926835	0	0	0.569003	0.082603
Mitochondrial translation Homo sapiens R-HSA-5368287	1/90	0.866791	0.927505	0	0	0.496038	0.070912
HIV Infection Homo sapiens R-HSA-162906	3/222	0.871524	0.931181	0	0	0.603457	0.082983
Extracellular matrix organization Homo sapiens R-HSA-1474244	4/283	0.874714	0.933202	0	0	0.631054	0.084471
Separation of Sister Chromatids Homo sapiens R-HSA-2467813	2/162	0.876239	0.933442	0	0	0.55108	0.072807
Cell Cycle Homo sapiens R-HSA-1640170	9/566	0.881812	0.937986	0	0	0.709048	0.089182
L1CAM interactions Homo sapiens R-HSA-373760	1/96	0.88358	0.938477	0	0	0.464566	0.057501
Regulation of actin dynamics for phagocytic cup formation Homo sapiens R-HSA-2029482	1/97	0.886165	0.939833	0	0	0.459703	0.055556
Phase II conjugation Homo sapiens R-HSA-156580	1/100	0.893583	0.946301	0	0	0.445704	0.050149
Cell surface interactions at the vascular wall Homo sapiens R-HSA-202733	1/101	0.895946	0.946664	0	0	0.441224	0.04848
Mitotic Anaphase Homo sapiens R-HSA-68882	2/173	0.898207	0.946664	0	0	0.515338	0.055324
Mitotic Metaphase and Anaphase Homo sapiens R-HSA-2555396	2/174	0.900014	0.946664	0	0	0.512315	0.05397
MHC class II antigen presentation Homo sapiens R-HSA-2132295	1/103	0.900517	0.946664	0	0	0.432529	0.045323
tRNA processing Homo sapiens R-HSA-72306	1/103	0.900517	0.946664	0	0	0.432529	0.045323
G alpha (i) signalling events Homo sapiens R-HSA-418594	3/240	0.902659	0.947528	0	0	0.557106	0.057053

Nonsense-Mediated Decay (NMD) Homo sapiens R-HSA-927802	1/106	0.907001	0.94931	0	0	0.420106	0.041007
Nonsense Mediated Decay (NMD) enhanced by the Exon Junction Complex (EJC) Homo sapiens R-HSA-975957	1/106	0.907001	0.94931	0	0	0.420106	0.041007
Signaling by Rho GTPases Homo sapiens R-HSA-194315	5/367	0.911022	0.952125	0	0	0.606723	0.056539
Respiratory electron transport. ATP synthesis by chemiosmotic coupling. and heat production by uncoupling proteins. Homo sapiens R-HSA-163200	1/109	0.913063	0.952125	0	0	0.408373	0.037142
Integration of energy metabolism Homo sapiens R-HSA-163685	1/110	0.914995	0.952125	0	0	0.404606	0.035944
Golgi-to-ER retrograde transport Homo sapiens R-HSA-8856688	1/110	0.914995	0.952125	0	0	0.404606	0.035944
Assembly of the primary cilium Homo sapiens R-HSA-5617833	2/187	0.920928	0.956913	0	0	0.475995	0.039209
RHO GTPase Effectors Homo sapiens R-HSA-195258	3/255	0.923222	0.95791	0	0	0.523538	0.041823
Fcgamma receptor (FCGR) dependent phagocytosis Homo sapiens R-HSA-2029480	1/120	0.932107	0.965733	0	0	0.370415	0.026043
RNA Polymerase II Transcription Homo sapiens R-HSA-73857	1/124	0.937947	0.970383	0	0	0.358295	0.022953
Ion channel transport Homo sapiens R-HSA-983712	2/203	0.941035	0.972177	0	0	0.437743	0.026604
Influenza Viral RNA Transcription and Replication Homo sapiens R-HSA-168273	1/128	0.943285	0.973102	0	0	0.346939	0.020257
Cell-Cell communication Homo sapiens R-HSA-1500931	1/131	0.946986	0.97412	0	0	0.33888	0.018459
ER to Golgi Anterograde Transport Homo sapiens R-HSA-199977	1/131	0.946986	0.97412	0	0	0.33888	0.018459
mRNA Splicing - Major Pathway Homo sapiens R-HSA-72163	1/134	0.950446	0.976017	0	0	0.331185	0.016832
Cardiac conduction Homo sapiens R-HSA-5576891	1/135	0.951549	0.976017	0	0	0.328697	0.016325
mRNA Splicing Homo sapiens R-HSA-72172	1/144	0.960432	0.983724	0	0	0.307867	0.012429

Metabolism of proteins Homo sapiens R-HSA-392499	16/1074	0.966751	0.988155	0	0	0.656745	0.022207
Signaling by GPCR Homo sapiens R-HSA-372790	20/1293	0.967511	0.988155	0	0	0.680744	0.022484
Transport to the Golgi and subsequent modification Homo sapiens R-HSA-948021	1/162	0.973619	0.99298	0	0	0.273193	0.007304
Major pathway of rRNA processing in the nucleolus Homo sapiens R-HSA-6791226	1/166	0.975892	0.993657	0	0	0.266515	0.006504
Platelet activation. signaling and aggregation Homo sapiens R-HSA-76002	2/253	0.97705	0.993657	0	0	0.349638	0.008118
rRNA processing Homo sapiens R-HSA-72312	1/180	0.982417	0.996514	0	0	0.245493	0.004355
SLC-mediated transmembrane transport Homo sapiens R-HSA-425407	2/268	0.982821	0.996514	0	0	0.329665	0.005712
Hemostasis Homo sapiens R-HSA-109582	6/552	0.984023	0.996514	0	0	0.479181	0.007718
Processing of Capped Intron-Containing Pre-mRNA Homo sapiens R-HSA-72203	1/193	0.986886	0.997469	0	0	0.228718	0.003019
Muscle contraction Homo sapiens R-HSA-397014	1/196	0.987744	0.997469	0	0	0.225164	0.002777
Metabolism of lipids and lipoproteins Homo sapiens R-HSA-556833	7/659	0.991372	0.999727	0	0	0.466617	0.004043
Metabolism Homo sapiens R-HSA-1430728	28/1908	0.994177	0.999833	0	0	0.635965	0.003714
Asparagine N-linked glycosylation Homo sapiens R-HSA-446203	1/259	0.997048	0.999833	0	0	0.169629	5.01E-04
Transmembrane transport of small molecules Homo sapiens R-HSA-382551	4/594	0.999229	0.999833	0	0	0.2936	2.26E-04
Post-translational protein modification Homo sapiens R-HSA-597592	3/521	0.999351	0.999833	0	0	0.251185	1.63E-04
Metabolism of amino acids and derivatives Homo sapiens R-HSA-71291	1/335	0.999471	0.999833	0	0	0.130514	6.90E-05
GPCR downstream signaling Homo sapiens R-HSA-388396	8/983	0.999833	0.999833	0	0	0.351327	5.88E-05
