# Supplementary material for: Evidence for mitochondrial Lonp1 expression in the nucleus
Source: Sci Rep. 2022 Jun 27;12:10877. doi: 10.1038/s41598-022-14860-0 (PMC9237102; doi:10.1038/s41598-022-14860-0)
Supplement: Supplementary file 5 — Supplementary Table 3. [file 41598_2022_14860_MOESM5_ESM.pdf]

**Supplementary Table 3**

| Ctrl siLonp1 vs siScramble | | | | | | | |
|---|---|---|---|---|---|---|---|
| NAME | SIZE | ES | NES | NOM p-val | FDR q-val | FWER p-val | RANK AT MAX |
| REACTOME_NUCLEAR_EVENTS_KINASE_AND_TRANSCRIPTION_FACTOR_ACTIVATION_ | 54 | 0.724 | 2.478 | 0.000 | 0.000 | 0 | 1692 |
| REACTOME_NGF_STIMULATED_TRANSCRIPTION | 33 | 0.780 | 2.386 | 0.000 | 0.000 | 0 | 1342 |
| REACTOME_ELASTIC_FIBRE_FORMATION | 25 | 0.785 | 2.294 | 0.000 | 0.000 | 0 | 1534 |
| REACTOME_SIGNALING_BY_NTRKS | 113 | 0.573 | 2.209 | 0.000 | 0.000 | 0 | 2902 |
| REACTOME_EXTRACELLULAR_MATRIX_ORGANIZATION | 164 | 0.539 | 2.170 | 0.000 | 0.000 | 0 | 1559 |
| REACTOME_KERATINIZATION | 38 | 0.664 | 2.130 | 0.000 | 0.000 | 0.001 | 1514 |
| REACTOME_MOLECULES_ASSOCIATED_WITH_ELASTIC_FIBRES | 19 | 0.782 | 2.121 | 0.000 | 0.000 | 0.001 | 1534 |
| REACTOME_CONSTITUTIVE_SIGNALING_BY_ABERRANT_PI3K_IN_CANCER | 39 | 0.647 | 2.067 | 0.000 | 0.001 | 0.008 | 1477 |
| REACTOME_ASSEMBLY_AND_CELL_SURFACE_PRESENTATION_OF_NMDA_RECEPTORS | 24 | 0.710 | 2.064 | 0.000 | 0.001 | 0.009 | 2396 |
| REACTOME_FORMATION_OF_THE_CORNIFIED_ENVELOPE | 34 | 0.671 | 2.064 | 0.000 | 0.001 | 0.009 | 1020 |
| REACTOME_SIGNALING_BY_RECEPTOR_TYROSINE_KINASES | 377 | 0.464 | 2.050 | 0.000 | 0.001 | 0.011 | 3113 |
| REACTOME_MAPK_TARGETS_NUCLEAR_EVENTS_MEDIATED_BY_MAP_KINASES | 29 | 0.669 | 2.023 | 0.000 | 0.002 | 0.021 | 2240 |
| REACTOME_SYNDECAN_INTERACTIONS | 18 | 0.757 | 2.015 | 0.000 | 0.002 | 0.025 | 1687 |
| REACTOME_ROS_AND_RNS_PRODUCTION_IN_PHAGOCYTES | 21 | 0.702 | 1.993 | 0.000 | 0.003 | 0.039 | 3038 |
| REACTOME_PI3K_AKT_SIGNALING_IN_CANCER | 66 | 0.566 | 1.991 | 0.000 | 0.003 | 0.04 | 2102 |
| REACTOME_CHONDROITIN_SULFATE_DERMATAN_SULFATE_METABOLISM | 36 | 0.628 | 1.990 | 0.000 | 0.003 | 0.042 | 2333 |
| REACTOME_NEGATIVE_REGULATION_OF_THE_PI3K_AKT_NETWORK | 70 | 0.547 | 1.942 | 0.000 | 0.009 | 0.118 | 2742 |
| REACTOME_FORMATION_OF_TUBULIN_FOLDING_INTERMEDIATES_BY_CCT_TRIC | 19 | 0.713 | 1.932 | 0.002 | 0.010 | 0.139 | 2113 |
| REACTOME_DEGRADATION_OF_THE_EXTRACELLULAR_MATRIX | 70 | 0.540 | 1.924 | 0.000 | 0.011 | 0.162 | 1321 |
| REACTOME_VOLTAGE_GATED_POTASSIUM_CHANNELS | 12 | 0.772 | 1.921 | 0.002 | 0.011 | 0.168 | 1676 |

| | | | | | | | |
|---|---|---|---|---|---|---|---|
| REACTOME_SIGNALING_BY_INSULIN_RECEPTOR | 54 | 0.561 | 1.917 | 0.000 | 0.011 | 0.18 | 3038 |
| REACTOME_HEPARAN_SULFATE_HEPARIN_HS_GAG_METABOLISM | 34 | 0.613 | 1.912 | 0.003 | 0.011 | 0.19 | 1755 |
| REACTOME_GAP_JUNCTION_TRAFFICKING_AND_REGULATION | 31 | 0.629 | 1.911 | 0.000 | 0.011 | 0.19 | 2601 |
| REACTOME_ESTROGEN_DEPENDENT_NUCLEAR_EVENTS_DOWNSTREAM_OF_ESR_MEMBRANE_SIGNALING | 20 | 0.691 | 1.910 | 0.000 | 0.010 | 0.193 | 2042 |
| REACTOME_INTEGRIN_CELL_SURFACE_INTERACTIONS | 45 | 0.574 | 1.906 | 0.000 | 0.010 | 0.201 | 1511 |
| REACTOME_ECM_PROTEOGLYCANS | 37 | 0.603 | 1.906 | 0.000 | 0.010 | 0.204 | 1511 |
| REACTOME_NEUREXINS_AND_NEUROLIGINS | 34 | 0.613 | 1.900 | 0.000 | 0.010 | 0.215 | 2396 |
| REACTOME_COLLAGEN_DEGRADATION | 29 | 0.626 | 1.894 | 0.000 | 0.011 | 0.232 | 1250 |
| REACTOME_A_TETRASACCHARIDE_LINKER_SEQUENCE_IS_REQUIRED_FOR_GAG_SYNTHESIS | 15 | 0.747 | 1.883 | 0.000 | 0.012 | 0.26 | 2333 |
| REACTOME_INSULIN_RECEPTOR_RECYCLING | 21 | 0.676 | 1.872 | 0.002 | 0.015 | 0.316 | 3330 |
| REACTOME_DISSOLUTION_OF_FIBRIN_CLOT | 10 | 0.806 | 1.868 | 0.000 | 0.016 | 0.333 | 1236 |
| REACTOME_COLLAGEN_CHAIN_TRIMERIZATION | 15 | 0.721 | 1.866 | 0.000 | 0.016 | 0.349 | 1250 |
| REACTOME_PLASMA_LIPOPROTEIN_ASSEMBLY | 9 | 0.820 | 1.855 | 0.002 | 0.018 | 0.405 | 685 |
| REACTOME_RHO_GTPASES_ACTIVATE_CIT | 18 | 0.675 | 1.855 | 0.002 | 0.018 | 0.411 | 2597 |
| REACTOME_GAP_JUNCTION_ASSEMBLY | 18 | 0.690 | 1.853 | 0.000 | 0.018 | 0.417 | 2209 |
| REACTOME_CELL_EXTRACELLULAR_MATRIX_INTERACTIONS | 17 | 0.701 | 1.847 | 0.002 | 0.018 | 0.438 | 2601 |
| REACTOME_RAF_INDEPENDENT_MAPK1_3_ACTIVATION | 22 | 0.658 | 1.843 | 0.002 | 0.020 | 0.465 | 3595 |
| REACTOME_DEFECTIVE_B4GALT7_CAUSES_EDS_PROGEROID_TYPE | 11 | 0.774 | 1.841 | 0.002 | 0.020 | 0.474 | 1541 |
| REACTOME_NON_INTEGRIN_MEMBRANE_ECM_INTERACTIONS | 37 | 0.577 | 1.838 | 0.000 | 0.020 | 0.489 | 1687 |
| REACTOME_RECYCLING_PATHWAY_OF_L1 | 39 | 0.573 | 1.829 | 0.002 | 0.022 | 0.53 | 1399 |
| REACTOME_CHONDROITIN_SULFATE_BIOSYNTHESIS | 13 | 0.753 | 1.828 | 0.002 | 0.022 | 0.538 | 1917 |
| REACTOME_SHC1_EVENTS_IN_EGFR_SIGNALING | 10 | 0.794 | 1.825 | 0.002 | 0.022 | 0.556 | 770 |
| REACTOME_TGF_BETA_RECEPTOR_SIGNALING_ACTIVATES_SMADS | 32 | 0.588 | 1.823 | 0.000 | 0.022 | 0.567 | 2290 |

| | | | | | | | |
|---|---|---|---|---|---|---|---|
| REACTOME_RESPONSE_TO_METAL_IONS | 10 | 0.776 | 1.810 | 0.002 | 0.026 | 0.626 | 1747 |
| REACTOME_COLLAGEN_FORMATION | 50 | 0.532 | 1.808 | 0.000 | 0.026 | 0.635 | 1817 |
| REACTOME_INTERLEUKIN_6_FAMILY_SIGNALING | 14 | 0.707 | 1.806 | 0.003 | 0.026 | 0.645 | 787 |
| REACTOME_NEGATIVE_REGULATION_OF_MAPK_PATHWAY | 42 | 0.551 | 1.801 | 0.000 | 0.028 | 0.671 | 3513 |
| REACTOME_YAP1_AND_WWTR1_TAZ_STIMULATED_GENE_EXPRESSION | 12 | 0.747 | 1.801 | 0.004 | 0.027 | 0.671 | 2313 |
| REACTOME_SIGNALING_BY_INTERLEUKINS | 302 | 0.417 | 1.800 | 0.000 | 0.027 | 0.677 | 1692 |
| REACTOME_CHEMOKINE_RECEPTORS_BIND_CHEMOKINES | 10 | 0.780 | 1.800 | 0.002 | 0.026 | 0.681 | 899 |
| REACTOME_INTERLEUKIN_17_SIGNALING | 65 | 0.507 | 1.799 | 0.000 | 0.026 | 0.681 | 2920 |
| REACTOME_BASIGIN_INTERACTIONS | 19 | 0.659 | 1.795 | 0.002 | 0.027 | 0.704 | 2929 |
| REACTOME_TRANSPORT_OF_CONNEXONS_TO_THE_PLASMA_MEMBRANE | 12 | 0.750 | 1.794 | 0.002 | 0.027 | 0.705 | 1399 |
| REACTOME_WNT_LIGAND_BIOGENESIS_AND_TRAFFICKING | 13 | 0.712 | 1.790 | 0.006 | 0.028 | 0.72 | 1887 |
| REACTOME_ACTIVATION_OF_NMDA_RECEPTORS_AND_POSTSYNAPTIC_EVENTS | 63 | 0.501 | 1.786 | 0.000 | 0.029 | 0.74 | 2419 |
| REACTOME_PARASITE_INFECTION | 48 | 0.533 | 1.785 | 0.000 | 0.029 | 0.747 | 3175 |
| REACTOME_FLT3_SIGNALING | 217 | 0.424 | 1.783 | 0.000 | 0.029 | 0.754 | 2029 |
| REACTOME_COLLAGEN_BIOSYNTHESIS_AND_MODIFYING_ENZYMES | 33 | 0.572 | 1.779 | 0.003 | 0.030 | 0.775 | 1250 |
| REACTOME_MAPK_FAMILY_SIGNALING_CASCADES | 245 | 0.420 | 1.775 | 0.000 | 0.031 | 0.788 | 3074 |
| REACTOME_COOPERATION_OF_PREFOLDIN_AND_TRIC_CCT_IN_ACTIN_AND_TUBULIN_FOLDING | 26 | 0.607 | 1.774 | 0.000 | 0.031 | 0.79 | 2753 |
| REACTOME_SEALING_OF_THE_NUCLEAR_ENVELOPE_NE_BY_ESCRT_III | 24 | 0.625 | 1.774 | 0.003 | 0.031 | 0.792 | 2949 |
| REACTOME_NEURONAL_SYSTEM | 196 | 0.431 | 1.773 | 0.000 | 0.030 | 0.795 | 2466 |
| REACTOME_ASSEMBLY_OF_COLLAGEN_FIBRILS_AND_OTHER_MULTIMERIC_STRUCTURES | 32 | 0.572 | 1.772 | 0.003 | 0.031 | 0.803 | 1530 |
| REACTOME_HEMOSTASIS | 385 | 0.398 | 1.765 | 0.000 | 0.033 | 0.827 | 2447 |
| REACTOME_EPHRIN_SIGNALING | 17 | 0.661 | 1.760 | 0.002 | 0.035 | 0.843 | 3298 |
| REACTOME_DOWNREGULATION_OF_TGF_BETA_RECEPTOR_SIGNALING | 26 | 0.598 | 1.759 | 0.003 | 0.035 | 0.845 | 2290 |

| | | | | | | | |
|---|---|---|---|---|---|---|---|
| REACTOME_FOXO_MEDIATED_TRANSCRIPTION_OF_CELL_CYCLE_GENES | 16 | 0.660 | 1.756 | 0.004 | 0.036 | 0.858 | 1367 |
| REACTOME_HS_GAG_BIOSYNTHESIS | 18 | 0.652 | 1.747 | 0.004 | 0.039 | 0.887 | 1755 |
| REACTOME_RHO_GTPASES_ACTIVATE_WASPS_AND_WAVES | 33 | 0.572 | 1.742 | 0.002 | 0.041 | 0.9 | 3175 |
| REACTOME_PROTEIN_PROTEIN_INTERACTIONS_AT_SYNAPSES | 50 | 0.518 | 1.741 | 0.002 | 0.041 | 0.905 | 2674 |
| REACTOME_INTERLEUKIN_4_AND_INTERLEUKIN_13_SIGNALING | 62 | 0.503 | 1.741 | 0.002 | 0.041 | 0.906 | 2163 |
| REACTOME_GLYCOSAMINOGLYCAN_METABOLISM | 85 | 0.475 | 1.739 | 0.003 | 0.041 | 0.909 | 1917 |
| REACTOME_CELL_SURFACE_INTERACTIONS_AT_THE_VASCULAR_WALL | 75 | 0.475 | 1.735 | 0.000 | 0.043 | 0.924 | 1732 |
| REACTOME_RHO_GTPASES_ACTIVATE_IQGAPS | 23 | 0.602 | 1.726 | 0.000 | 0.047 | 0.942 | 3589 |
| REACTOME_METALLOTHIONEINS_BIND_METALS | 8 | 0.784 | 1.725 | 0.004 | 0.047 | 0.942 | 1747 |
| REACTOME_GPCR_LIGAND_BINDING | 106 | 0.450 | 1.724 | 0.000 | 0.047 | 0.944 | 1669 |
| REACTOME_MUSCLE_CONTRACTION | 99 | 0.460 | 1.724 | 0.000 | 0.047 | 0.946 | 2076 |
| REACTOME_LOSS_OF_FUNCTION_OF_SMAD2_3_IN_CANCER | 7 | 0.820 | 1.723 | 0.000 | 0.047 | 0.948 | 2290 |
| REACTOME_IL_6_TYPE_CYTOKINE_RECEPTOR_LIGAND_INTERACTIONS | 8 | 0.800 | 1.722 | 0.007 | 0.046 | 0.95 | 787 |
| REACTOME_ERK_MAPK_TARGETS | 21 | 0.612 | 1.716 | 0.005 | 0.049 | 0.966 | 2240 |
| REACTOME_TOLL_LIKE_RECEPTOR_TLR1_TLR2_CASCADE | 82 | 0.458 | 1.715 | 0.000 | 0.049 | 0.971 | 2920 |
| REACTOME_NEGATIVE_REGULATION_OF_TCF_DEPENDENT_SIGNALING_BY_WNT_LIGAND_ANTAGONISTS | 5 | 0.906 | 1.713 | 0.002 | 0.049 | 0.972 | 734 |
| REACTOME_REGULATION_OF_INSULIN_LIKE_GROWTH_FACTOR_IGF_TRANSPORT_AND_UPTAKE_BY_INSULIN_LIKE_GROWTH_FACTOR_BINDING_PROTEINS_IGFBPS_ | 65 | 0.483 | 1.710 | 0.003 | 0.051 | 0.975 | 2600 |
| REACTOME_SIGNAL_TRANSDUCTION_BY_L1 | 17 | 0.652 | 1.708 | 0.004 | 0.051 | 0.977 | 3513 |
| REACTOME_PLASMA_LIPOPROTEIN_REMODELING | 15 | 0.664 | 1.700 | 0.010 | 0.055 | 0.981 | 1539 |
| REACTOME_POST_CHAPERONIN_TUBULIN_FOLDING_PATHWAY | 17 | 0.643 | 1.698 | 0.008 | 0.055 | 0.981 | 1399 |
| REACTOME_INTEGRIN_SIGNALING | 21 | 0.594 | 1.696 | 0.013 | 0.056 | 0.982 | 2395 |
| REACTOME_GROWTH_HORMONE_RECEPTOR_SIGNALING | 19 | 0.619 | 1.694 | 0.009 | 0.057 | 0.984 | 399 |

| | | | | | | | |
|---|---|---|---|---|---|---|---|
| REACTOME_STRIATED_MUSCLE_CONTRACTION | 15 | 0.658 | 1.694 | 0.007 | 0.057 | 0.984 | 1569 |
| REACTOME_SIGNALING_BY_TGF_BETA_RECEPTOR_COMPLEX_IN_CANCER | 8 | 0.784 | 1.693 | 0.006 | 0.056 | 0.986 | 2290 |
| REACTOME_RESPONSE_TO_ELEVATED_PLATELET_CYTOSOLIC_CA2_ | 85 | 0.454 | 1.682 | 0.000 | 0.063 | 0.992 | 2704 |
| REACTOME_DISEASES_OF_SIGNAL_TRANSDUCTION_BY_GROWTH_FACTOR_RECEPTORS_AND_SECOND_MESSENGERS | 329 | 0.383 | 1.681 | 0.000 | 0.063 | 0.992 | 2607 |
| REACTOME_SIGNALING_BY_PDGF | 40 | 0.528 | 1.681 | 0.004 | 0.063 | 0.992 | 1707 |
| REACTOME_TYPE_I_HEMIDESMOSOME_ASSEMBLY | 9 | 0.747 | 1.678 | 0.011 | 0.064 | 0.993 | 1817 |
| REACTOME_MISSPLICED_LRP5_MUTANTS_HAVE_ENHANCED_BETA_CATENIN_DEPENDENT_SIGNALING | 4 | 0.936 | 1.675 | 0.004 | 0.065 | 0.993 | 734 |
| REACTOME_PLATELET_ACTIVATION_SIGNALING_AND_AGGREGATION | 167 | 0.416 | 1.675 | 0.000 | 0.065 | 0.993 | 3235 |
| REACTOME_PLASMA_LIPOPROTEIN_ASSEMBLY_REMODELING_AND_CLEARANCE | 47 | 0.501 | 1.673 | 0.000 | 0.065 | 0.995 | 1539 |
| REACTOME_P130CAS_LINKAGE_TO_MAPK_SIGNALING_FOR_INTEGRINS | 11 | 0.710 | 1.670 | 0.013 | 0.066 | 0.996 | 998 |
| REACTOME_ER_TO_GOLGI_ANTEROGRADE_TRANSPORT | 139 | 0.421 | 1.669 | 0.001 | 0.067 | 0.996 | 3709 |
| REACTOME_FOXO_MEDIATED_TRANSCRIPTION_OF_OXIDATIVE_STRESS_METABOLIC_AND_NEURONAL_GENES | 20 | 0.595 | 1.665 | 0.016 | 0.069 | 0.996 | 1765 |
| REACTOME_CLASS_A_1_RHODOPSIN_LIKE_RECEPTORS_ | 65 | 0.463 | 1.662 | 0.002 | 0.071 | 0.997 | 1669 |
| REACTOME_GENERATION_OF_SECOND_MESSENGER_MOLECULES | 13 | 0.673 | 1.659 | 0.017 | 0.072 | 0.997 | 1355 |
| REACTOME_TRANSLOCATION_OF_SLC2A4_GLUT4_TO_THE_PLASMA_MEMBRANE | 63 | 0.472 | 1.657 | 0.007 | 0.073 | 0.997 | 3523 |
| REACTOME_ACTIVATION_OF_BAD_AND_TRANSLOCATION_TO_MITOCHONDRIA_ | 15 | 0.637 | 1.656 | 0.026 | 0.073 | 0.998 | 3484 |
| REACTOME_RUNX2_REGULATES_BONE_DEVELOPMENT | 23 | 0.571 | 1.652 | 0.010 | 0.075 | 0.998 | 3195 |
| REACTOME_O_GLYCOSYLATION_OF_TSR_DOMAIN_CONTAINING_PROTEINS | 20 | 0.601 | 1.651 | 0.019 | 0.075 | 0.998 | 640 |
| REACTOME_CREB_PHOSPHORYLATION | 7 | 0.783 | 1.647 | 0.022 | 0.079 | 0.998 | 1267 |

| | | | | | | | |
|---|---|---|---|---|---|---|---|
| REACTOME_SIGNALING_BY_LEPTIN | 10 | 0.708 | 1.644 | 0.025 | 0.080 | 0.999 | 255 |
| REACTOME_GAB1_SIGNALOSOME | 12 | 0.694 | 1.640 | 0.020 | 0.083 | 0.999 | 1406 |
| REACTOME_CELL_CELL_COMMUNICATION | 84 | 0.441 | 1.640 | 0.003 | 0.082 | 0.999 | 1817 |
| REACTOME_TOLL_LIKE_RECEPTOR_10_TLR10_CASCADE | 77 | 0.460 | 1.635 | 0.003 | 0.086 | 0.999 | 2920 |
| REACTOME_COPI_MEDIATED_ANTEROGRADE_TRANSPORT | 88 | 0.437 | 1.633 | 0.003 | 0.087 | 1 | 3276 |
| REACTOME_PEPTIDE_LIGAND_BINDING_RECEPTORS | 37 | 0.513 | 1.632 | 0.015 | 0.087 | 1 | 2379 |
| REACTOME_DEFECTIVE_EXT2_CAUSES_EXOSTOSES_2 | 10 | 0.722 | 1.630 | 0.027 | 0.089 | 1 | 1511 |
| REACTOME_ACTIVATION_OF_THE_AP_1_FAMILY_OF_TRANSCRIPTION_FACTORS | 9 | 0.746 | 1.629 | 0.018 | 0.088 | 1 | 2887 |
| REACTOME_TOLL_LIKE_RECEPTOR_9_TLR9_CASCADE | 85 | 0.441 | 1.623 | 0.005 | 0.093 | 1 | 2920 |
| REACTOME_FCERI_MEDIATED_MAPK_ACTIVATION | 25 | 0.549 | 1.622 | 0.016 | 0.094 | 1 | 3335 |
| REACTOME_EGFR_DOWNREGULATION | 25 | 0.558 | 1.619 | 0.012 | 0.095 | 1 | 3855 |
| REACTOME_PLATELET_AGGREGATION_PLUG_FORMATION_ | 24 | 0.556 | 1.618 | 0.015 | 0.096 | 1 | 2395 |
| REACTOME_SIGNALING_BY_MAPK_MUTANTS | 7 | 0.768 | 1.615 | 0.017 | 0.098 | 1 | 2887 |
| REACTOME_CARDIAC_CONDUCTION | 65 | 0.461 | 1.614 | 0.002 | 0.098 | 1 | 2076 |
| REACTOME_TRANSFERRIN_ENDOCYTOSIS_AND_RECYCLING | 26 | 0.554 | 1.614 | 0.019 | 0.097 | 1 | 3330 |
| REACTOME_SIGNAL_ATTENUATION | 9 | 0.719 | 1.614 | 0.015 | 0.097 | 1 | 770 |
| REACTOME_P75NTR_NEGATIVELY_REGULATES_CELL_CYCLE_VIA_SC1 | 5 | 0.841 | 1.612 | 0.008 | 0.097 | 1 | 4 |
| REACTOME_CHYLOMICRON_ASSEMBLY | 3 | 0.967 | 1.609 | 0.000 | 0.100 | 1 | 112 |
| REACTOME_SIGNALING_BY_WNT_IN_CANCER | 30 | 0.534 | 1.607 | 0.005 | 0.101 | 1 | 2549 |
| REACTOME_NERVOUS_SYSTEM_DEVELOPMENT | 448 | 0.360 | 1.606 | 0.000 | 0.101 | 1 | 2159 |
| REACTOME_EGFR_INTERACTS_WITH_PHOSPHOLIPASE_C_GAMMA | 5 | 0.859 | 1.606 | 0.014 | 0.101 | 1 | 750 |
| REACTOME_NEUTROPHIL_DEGRANULATION | 342 | 0.364 | 1.603 | 0.000 | 0.102 | 1 | 3019 |
| REACTOME_TOLL_LIKE_RECEPTOR_4_TLR4_CASCADE | 106 | 0.412 | 1.600 | 0.000 | 0.105 | 1 | 3122 |
| REACTOME_CTLA4_INHIBITORY_SIGNALING | 18 | 0.595 | 1.600 | 0.024 | 0.104 | 1 | 3080 |

| | | | | | | | |
|---|---|---|---|---|---|---|---|
| REACTOME_COPI_INDEPENDENT_GOLGI_TO_ER_RETROGRADE_TRAFFIC | 41 | 0.496 | 1.599 | 0.010 | 0.104 | 1 | 2113 |
| REACTOME_INSULIN_RECEPTOR_SIGNALLING_CASCADE | 34 | 0.520 | 1.598 | 0.015 | 0.104 | 1 | 3012 |
| REACTOME_CELL_JUNCTION_ORGANIZATION | 59 | 0.466 | 1.598 | 0.005 | 0.104 | 1 | 1817 |
| REACTOME_GASTRIN_CREB_SIGNALLING_PATHWAY_VIA_PKC_AND_MAPK | 14 | 0.630 | 1.597 | 0.020 | 0.104 | 1 | 716 |
| REACTOME_SIGNALING_BY_FGFR1 | 33 | 0.520 | 1.597 | 0.012 | 0.104 | 1 | 2887 |
| REACTOME_REGULATION_OF_LOCALIZATION_OF_FOXO_TRANSCRIPTION_FACTORS | 12 | 0.662 | 1.594 | 0.023 | 0.105 | 1 | 4003 |
| REACTOME_RHO_GTPASES_ACTIVATE_ROCKS | 18 | 0.598 | 1.586 | 0.025 | 0.114 | 1 | 3499 |
| REACTOME_TRANSCRIPTIONAL_REGULATION_BY_THE_AP_2_TFAP2_FAMILY_OF_TRANSCRIPTION_FACTORS | 28 | 0.539 | 1.585 | 0.019 | 0.114 | 1 | 750 |
| REACTOME_SHC_MEDIATED_CASCADE_FGFR4 | 10 | 0.674 | 1.584 | 0.024 | 0.114 | 1 | 1208 |
| REACTOME_INTERLEUKIN_7_SIGNALING | 16 | 0.609 | 1.584 | 0.024 | 0.113 | 1 | 255 |
| REACTOME_INTERLEUKIN_10_SIGNALING | 18 | 0.597 | 1.582 | 0.022 | 0.114 | 1 | 763 |
| REACTOME_NCAM_SIGNALING_FOR_NEURITE_OUT_GROWTH | 34 | 0.506 | 1.582 | 0.019 | 0.113 | 1 | 2967 |
| REACTOME_CREB1_PHOSPHORYLATION_THROUGH_NMDA_RECEPTOR_MEDIATED_ACTIVATION_OF_RAS_SIGNALING | 22 | 0.553 | 1.582 | 0.009 | 0.113 | 1 | 2887 |
| REACTOME_MYD88_INDEPENDENT_TLR4_CASCADE_ | 90 | 0.418 | 1.580 | 0.006 | 0.114 | 1 | 3122 |
| REACTOME_SIGNALING_BY_VEGF | 86 | 0.420 | 1.580 | 0.008 | 0.113 | 1 | 3192 |
| REACTOME_FOXO_MEDIATED_TRANSCRIPTION | 55 | 0.454 | 1.576 | 0.015 | 0.117 | 1 | 3199 |
| REACTOME_CARGO_CONCENTRATION_IN_THE_ER | 31 | 0.515 | 1.575 | 0.016 | 0.117 | 1 | 2028 |
| REACTOME_EXTRA_NUCLEAR_ESTROGEN_SIGNALING | 52 | 0.472 | 1.575 | 0.007 | 0.117 | 1 | 2053 |
| REACTOME_DOWNREGULATION_OF_SMAD2_3_SMAD4_TRANSCRIPTIONAL_ACTIVITY | 23 | 0.549 | 1.574 | 0.029 | 0.117 | 1 | 3842 |
| REACTOME_SIGNALING_BY_MST1 | 5 | 0.817 | 1.573 | 0.020 | 0.117 | 1 | 1708 |
| REACTOME_TRAFFICKING_OF_AMPA_RECEPTORS | 18 | 0.591 | 1.573 | 0.027 | 0.116 | 1 | 2466 |
| REACTOME_ION_CHANNEL_TRANSPORT | 111 | 0.409 | 1.573 | 0.003 | 0.116 | 1 | 3082 |

| | | | | | | | |
|---|---|---|---|---|---|---|---|
| REACTOME_REVERSIBLE_HYDRATION_OF_CARBON_DIOXIDE | 7 | 0.755 | 1.572 | 0.020 | 0.116 | 1 | 2318 |
| REACTOME_COSTIMULATION_BY_THE_CD28_FAMILY | 44 | 0.471 | 1.570 | 0.020 | 0.117 | 1 | 3235 |
| REACTOME_SEMAPHORIN_INTERACTIONS | 51 | 0.460 | 1.567 | 0.015 | 0.120 | 1 | 1745 |
| REACTOME_GRB2_SOS_PROVIDES_LINKAGE_TO_MAPK_SIGNALING_FOR_INTEGRINS_ | 11 | 0.644 | 1.567 | 0.036 | 0.119 | 1 | 2029 |
| REACTOME_SIGNALLING_TO_ERKS | 30 | 0.513 | 1.567 | 0.027 | 0.119 | 1 | 3687 |
| REACTOME_RAB_GERANYLGERANYLATION | 48 | 0.466 | 1.563 | 0.012 | 0.122 | 1 | 3851 |
| REACTOME_REGULATION_OF_GENE_EXPRESSION_BY_HYPOXIA_INDUCIBLE_FACTOR | 9 | 0.701 | 1.562 | 0.029 | 0.122 | 1 | 1852 |
| REACTOME_RUNX3_REGULATES_CDKN1A_TRANSCRIPTION | 7 | 0.772 | 1.562 | 0.030 | 0.122 | 1 | 2084 |
| REACTOME_HDL_REMODELING | 4 | 0.879 | 1.561 | 0.014 | 0.122 | 1 | 1539 |
| REACTOME_RET_SIGNALING | 29 | 0.519 | 1.560 | 0.016 | 0.122 | 1 | 1470 |
| REACTOME_PHYSIOLOGICAL_FACTORS | 7 | 0.738 | 1.559 | 0.037 | 0.122 | 1 | 2076 |
| REACTOME_NEUROTRANSMITTER_RECEPTORS_AND_POSTSYNAPTIC_SIGNAL_TRANSMISSION | 109 | 0.407 | 1.558 | 0.002 | 0.123 | 1 | 2466 |
| REACTOME_TRANSPORT_TO_THE_GOLGI_AND_SUBSEQUENT_MODIFICATION | 159 | 0.384 | 1.556 | 0.000 | 0.124 | 1 | 3709 |
| REACTOME_CD163_MEDIATING_AN_ANTI_INFLAMMATORY_RESPONSE | 7 | 0.742 | 1.553 | 0.019 | 0.127 | 1 | 2962 |
| REACTOME_UPTAKE_AND_ACTIONS_OF_BACTERIAL_TOXINS | 23 | 0.531 | 1.552 | 0.026 | 0.128 | 1 | 4224 |
| REACTOME_CLATHRIN_MEDIATED_ENDOCYTOSIS | 116 | 0.403 | 1.551 | 0.002 | 0.127 | 1 | 4123 |
| REACTOME_ACTIVATION_OF_AMPK_DOWNSTREAM_OF_NMDARS | 20 | 0.566 | 1.551 | 0.019 | 0.127 | 1 | 1399 |
| REACTOME_SEMA4D_IN_SEMAPHORIN_SIGNALING | 23 | 0.551 | 1.551 | 0.032 | 0.127 | 1 | 3574 |
| REACTOME_SIGNALING_BY_TGFB_FAMILY_MEMBERS | 86 | 0.418 | 1.550 | 0.008 | 0.126 | 1 | 3842 |
| REACTOME_INFECTION_WITH_MYCOBACTERIUM_TUBERCULOSIS | 23 | 0.541 | 1.548 | 0.030 | 0.128 | 1 | 3275 |
| REACTOME_DISEASES_ASSOCIATED_WITH_GLYCOSAMINOGLYCAN_METABOLISM | 27 | 0.516 | 1.544 | 0.024 | 0.132 | 1 | 1541 |
| REACTOME_SIGNALING_BY_TGF_BETA_RECEPTOR_COMPLEX | 72 | 0.434 | 1.543 | 0.013 | 0.132 | 1 | 3842 |
| REACTOME_FGFR2_MUTANT_RECEPTOR_ACTIVATION | 19 | 0.562 | 1.540 | 0.030 | 0.135 | 1 | 2715 |

| | | | | | | | |
|---|---|---|---|---|---|---|---|
| REACTOME_GPVI_MEDIATED_ACTIVATION_CASCADE | 24 | 0.521 | 1.539 | 0.021 | 0.135 | 1 | 3235 |
| REACTOME_HS_GAG_DEGRADATION | 17 | 0.586 | 1.539 | 0.030 | 0.135 | 1 | 1511 |
| REACTOME_MET_RECEPTOR_ACTIVATION | 4 | 0.866 | 1.539 | 0.014 | 0.135 | 1 | 1708 |
| REACTOME_SYNAPTIC_ADHESION_LIKE_MOLECULES | 14 | 0.603 | 1.538 | 0.040 | 0.135 | 1 | 2639 |
| REACTOME_DISEASES_OF_GLYCOSYLATION | 97 | 0.409 | 1.537 | 0.009 | 0.136 | 1 | 1541 |
| REACTOME_INTERLEUKIN_6_SIGNALING | 10 | 0.656 | 1.534 | 0.048 | 0.138 | 1 | 3168 |
| REACTOME_NEGATIVE_REGULATION_OF_FGFR4_SIGNALING | 21 | 0.549 | 1.534 | 0.026 | 0.137 | 1 | 2887 |
| REACTOME_SIGNALING_BY_EGFR_IN_CANCER | 20 | 0.550 | 1.534 | 0.043 | 0.137 | 1 | 770 |
| REACTOME_SIGNALING_BY_PTK6 | 42 | 0.478 | 1.534 | 0.015 | 0.136 | 1 | 2804 |
| REACTOME_SHC_MEDIATED_CASCADE_FGFR3 | 9 | 0.680 | 1.534 | 0.039 | 0.136 | 1 | 1208 |
| REACTOME_RESPONSE_OF_MTB_TO_PHAGOCYTOSIS | 21 | 0.554 | 1.533 | 0.041 | 0.136 | 1 | 3275 |
| REACTOME_SIGNALING_BY_GPCR | 314 | 0.351 | 1.530 | 0.000 | 0.138 | 1 | 1725 |
| REACTOME_MAP2K_AND_MAPK_ACTIVATION | 35 | 0.478 | 1.530 | 0.035 | 0.137 | 1 | 3589 |
| REACTOME_OLFACTORY_SIGNALING_PATHWAY | 8 | 0.712 | 1.530 | 0.031 | 0.137 | 1 | 308 |
| REACTOME_FORMATION_OF_FIBRIN_CLOT_CLOTTING_CASCADE_ | 18 | 0.565 | 1.528 | 0.054 | 0.139 | 1 | 301 |
| REACTOME_ION_TRANSPORT_BY_P_TYPE_ATPASES | 36 | 0.484 | 1.527 | 0.040 | 0.140 | 1 | 1306 |
| REACTOME_IONOTROPIC_ACTIVITY_OF_KAINATE_RECEPTORS | 6 | 0.771 | 1.526 | 0.025 | 0.140 | 1 | 2305 |
| REACTOME_NEGATIVE_REGULATION_OF_FGFR2_SIGNALING | 20 | 0.550 | 1.522 | 0.050 | 0.144 | 1 | 2887 |
| REACTOME_FGFR3B_LIGAND_BINDING_AND_ACTIVATION | 3 | 0.905 | 1.518 | 0.020 | 0.149 | 1 | 1208 |
| REACTOME_CD28_DEPENDENT_VAV1_PATHWAY | 8 | 0.693 | 1.517 | 0.035 | 0.150 | 1 | 3687 |
| REACTOME_TFAP2_AP_2_FAMILY_REGULATES_TRANSCRIPTION_OF_GROWTH_FACTORS_AND_THEIR_RECEPTORS | 7 | 0.728 | 1.513 | 0.048 | 0.153 | 1 | 750 |
| REACTOME_FGFRL1_MODULATION_OF_FGFR1_SIGNALING | 5 | 0.786 | 1.510 | 0.028 | 0.156 | 1 | 1108 |
| REACTOME_ACETYLCHOLINE_BINDING_AND_DOWNSTREAM_EVENTS | 5 | 0.799 | 1.508 | 0.030 | 0.159 | 1 | 236 |
| REACTOME_AKT_PHOSPHORYLATES_TARGETS_IN_THE_NUCLEUS | 10 | 0.664 | 1.508 | 0.053 | 0.158 | 1 | 714 |

| | | | | | | | |
|---|---|---|---|---|---|---|---|
| REACTOME_METABOLISM_OF_CARBOHYDRATES | 221 | 0.357 | 1.508 | 0.000 | 0.157 | 1 | 2265 |
| REACTOME_NEGATIVE_REGULATION_OF_FGFR3_SIGNALING | 20 | 0.553 | 1.506 | 0.036 | 0.158 | 1 | 2887 |
| REACTOME_PRESYNAPTIC_DEPOLARIZATION_AND_CALCIUM_CHANNEL_OPENING | 4 | 0.836 | 1.504 | 0.022 | 0.160 | 1 | 210 |
| REACTOME_ESR_MEDIATED_SIGNALING | 142 | 0.375 | 1.503 | 0.003 | 0.161 | 1 | 2057 |
| REACTOME_DEFECTS_OF_CONTACT_ACTIVATION_SYSTEM_CAS_AND_KALLIKREIN_KININ_SYSTEM_KKS_ | 8 | 0.697 | 1.502 | 0.055 | 0.161 | 1 | 892 |
| REACTOME_TRANSMISSION_ACROSS_CHEMICAL_SYNAPSES | 139 | 0.377 | 1.498 | 0.006 | 0.165 | 1 | 2466 |
| REACTOME_PECAM1_INTERACTIONS | 10 | 0.647 | 1.498 | 0.059 | 0.165 | 1 | 782 |
| REACTOME_POTASSIUM_CHANNELS | 33 | 0.494 | 1.496 | 0.024 | 0.166 | 1 | 1819 |
| REACTOME_RUNX2_REGULATES_OSTEOBLAST_DIFFERENTIATION | 18 | 0.555 | 1.494 | 0.047 | 0.169 | 1 | 3195 |
| REACTOME_DISEASES_ASSOCIATED_WITH_O_GLYCOSYLATION_OF_PROTEINS | 36 | 0.475 | 1.491 | 0.043 | 0.171 | 1 | 1369 |
| REACTOME_COPI_DEPENDENT_GOLGI_TO_ER_RETROGRADE_TRAFFIC | 88 | 0.401 | 1.491 | 0.014 | 0.171 | 1 | 2894 |
| REACTOME_CASPASE_ACTIVATION_VIA_DEPENDENCE_RECEPTORS_IN_THE_ABSENCE_OF_LIGAND | 7 | 0.713 | 1.491 | 0.043 | 0.171 | 1 | 464 |
| REACTOME_COPII_MEDIATED_VESICLE_TRANSPORT | 66 | 0.423 | 1.490 | 0.017 | 0.170 | 1 | 3709 |
| REACTOME_ERYTHROPOIETIN_ACTIVATES_RAS | 13 | 0.596 | 1.487 | 0.048 | 0.175 | 1 | 770 |
| REACTOME_LGI_ADAM_INTERACTIONS | 6 | 0.756 | 1.484 | 0.039 | 0.178 | 1 | 210 |
| REACTOME_TFAP2_AP_2_FAMILY_REGULATES_TRANSCRIPTION_OF_CELL_CYCLE_FACTORS | 5 | 0.774 | 1.483 | 0.055 | 0.178 | 1 | 1978 |
| REACTOME_IRON_UPTAKE_AND_TRANSPORT | 47 | 0.451 | 1.483 | 0.023 | 0.177 | 1 | 3330 |
| REACTOME_TP53_REGULATES_TRANSCRIPTION_OF_GENES_INVOLVED_IN_CYTOCHROME_C_RELEASE | 18 | 0.549 | 1.480 | 0.041 | 0.181 | 1 | 704 |
| REACTOME_CATION_COUPLED_CHLORIDE_COTRANSPORTERS | 4 | 0.839 | 1.479 | 0.049 | 0.182 | 1 | 1126 |
| REACTOME_NEGATIVE_REGULATION_OF_FGFR1_SIGNALING | 20 | 0.539 | 1.477 | 0.043 | 0.183 | 1 | 2887 |

| | | | | | | | |
|---|---|---|---|---|---|---|---|
| REACTOME_RUNX3_REGULATES_YAP1_MEDIATED_TRANSCRIPTION | 8 | 0.673 | 1.477 | 0.062 | 0.182 | 1 | 2313 |
| REACTOME_DEFECTIVE_CHST14_CAUSES_EDS_MUSCULOCONTRACTURAL_TYPE | 3 | 0.879 | 1.477 | 0.033 | 0.182 | 1 | 1541 |
| REACTOME_EXTRINSIC_PATHWAY_OF_FIBRIN_CLOT_FORMATION | 4 | 0.823 | 1.476 | 0.046 | 0.183 | 1 | 301 |
| REACTOME_SIGNALING_BY_FGFR | 65 | 0.422 | 1.473 | 0.031 | 0.186 | 1 | 3842 |
| REACTOME_LISTERIA_MONOCYTOGENES_ENTRY_INTO_HOST_CELLS | 18 | 0.550 | 1.473 | 0.061 | 0.186 | 1 | 3855 |
| REACTOME_P75NTR_REGULATES_AXONOGENESIS | 5 | 0.768 | 1.472 | 0.056 | 0.186 | 1 | 1327 |
| REACTOME_ERKS_ARE_INACTIVATED | 13 | 0.587 | 1.471 | 0.085 | 0.186 | 1 | 2887 |
| REACTOME_EPH_EPHRIN_SIGNALING | 79 | 0.400 | 1.470 | 0.014 | 0.186 | 1 | 3499 |
| REACTOME_INTERLEUKIN_2_FAMILY_SIGNALING | 26 | 0.492 | 1.468 | 0.053 | 0.189 | 1 | 3342 |
| REACTOME_CARBOXYTERMINAL_POST_TRANSLATIONAL_MODIFICATIONS_OF_TUBULIN | 26 | 0.498 | 1.466 | 0.033 | 0.191 | 1 | 2113 |
| REACTOME_PTK6_PROMOTES_HIF1A_STABILIZATION | 3 | 0.900 | 1.463 | 0.024 | 0.194 | 1 | 860 |
| REACTOME_UPTAKE_AND_FUNCTION_OF_ANTHRAX_TOXINS | 11 | 0.623 | 1.462 | 0.057 | 0.196 | 1 | 3513 |
| REACTOME_EPHB_MEDIATED_FORWARD_SIGNALING | 39 | 0.451 | 1.462 | 0.035 | 0.195 | 1 | 3499 |
| REACTOME_SIGNALING_BY_EGFR | 42 | 0.450 | 1.458 | 0.039 | 0.200 | 1 | 4119 |
| REACTOME_RUNX3_REGULATES_P14_ARF | 10 | 0.637 | 1.455 | 0.068 | 0.204 | 1 | 3249 |
| REACTOME_INTERLEUKIN_12_FAMILY_SIGNALING | 42 | 0.440 | 1.454 | 0.044 | 0.204 | 1 | 3443 |
| REACTOME_L1CAM_INTERACTIONS | 84 | 0.402 | 1.452 | 0.022 | 0.206 | 1 | 2113 |
| REACTOME_SIGNALING_BY_FGFR4 | 30 | 0.487 | 1.447 | 0.049 | 0.213 | 1 | 2887 |
| REACTOME_DEFECTIVE_CHST3_CAUSES_SEDCJD | 3 | 0.879 | 1.445 | 0.038 | 0.216 | 1 | 1541 |
| REACTOME_LEISHMANIA_INFECTION | 122 | 0.369 | 1.442 | 0.017 | 0.220 | 1 | 3050 |
| REACTOME_FCGAMMA_RECEPTOR_FCGR_DEPENDENT_PHAGOCYTOSIS | 71 | 0.402 | 1.441 | 0.016 | 0.220 | 1 | 3192 |
| REACTOME_RUNX1_REGULATES_TRANSCRIPTION_OF_GENES_INVOLVED_IN_DIFFERENTIATION_OF_MYELOID_CELLS | 5 | 0.745 | 1.438 | 0.071 | 0.224 | 1 | 3249 |

| | | | | | | | |
|---|---|---|---|---|---|---|---|
| REACTOME_TRANSCRIPTIONAL_ACTIVITY_OF_SMAD2_SMAD3_SMAD4_HETEROTRIMER | 43 | 0.434 | 1.437 | 0.041 | 0.224 | 1 | 3842 |
| REACTOME_SIGNALING_BY_TYPE_1_INSULIN_LIKE_GROWTH_FACTOR_1_RECEPTOR_IGF1R_ | 31 | 0.468 | 1.437 | 0.064 | 0.224 | 1 | 1997 |
| REACTOME_VITAMIN_C_ASCORBATE_METABOLISM | 7 | 0.691 | 1.431 | 0.066 | 0.233 | 1 | 2297 |
| REACTOME_ADVANCED_GLYCOSYLATION_ENDPRODUCT_RECEPTOR_SIGNALING | 10 | 0.623 | 1.430 | 0.077 | 0.233 | 1 | 3602 |
| REACTOME_RHO_GTPASE_EFFECTORS | 241 | 0.338 | 1.429 | 0.010 | 0.234 | 1 | 3589 |
| REACTOME_PLATELET_SENSITIZATION_BY_LDL | 13 | 0.580 | 1.428 | 0.103 | 0.235 | 1 | 3235 |
| REACTOME_SEMA4D_INDUCED_CELL_MIGRATION_AND_GROWTH_CONE_COLLAPSE | 19 | 0.519 | 1.428 | 0.084 | 0.234 | 1 | 3574 |
| REACTOME_REGULATION_OF_CYTOSKELETAL_REMODELING_AND_CELL_SPREADING_BY_IPP_COMPLEX_COMPONENTS | 7 | 0.679 | 1.427 | 0.099 | 0.234 | 1 | 3882 |
| REACTOME_SIGNALING_BY_MET | 63 | 0.404 | 1.427 | 0.040 | 0.234 | 1 | 3855 |
| REACTOME_BIOSYNTHESIS_OF_MARESINS | 2 | 0.964 | 1.426 | 0.008 | 0.234 | 1 | 243 |
| REACTOME_AGGREPHAGY | 32 | 0.467 | 1.424 | 0.061 | 0.236 | 1 | 2317 |
| REACTOME_NCAM1_INTERACTIONS | 15 | 0.552 | 1.424 | 0.090 | 0.236 | 1 | 1511 |
| REACTOME_ONCOGENIC_MAPK_SIGNALING | 74 | 0.394 | 1.423 | 0.023 | 0.237 | 1 | 2887 |
| REACTOME_ADRENOCEPTORS | 2 | 0.948 | 1.420 | 0.022 | 0.241 | 1 | 172 |
| REACTOME_RHO_GTPASES_ACTIVATE_PAKS | 19 | 0.519 | 1.419 | 0.079 | 0.242 | 1 | 3546 |
| REACTOME_HSP90_CHAPERONE_CYCLE_FOR_STEROID_HORMONE_RECEPTORS_SHR_ | 44 | 0.432 | 1.419 | 0.064 | 0.241 | 1 | 2113 |
| REACTOME_VISUAL_PHOTOTRANSDUCTION | 47 | 0.427 | 1.416 | 0.048 | 0.245 | 1 | 1511 |
| REACTOME_ION_HOMEOSTASIS | 32 | 0.458 | 1.415 | 0.055 | 0.246 | 1 | 2074 |
| REACTOME_DEFECTIVE_FACTOR_VIII_CAUSES_HEMOPHILIA_A | 6 | 0.704 | 1.413 | 0.089 | 0.248 | 1 | 892 |
| REACTOME_ABERRANT_REGULATION_OF_MITOTIC_G1_S_TRANSITION_IN_CANCER_DUE_TO_RB1_DEFECTS | 16 | 0.544 | 1.412 | 0.071 | 0.249 | 1 | 1052 |
| REACTOME_RUNX2_REGULATES_GENES_INVOLVED_IN_CELL_MIGRATION | 6 | 0.706 | 1.407 | 0.092 | 0.256 | 1 | 3080 |
| REACTOME_ACTIVATION_OF_RAC1 | 10 | 0.617 | 1.406 | 0.110 | 0.256 | 1 | 3342 |

| | | | | | | | |
|---|---|---|---|---|---|---|---|
| REACTOME_TRANSPORT_OF_FATTY_ACIDS | 2 | 0.955 | 1.406 | 0.021 | 0.257 | 1 | 383 |
| REACTOME_SYNTHESIS_OF_ACTIVE_UBIQUITIN_ROLES_OF_E1_AND_E2_ENZYMES | 30 | 0.469 | 1.405 | 0.066 | 0.256 | 1 | 4023 |
| REACTOME_SIGNALING_BY_NUCLEAR_RECEPTORS | 195 | 0.339 | 1.404 | 0.013 | 0.257 | 1 | 2057 |
| REACTOME_IRS_MEDIATED_SIGNALLING | 29 | 0.462 | 1.402 | 0.063 | 0.260 | 1 | 1997 |
| REACTOME_GOLGI_TO_ER_RETROGRADE_TRANSPORT | 118 | 0.361 | 1.401 | 0.030 | 0.260 | 1 | 1883 |
| REACTOME_NADE_MODULATES_DEATH_SIGNALLING | 5 | 0.751 | 1.400 | 0.083 | 0.261 | 1 | 4 |
| REACTOME_FGFR2_LIGAND_BINDING_AND_ACTIVATION | 5 | 0.736 | 1.400 | 0.092 | 0.260 | 1 | 1208 |
| REACTOME_EARLY_PHASE_OF_HIV_LIFE_CYCLE | 13 | 0.575 | 1.396 | 0.093 | 0.266 | 1 | 2792 |
| REACTOME_PHOSPHOLIPASE_C_MEDIATED_CASCADE_FGFR4 | 5 | 0.743 | 1.395 | 0.090 | 0.268 | 1 | 1208 |
| REACTOME_PI_3K_CASCADE_FGFR4 | 9 | 0.627 | 1.392 | 0.099 | 0.271 | 1 | 1208 |
| REACTOME_SMAD2_SMAD3_SMAD4_HETEROTRIMER_REGULATES_TRANSCRIPTION | 31 | 0.455 | 1.391 | 0.067 | 0.272 | 1 | 2159 |
| REACTOME_ACTIVATION_OF_THE_TFAP2_AP_2_FAMILY_OF_TRANSCRIPTION_FACTORS | 9 | 0.615 | 1.391 | 0.091 | 0.271 | 1 | 234 |
| REACTOME_REGULATION_OF_GLYCOLYSIS_BY_FRUCTOSE_2_6_BISPHOSPHATE_METABOLISM | 10 | 0.603 | 1.390 | 0.103 | 0.271 | 1 | 2960 |
| REACTOME_SIGNALING_BY_FGFR2 | 57 | 0.403 | 1.389 | 0.047 | 0.272 | 1 | 4118 |
| REACTOME_SHC_MEDIATED_CASCADE_FGFR1 | 9 | 0.621 | 1.386 | 0.100 | 0.276 | 1 | 4118 |
| REACTOME_COMMON_PATHWAY_OF_FIBRIN_CLOT_FORMATION | 11 | 0.576 | 1.386 | 0.109 | 0.275 | 1 | 301 |
| REACTOME_RSK_ACTIVATION | 7 | 0.663 | 1.386 | 0.094 | 0.274 | 1 | 2887 |
| REACTOME_FGFR1_LIGAND_BINDING_AND_ACTIVATION | 3 | 0.843 | 1.385 | 0.058 | 0.275 | 1 | 1997 |
| REACTOME_RUNX2_REGULATES_CHONDROCYTE_MATURATION | 3 | 0.832 | 1.385 | 0.078 | 0.275 | 1 | 1966 |
| REACTOME_DERMATAN_SULFATE_BIOSYNTHESIS | 5 | 0.732 | 1.384 | 0.101 | 0.276 | 1 | 1541 |
| REACTOME_TP53_REGULATES_TRANSCRIPTION_OF_GENES_INVOLVED_IN_G1_CELL_CYCLE_ARREST | 13 | 0.551 | 1.384 | 0.113 | 0.275 | 1 | 462 |
| REACTOME_INTRACELLULAR_SIGNALING_BY_SECOND_MESSENGERS | 247 | 0.326 | 1.383 | 0.006 | 0.276 | 1 | 2742 |
| REACTOME_EPHA_MEDIATED_GROWTH_CONE_COLLAPSE | 23 | 0.481 | 1.382 | 0.091 | 0.277 | 1 | 1916 |

| | | | | | | | |
|---|---|---|---|---|---|---|---|
| REACTOME_TBC_RABGAPS | 39 | 0.421 | 1.381 | 0.082 | 0.276 | 1 | 2814 |
| REACTOME_TOLL_LIKE_RECEPTOR_CASCADES | 121 | 0.355 | 1.381 | 0.031 | 0.277 | 1 | 2317 |
| REACTOME_CHYLOMICRON_CLEARANCE | 4 | 0.763 | 1.380 | 0.102 | 0.276 | 1 | 1141 |
| REACTOME_PI_3K_CASCADE_FGFR2 | 8 | 0.624 | 1.377 | 0.115 | 0.282 | 1 | 1208 |
| REACTOME_SIGNALING_BY_ACTIVIN | 10 | 0.592 | 1.375 | 0.105 | 0.284 | 1 | 1697 |
| REACTOME_SPHINGOLIPID_DE_NOVO_BIOSYNTHESIS | 35 | 0.440 | 1.375 | 0.072 | 0.283 | 1 | 2947 |
| REACTOME_SIGNALING_BY_RHO_GTPASES | 348 | 0.313 | 1.374 | 0.001 | 0.283 | 1 | 2632 |
| REACTOME_SUMOYLATION_OF_TRANSCRIPTION_COFACTORS | 43 | 0.412 | 1.374 | 0.064 | 0.283 | 1 | 3604 |
| REACTOME_SIGNALLING_TO_RAS | 17 | 0.528 | 1.373 | 0.102 | 0.284 | 1 | 4526 |
| REACTOME_ACTIVATION_OF_MATRIX_METALLOPROTEINASES | 14 | 0.550 | 1.370 | 0.118 | 0.288 | 1 | 1528 |
| REACTOME_PI5P_REGULATES_TP53_ACETYLATION | 9 | 0.615 | 1.369 | 0.117 | 0.288 | 1 | 2725 |
| REACTOME_RUNX1_AND_FOXP3_CONTROL_THE_DEVELOPMENT_OF_REGULATORY_T_LYMPHOCYTES_TREGS_ | 5 | 0.731 | 1.369 | 0.106 | 0.288 | 1 | 3426 |
| REACTOME_IRS_ACTIVATION | 4 | 0.756 | 1.369 | 0.106 | 0.287 | 1 | 570 |
| REACTOME_NUCLEAR_ENVELOPE_NE_REASSEMBLY | 68 | 0.382 | 1.368 | 0.043 | 0.288 | 1 | 2949 |
| REACTOME_CARGO_RECOGNITION_FOR_CLATHRIN_MEDIATED_ENDOCYTOSIS | 78 | 0.376 | 1.367 | 0.052 | 0.288 | 1 | 3953 |
| REACTOME_DOWNSTREAM_SIGNALING_OF_ACTIVATED_FGFR4 | 16 | 0.511 | 1.366 | 0.101 | 0.290 | 1 | 1208 |
| REACTOME_CLASS_B_2_SECRETIN_FAMILY_RECEPTORS_ | 38 | 0.424 | 1.365 | 0.073 | 0.291 | 1 | 1257 |
| REACTOME_CROSS_PRESENTATION_OF_PARTICULATE_EXOGENOUS_ANTIGENS_PHAGOSOMES_ | 3 | 0.813 | 1.364 | 0.101 | 0.291 | 1 | 1967 |
| REACTOME_INTERLEUKIN_21_SIGNALING | 6 | 0.686 | 1.358 | 0.132 | 0.302 | 1 | 2771 |
| REACTOME_SIGNALING_BY_NTRK3_TRKC_ | 15 | 0.530 | 1.357 | 0.110 | 0.303 | 1 | 770 |
| REACTOME_SIGNALING_BY_NODAL | 12 | 0.573 | 1.356 | 0.112 | 0.303 | 1 | 1367 |
| REACTOME_EGR2_AND_SOX10_MEDIATED_INITIATION_OF_SCHWANN_CELL_MYELINATION | 24 | 0.472 | 1.352 | 0.101 | 0.309 | 1 | 1320 |
| REACTOME_SUPPRESSION_OF_APOPTOSIS | 6 | 0.681 | 1.352 | 0.122 | 0.309 | 1 | 3275 |
| REACTOME_PHASE_0_RAPID_DEPOLARISATION | 16 | 0.517 | 1.351 | 0.122 | 0.310 | 1 | 2906 |

| | | | | | | | |
|---|---|---|---|---|---|---|---|
| REACTOME_CHYLOMICRON_REMODELING | 1 | 0.992 | 1.350 | 0.020 | 0.311 | 1 | 99 |
| REACTOME_PI_3K_CASCADE_FGFR3 | 8 | 0.632 | 1.349 | 0.109 | 0.311 | 1 | 1208 |
| REACTOME_REGULATION_OF_IFNG_SIGNALING | 13 | 0.552 | 1.348 | 0.110 | 0.313 | 1 | 4537 |
| REACTOME_INTRINSIC_PATHWAY_OF_FIBRIN_CLOT_FORMATION | 10 | 0.584 | 1.346 | 0.144 | 0.316 | 1 | 301 |
| REACTOME_METABOLISM_OF_FAT_SOLUBLE_VITAMINS | 27 | 0.459 | 1.345 | 0.120 | 0.316 | 1 | 1511 |
| REACTOME_DEFECTIVE_CHSY1_CAUSES_TPBS | 3 | 0.816 | 1.344 | 0.097 | 0.318 | 1 | 1541 |
| REACTOME_NR1H3_NR1H2_REGULATE_GENE_EXPRESSION_LINKED_TO_CHOLESTEROL_TRANSPORT_AND_EFFLUX | 31 | 0.434 | 1.343 | 0.090 | 0.318 | 1 | 1671 |
| REACTOME_TRANSPORT_OF_SMALL_MOLECULES | 455 | 0.298 | 1.342 | 0.001 | 0.320 | 1 | 1863 |
| REACTOME_INTERLEUKIN_2_SIGNALING | 7 | 0.651 | 1.341 | 0.130 | 0.320 | 1 | 971 |
| REACTOME_KINESINS | 50 | 0.398 | 1.341 | 0.062 | 0.320 | 1 | 1025 |
| REACTOME_P38MAPK_EVENTS | 13 | 0.542 | 1.339 | 0.112 | 0.322 | 1 | 4927 |
| REACTOME_REGULATION_OF_COMMISSURAL_AXON_PATHFINDING_BY_SLIT_AND_ROBO | 4 | 0.749 | 1.333 | 0.113 | 0.332 | 1 | 2948 |
| REACTOME_SYNTHESIS_OF_PIPS_AT_THE_PLASMA_MEMBRANE | 45 | 0.403 | 1.333 | 0.085 | 0.331 | 1 | 2725 |
| REACTOME_SIGNALING_BY_ERBB4 | 38 | 0.419 | 1.333 | 0.100 | 0.332 | 1 | 1375 |
| REACTOME_MHC_CLASS_II_ANTIGEN_PRESENTATION | 95 | 0.358 | 1.331 | 0.061 | 0.334 | 1 | 2913 |
| REACTOME_FGFR2C_LIGAND_BINDING_AND_ACTIVATION | 2 | 0.905 | 1.328 | 0.080 | 0.339 | 1 | 1208 |
| REACTOME_ACTIVATION_OF_THE_PHOTOTRANSDUCTION_CASCADE | 2 | 0.867 | 1.327 | 0.117 | 0.339 | 1 | 254 |
| REACTOME_ACTIVATED_NTRK3_SIGNALS_THROUGH_RAS | 7 | 0.636 | 1.327 | 0.153 | 0.340 | 1 | 770 |
| REACTOME_INLA_MEDIATED_ENTRY_OF_LISTERIA_MONOCYTOGENES_INTO_HOST_CELLS | 9 | 0.597 | 1.326 | 0.147 | 0.339 | 1 | 3842 |
| REACTOME_UPTAKE_AND_FUNCTION_OF_DIPHTHERIA_TOXIN | 6 | 0.676 | 1.326 | 0.140 | 0.338 | 1 | 1562 |
| REACTOME_PHASE_3_RAPID_REPOLARISATION | 4 | 0.745 | 1.326 | 0.137 | 0.338 | 1 | 446 |
| REACTOME_PRE_NOTCH_PROCESSING_IN_GOLGI | 16 | 0.500 | 1.325 | 0.141 | 0.338 | 1 | 2606 |
| REACTOME_NEGATIVE_REGULATION_OF_MET_ACTIVITY | 18 | 0.482 | 1.325 | 0.146 | 0.337 | 1 | 4537 |
| REACTOME_RUNX1_REGULATES_GENES_INVOLVED_IN_MEGAK | 44 | 0.396 | 1.323 | 0.107 | 0.340 | 1 | 1204 |

| | | | | | | | |
|---|---|---|---|---|---|---|---|
| ARYOCYTE_DIFFERENTIATION_AND_PLATELET_FUNCTION | | | | | | | |
| REACTOME_ACTIVATED_TAK1_MEDIATES_P38_MAPK_ACTIVATION | 23 | 0.460 | 1.322 | 0.115 | 0.342 | 1 | 2889 |
| REACTOME_SIGNALING_BY_FGFR3 | 30 | 0.436 | 1.320 | 0.114 | 0.344 | 1 | 2887 |
| REACTOME_LAMININ_INTERACTIONS | 22 | 0.457 | 1.320 | 0.124 | 0.344 | 1 | 1462 |
| REACTOME_SUPPRESSION_OF_PHAGOSOMAL_MATURATION | 12 | 0.547 | 1.318 | 0.144 | 0.346 | 1 | 2653 |
| REACTOME_INFLAMMASOMES | 12 | 0.562 | 1.316 | 0.148 | 0.349 | 1 | 2185 |
| REACTOME_DEFECTIVE_FACTOR_IX_CAUSES_HEMOPHILIA_B | 3 | 0.790 | 1.316 | 0.135 | 0.349 | 1 | 301 |
| REACTOME_PTK6_REGULATES_RHO_GTPASES_RAS_GTPASE_AND_MAP_KINASES | 13 | 0.529 | 1.315 | 0.156 | 0.350 | 1 | 4118 |
| REACTOME_ASSEMBLY_OF_ACTIVE_LPL_AND_LIPC_LIPASE_COMPLEXES | 11 | 0.563 | 1.315 | 0.148 | 0.349 | 1 | 1248 |
| REACTOME_PHOSPHOLIPASE_C_MEDIATED_CASCADE_FGFR2 | 4 | 0.751 | 1.313 | 0.149 | 0.351 | 1 | 1208 |
| REACTOME_FRS_MEDIATED_FGFR4_SIGNALING | 12 | 0.536 | 1.311 | 0.151 | 0.355 | 1 | 1208 |
| REACTOME_SIGNALING_BY_FGFR_IN_DISEASE | 48 | 0.390 | 1.310 | 0.100 | 0.355 | 1 | 3012 |
| REACTOME_RAS_ACTIVATION_UPON_CA2_INFLUX_THROUGH_NMDA_RECEPTOR | 14 | 0.509 | 1.308 | 0.140 | 0.358 | 1 | 2027 |
| REACTOME_INTERLEUKIN_20_FAMILY_SIGNALING | 16 | 0.486 | 1.307 | 0.140 | 0.359 | 1 | 146 |
| REACTOME_DOWNSTREAM_SIGNALING_OF_ACTIVATED_FGFR2 | 15 | 0.503 | 1.306 | 0.135 | 0.361 | 1 | 1208 |
| REACTOME_BIOSYNTHESIS_OF_MARESIN_LIKE_SPMS | 1 | 0.981 | 1.306 | 0.045 | 0.360 | 1 | 243 |
| REACTOME_CREATINE_METABOLISM | 5 | 0.689 | 1.305 | 0.169 | 0.361 | 1 | 889 |
| REACTOME_DOWNSTREAM_SIGNALING_OF_ACTIVATED_FGFR3 | 15 | 0.509 | 1.303 | 0.133 | 0.363 | 1 | 1208 |
| REACTOME_MYOGENESIS | 16 | 0.501 | 1.303 | 0.127 | 0.362 | 1 | 3463 |
| REACTOME_GAP_JUNCTION_DEGRADATION | 11 | 0.550 | 1.303 | 0.156 | 0.361 | 1 | 2601 |
| REACTOME_MET_ACTIVATES_RAS_SIGNALING | 9 | 0.585 | 1.303 | 0.169 | 0.361 | 1 | 4118 |
| REACTOME_RHO_GTPASE_CYCLE | 117 | 0.337 | 1.301 | 0.051 | 0.363 | 1 | 2102 |
| REACTOME_NUCLEAR_SIGNALING_BY_ERBB4 | 20 | 0.478 | 1.300 | 0.147 | 0.364 | 1 | 1375 |
| REACTOME_PROTEIN_FOLDING | 75 | 0.361 | 1.300 | 0.078 | 0.363 | 1 | 3770 |
| REACTOME_NEF_AND_SIGNAL_TRANSDUCTION | 4 | 0.726 | 1.299 | 0.156 | 0.365 | 1 | 2981 |

| | | | | | | | |
|---|---|---|---|---|---|---|---|
| REACTOME_THYROXINE_BIOSYNTHESIS | 1 | 0.965 | 1.296 | 0.065 | 0.370 | 1 | 443 |
| REACTOME_SIGNALING_BY_MODERATE_KINASE_ACTIVITY_BRAF_MUTANTS | 38 | 0.407 | 1.296 | 0.128 | 0.370 | 1 | 3589 |
| REACTOME_SUMOYLATION_OF_TRANSCRIPTION_FACTORS | 19 | 0.466 | 1.296 | 0.134 | 0.369 | 1 | 3483 |
| REACTOME_ACTIVATED_NTRK3_SIGNALS_THROUGH_PI3K | 5 | 0.704 | 1.296 | 0.164 | 0.368 | 1 | 226 |
| REACTOME_RUNX1_REGULATES_TRANSCRIPTION_OF_GENES_INVOLVED_IN_DIFFERENTIATION_OF_KERATINOCYTES | 7 | 0.615 | 1.295 | 0.169 | 0.369 | 1 | 686 |
| REACTOME_MET_INTERACTS_WITH_TNS_PROTEINS | 4 | 0.731 | 1.295 | 0.171 | 0.368 | 1 | 2143 |
| REACTOME_CHOLINE_CATABOLISM | 4 | 0.729 | 1.295 | 0.184 | 0.367 | 1 | 353 |
| REACTOME_VEGF_LIGAND_RECEPTOR_INTERACTIONS | 3 | 0.783 | 1.294 | 0.164 | 0.368 | 1 | 1621 |
| REACTOME_PLATELET_HOMEOSTASIS | 45 | 0.391 | 1.293 | 0.133 | 0.369 | 1 | 2618 |
| REACTOME_EGFR_TRANSACTIVATION_BY_GASTRIN | 7 | 0.618 | 1.291 | 0.181 | 0.372 | 1 | 102 |
| REACTOME_PD_1_SIGNALING | 6 | 0.636 | 1.291 | 0.194 | 0.371 | 1 | 4442 |
| REACTOME_NOTCH2_ACTIVATION_AND_TRANSMISSION_OF_SIGNAL_TO_THE_NUCLEUS | 20 | 0.457 | 1.289 | 0.157 | 0.373 | 1 | 1273 |
| REACTOME_RHO_GTPASES_ACTIVATE_PKNS | 39 | 0.393 | 1.288 | 0.130 | 0.375 | 1 | 2597 |
| REACTOME_VLDL_ASSEMBLY | 2 | 0.862 | 1.287 | 0.140 | 0.376 | 1 | 685 |
| REACTOME_DISEASES_OF_METABOLISM | 165 | 0.319 | 1.287 | 0.054 | 0.375 | 1 | 1541 |
| REACTOME_ASPARAGINE_N_LINKED_GLYCOSYLATION | 262 | 0.303 | 1.287 | 0.033 | 0.374 | 1 | 3899 |
| REACTOME_CONSTITUTIVE_SIGNALING_BY_AKT1_E17K_IN_CANCER | 26 | 0.434 | 1.284 | 0.155 | 0.378 | 1 | 714 |
| REACTOME_MAPK3_ERK1_ACTIVATION | 9 | 0.568 | 1.283 | 0.182 | 0.379 | 1 | 3168 |
| REACTOME_ABC_TRANSPORTERS_IN_LIPID_HOMEOSTASIS | 9 | 0.565 | 1.283 | 0.173 | 0.380 | 1 | 3568 |
| REACTOME_SHC1_EVENTS_IN_ERBB2_SIGNALING | 15 | 0.500 | 1.280 | 0.189 | 0.385 | 1 | 1170 |
| REACTOME_MET_ACTIVATES_PTK2_SIGNALING | 20 | 0.471 | 1.279 | 0.170 | 0.385 | 1 | 1146 |
| REACTOME_THE_ROLE_OF_NEF_IN_HIV_1_REPLICATION_AND_DISEASE_PATHOGENESIS | 22 | 0.462 | 1.279 | 0.160 | 0.385 | 1 | 2981 |
| REACTOME_INTERLEUKIN_RECEPTOR_SHC_SIGNALING | 15 | 0.493 | 1.278 | 0.155 | 0.385 | 1 | 3687 |
| REACTOME_SPRY_REGULATION_OF_FGF_SIGNALING | 16 | 0.495 | 1.277 | 0.163 | 0.386 | 1 | 3842 |

| | | | | | | | |
|---|---|---|---|---|---|---|---|
| REACTOME_SIGNALING_BY_ERYTHROPOIETIN | 21 | 0.462 | 1.274 | 0.152 | 0.391 | 1 | 3288 |
| REACTOME_PTK6_REGULATES_RTKS_AND_THEIR_EFFECTORS_AKT1_AND_DOK1 | 9 | 0.559 | 1.273 | 0.183 | 0.393 | 1 | 3842 |
| REACTOME_TRANSCRIPTIONAL_REGULATION_BY_RUNX1 | 162 | 0.314 | 1.273 | 0.065 | 0.392 | 1 | 1204 |
| REACTOME_INLB_MEDIATED_ENTRY_OF_LISTERIA_MONOCYTOGENES_INTO_HOST_CELL | 13 | 0.513 | 1.273 | 0.203 | 0.392 | 1 | 3855 |
| REACTOME_EPH_EPHRIN_MEDIATED_REPULSION_OF_CELLS | 42 | 0.395 | 1.272 | 0.126 | 0.392 | 1 | 2601 |
| REACTOME_FCERI_MEDIATED_CA_2_MOBILIZATION | 23 | 0.442 | 1.271 | 0.160 | 0.392 | 1 | 937 |
| REACTOME_CLASS_C_3_METABOTROPIC_GLUTAMATE_PHEROMONE_RECEPTORS_ | 3 | 0.769 | 1.271 | 0.161 | 0.393 | 1 | 266 |
| REACTOME_HCMV_EARLY_EVENTS | 72 | 0.360 | 1.270 | 0.108 | 0.393 | 1 | 2113 |
| REACTOME_THROMBIN_SIGNALLING_THROUGH_PROTEINASE_ACTIVATED_RECEPTORS_PARS_ | 19 | 0.477 | 1.270 | 0.170 | 0.393 | 1 | 1425 |
| REACTOME_MET_ACTIVATES_RAP1_AND_RAC1 | 9 | 0.568 | 1.269 | 0.185 | 0.393 | 1 | 3687 |
| REACTOME_IRE1ALPHA_ACTIVATES_CHAPERONES | 47 | 0.380 | 1.268 | 0.125 | 0.394 | 1 | 2724 |
| REACTOME_SUMO_IS_PROTEOLYTICALLY_PROCESSED | 6 | 0.626 | 1.268 | 0.204 | 0.394 | 1 | 4753 |
| REACTOME_FGFR1C_LIGAND_BINDING_AND_ACTIVATION | 2 | 0.843 | 1.266 | 0.150 | 0.397 | 1 | 1997 |
| REACTOME_CROSSLINKING_OF_COLLAGEN_FIBRILS | 9 | 0.553 | 1.265 | 0.175 | 0.398 | 1 | 611 |
| REACTOME_PROSTANOID_LIGAND_RECEPTORS | 1 | 0.949 | 1.263 | 0.102 | 0.400 | 1 | 652 |
| REACTOME_SYNTHESIS_OF_PE | 10 | 0.543 | 1.262 | 0.188 | 0.402 | 1 | 2886 |
| REACTOME_IMMUNOREGULATORY_INTERACTIONS_BETWEEN_A_LYMPHOID_AND_A_NON_LYMPHOID_CELL | 29 | 0.414 | 1.261 | 0.168 | 0.404 | 1 | 2991 |
| REACTOME_REGULATION_OF_SIGNALING_BY_CBL | 20 | 0.450 | 1.259 | 0.171 | 0.407 | 1 | 2159 |
| REACTOME_RUNX3_REGULATES_BCL2L11_BIM_TRANSCRIPTION | 5 | 0.658 | 1.257 | 0.197 | 0.410 | 1 | 1367 |
| REACTOME_DEFECTIVE_C1GALT1C1_CAUSES_TN_POLYAGGLUTINATION_SYNDROME_TNPS_ | 5 | 0.669 | 1.257 | 0.184 | 0.409 | 1 | 1156 |
| REACTOME_SURFACTANT_METABOLISM | 11 | 0.536 | 1.256 | 0.174 | 0.409 | 1 | 1592 |
| REACTOME_PHOSPHORYLATION_SITE_MUTANTS_OF_CTNNB1_ARE_NOT_TARGETED_TO_THE_PROTEASOME_BY_THE_DESTRUCTION_COMPLEX | 15 | 0.482 | 1.255 | 0.200 | 0.411 | 1 | 4060 |

| | | | | | | | |
|---|---|---|---|---|---|---|---|
| REACTOME_LYSOSOME_VESICLE_BIOGENESIS | 31 | 0.411 | 1.255 | 0.162 | 0.410 | 1 | 4650 |
| REACTOME_P75NTR_RECRUITS_SIGNALLING_COMPLEXES | 12 | 0.502 | 1.255 | 0.190 | 0.409 | 1 | 3842 |
| REACTOME_POST_TRANSLATIONAL_MODIFICATION_SYNTHESIS_OF_GPI_ANCHORED_PROTEINS | 40 | 0.391 | 1.254 | 0.146 | 0.409 | 1 | 872 |
| REACTOME_FRS_MEDIATED_FGFR2_SIGNALING | 11 | 0.527 | 1.254 | 0.213 | 0.408 | 1 | 1208 |
| REACTOME_LONG_TERM_POTENTIATION | 11 | 0.519 | 1.253 | 0.191 | 0.409 | 1 | 2027 |
| REACTOME_FRS_MEDIATED_FGFR3_SIGNALING | 11 | 0.536 | 1.252 | 0.215 | 0.411 | 1 | 1208 |
| REACTOME_DSCAM_INTERACTIONS | 7 | 0.600 | 1.251 | 0.211 | 0.412 | 1 | 3067 |
| REACTOME_REGULATION_OF_TLR_BY_ENDOGENOUS_LIGAND | 5 | 0.654 | 1.251 | 0.221 | 0.411 | 1 | 1424 |
| REACTOME_UNBLOCKING_OF_NMDA_RECEPTORS_GLUTAMATE_BINDING_AND_ACTIVATION | 10 | 0.540 | 1.251 | 0.207 | 0.411 | 1 | 2027 |
| REACTOME_SIGNAL_REGULATORY_PROTEIN_FAMILY_INTERACTIONS | 10 | 0.541 | 1.248 | 0.190 | 0.415 | 1 | 4442 |
| REACTOME_RAF_ACTIVATION | 32 | 0.412 | 1.248 | 0.158 | 0.416 | 1 | 4224 |
| REACTOME_CIRCADIAN_CLOCK | 67 | 0.351 | 1.247 | 0.129 | 0.415 | 1 | 3842 |
| REACTOME_PHENYLALANINE_AND_TYROSINE_METABOLISM | 7 | 0.595 | 1.247 | 0.233 | 0.416 | 1 | 3543 |
| REACTOME_NETRIN_MEDIATED_REPULSION_SIGNALS | 4 | 0.707 | 1.246 | 0.202 | 0.416 | 1 | 453 |
| REACTOME_INTERLEUKIN_23_SIGNALING | 5 | 0.661 | 1.246 | 0.230 | 0.415 | 1 | 31 |
| REACTOME_DISEASES_OF_PROGRAMMED_CELL_DEATH | 23 | 0.436 | 1.246 | 0.180 | 0.414 | 1 | 673 |
| REACTOME_INTERLEUKIN_3_INTERLEUKIN_5_AND_GM_CSF_SIGNALING | 34 | 0.394 | 1.246 | 0.158 | 0.414 | 1 | 4643 |
| REACTOME_FACTORS_INVOLVED_IN_MEGAKARYOCYTE_DEVELOPMENT_AND_PLATELET_PRODUCTION | 117 | 0.319 | 1.245 | 0.090 | 0.415 | 1 | 2113 |
| REACTOME_LYSOSPHINGOLIPID_AND_LPA_RECEPTORS | 6 | 0.621 | 1.245 | 0.220 | 0.414 | 1 | 1478 |
| REACTOME_SUMOYLATION_OF_IMMUNE_RESPONSE_PROTEINS | 11 | 0.528 | 1.243 | 0.198 | 0.417 | 1 | 5290 |
| REACTOME_MET_PROMOTES_CELL_MOTILITY | 30 | 0.407 | 1.243 | 0.180 | 0.416 | 1 | 3341 |
| REACTOME_CREATION_OF_C4_AND_C2_ACTIVATORS | 2 | 0.838 | 1.240 | 0.190 | 0.421 | 1 | 2064 |
| REACTOME_ESTROGEN_DEPENDENT_GENE_EXPRESSION | 93 | 0.333 | 1.238 | 0.121 | 0.423 | 1 | 3542 |
| REACTOME_CYCLIN_D_ASSOCIATED_EVENTS_IN_G1 | 46 | 0.376 | 1.236 | 0.140 | 0.428 | 1 | 2397 |

| | | | | | | | |
|---|---|---|---|---|---|---|---|
| REACTOME_SIGNALING_BY_MRAS_COMPLEX_MUTANTS | 8 | 0.567 | 1.235 | 0.213 | 0.429 | 1 | 3484 |
| REACTOME_STIMULI_SENSING_CHANNELS | 57 | 0.355 | 1.232 | 0.148 | 0.434 | 1 | 2203 |
| REACTOME_RNA_POLYMERASE_III_CHAIN_ELONGATION | 18 | 0.458 | 1.232 | 0.200 | 0.433 | 1 | 1742 |
| REACTOME_RUNX3_REGULATES_IMMUNE_RESPONSE_AND_CELL_MIGRATION | 2 | 0.809 | 1.231 | 0.189 | 0.433 | 1 | 1966 |
| REACTOME_ONCOGENE_INDUCED_SENESCENCE | 33 | 0.402 | 1.231 | 0.181 | 0.433 | 1 | 3170 |
| REACTOME_ENOS_ACTIVATION | 10 | 0.534 | 1.231 | 0.225 | 0.432 | 1 | 1167 |
| REACTOME_FGFR1B_LIGAND_BINDING_AND_ACTIVATION | 2 | 0.843 | 1.229 | 0.194 | 0.436 | 1 | 1997 |
| REACTOME_PROLONGED_ERK_ACTIVATION_EVENTS | 12 | 0.507 | 1.227 | 0.208 | 0.438 | 1 | 3513 |
| REACTOME_ACTIVATED_NTRK2_SIGNALS_THROUGH_CDK5 | 5 | 0.625 | 1.224 | 0.259 | 0.443 | 1 | 2678 |
| REACTOME_NEF_MEDIATED_CD4_DOWN_REGULATION | 9 | 0.548 | 1.223 | 0.235 | 0.446 | 1 | 2653 |
| REACTOME_ACTIVATION_OF_BH3_ONLY_PROTEINS | 29 | 0.405 | 1.222 | 0.194 | 0.446 | 1 | 3484 |
| REACTOME_ELECTRIC_TRANSMISSION_ACROSS_GAP_JUNCTIONS | 2 | 0.826 | 1.222 | 0.181 | 0.445 | 1 | 2209 |
| REACTOME_SIGNALING_BY_KIT_IN_DISEASE | 19 | 0.451 | 1.222 | 0.213 | 0.444 | 1 | 4612 |
| REACTOME_G_ALPHA_I_SIGNALLING_EVENTS | 145 | 0.307 | 1.222 | 0.103 | 0.444 | 1 | 3414 |
| REACTOME_TP53_REGULATES_TRANSCRIPTION_OF_CELL_CYCLE_GENES | 48 | 0.362 | 1.220 | 0.155 | 0.446 | 1 | 1074 |
| REACTOME_APOBEC3G_MEDIATED_RESISTANCE_TO_HIV_1_INFECTION | 5 | 0.638 | 1.220 | 0.222 | 0.445 | 1 | 3854 |
| REACTOME_RUNX1_REGULATES_TRANSCRIPTION_OF_GENES_INVOLVED_IN_WNT_SIGNALING | 4 | 0.678 | 1.220 | 0.234 | 0.445 | 1 | 3426 |
| REACTOME_NEF_MEDIATES_DOWN_MODULATION_OF_CELL_SURFACE_RECEPTORS_BY_RECRUITING_THEM_TO_CLATHRIN_ADAPTERS | 19 | 0.450 | 1.219 | 0.213 | 0.447 | 1 | 2755 |
| REACTOME_TRAF6_MEDIATED_INDUCTION_OF_TAK1_COMPLEX_WITHIN_TLR4_COMPLEX | 14 | 0.487 | 1.218 | 0.228 | 0.447 | 1 | 4190 |
| REACTOME_INTERLEUKIN_27_SIGNALING | 8 | 0.564 | 1.218 | 0.245 | 0.446 | 1 | 3168 |
| REACTOME_VEGFR2_MEDIATED_VASCULAR_PERMEABILITY | 23 | 0.425 | 1.216 | 0.208 | 0.450 | 1 | 3080 |
| REACTOME_SOS_MEDIATED_SIGNALLING | 7 | 0.591 | 1.215 | 0.243 | 0.450 | 1 | 4118 |

| | | | | | | | |
|---|---|---|---|---|---|---|---|
| REACTOME_NRAGE_SIGNALS_DEATH_THROUGH_JNK | 52 | 0.359 | 1.215 | 0.162 | 0.449 | 1 | 615 |
| REACTOME_SELECTIVE_AUTOPHAGY | 69 | 0.338 | 1.214 | 0.169 | 0.451 | 1 | 2317 |
| REACTOME_TRANSPORT_OF_NUCLEOTIDE_SUGARS | 9 | 0.531 | 1.214 | 0.227 | 0.451 | 1 | 1636 |
| REACTOME_G_ALPHA_12_13_SIGNALLING_EVENTS | 60 | 0.348 | 1.213 | 0.163 | 0.450 | 1 | 1916 |
| REACTOME_RECEPTOR_MEDIATED_MITOPHAGY | 11 | 0.514 | 1.213 | 0.215 | 0.449 | 1 | 4201 |
| REACTOME_UNFOLDED_PROTEIN_RESPONSE_UPR_ | 87 | 0.322 | 1.210 | 0.143 | 0.455 | 1 | 1783 |
| REACTOME_ESTROGEN_STIMULATED_SIGNALING_THROUGH_PRKCZ | 6 | 0.613 | 1.209 | 0.247 | 0.455 | 1 | 4927 |
| REACTOME_REGULATED_PROTEOLYSIS_OF_P75NTR | 11 | 0.505 | 1.209 | 0.248 | 0.456 | 1 | 4 |
| REACTOME_ROBO_RECEPTORS_BIND_AKAP5 | 7 | 0.579 | 1.208 | 0.237 | 0.456 | 1 | 2206 |
| REACTOME_OXIDATIVE_STRESS_INDUCED_SENESCENCE | 72 | 0.334 | 1.208 | 0.165 | 0.455 | 1 | 3877 |
| REACTOME_RNA_POLYMERASE_III_TRANSCRIPTION_INITIATION_FROM_TYPE_3_PROMOTER | 28 | 0.401 | 1.208 | 0.196 | 0.455 | 1 | 2381 |
| REACTOME_INTERLEUKIN_35_SIGNALLING | 8 | 0.564 | 1.207 | 0.260 | 0.456 | 1 | 3168 |
| REACTOME_ERYTHROPOIETIN_ACTIVATES_STAT5 | 6 | 0.615 | 1.207 | 0.246 | 0.455 | 1 | 255 |
| REACTOME_CALCINEURIN_ACTIVATES_NFAT | 9 | 0.548 | 1.207 | 0.261 | 0.454 | 1 | 4224 |
| REACTOME_INACTIVATION_OF_CDC42_AND_RAC1 | 6 | 0.606 | 1.206 | 0.264 | 0.454 | 1 | 2870 |
| REACTOME_HIGHLY_CALCIUM_PERMEABLE_NICOTINIC_ACETYLCHOLINE_RECEPTORS | 4 | 0.665 | 1.204 | 0.252 | 0.457 | 1 | 236 |
| REACTOME_DARPP_32_EVENTS | 19 | 0.440 | 1.201 | 0.213 | 0.462 | 1 | 3697 |
| REACTOME_THE_ROLE_OF_GTSE1_IN_G2_M_PROGRESSION_AFTER_G2_CHECKPOINT | 69 | 0.334 | 1.201 | 0.172 | 0.462 | 1 | 2159 |
| REACTOME_NR1H2_AND_NR1H3_MEDIATED_SIGNALING | 37 | 0.380 | 1.199 | 0.204 | 0.465 | 1 | 1765 |
| REACTOME_GOLGI_CISTERNAE_PERICENTRIOLAR_STACK_REORGANIZATION | 14 | 0.489 | 1.198 | 0.221 | 0.467 | 1 | 3062 |
| REACTOME_RAS_SIGNALING_DOWNSTREAM_OF_NF1_LOSS_OF_FUNCTION_VARIANTS | 7 | 0.589 | 1.198 | 0.269 | 0.466 | 1 | 4927 |
| REACTOME_REMOVAL_OF_AMINOTERMINAL_PROPEPTIDES_FROM_GAMMA_CARBOXYLATED_PROTEINS | 7 | 0.561 | 1.197 | 0.264 | 0.467 | 1 | 1175 |
| REACTOME_CELLULAR_SENESCENCE | 134 | 0.299 | 1.196 | 0.138 | 0.467 | 1 | 3641 |

| | | | | | | | |
|---|---|---|---|---|---|---|---|
| REACTOME_TP53_REGULATES_TRANSCRIPTION_OF_CELL_DEATH_GENES | 37 | 0.379 | 1.196 | 0.194 | 0.466 | 1 | 2084 |
| REACTOME_DOWNREGULATION_OF_ERBB2_ERBB3_SIGNALING | 11 | 0.499 | 1.192 | 0.241 | 0.474 | 1 | 3842 |
| REACTOME_CALNEXIN_CALRETICULIN_CYCLE | 26 | 0.400 | 1.192 | 0.239 | 0.474 | 1 | 4248 |
| REACTOME_NOSTRIN_MEDIATED_ENOS_TRAFFICKING | 3 | 0.718 | 1.192 | 0.281 | 0.473 | 1 | 240 |
| REACTOME_SLC_MEDIATED_TRANSMEMBRANE_TRANSPORT | 132 | 0.305 | 1.190 | 0.144 | 0.475 | 1 | 1793 |
| REACTOME_GAMMA_CARBOXYLATION_TRANSPORT_AND_AMINO_TERMINAL_CLEAVAGE_OF_PROTEINS | 8 | 0.560 | 1.188 | 0.271 | 0.480 | 1 | 1175 |
| REACTOME_HIGHLY_SODIUM_PERMEABLE_POSTSYNAPTIC_ACETYLCHOLINE_NICOTINIC_RECEPTORS | 2 | 0.796 | 1.186 | 0.259 | 0.482 | 1 | 189 |
| REACTOME_INTEGRATION_OF_PROVIRUS | 9 | 0.529 | 1.185 | 0.271 | 0.484 | 1 | 3854 |
| REACTOME_HIGHLY_CALCIUM_PERMEABLE_POSTSYNAPTIC_NICOTINIC_ACETYLCHOLINE_RECEPTORS | 4 | 0.665 | 1.184 | 0.280 | 0.485 | 1 | 236 |
| REACTOME_CD28_CO_STIMULATION | 28 | 0.403 | 1.180 | 0.232 | 0.493 | 1 | 3080 |
| REACTOME_INTERLEUKIN_9_SIGNALING | 5 | 0.633 | 1.179 | 0.284 | 0.494 | 1 | 2771 |
| REACTOME_RHO_GTPASES_ACTIVATE_NADPH_OXIDASES | 14 | 0.469 | 1.179 | 0.250 | 0.493 | 1 | 2887 |
| REACTOME_P75NTR_SIGNALS_VIA_NF_KB | 15 | 0.458 | 1.178 | 0.254 | 0.495 | 1 | 3842 |
| REACTOME_CELLULAR_HEXOSE_TRANSPORT | 12 | 0.486 | 1.176 | 0.273 | 0.499 | 1 | 1476 |
| REACTOME_TP53_REGULATES_TRANSCRIPTION_OF_SEVERAL_ADDITIONAL_CELL_DEATH_GENES_WHOSE_SPECIFIC_ROLES_IN_P53_DEPENDENT_APOPTOSIS_REMAIN_UNCERTAIN | 12 | 0.484 | 1.174 | 0.251 | 0.500 | 1 | 2084 |
| REACTOME_ACROSOME_REACTION_AND_SPERM_OOCYTE_MEMBRANE_BINDING | 1 | 0.877 | 1.174 | 0.234 | 0.500 | 1 | 1562 |
| REACTOME_NF_KB_IS_ACTIVATED_AND_SIGNALS_SURVIVAL | 12 | 0.483 | 1.172 | 0.277 | 0.504 | 1 | 4 |
| REACTOME_GLUCURONIDATION | 4 | 0.656 | 1.171 | 0.303 | 0.505 | 1 | 1222 |
| REACTOME_ER_QUALITY_CONTROL_COMPARTMENT_ERQC_ | 21 | 0.423 | 1.171 | 0.274 | 0.504 | 1 | 4248 |
| REACTOME_UREA_CYCLE | 5 | 0.620 | 1.170 | 0.311 | 0.504 | 1 | 824 |
| REACTOME_O_LINKED_GLYCOSYLATION | 61 | 0.332 | 1.170 | 0.213 | 0.504 | 1 | 2874 |

| | | | | | | | |
|---|---|---|---|---|---|---|---|
| REACTOME_PYRIMIDINE_SALVAGE | 8 | 0.528 | 1.169 | 0.288 | 0.504 | 1 | 1122 |
| REACTOME_PTK6_REGULATES_CELL_CYCLE | 6 | 0.579 | 1.168 | 0.295 | 0.507 | 1 | 1052 |
| REACTOME_GLYCOGEN_SYNTHESIS | 13 | 0.481 | 1.167 | 0.282 | 0.507 | 1 | 3842 |
| REACTOME_DISEASES_OF_MITOTIC_CELL_CYCLE | 35 | 0.373 | 1.166 | 0.250 | 0.508 | 1 | 1433 |
| REACTOME_FGFR3_LIGAND_BINDING_AND_ACTIVATION | 4 | 0.658 | 1.163 | 0.309 | 0.513 | 1 | 1208 |
| REACTOME_SMOOTH_MUSCLE_CONTRACTION | 25 | 0.403 | 1.163 | 0.238 | 0.513 | 1 | 3067 |
| REACTOME_TRANSCRIPTIONAL_REGULATION_BY_RUNX3 | 92 | 0.308 | 1.163 | 0.178 | 0.512 | 1 | 2466 |
| REACTOME_NEGATIVE_REGULATION_OF_NMDA_RECEPTOR_MEDIATED_NEURONAL_TRANSMISSION | 12 | 0.476 | 1.161 | 0.276 | 0.514 | 1 | 2027 |
| REACTOME_PLASMA_LIPOPROTEIN_CLEARANCE | 27 | 0.397 | 1.161 | 0.246 | 0.515 | 1 | 1372 |
| REACTOME_TRANSPORT_OF_VITAMINS_NUCLEOSIDES_AND_RELATED_MOLECULES | 28 | 0.394 | 1.159 | 0.254 | 0.517 | 1 | 1636 |
| REACTOME_FRS_MEDIATED_FGFR1_SIGNALING | 11 | 0.497 | 1.157 | 0.290 | 0.520 | 1 | 4442 |
| REACTOME_SIGNALING_BY_FGFR2_IN_DISEASE | 28 | 0.396 | 1.157 | 0.251 | 0.519 | 1 | 2804 |
| REACTOME_DOWNSTREAM_SIGNAL_TRANSDUCTION | 28 | 0.390 | 1.157 | 0.258 | 0.519 | 1 | 2804 |
| REACTOME_CS_DS_DEGRADATION | 9 | 0.517 | 1.157 | 0.297 | 0.518 | 1 | 1541 |
| REACTOME_BMAL1_CLOCK_NPAS2_ACTIVATES_CIRCADIAN_GENE_EXPRESSION | 26 | 0.398 | 1.156 | 0.255 | 0.518 | 1 | 1695 |
| REACTOME_RUNX1_REGULATES_EXPRESSION_OF_COMPONENTS_OF_TIGHT_JUNCTIONS | 4 | 0.649 | 1.156 | 0.323 | 0.518 | 1 | 4464 |
| REACTOME_NUCLEOTIDE_BINDING_DOMAIN_LEUCINE_RICH_REPEAT_CONTAINING_RECEPTOR_NLR_SIGNALING_PATHWAYS | 47 | 0.349 | 1.156 | 0.234 | 0.517 | 1 | 2742 |
| REACTOME_METABOLISM_OF_NITRIC_OXIDE_NOS3_ACTIVATION_AND_REGULATION | 13 | 0.472 | 1.155 | 0.293 | 0.519 | 1 | 1167 |
| REACTOME_CYP2E1_REACTIONS | 3 | 0.706 | 1.151 | 0.321 | 0.526 | 1 | 243 |
| REACTOME_SEMA4D_MEDIATED_INHIBITION_OF_CELL_ATTACHMENT_AND_MIGRATION | 8 | 0.525 | 1.151 | 0.293 | 0.525 | 1 | 2958 |
| REACTOME_NRIF_SIGNALS_CELL_DEATH_FROM_THE_NUCLEUS | 15 | 0.453 | 1.151 | 0.286 | 0.525 | 1 | 4 |
| REACTOME_INTRA_GOLGI_AND_RETROGRADE_GOLGI_TO_ER_TRAFFIC | 183 | 0.278 | 1.148 | 0.173 | 0.530 | 1 | 2170 |

| | | | | | | | |
|---|---|---|---|---|---|---|---|
| REACTOME_PI_3K_CASCADE_FGFR1 | 8 | 0.528 | 1.147 | 0.305 | 0.532 | 1 | 1997 |
| REACTOME_ERYTHROPOIETIN_ACTIVATES_PHOSPHOINOSITIDE_3_KINASE_PI3K_ | 8 | 0.531 | 1.146 | 0.301 | 0.532 | 1 | 1470 |
| REACTOME_RAP1_SIGNALLING | 13 | 0.459 | 1.145 | 0.306 | 0.533 | 1 | 3484 |
| REACTOME_INTRINSIC_PATHWAY_FOR_APOPTOSIS | 51 | 0.338 | 1.143 | 0.246 | 0.536 | 1 | 3264 |
| REACTOME_SIGNALING_BY_BRAF_AND_RAF_FUSIONS | 57 | 0.329 | 1.143 | 0.231 | 0.536 | 1 | 3589 |
| REACTOME_ACTIVATION_OF_IRF3_IRF7_MEDIATED_BY_TBK1_IKK_EPSILON | 15 | 0.447 | 1.143 | 0.303 | 0.535 | 1 | 2159 |
| REACTOME_AUTOPHAGY | 134 | 0.286 | 1.140 | 0.195 | 0.539 | 1 | 3259 |
| REACTOME_REGULATION_OF_FOXO_TRANSCRIPTIONAL_ACTIVITY_BY_ACETYLATION | 10 | 0.499 | 1.139 | 0.321 | 0.541 | 1 | 1204 |
| REACTOME_PREVENTION_OF_PHAGOSOMAL_LYSOSOMAL_FUSION | 9 | 0.508 | 1.136 | 0.330 | 0.548 | 1 | 2538 |
| REACTOME_SHC1_EVENTS_IN_ERBB4_SIGNALING | 9 | 0.522 | 1.136 | 0.312 | 0.547 | 1 | 770 |
| REACTOME_CELLULAR_RESPONSES_TO_EXTERNAL_STIMULI | 499 | 0.252 | 1.132 | 0.133 | 0.553 | 1 | 3038 |
| REACTOME_MAPK1_ERK2_ACTIVATION | 8 | 0.517 | 1.131 | 0.310 | 0.556 | 1 | 3513 |
| REACTOME_RHO_GTPASES_ACTIVATE_FORMINS | 129 | 0.285 | 1.129 | 0.206 | 0.559 | 1 | 2870 |
| REACTOME_SIGNALLING_TO_P38_VIA_RIT_AND_RIN | 2 | 0.739 | 1.129 | 0.338 | 0.558 | 1 | 486 |
| REACTOME_RUNX1_INTERACTS_WITH_CO_FACTORS_WHOSE_PRECISE_EFFECT_ON_RUNX1_TARGETS_IS_NOT_KNOWN | 35 | 0.361 | 1.129 | 0.269 | 0.558 | 1 | 4020 |
| REACTOME_PI3K_AKT_ACTIVATION | 7 | 0.544 | 1.128 | 0.340 | 0.557 | 1 | 255 |
| REACTOME_NEGATIVE_FEEDBACK_REGULATION_OF_MAPK_PATHWAY | 6 | 0.558 | 1.126 | 0.354 | 0.561 | 1 | 3513 |
| REACTOME_ALPHA_PROTEIN_KINASE_1_SIGNALING_PATHWAY | 11 | 0.474 | 1.125 | 0.336 | 0.563 | 1 | 3849 |
| REACTOME_ROLE_OF_LAT2_NTAL_LAB_ON_CALCIUM_MOBILIZATION | 13 | 0.465 | 1.125 | 0.337 | 0.563 | 1 | 1652 |
| REACTOME_NEGATIVE_REGULATION_OF_ACTIVITY_OF_TFAP2_AP_2_FAMILY_TRANSCRIPTION_FACTORS | 7 | 0.528 | 1.124 | 0.353 | 0.562 | 1 | 78 |
| REACTOME_INSULIN_PROCESSING | 22 | 0.392 | 1.123 | 0.305 | 0.564 | 1 | 3527 |

| | | | | | | | |
|---|---|---|---|---|---|---|---|
| REACTOME_SENESCENCE_ASSOCIATED_SECRETORY_PHENOTYPE_SASP_ | 57 | 0.330 | 1.120 | 0.277 | 0.570 | 1 | 3842 |
| REACTOME_GLYCOGEN_BREAKDOWN_GLYCOGENOLYSIS_ | 15 | 0.438 | 1.116 | 0.319 | 0.578 | 1 | 377 |
| REACTOME_G_ALPHA_Q_SIGNALLING_EVENTS | 87 | 0.303 | 1.116 | 0.264 | 0.578 | 1 | 1263 |
| REACTOME_ANTI_INFLAMMATORY_RESPONSE_FAVOURING_LEISHMANIA_PARASITE_INFECTION | 58 | 0.320 | 1.114 | 0.277 | 0.580 | 1 | 3192 |
| REACTOME_ROLE_OF_ABL_IN_ROBO_SLIT_SIGNALING | 7 | 0.527 | 1.113 | 0.368 | 0.583 | 1 | 2324 |
| REACTOME_TYROSINE_CATABOLISM | 3 | 0.695 | 1.112 | 0.376 | 0.583 | 1 | 3543 |
| REACTOME_TOXICITY_OF_BOTULINUM_TOXIN_TYPE_D_BONT_D_ | 3 | 0.672 | 1.109 | 0.377 | 0.589 | 1 | 4173 |
| REACTOME_RHO_GTPASES_ACTIVATE_KTN1 | 11 | 0.468 | 1.106 | 0.336 | 0.594 | 1 | 3357 |
| REACTOME_THE_NLRP3_INFLAMMASOME | 10 | 0.477 | 1.105 | 0.339 | 0.596 | 1 | 2185 |
| REACTOME_SIGNALING_BY_NTRK2_TRKB_ | 21 | 0.394 | 1.104 | 0.337 | 0.596 | 1 | 770 |
| REACTOME_SLC_TRANSPORTER_DISORDERS | 54 | 0.320 | 1.104 | 0.291 | 0.597 | 1 | 2107 |
| REACTOME_HCMV_INFECTION | 96 | 0.289 | 1.102 | 0.286 | 0.599 | 1 | 2152 |
| REACTOME_THE_PHOTOTRANSDUCTION_CASCADE | 15 | 0.426 | 1.101 | 0.327 | 0.601 | 1 | 1257 |
| REACTOME_GENE_AND_PROTEIN_EXPRESSION_BY_JAK_STAT_SIGNALING_AFTER_INTERLEUKIN_12_STIMULATION | 31 | 0.358 | 1.100 | 0.327 | 0.601 | 1 | 4510 |
| REACTOME_ATF4_ACTIVATES_GENES_IN_RESPONSE_TO_ENDOPLASMIC_RETICULUM_STRESS | 26 | 0.378 | 1.099 | 0.330 | 0.602 | 1 | 792 |
| REACTOME_MAP3K8_TPL2_DEPENDENT_MAPK1_3_ACTIVATION | 16 | 0.422 | 1.099 | 0.333 | 0.601 | 1 | 4551 |
| REACTOME_REGULATION_OF_RUNX1_EXPRESSION_AND_ACTIVITY | 16 | 0.425 | 1.098 | 0.330 | 0.602 | 1 | 2397 |
| REACTOME_CRMPS_IN_SEMA3A_SIGNALING | 12 | 0.451 | 1.093 | 0.368 | 0.615 | 1 | 1228 |
| REACTOME_REDUCTION_OF_CYTOSOLIC_CA_LEVELS | 7 | 0.517 | 1.091 | 0.370 | 0.617 | 1 | 604 |
| REACTOME_FC_EPSILON_RECEPTOR_FCERI_SIGNALING | 117 | 0.280 | 1.091 | 0.320 | 0.617 | 1 | 3067 |
| REACTOME_CELL_DEATH_SIGNALLING_VIA_NRAGE_NRIF_AND_NADE | 69 | 0.305 | 1.090 | 0.302 | 0.617 | 1 | 615 |
| REACTOME_ERBB2_ACTIVATES_PTK6_SIGNALING | 6 | 0.550 | 1.090 | 0.385 | 0.616 | 1 | 695 |
| REACTOME_MATURATION_OF_SPIKE_PROTEIN | 5 | 0.581 | 1.089 | 0.388 | 0.617 | 1 | 4107 |

| | | | | | | | |
|---|---|---|---|---|---|---|---|
| REACTOME_PI3K_EVENTS_IN_ERBB2_SIGNALING | 8 | 0.495 | 1.086 | 0.360 | 0.625 | 1 | 695 |
| REACTOME_KERATAN_SULFATE_KERATIN_METABOLISM | 23 | 0.377 | 1.086 | 0.351 | 0.624 | 1 | 2876 |
| REACTOME_REGULATION_OF_FZD_BY_UBIQUITINATION | 15 | 0.427 | 1.085 | 0.368 | 0.623 | 1 | 2159 |
| REACTOME_REGULATION_OF_PTEN_GENE_TRANSCRIPTION | 60 | 0.313 | 1.084 | 0.320 | 0.625 | 1 | 2605 |
| REACTOME_INTERACTIONS_OF_VPR_WITH_HOST_CELLULAR_PROTEINS | 37 | 0.340 | 1.083 | 0.324 | 0.627 | 1 | 4177 |
| REACTOME_ERBB2_REGULATES_CELL_MOTILITY | 8 | 0.511 | 1.082 | 0.392 | 0.627 | 1 | 695 |
| REACTOME_SIGNALING_BY_NOTCH2 | 28 | 0.365 | 1.081 | 0.360 | 0.628 | 1 | 472 |
| REACTOME_SEMA3A_PLEXIN_REPULSION_SIGNALING_BY_INHIBITING_INTEGRIN_ADHESION | 12 | 0.444 | 1.081 | 0.364 | 0.628 | 1 | 2958 |
| REACTOME_REACTIONS_SPECIFIC_TO_THE_COMPLEX_N_GLYCAN_SYNTHESIS_PATHWAY | 5 | 0.567 | 1.079 | 0.418 | 0.632 | 1 | 2489 |
| REACTOME_VXPX_CARGO_TARGETING_TO_CILIUM | 17 | 0.407 | 1.078 | 0.374 | 0.633 | 1 | 3269 |
| REACTOME_NETRIN_1_SIGNALING | 36 | 0.343 | 1.078 | 0.336 | 0.632 | 1 | 3067 |
| REACTOME_GLYCOLYSIS | 62 | 0.308 | 1.076 | 0.314 | 0.635 | 1 | 2240 |
| REACTOME_GLYCOGEN_METABOLISM | 24 | 0.379 | 1.076 | 0.375 | 0.634 | 1 | 1171 |
| REACTOME_TCR_SIGNALING | 94 | 0.283 | 1.075 | 0.320 | 0.634 | 1 | 3067 |
| REACTOME_REGULATION_OF_TP53_ACTIVITY_THROUGH_ACETYLATION | 30 | 0.354 | 1.075 | 0.336 | 0.633 | 1 | 5067 |
| REACTOME_SIGNALING_BY_WNT | 221 | 0.255 | 1.075 | 0.282 | 0.634 | 1 | 1710 |
| REACTOME_2_LTR_CIRCLE_FORMATION | 7 | 0.515 | 1.073 | 0.399 | 0.635 | 1 | 3854 |
| REACTOME_DOWNSTREAM_SIGNALING_OF_ACTIVATED_FGFR1 | 15 | 0.414 | 1.073 | 0.362 | 0.634 | 1 | 2804 |
| REACTOME_PTK6_EXPRESSION | 5 | 0.576 | 1.072 | 0.417 | 0.636 | 1 | 1852 |
| REACTOME_HORMONE_LIGAND_BINDING_RECEPTORS | 1 | 0.809 | 1.070 | 0.398 | 0.640 | 1 | 2429 |
| REACTOME_INTRAFLAGELLAR_TRANSPORT | 48 | 0.318 | 1.069 | 0.367 | 0.641 | 1 | 910 |
| REACTOME_RNF_MUTANTS_SHOW_ENHANCED_WNT_SIGNALING_AND_PROLIFERATION | 6 | 0.532 | 1.068 | 0.407 | 0.642 | 1 | 990 |
| REACTOME_MET_ACTIVATES_PTPN11 | 3 | 0.651 | 1.068 | 0.437 | 0.642 | 1 | 4442 |
| REACTOME_PLATELET_CALCIUM_HOMEOSTASIS | 17 | 0.393 | 1.067 | 0.365 | 0.642 | 1 | 2373 |
| REACTOME_INTERLEUKIN_12_SIGNALING | 36 | 0.338 | 1.067 | 0.355 | 0.641 | 1 | 4017 |

| | | | | | | | |
|---|---|---|---|---|---|---|---|
| REACTOME_INTERLEUKIN_37_SIGNALING | 14 | 0.420 | 1.067 | 0.388 | 0.641 | 1 | 3283 |
| REACTOME_MISCELLANEOUS_TRANSPORT_AND_BINDING_EVENTS | 21 | 0.374 | 1.067 | 0.367 | 0.641 | 1 | 2664 |
| REACTOME_SIGNALING_BY_HIPPO | 19 | 0.395 | 1.064 | 0.369 | 0.646 | 1 | 2944 |
| REACTOME_ARYL_HYDROCARBON_RECEPTOR_SIGNALLING | 6 | 0.526 | 1.064 | 0.387 | 0.645 | 1 | 2050 |
| REACTOME_SIGNALING_BY_ERBB2_IN_CANCER | 18 | 0.404 | 1.060 | 0.384 | 0.653 | 1 | 1170 |
| REACTOME_SYNTHESIS_OF_IP2_IP_AND_INS_IN_THE_CYTOSOL | 11 | 0.456 | 1.060 | 0.373 | 0.652 | 1 | 2901 |
| REACTOME_MUCOPOLYSACCHARIDOSES | 11 | 0.442 | 1.059 | 0.378 | 0.652 | 1 | 1498 |
| REACTOME_PI_METABOLISM | 69 | 0.297 | 1.059 | 0.378 | 0.652 | 1 | 2725 |
| REACTOME_ACYL_CHAIN_REMODELLING_OF_PG | 6 | 0.527 | 1.056 | 0.424 | 0.658 | 1 | 2191 |
| REACTOME_RHO_GTPASES_ACTIVATE_RHOTEKIN_AND_RHOPHILINS | 8 | 0.473 | 1.054 | 0.403 | 0.660 | 1 | 4089 |
| REACTOME_PHASE_2_PLATEAU_PHASE | 9 | 0.470 | 1.054 | 0.427 | 0.661 | 1 | 2906 |
| REACTOME_TRANSCRIPTIONAL_REGULATION_BY_RUNX2 | 101 | 0.278 | 1.053 | 0.336 | 0.660 | 1 | 3249 |
| REACTOME_NEPHRIN_FAMILY_INTERACTIONS | 16 | 0.401 | 1.051 | 0.403 | 0.665 | 1 | 3589 |
| REACTOME_AMINO_ACIDS_REGULATE_MTORC1 | 50 | 0.311 | 1.051 | 0.367 | 0.665 | 1 | 3038 |
| REACTOME_AMINO_ACID_TRANSPORT_ACROSS_THE_PLASMA_MEMBRANE | 21 | 0.375 | 1.050 | 0.400 | 0.665 | 1 | 3233 |
| REACTOME_RAB_REGULATION_OF_TRAFFICKING | 111 | 0.270 | 1.048 | 0.353 | 0.667 | 1 | 3679 |
| REACTOME_INTERFERON_SIGNALING | 142 | 0.265 | 1.048 | 0.347 | 0.668 | 1 | 3688 |
| REACTOME_LTC4_CYSLTR_MEDIATED_IL4_PRODUCTION | 2 | 0.699 | 1.047 | 0.461 | 0.668 | 1 | 3827 |
| REACTOME_ABORTIVE_ELONGATION_OF_HIV_1_TRANSCRIPT_IN_THE_ABSENCE_OF_TAT | 23 | 0.370 | 1.046 | 0.398 | 0.671 | 1 | 3778 |
| REACTOME_PRE_NOTCH_EXPRESSION_AND_PROCESSING | 57 | 0.306 | 1.044 | 0.400 | 0.674 | 1 | 2126 |
| REACTOME_PERK_REGULATES_GENE_EXPRESSION | 31 | 0.340 | 1.042 | 0.394 | 0.676 | 1 | 792 |
| REACTOME_SRP_DEPENDENT_COTRANSLATIONAL_PROTEIN_TARGETING_TO_MEMBRANE | 108 | 0.267 | 1.040 | 0.379 | 0.681 | 1 | 5937 |
| REACTOME_FGFR2B_LIGAND_BINDING_AND_ACTIVATION | 3 | 0.635 | 1.039 | 0.495 | 0.682 | 1 | 3003 |
| REACTOME_IRAK2_MEDIATED_ACTIVATION_OF_TAK1_COMPLEX | 10 | 0.455 | 1.038 | 0.409 | 0.684 | 1 | 4190 |

| | | | | | | | |
|---|---|---|---|---|---|---|---|
| REACTOME_INTERLEUKIN_15_SIGNALING | 10 | 0.446 | 1.036 | 0.406 | 0.686 | 1 | 3687 |
| REACTOME_PEPTIDE_HORMONE_BIOSYNTHESIS | 3 | 0.644 | 1.035 | 0.481 | 0.688 | 1 | 2127 |
| REACTOME_ATTACHMENT_OF_GPI_ANCHOR_TO_UPAR | 7 | 0.499 | 1.035 | 0.438 | 0.687 | 1 | 3455 |
| REACTOME_PROTON_COUPLED_MONOCARBOXYLATE_TRANSPORT | 3 | 0.624 | 1.034 | 0.470 | 0.689 | 1 | 4784 |
| REACTOME_FCGR_ACTIVATION | 5 | 0.553 | 1.033 | 0.470 | 0.689 | 1 | 484 |
| REACTOME_ACTIVATION_OF_KAINATE_RECEPTORS_UPON_GLUTAMATE_BINDING | 15 | 0.406 | 1.032 | 0.407 | 0.690 | 1 | 2618 |
| REACTOME_TGF_BETA_RECEPTOR_SIGNALING_IN_EMT_EPITHELIAL_TO_MESENCHYMAL_TRANSITION_ | 16 | 0.402 | 1.031 | 0.432 | 0.691 | 1 | 4089 |
| REACTOME_FGFR1_MUTANT_RECEPTOR_ACTIVATION | 22 | 0.371 | 1.031 | 0.425 | 0.690 | 1 | 5475 |
| REACTOME_TRP_CHANNELS | 13 | 0.419 | 1.029 | 0.435 | 0.693 | 1 | 1969 |
| REACTOME_ADORA2B_MEDIATED_ANTI_INFLAMMATORY_CYTOKINES_PRODUCTION | 37 | 0.320 | 1.025 | 0.430 | 0.703 | 1 | 3050 |
| REACTOME_ZINC_EFFLUX_AND_COMPARTMENTALIZATION_BY_THE_SLC30_FAMILY | 6 | 0.516 | 1.024 | 0.443 | 0.702 | 1 | 3880 |
| REACTOME_SIGNALING_BY_NOTCH1_HD_DOMAIN_MUTANTS_IN_CANCER | 14 | 0.414 | 1.021 | 0.427 | 0.708 | 1 | 2159 |
| REACTOME_INOSITOL_PHOSPHATE_METABOLISM | 36 | 0.318 | 1.021 | 0.419 | 0.709 | 1 | 3487 |
| REACTOME_ACTIVATION_OF_RAS_IN_B_CELLS | 3 | 0.612 | 1.019 | 0.482 | 0.711 | 1 | 4927 |
| REACTOME_PREGNENOLONE_BIOSYNTHESIS | 9 | 0.464 | 1.019 | 0.462 | 0.711 | 1 | 530 |
| REACTOME_RUNX1_REGULATES_TRANSCRIPTION_OF_GENES_INVOLVED_IN_BCR_SIGNALING | 4 | 0.574 | 1.018 | 0.479 | 0.711 | 1 | 3249 |
| REACTOME_SIGNALING_BY_NOTCH1_PEST_DOMAIN_MUTANTS_IN_CANCER | 56 | 0.293 | 1.018 | 0.424 | 0.711 | 1 | 2159 |
| REACTOME_PHENYLALANINE_METABOLISM | 4 | 0.573 | 1.017 | 0.485 | 0.711 | 1 | 2045 |
| REACTOME_PI3K_EVENTS_IN_ERBB4_SIGNALING | 5 | 0.539 | 1.017 | 0.492 | 0.711 | 1 | 695 |
| REACTOME_SODIUM_COUPLED_PHOSPHATE_COTRANSPORTERS | 2 | 0.692 | 1.016 | 0.489 | 0.711 | 1 | 1525 |
| REACTOME_WAX_AND_PLASMALOGEN_BIOSYNTHESIS | 4 | 0.561 | 1.016 | 0.492 | 0.711 | 1 | 753 |
| REACTOME_SODIUM_PROTON_EXCHANGERS | 5 | 0.526 | 1.010 | 0.492 | 0.723 | 1 | 4045 |

| | | | | | | | |
|---|---|---|---|---|---|---|---|
| REACTOME_SIGNALING_BY_FGFR2_IIIA_TM | 16 | 0.386 | 1.009 | 0.445 | 0.725 | 1 | 2715 |
| REACTOME_P75_NTR_RECEPTOR_MEDIATED_SIGNALLING | 87 | 0.273 | 1.008 | 0.420 | 0.725 | 1 | 3450 |
| REACTOME_OPSINS | 1 | 0.757 | 1.004 | 0.525 | 0.734 | 1 | 3093 |
| REACTOME_COMPETING_ENDOGENOUS_RNAS_CERNAS_REGULATE_PTEN_TRANSLATION | 8 | 0.455 | 1.004 | 0.459 | 0.733 | 1 | 1061 |
| REACTOME_GLUCAGON_LIKE_PEPTIDE_1_GLP1_REGULATES_INSULIN_SECRETION | 22 | 0.360 | 1.004 | 0.446 | 0.732 | 1 | 2618 |
| REACTOME_CYTOSOLIC_SULFONATION_OF_SMALL_MOLECULES | 15 | 0.388 | 1.004 | 0.437 | 0.731 | 1 | 926 |
| REACTOME_GRB2_EVENTS_IN_ERBB2_SIGNALING | 9 | 0.450 | 1.001 | 0.464 | 0.737 | 1 | 695 |
| REACTOME_INWARDLY_RECTIFYING_K_CHANNELS | 13 | 0.407 | 0.999 | 0.464 | 0.740 | 1 | 266 |
| REACTOME_THROMBOXANE_SIGNALLING_THROUGH_TP_RECEPTOR | 11 | 0.421 | 0.998 | 0.466 | 0.742 | 1 | 1425 |
| REACTOME_SUMOYLATION_OF_DNA_METHYLATION_PROTEINS | 16 | 0.379 | 0.997 | 0.444 | 0.742 | 1 | 3853 |
| REACTOME_PHOSPHORYLATION_OF_EMI1 | 6 | 0.499 | 0.993 | 0.508 | 0.751 | 1 | 4176 |
| REACTOME_G_BETA_GAMMA_SIGNALLING_THROUGH_PI3KGAMMA | 12 | 0.415 | 0.989 | 0.481 | 0.759 | 1 | 3080 |
| REACTOME_PROTEIN_UBIQUITINATION | 65 | 0.281 | 0.989 | 0.469 | 0.757 | 1 | 4659 |
| REACTOME_CHK1_CHK2_CDS1_MEDIATED_INACTIVATION_OF_CYCLIN_B_CDK1_COMPLEX | 13 | 0.398 | 0.989 | 0.488 | 0.757 | 1 | 3484 |
| REACTOME_ERYTHROPOIETIN_ACTIVATES_PHOSPHOLIPASE_C_GAMMA_PLCG_ | 6 | 0.486 | 0.987 | 0.503 | 0.760 | 1 | 3168 |
| REACTOME_PROCESSING_AND_ACTIVATION_OF_SUMO | 10 | 0.424 | 0.987 | 0.470 | 0.760 | 1 | 4753 |
| REACTOME_G_ALPHA_S_SIGNALLING_EVENTS | 51 | 0.292 | 0.986 | 0.489 | 0.760 | 1 | 2618 |
| REACTOME_CD22_MEDIATED_BCR_REGULATION | 2 | 0.659 | 0.985 | 0.551 | 0.762 | 1 | 4340 |
| REACTOME_DOWNREGULATION_OF_ERBB2_SIGNALING | 21 | 0.349 | 0.982 | 0.492 | 0.768 | 1 | 2159 |
| REACTOME_ADP_SIGNALLING_THROUGH_P2Y_PURINOCEPTOR_1 | 11 | 0.418 | 0.981 | 0.482 | 0.768 | 1 | 2618 |
| REACTOME_ACTIVATION_OF_NIMA_KINASES_NEK9_NEK6_NEK7 | 7 | 0.466 | 0.981 | 0.507 | 0.768 | 1 | 4176 |
| REACTOME_GLUCOCORTICOID_BIOSYNTHESIS | 2 | 0.654 | 0.980 | 0.561 | 0.768 | 1 | 4197 |
| REACTOME_TRYPTOPHAN_CATABOLISM | 8 | 0.452 | 0.979 | 0.501 | 0.770 | 1 | 3635 |

| | | | | | | | |
|---|---|---|---|---|---|---|---|
| REACTOME_REGULATION_OF_PTEN_MRNA_TRANSLATION | 9 | 0.440 | 0.978 | 0.505 | 0.770 | 1 | 1061 |
| REACTOME_DISEASES_OF_CARBOHYDRATE_METABOLISM | 27 | 0.328 | 0.978 | 0.491 | 0.770 | 1 | 1498 |
| REACTOME_ORGANIC_CATION_ANION_ZWITTERION_TRANSPORT | 6 | 0.483 | 0.977 | 0.506 | 0.770 | 1 | 121 |
| REACTOME_RUNX1_REGULATES_ESTROGEN_RECEPTOR_MEDIATED_TRANSCRIPTION | 5 | 0.511 | 0.977 | 0.510 | 0.769 | 1 | 3249 |
| REACTOME_JNK_C_JUN_KINASES_PHOSPHORYLATION_AND_ACTIVATION_MEDIATED_BY_ACTIVATED_HUMAN_TAK1 | 21 | 0.343 | 0.977 | 0.480 | 0.768 | 1 | 3849 |
| REACTOME_OTHER_INTERLEUKIN_SIGNALING | 16 | 0.375 | 0.975 | 0.493 | 0.772 | 1 | 3528 |
| REACTOME_TP53_REGULATES_TRANSCRIPTION_OF_DEATH_RECEPTORS_AND_LIGANDS | 10 | 0.431 | 0.975 | 0.513 | 0.772 | 1 | 2084 |
| REACTOME_SIGNALING_BY_NOTCH | 166 | 0.239 | 0.973 | 0.521 | 0.774 | 1 | 2163 |
| REACTOME_SYNTHESIS_OF_IP3_AND_IP4_IN_THE_CYTOSOL | 20 | 0.347 | 0.973 | 0.498 | 0.774 | 1 | 821 |
| REACTOME_PTK6_REGULATES_PROTEINS_INVOLVED_IN_RNA_PROCESSING | 4 | 0.540 | 0.972 | 0.548 | 0.774 | 1 | 3521 |
| REACTOME_NOTCH4_INTRACELLULAR_DOMAIN_REGULATES_TRANSCRIPTION | 17 | 0.362 | 0.972 | 0.478 | 0.773 | 1 | 4374 |
| REACTOME_PHASE_II_CONJUGATION_OF_COMPOUNDS | 51 | 0.285 | 0.968 | 0.498 | 0.782 | 1 | 1419 |
| REACTOME_REGULATION_OF_KIT_SIGNALING | 15 | 0.390 | 0.967 | 0.505 | 0.782 | 1 | 1580 |
| REACTOME_INTERFERON_ALPHA_BETA_SIGNALING | 47 | 0.287 | 0.965 | 0.499 | 0.785 | 1 | 1150 |
| REACTOME_TRANSCRIPTIONAL_REGULATION_BY_MECP2 | 46 | 0.296 | 0.965 | 0.522 | 0.784 | 1 | 1146 |
| REACTOME_ORGANIC_CATION_TRANSPORT | 6 | 0.483 | 0.965 | 0.546 | 0.784 | 1 | 121 |
| REACTOME_SMALL_INTERFERING_RNA_SIRNA_BIOGENESIS | 9 | 0.435 | 0.964 | 0.506 | 0.784 | 1 | 195 |
| REACTOME_FGFR2_ALTERNATIVE_SPLICING | 26 | 0.325 | 0.961 | 0.509 | 0.791 | 1 | 4379 |
| REACTOME_NUCLEOTIDE_SALVAGE | 19 | 0.356 | 0.959 | 0.515 | 0.793 | 1 | 3878 |
| REACTOME_SIGNALING_BY_ERBB2 | 41 | 0.299 | 0.957 | 0.507 | 0.798 | 1 | 1170 |
| REACTOME_DISASSEMBLY_OF_THE_DESTRUCTION_COMPLEX_AND_RECRUITMENT_OF_AXIN_TO_THE_MEMBRANE | 26 | 0.326 | 0.957 | 0.490 | 0.797 | 1 | 267 |

| | | | | | | | |
|---|---|---|---|---|---|---|---|
| REACTOME_METABOLISM_OF_ANGIOTENSINOGEN_TO_ANGIOTENSINS | 7 | 0.462 | 0.953 | 0.537 | 0.803 | 1 | 2765 |
| REACTOME_SIGNALING_BY_SCF_KIT | 38 | 0.299 | 0.953 | 0.537 | 0.804 | 1 | 3235 |
| REACTOME_INSERTION_OF_TAIL_ANCHORED_PROTEINS_INTO_THE_ENDOPLASMIC_RETICULUM_MEMBRANE | 21 | 0.340 | 0.951 | 0.530 | 0.806 | 1 | 2256 |
| REACTOME_HIV_LIFE_CYCLE | 144 | 0.237 | 0.949 | 0.578 | 0.809 | 1 | 3870 |
| REACTOME_PROTEIN_REPAIR | 6 | 0.487 | 0.949 | 0.564 | 0.809 | 1 | 4278 |
| REACTOME_SUMOYLATION_OF_RNA_BINDING_PROTEINS | 47 | 0.289 | 0.947 | 0.545 | 0.811 | 1 | 4177 |
| REACTOME_RUNX1_REGULATES_TRANSCRIPTION_OF_GENES_INVOLVED_IN_INTERLEUKIN_SIGNALING | 3 | 0.579 | 0.946 | 0.577 | 0.812 | 1 | 3249 |
| REACTOME_TRANSCRIPTIONAL_ACTIVATION_OF_MITOCHONDRIAL_BIOGENESIS | 51 | 0.275 | 0.943 | 0.587 | 0.819 | 1 | 3542 |
| REACTOME_PROLACTIN_RECEPTOR_SIGNALING | 10 | 0.410 | 0.942 | 0.557 | 0.819 | 1 | 4551 |
| REACTOME_SIGNALING_BY_MEMBRANE_TETHERED_FUSIONS_OF_PDGFRA_OR_PDGFRB | 3 | 0.564 | 0.942 | 0.591 | 0.818 | 1 | 2586 |
| REACTOME_ABACAVIR_METABOLISM | 4 | 0.525 | 0.942 | 0.586 | 0.817 | 1 | 3236 |
| REACTOME_ASPARTATE_AND_ASPARAGINE_METABOLISM | 6 | 0.469 | 0.941 | 0.539 | 0.818 | 1 | 364 |
| REACTOME_FOXO_MEDIATED_TRANSCRIPTION_OF_CELL_DEATH_GENES | 16 | 0.359 | 0.941 | 0.545 | 0.818 | 1 | 1452 |
| REACTOME_NOTCH3_INTRACELLULAR_DOMAIN_REGULATES_TRANSCRIPTION | 19 | 0.347 | 0.939 | 0.526 | 0.821 | 1 | 4374 |
| REACTOME_REGULATION_OF_TP53_ACTIVITY_THROUGH_ASSOCIATION_WITH_CO_FACTORS | 12 | 0.386 | 0.938 | 0.540 | 0.821 | 1 | 2084 |
| REACTOME_SIGNALING_BY_FGFR1_IN_DISEASE | 28 | 0.313 | 0.938 | 0.550 | 0.821 | 1 | 5475 |
| REACTOME_PROSTACYCLIN_SIGNALLING_THROUGH_PROSTACYCLIN_RECEPTOR | 8 | 0.433 | 0.937 | 0.562 | 0.822 | 1 | 2618 |
| REACTOME_AKT_PHOSPHORYLATES_TARGETS_IN_THE_CYTOSOL | 14 | 0.365 | 0.937 | 0.513 | 0.821 | 1 | 4296 |
| REACTOME_EICOSANOIDS | 1 | 0.703 | 0.936 | 0.594 | 0.822 | 1 | 3774 |
| REACTOME_CONSTITUTIVE_SIGNALING_BY_OVEREXPRESSED_ERBB2 | 11 | 0.403 | 0.935 | 0.555 | 0.823 | 1 | 4118 |
| REACTOME_RAB_GEFS_EXCHANGE_GTP_FOR_GDP_ON_RABS | 82 | 0.253 | 0.932 | 0.595 | 0.827 | 1 | 4266 |

| | | | | | | | |
|---|---|---|---|---|---|---|---|
| REACTOME_NOD1_2_SIGNALING_PATHWAY | 35 | 0.295 | 0.929 | 0.579 | 0.833 | 1 | 2742 |
| REACTOME_TRANS_GOLGI_NETWORK_VESICLE_BUDDING | 68 | 0.256 | 0.929 | 0.590 | 0.833 | 1 | 4144 |
| REACTOME_INTERFERON_GAMMA_SIGNALING | 57 | 0.269 | 0.928 | 0.589 | 0.834 | 1 | 3533 |
| REACTOME_NOTCH1_INTRACELLULAR_DOMAIN_REGULATES_TRANSCRIPTION | 45 | 0.282 | 0.927 | 0.579 | 0.835 | 1 | 2159 |
| REACTOME_G_BETA_GAMMA_SIGNALLING_THROUGH_CDC42 | 9 | 0.416 | 0.923 | 0.557 | 0.842 | 1 | 3067 |
| REACTOME_TNF_RECEPTOR_SUPERFAMILY_TNFSF_MEMBERS_MEDIATING_NON_CANONICAL_NF_KB_PATHWAY | 8 | 0.427 | 0.921 | 0.586 | 0.847 | 1 | 3185 |
| REACTOME_GABA_B_RECEPTOR_ACTIVATION | 19 | 0.341 | 0.917 | 0.586 | 0.853 | 1 | 266 |
| REACTOME_ANTIVIRAL_MECHANISM_BY_IFN_STIMULATED_GENES | 74 | 0.253 | 0.917 | 0.624 | 0.852 | 1 | 3870 |
| REACTOME_SIGNALING_BY_NOTCH1 | 66 | 0.258 | 0.915 | 0.619 | 0.855 | 1 | 1291 |
| REACTOME_N_GLYCAN_TRIMMING_IN_THE_ER_AND_CALNEXIN_CALRETICULIN_CYCLE | 35 | 0.293 | 0.915 | 0.577 | 0.854 | 1 | 3899 |
| REACTOME_MET_RECEPTOR_RECYCLING | 7 | 0.442 | 0.913 | 0.571 | 0.858 | 1 | 3687 |
| REACTOME_MECP2_REGULATES_TRANSCRIPTION_FACTORS | 3 | 0.554 | 0.913 | 0.625 | 0.857 | 1 | 5367 |
| REACTOME_BUDDING_AND_MATURATION_OF_HIV_VIRION | 28 | 0.304 | 0.910 | 0.596 | 0.861 | 1 | 4257 |
| REACTOME_IKK_COMPLEX_RECRUITMENT_MEDIATED_BY_RIP1 | 21 | 0.322 | 0.909 | 0.605 | 0.862 | 1 | 3094 |
| REACTOME_ROLE_OF_SECOND_MESSENGERS_IN_NETRIN_1_SIGNALING | 3 | 0.563 | 0.909 | 0.647 | 0.862 | 1 | 1754 |
| REACTOME_FORMATION_OF_RNA_POL_II_ELONGATION_COMPLEX_ | 56 | 0.266 | 0.909 | 0.596 | 0.861 | 1 | 3815 |
| REACTOME_BETA_CATENIN_PHOSPHORYLATION_CASCADE | 16 | 0.346 | 0.908 | 0.577 | 0.861 | 1 | 4060 |
| REACTOME_TRANSPORT_OF_THE_SLBP_DEPENDANT_MATURE_MRNA | 36 | 0.283 | 0.908 | 0.619 | 0.861 | 1 | 4377 |
| REACTOME_NRCAM_INTERACTIONS | 6 | 0.455 | 0.907 | 0.603 | 0.862 | 1 | 2027 |
| REACTOME_ANTIGEN_PRESENTATION_FOLDING_ASSEMBLY_AND_PEPTIDE_LOADING_OF_CLASS_I_MHC | 24 | 0.310 | 0.906 | 0.594 | 0.862 | 1 | 1068 |
| REACTOME_BH3_ONLY_PROTEINS_ASSOCIATE_WITH_AND_INA | 9 | 0.401 | 0.905 | 0.592 | 0.864 | 1 | 704 |

| | | | | | | | |
|---|---|---|---|---|---|---|---|
| CTIVATE_ANTI_APOPTOTIC_BCL_2_MEMBERS | | | | | | | |
| REACTOME_CREB1_PHOSPHORYLATION_THROUGH_THE_ACTIVATION_OF_ADENYLATE_CYCLASE | 8 | 0.413 | 0.903 | 0.588 | 0.866 | 1 | 779 |
| REACTOME_SIGNAL_AMPLIFICATION | 16 | 0.338 | 0.902 | 0.602 | 0.868 | 1 | 3050 |
| REACTOME_HIV_ELONGATION_ARREST_AND_RECOVERY | 32 | 0.297 | 0.901 | 0.620 | 0.868 | 1 | 4042 |
| REACTOME_CELLULAR_RESPONSE_TO_HYPOXIA | 70 | 0.252 | 0.900 | 0.659 | 0.870 | 1 | 3159 |
| REACTOME_TERMINATION_OF_O_GLYCAN_BIOSYNTHESIS | 8 | 0.420 | 0.894 | 0.605 | 0.882 | 1 | 2813 |
| REACTOME_TRANSCRIPTIONAL_REGULATION_OF_GRANULOPOIESIS | 35 | 0.283 | 0.893 | 0.625 | 0.882 | 1 | 1978 |
| REACTOME_G0_AND_EARLY_G1 | 26 | 0.301 | 0.893 | 0.623 | 0.882 | 1 | 2647 |
| REACTOME_PURINE_RIBONUCLEOSIDE_MONOPHOSPHATE_BIOSYNTHESIS | 12 | 0.364 | 0.892 | 0.608 | 0.882 | 1 | 676 |
| REACTOME_GLUCOSE_METABOLISM | 77 | 0.250 | 0.890 | 0.668 | 0.886 | 1 | 3995 |
| REACTOME_RUNX3_REGULATES_WNT_SIGNALING | 8 | 0.406 | 0.889 | 0.607 | 0.886 | 1 | 2079 |
| REACTOME_KILLING_MECHANISMS | 9 | 0.396 | 0.888 | 0.627 | 0.888 | 1 | 2419 |
| REACTOME_TRANSCRIPTIONAL_REGULATION_BY_TP53 | 343 | 0.203 | 0.887 | 0.803 | 0.889 | 1 | 3232 |
| REACTOME_RUNX3_REGULATES_NOTCH_SIGNALING | 14 | 0.349 | 0.884 | 0.633 | 0.895 | 1 | 4374 |
| REACTOME_TRANSPORT_OF_INORGANIC_CATIONS_ANIONS_AND_AMINO_ACIDS_OLIGOPEPTIDES | 53 | 0.258 | 0.883 | 0.667 | 0.895 | 1 | 3233 |
| REACTOME_ACTIVATION_OF_PUMA_AND_TRANSLOCATION_TO_MITOCHONDRIA | 8 | 0.405 | 0.882 | 0.621 | 0.895 | 1 | 211 |
| REACTOME_AMINE_LIGAND_BINDING_RECEPTORS | 6 | 0.440 | 0.880 | 0.647 | 0.899 | 1 | 172 |
| REACTOME_G1_S_DNA_DAMAGE_CHECKPOINTS | 65 | 0.250 | 0.879 | 0.723 | 0.900 | 1 | 462 |
| REACTOME_GLUCAGON_TYPE_LIGAND_RECEPTORS | 11 | 0.362 | 0.877 | 0.613 | 0.902 | 1 | 2618 |
| REACTOME_GP1B_IX_V_ACTIVATION_SIGNALLING | 7 | 0.422 | 0.877 | 0.621 | 0.902 | 1 | 5244 |
| REACTOME_SYNTHESIS_OF_VERY_LONG_CHAIN_FATTY_ACYL_COAS | 15 | 0.342 | 0.876 | 0.614 | 0.901 | 1 | 2450 |
| REACTOME_POU5F1_OCT4_SOX2_NANOG_REPRESS_GENES_RELATED_TO_DIFFERENTIATION | 7 | 0.414 | 0.874 | 0.637 | 0.906 | 1 | 10 |
| REACTOME_TRANSPORT_OF_NUCLEOSIDES_AND_FREE_PURINE | 9 | 0.385 | 0.872 | 0.630 | 0.908 | 1 | 2562 |

| | | | | | | | |
|---|---|---|---|---|---|---|---|
| _AND_PYRIMIDINE_BASES_ACROSS_THE_PLASMA_MEMBRANE | | | | | | | |
| REACTOME_MET_ACTIVATES_PI3K_AKT_SIGNALING | 4 | 0.476 | 0.871 | 0.634 | 0.908 | 1 | 1146 |
| REACTOME_RESPONSE_OF_EIF2AK1_HRI_TO_HEME_DEFICIENCY | 15 | 0.338 | 0.871 | 0.635 | 0.907 | 1 | 2459 |
| REACTOME_REGULATION_OF_IFNA_SIGNALING | 12 | 0.369 | 0.871 | 0.655 | 0.907 | 1 | 16 |
| REACTOME_SPHINGOLIPID_METABOLISM | 71 | 0.240 | 0.871 | 0.708 | 0.906 | 1 | 2720 |
| REACTOME_RNA_POLYMERASE_III_TRANSCRIPTION_INITIATION_FROM_TYPE_1_PROMOTER | 28 | 0.292 | 0.869 | 0.674 | 0.909 | 1 | 1742 |
| REACTOME_CASPASE_ACTIVATION_VIA_EXTRINSIC_APOPTOTIC_SIGNALLING_PATHWAY | 21 | 0.308 | 0.868 | 0.668 | 0.909 | 1 | 2464 |
| REACTOME_DECTIN_2_FAMILY | 7 | 0.425 | 0.868 | 0.652 | 0.909 | 1 | 341 |
| REACTOME_PEXOPHAGY | 11 | 0.371 | 0.867 | 0.621 | 0.909 | 1 | 2159 |
| REACTOME_SYNTHESIS_OF_PC | 23 | 0.307 | 0.867 | 0.631 | 0.908 | 1 | 766 |
| REACTOME_HIV_INFECTION | 218 | 0.205 | 0.867 | 0.823 | 0.908 | 1 | 3870 |
| REACTOME_HOST_INTERACTIONS_OF_HIV_FACTORS | 123 | 0.221 | 0.864 | 0.756 | 0.913 | 1 | 3019 |
| REACTOME_COMPLEMENT_CASCADE | 15 | 0.338 | 0.863 | 0.667 | 0.912 | 1 | 2708 |
| REACTOME_NS1_MEDIATED_EFFECTS_ON_HOST_PATHWAYS | 40 | 0.268 | 0.863 | 0.699 | 0.912 | 1 | 2107 |
| REACTOME_ASSOCIATION_OF_TRIC_CCT_WITH_TARGET_PROTEINS_DURING_BIOSYNTHESIS | 35 | 0.272 | 0.863 | 0.676 | 0.911 | 1 | 3770 |
| REACTOME_SYNTHESIS_OF_PA | 27 | 0.288 | 0.863 | 0.692 | 0.910 | 1 | 2469 |
| REACTOME_RAS_PROCESSING | 23 | 0.300 | 0.861 | 0.649 | 0.912 | 1 | 4927 |
| REACTOME_PHASE_1_INACTIVATION_OF_FAST_NA_CHANNELS | 3 | 0.525 | 0.859 | 0.695 | 0.915 | 1 | 594 |
| REACTOME_GLYCOGEN_STORAGE_DISEASES | 12 | 0.366 | 0.857 | 0.668 | 0.918 | 1 | 3842 |
| REACTOME_FERTILIZATION | 6 | 0.431 | 0.856 | 0.655 | 0.918 | 1 | 1562 |
| REACTOME_NOTCH_HLH_TRANSCRIPTION_PATHWAY | 27 | 0.291 | 0.856 | 0.674 | 0.918 | 1 | 4473 |
| REACTOME_PYRUVATE_METABOLISM | 26 | 0.293 | 0.856 | 0.677 | 0.917 | 1 | 2750 |
| REACTOME_BIOSYNTHESIS_OF_EPA_DERIVED_SPMS | 2 | 0.579 | 0.856 | 0.707 | 0.916 | 1 | 4627 |
| REACTOME_ANTIGEN_ACTIVATES_B_CELL_RECEPTOR_BCR_LEADING_TO_GENERATION_OF_SECOND_MESSENGERS | 22 | 0.303 | 0.854 | 0.675 | 0.918 | 1 | 3235 |
| REACTOME_KERATAN_SULFATE_BIOSYNTHESIS | 17 | 0.320 | 0.852 | 0.658 | 0.920 | 1 | 2876 |
| REACTOME_LYSINE_CATABOLISM | 10 | 0.378 | 0.852 | 0.659 | 0.920 | 1 | 2973 |
| REACTOME_SIGNALING_BY_NOTCH3 | 40 | 0.266 | 0.852 | 0.710 | 0.919 | 1 | 2163 |

| | | | | | | | |
|---|---|---|---|---|---|---|---|
| REACTOME_SYNTHESIS_OF_PYROPHOSPHATES_IN_THE_CYTOSOL | 7 | 0.418 | 0.850 | 0.654 | 0.921 | 1 | 3487 |
| REACTOME_SIGNALING_BY_FGFR3_FUSIONS_IN_CANCER | 9 | 0.379 | 0.850 | 0.670 | 0.921 | 1 | 4118 |
| REACTOME_NEDDYLATION | 208 | 0.201 | 0.848 | 0.874 | 0.923 | 1 | 3506 |
| REACTOME_PHOSPHOLIPID_METABOLISM | 165 | 0.206 | 0.847 | 0.815 | 0.924 | 1 | 2725 |
| REACTOME_TCF_DEPENDENT_SIGNALING_IN_RESPONSE_TO_WNT | 150 | 0.211 | 0.845 | 0.797 | 0.927 | 1 | 2240 |
| REACTOME_REGULATION_OF_LIPID_METABOLISM_BY_PPARALPHA | 105 | 0.221 | 0.844 | 0.807 | 0.927 | 1 | 1671 |
| REACTOME_SERINE_BIOSYNTHESIS | 9 | 0.380 | 0.843 | 0.660 | 0.927 | 1 | 3160 |
| REACTOME_SPERM_MOTILITY_AND_TAXES | 2 | 0.570 | 0.843 | 0.751 | 0.927 | 1 | 823 |
| REACTOME_EUKARYOTIC_TRANSLATION_ELONGATION | 89 | 0.228 | 0.842 | 0.749 | 0.928 | 1 | 5937 |
| REACTOME_MTORC1_MEDIATED_SIGNALLING | 23 | 0.297 | 0.842 | 0.698 | 0.927 | 1 | 2812 |
| REACTOME_SUMO_IS_TRANSFERRED_FROM_E1_TO_E2_UBE2I_UBC9_ | 7 | 0.401 | 0.842 | 0.677 | 0.926 | 1 | 4423 |
| REACTOME_ACTIVATED_NTRK2_SIGNALS_THROUGH_RAS | 7 | 0.409 | 0.841 | 0.696 | 0.926 | 1 | 4118 |
| REACTOME_HIV_TRANSCRIPTION_ELONGATION | 41 | 0.260 | 0.839 | 0.742 | 0.929 | 1 | 4042 |
| REACTOME_FATTY_ACIDS | 2 | 0.574 | 0.838 | 0.736 | 0.930 | 1 | 1352 |
| REACTOME_AMYLOID_FIBER_FORMATION | 42 | 0.261 | 0.836 | 0.728 | 0.932 | 1 | 2333 |
| REACTOME_DIGESTION_OF_DIETARY_LIPID | 1 | 0.628 | 0.836 | 0.738 | 0.931 | 1 | 4730 |
| REACTOME_MITOPHAGY | 28 | 0.282 | 0.834 | 0.696 | 0.933 | 1 | 4201 |
| REACTOME_IRAK4_DEFICIENCY_TLR2_4_ | 5 | 0.436 | 0.834 | 0.672 | 0.933 | 1 | 3437 |
| REACTOME_HDMS_DEMETHYLATE_HISTONES | 21 | 0.303 | 0.833 | 0.718 | 0.933 | 1 | 3856 |
| REACTOME_DEFECTIVE_GALNT3_CAUSES_FAMILIAL_HYPERPHOSPHATEMIC_TUMORAL_CALCINOSIS_HFTC_ | 4 | 0.471 | 0.828 | 0.715 | 0.941 | 1 | 341 |
| REACTOME_DEATH_RECEPTOR_SIGNALLING | 126 | 0.211 | 0.823 | 0.862 | 0.949 | 1 | 3849 |
| REACTOME_SHC_RELATED_EVENTS_TRIGGERED_BY_IGF1R | 7 | 0.395 | 0.820 | 0.716 | 0.953 | 1 | 4118 |
| REACTOME_MASTL_FACILITATES_MITOTIC_PROGRESSION | 10 | 0.357 | 0.820 | 0.680 | 0.952 | 1 | 2479 |
| REACTOME_ERYTHROCYTES_TAKE_UP_CARBON_DIOXIDE_AND_RELEASE_OXYGEN | 6 | 0.404 | 0.817 | 0.700 | 0.956 | 1 | 358 |

| | | | | | | | |
|---|---|---|---|---|---|---|---|
| REACTOME_TRANSLATION_OF_REPLICASE_AND_ASSEMBLY_OF_THE_REPLICATION_TRANSCRIPTION_COMPLEX | 13 | 0.335 | 0.817 | 0.696 | 0.956 | 1 | 2949 |
| REACTOME_BETA_OXIDATION_OF_PRISTANOYL_COA | 8 | 0.377 | 0.817 | 0.720 | 0.955 | 1 | 2748 |
| REACTOME_NOTCH3_ACTIVATION_AND_TRANSMISSION_OF_SIGNAL_TO_THE_NUCLEUS | 21 | 0.288 | 0.816 | 0.725 | 0.955 | 1 | 1273 |
| REACTOME_APOPTOTIC_CLEAVAGE_OF_CELL_ADHESION_PROTEINS | 8 | 0.375 | 0.815 | 0.697 | 0.955 | 1 | 3441 |
| REACTOME_SIGNALING_BY_CYTOSOLIC_FGFR1_FUSION_MUTANTS | 18 | 0.298 | 0.813 | 0.704 | 0.958 | 1 | 5475 |
| REACTOME_DOPAMINE_NEUROTRANSMITTER_RELEASE_CYCLE | 14 | 0.323 | 0.813 | 0.741 | 0.957 | 1 | 2674 |
| REACTOME_ACTIVATION_OF_NOXA_AND_TRANSLOCATION_TO_MITOCHONDRIA | 5 | 0.414 | 0.811 | 0.685 | 0.959 | 1 | 2084 |
| REACTOME_CA2_PATHWAY | 45 | 0.243 | 0.810 | 0.785 | 0.960 | 1 | 2079 |
| REACTOME_SUMOYLATION_OF_UBIQUITINYLATION_PROTEINS | 39 | 0.255 | 0.810 | 0.773 | 0.959 | 1 | 4377 |
| REACTOME_REGULATION_OF_PYRUVATE_DEHYDROGENASE_PDH_COMPLEX | 15 | 0.312 | 0.809 | 0.718 | 0.958 | 1 | 2713 |
| REACTOME_MODULATION_BY_MTB_OF_HOST_IMMUNE_SYSTEM | 6 | 0.412 | 0.809 | 0.701 | 0.958 | 1 | 3842 |
| REACTOME_SCAVENGING_BY_CLASS_F_RECEPTORS | 4 | 0.449 | 0.806 | 0.715 | 0.962 | 1 | 1484 |
| REACTOME_NUCLEAR_PORE_COMPLEX_NPC_DISASSEMBLY | 36 | 0.256 | 0.805 | 0.775 | 0.961 | 1 | 2107 |
| REACTOME_CD209_DC_SIGN_SIGNALING | 18 | 0.302 | 0.805 | 0.738 | 0.961 | 1 | 4927 |
| REACTOME_SUMOYLATION_OF_CHROMATIN_ORGANIZATION_PROTEINS | 57 | 0.232 | 0.803 | 0.804 | 0.964 | 1 | 3995 |
| REACTOME_GOLGI_ASSOCIATED_VESICLE_BIOGENESIS | 54 | 0.233 | 0.801 | 0.805 | 0.965 | 1 | 4952 |
| REACTOME_INTERACTION_BETWEEN_L1_AND_ANKYRINS | 16 | 0.308 | 0.801 | 0.744 | 0.965 | 1 | 2709 |
| REACTOME_GLUCONEOGENESIS | 25 | 0.278 | 0.799 | 0.775 | 0.967 | 1 | 3792 |
| REACTOME_NEF_MEDIATED_DOWNREGULATION_OF_MHC_CLASS_I_COMPLEX_CELL_SURFACE_EXPRESSION | 9 | 0.368 | 0.797 | 0.737 | 0.969 | 1 | 4650 |
| REACTOME_SUMOYLATION_OF_DNA_REPLICATION_PROTEINS | 46 | 0.241 | 0.795 | 0.812 | 0.970 | 1 | 4377 |
| REACTOME_FCGR3A_MEDIATED_IL10_SYNTHESIS | 24 | 0.275 | 0.794 | 0.748 | 0.971 | 1 | 779 |

| | | | | | | | |
|---|---|---|---|---|---|---|---|
| REACTOME_TNFR1_MEDIATED_CERAMIDE_PRODUCTION | 4 | 0.440 | 0.792 | 0.732 | 0.973 | 1 | 4494 |
| REACTOME_RESOLUTION_OF_SISTER_CHROMATID_COHESION | 116 | 0.204 | 0.792 | 0.900 | 0.972 | 1 | 2549 |
| REACTOME_ACTIVATION_OF_THE_MRNA_UPON_BINDING_OF_THE_CAP_BINDING_COMPLEX_AND_EIFS_AND_SUBSEQUENT_BINDING_TO_43S | 56 | 0.228 | 0.791 | 0.817 | 0.973 | 1 | 9316 |
| REACTOME_G_ALPHA_Z_SIGNALLING_EVENTS | 24 | 0.273 | 0.789 | 0.776 | 0.974 | 1 | 3369 |
| REACTOME_VLDL_CLEARANCE | 3 | 0.474 | 0.785 | 0.747 | 0.980 | 1 | 685 |
| REACTOME_MICRORNA_MIRNA_BIOGENESIS | 24 | 0.272 | 0.781 | 0.787 | 0.984 | 1 | 2830 |
| REACTOME_TP53_REGULATES_TRANSCRIPTION_OF_ADDITIONAL_CELL_CYCLE_GENES_WHOSE_EXACT_ROLE_IN_THE_P53_PATHWAY_REMAIN_UNCERTAIN | 21 | 0.281 | 0.779 | 0.793 | 0.986 | 1 | 2287 |
| REACTOME_CONSTITUTIVE_SIGNALING_BY_LIGAND_RESPONSIVE_EGFR_CANCER_VARIANTS | 16 | 0.297 | 0.779 | 0.785 | 0.985 | 1 | 4118 |
| REACTOME_SIGNALING_BY_PDGFR_IN_DISEASE | 17 | 0.290 | 0.779 | 0.786 | 0.985 | 1 | 4118 |
| REACTOME_VIRAL_MESSENGER_RNA_SYNTHESIS | 44 | 0.238 | 0.778 | 0.848 | 0.985 | 1 | 2107 |
| REACTOME_TAK1_ACTIVATES_NFKB_BY_PHOSPHORYLATION_AND_ACTIVATION_OF_IKKS_COMPLEX | 29 | 0.259 | 0.778 | 0.783 | 0.984 | 1 | 4296 |
| REACTOME_METHYLATION | 10 | 0.337 | 0.777 | 0.763 | 0.984 | 1 | 1419 |
| REACTOME_CHAPERONE_MEDIATED_AUTOPHAGY | 17 | 0.290 | 0.777 | 0.799 | 0.983 | 1 | 4166 |
| REACTOME_MAPK6_MAPK4_SIGNALING | 82 | 0.214 | 0.774 | 0.876 | 0.986 | 1 | 3130 |
| REACTOME_SIGNALING_BY_PDGFRA_TRANSMEMBRANE_JUXTAMEMBRANE_AND_KINASE_DOMAIN_MUTANTS | 11 | 0.321 | 0.773 | 0.754 | 0.986 | 1 | 4118 |
| REACTOME_HEDGEHOG_OFF_STATE | 97 | 0.206 | 0.772 | 0.918 | 0.986 | 1 | 910 |
| REACTOME_ELEVATION_OF_CYTOSOLIC_CA2_LEVELS | 10 | 0.341 | 0.772 | 0.762 | 0.986 | 1 | 1383 |
| REACTOME_TRANSPORT_OF_MATURE_TRANSCRIPT_TO_CYTOPLASM | 79 | 0.211 | 0.768 | 0.876 | 0.991 | 1 | 4377 |
| REACTOME_IRAK1_RECRUITS_IKK_COMPLEX | 14 | 0.308 | 0.766 | 0.762 | 0.991 | 1 | 2742 |
| REACTOME_MRNA_CAPPING | 28 | 0.262 | 0.766 | 0.796 | 0.991 | 1 | 3778 |
| REACTOME_GENOME_REPLICATION_AND_TRANSCRIPTION | 6 | 0.385 | 0.765 | 0.753 | 0.991 | 1 | 1276 |

| | | | | | | | |
|---|---|---|---|---|---|---|---|
| REACTOME_SUMOYLATION_OF_SUMOYLATION_PROTEINS | 35 | 0.245 | 0.765 | 0.846 | 0.990 | 1 | 4377 |
| REACTOME_E3_UBIQUITIN_LIGASES_UBIQUITINATE_TARGET_PROTEINS | 45 | 0.229 | 0.765 | 0.865 | 0.989 | 1 | 4659 |
| REACTOME_ENDOSOMAL_SORTING_COMPLEX_REQUIRED_FOR_TRANSPORT_ESCRT_ | 31 | 0.252 | 0.764 | 0.838 | 0.989 | 1 | 4257 |
| REACTOME_DISEASES_ASSOCIATED_WITH_N_GLYCOSYLATION_OF_PROTEINS | 17 | 0.282 | 0.761 | 0.792 | 0.993 | 1 | 2489 |
| REACTOME_TRANSPORT_OF_MATURE_MRNAS_DERIVED_FROM_INTRONLESS_TRANSCRIPTS | 43 | 0.235 | 0.760 | 0.855 | 0.992 | 1 | 4177 |
| REACTOME_FORMATION_OF_THE_EARLY_ELONGATION_COMPLEX | 32 | 0.242 | 0.756 | 0.855 | 0.997 | 1 | 3778 |
| REACTOME_SYNTHESIS_OF_PIPS_AT_THE_GOLGI_MEMBRANE | 15 | 0.291 | 0.755 | 0.801 | 0.997 | 1 | 3066 |
| REACTOME_INITIAL_TRIGGERING_OF_COMPLEMENT | 7 | 0.363 | 0.753 | 0.791 | 0.999 | 1 | 2379 |
| REACTOME_TRANSCRIPTIONAL_REGULATION_OF_WHITE_ADIPOCYTE_DIFFERENTIATION | 73 | 0.210 | 0.753 | 0.915 | 0.998 | 1 | 1043 |
| REACTOME_GLUCAGON_SIGNALING_IN_METABOLIC_REGULATION | 16 | 0.285 | 0.752 | 0.797 | 0.998 | 1 | 779 |
| REACTOME_RNA_POLYMERASE_I_TRANSCRIPTION_TERMINATION | 30 | 0.248 | 0.751 | 0.840 | 0.998 | 1 | 3764 |
| REACTOME_VITAMINS | 3 | 0.447 | 0.751 | 0.774 | 0.997 | 1 | 2118 |
| REACTOME_SCAVENGING_BY_CLASS_B_RECEPTORS | 1 | 0.564 | 0.749 | 0.896 | 0.999 | 1 | 5537 |
| REACTOME_VASOPRESSIN_REGULATES_RENAL_WATER_HOMEOSTASIS_VIA_AQUAPORINS | 21 | 0.268 | 0.748 | 0.828 | 0.999 | 1 | 2618 |
| REACTOME_DEFECTIVE_LFNG_CAUSES_SCDO3 | 4 | 0.418 | 0.746 | 0.773 | 0.999 | 1 | 7395 |
| REACTOME_DAP12_SIGNALING | 21 | 0.267 | 0.746 | 0.845 | 0.999 | 1 | 770 |
| REACTOME_TP53_REGULATES_METABOLIC_GENES | 81 | 0.203 | 0.743 | 0.934 | 1.000 | 1 | 3232 |
| REACTOME_PTEN_REGULATION | 135 | 0.187 | 0.740 | 0.964 | 1.000 | 1 | 3097 |
| REACTOME_HSF1_DEPENDENT_TRANSACTIVATION | 31 | 0.240 | 0.738 | 0.872 | 1.000 | 1 | 4406 |
| REACTOME_REGULATION_OF_TP53_EXPRESSION_AND_DEGRADATION | 35 | 0.235 | 0.738 | 0.870 | 1.000 | 1 | 3170 |
| REACTOME_DAP12_INTERACTIONS | 24 | 0.257 | 0.738 | 0.824 | 1.000 | 1 | 2804 |
| REACTOME_MECP2_REGULATES_NEURONAL_RECEPTORS_AND_CHANNELS | 13 | 0.299 | 0.737 | 0.819 | 1.000 | 1 | 1146 |

| | | | | | | | |
|---|---|---|---|---|---|---|---|
| REACTOME_SUMO_IS_CONJUGATED_TO_E1_UBA2_SAE1_ | 5 | 0.387 | 0.734 | 0.781 | 1.000 | 1 | 3483 |
| REACTOME_FOLDING_OF_ACTIN_BY_CCT_TRIC | 9 | 0.330 | 0.733 | 0.790 | 1.000 | 1 | 2601 |
| REACTOME_TETRAHYDROBIOPTERIN_BH4_SYNTHESIS_RECYCLING_SALVAGE_AND_REGULATION | 9 | 0.334 | 0.733 | 0.813 | 1.000 | 1 | 1587 |
| REACTOME_EXPORT_OF_VIRAL_RIBONUCLEOPROTEINS_FROM_NUCLEUS | 33 | 0.239 | 0.732 | 0.882 | 1.000 | 1 | 2107 |
| REACTOME_MATURATION_OF_PROTEIN_3A | 6 | 0.360 | 0.731 | 0.810 | 1.000 | 1 | 3799 |
| REACTOME_N_GLYCAN_ANTENNAE_ELONGATION_IN_THE_MEDIAL_TRANS_GOLGI | 16 | 0.287 | 0.731 | 0.843 | 1.000 | 1 | 2876 |
| REACTOME_CYTOCHROME_C_MEDIATED_APOPTOTIC_RESPONSE | 13 | 0.296 | 0.731 | 0.825 | 1.000 | 1 | 2887 |
| REACTOME_REGULATION_OF_CHOLESTEROL_BIOSYNTHESIS_BY_SREBP_SREBF_ | 55 | 0.212 | 0.730 | 0.904 | 1.000 | 1 | 1248 |
| REACTOME_PURINE_SALVAGE | 12 | 0.303 | 0.728 | 0.818 | 1.000 | 1 | 2827 |
| REACTOME_METHIONINE_SALVAGE_PATHWAY | 6 | 0.360 | 0.725 | 0.814 | 1.000 | 1 | 3471 |
| REACTOME_METABOLISM_OF_STEROID_HORMONES | 17 | 0.274 | 0.723 | 0.852 | 1.000 | 1 | 4197 |
| REACTOME_PEPTIDE_HORMONE_METABOLISM | 44 | 0.219 | 0.723 | 0.935 | 1.000 | 1 | 3527 |
| REACTOME_HUR_ELAVL1_BINDS_AND_STABILIZES_MRNA | 8 | 0.335 | 0.723 | 0.819 | 1.000 | 1 | 4159 |
| REACTOME_TP53_REGULATES_TRANSCRIPTION_OF_CASPASE_ACTIVATORS_AND_CASPASES | 8 | 0.334 | 0.723 | 0.813 | 1.000 | 1 | 2575 |
| REACTOME_MITOTIC_PROPHASE | 91 | 0.191 | 0.722 | 0.946 | 1.000 | 1 | 4458 |
| REACTOME_NECTIN_NECL_TRANS_HETERODIMERIZATION | 4 | 0.403 | 0.721 | 0.805 | 1.000 | 1 | 2269 |
| REACTOME_PHOSPHATE_BOND_HYDROLYSIS_BY_NUDT_PROTEINS | 7 | 0.342 | 0.719 | 0.837 | 1.000 | 1 | 537 |
| REACTOME_SYNTHESIS_OF_12_EICOSATETRAENOIC_ACID_DERIVATIVES | 5 | 0.381 | 0.715 | 0.789 | 1.000 | 1 | 1084 |
| REACTOME_DCC_MEDIATED_ATTRACTIVE_SIGNALING | 12 | 0.297 | 0.714 | 0.845 | 1.000 | 1 | 312 |
| REACTOME_PYRUVATE_METABOLISM_AND_CITRIC_ACID_TCA_CYCLE | 49 | 0.215 | 0.713 | 0.902 | 1.000 | 1 | 2973 |
| REACTOME_SIGNALING_BY_HEDGEHOG | 123 | 0.184 | 0.713 | 0.979 | 1.000 | 1 | 910 |
| REACTOME_ATTENUATION_PHASE | 23 | 0.251 | 0.712 | 0.872 | 1.000 | 1 | 4406 |

| | | | | | | | |
|---|---|---|---|---|---|---|---|
| REACTOME_DISEASES_ASSOCIATED_WITH_SURFACTANT_METABOLISM | 1 | 0.539 | 0.709 | 0.933 | 1.000 | 1 | 5856 |
| REACTOME_SIGNALING_BY_BMP | 16 | 0.268 | 0.709 | 0.847 | 1.000 | 1 | 3722 |
| REACTOME_LRR_FLII_INTERACTING_PROTEIN_1_LRRFIP1_ACTIVATES_TYPE_I_IFN_PRODUCTION | 5 | 0.367 | 0.709 | 0.803 | 1.000 | 1 | 4925 |
| REACTOME_CLEC7A_DECTIN_1_INDUCES_NFAT_ACTIVATION | 11 | 0.294 | 0.704 | 0.842 | 1.000 | 1 | 1710 |
| REACTOME_ADP_SIGNALLING_THROUGH_P2Y_PURINOCEPTOR_12 | 10 | 0.301 | 0.702 | 0.825 | 1.000 | 1 | 3050 |
| REACTOME_N_GLYCAN_ANTENNAE_ELONGATION | 10 | 0.302 | 0.702 | 0.854 | 1.000 | 1 | 2876 |
| REACTOME_SIGNALING_BY_NOTCH1_T_7_9_NOTCH1_M1580_K2555_TRANSLOCATION_MUTANT | 7 | 0.342 | 0.701 | 0.826 | 1.000 | 1 | 472 |
| REACTOME_CELLULAR_RESPONSE_TO_HEAT_STRESS | 93 | 0.187 | 0.701 | 0.978 | 1.000 | 1 | 4406 |
| REACTOME_SIGNALING_BY_THE_B_CELL_RECEPTOR_BCR_ | 97 | 0.185 | 0.700 | 0.972 | 1.000 | 1 | 4340 |
| REACTOME_RNA_POLYMERASE_III_TRANSCRIPTION | 40 | 0.220 | 0.700 | 0.926 | 1.000 | 1 | 1742 |
| REACTOME_CLASS_I_MHC_MEDIATED_ANTIGEN_PROCESSING_PRESENTATION | 319 | 0.161 | 0.699 | 1.000 | 1.000 | 1 | 4026 |
| REACTOME_CARGO_TRAFFICKING_TO_THE_PERICILIARY_MEMBRANE | 43 | 0.210 | 0.696 | 0.943 | 1.000 | 1 | 3269 |
| REACTOME_HATS_ACETYLATE_HISTONES | 85 | 0.187 | 0.694 | 0.966 | 1.000 | 1 | 3159 |
| REACTOME_PINK1_PRKN_MEDIATED_MITOPHAGY | 21 | 0.248 | 0.693 | 0.899 | 1.000 | 1 | 2159 |
| REACTOME_NEUROTOXICITY_OF_CLOSTRIDIUM_TOXINS | 6 | 0.349 | 0.691 | 0.829 | 1.000 | 1 | 2256 |
| REACTOME_MULTIFUNCTIONAL_ANION_EXCHANGERS | 4 | 0.388 | 0.690 | 0.844 | 1.000 | 1 | 1633 |
| REACTOME_SCF_SKP2_MEDIATED_DEGRADATION_OF_P27_P21 | 59 | 0.195 | 0.689 | 0.961 | 1.000 | 1 | 1052 |
| REACTOME_ACTIVATION_OF_C3_AND_C5 | 5 | 0.360 | 0.686 | 0.822 | 1.000 | 1 | 2379 |
| REACTOME_DNA_DAMAGE_TELOMERE_STRESS_INDUCED_SENESCENCE | 38 | 0.215 | 0.686 | 0.931 | 1.000 | 1 | 462 |
| REACTOME_COOPERATION_OF_PDCL_PHLP1_AND_TRIC_CCT_IN_G_PROTEIN_BETA_FOLDING | 24 | 0.241 | 0.686 | 0.888 | 1.000 | 1 | 3369 |
| REACTOME_MITOCHONDRIAL_BIOGENESIS | 87 | 0.184 | 0.685 | 0.980 | 1.000 | 1 | 3361 |
| REACTOME_TRANSCRIPTION_OF_THE_HIV_GENOME | 66 | 0.195 | 0.682 | 0.963 | 1.000 | 1 | 3815 |

| | | | | | | | |
|---|---|---|---|---|---|---|---|
| REACTOME_OVARIAN_TUMOR_DOMAIN_PROTEASES | 34 | 0.221 | 0.682 | 0.927 | 1.000 | 1 | 3082 |
| REACTOME_NUCLEOTIDE_LIKE_PURINERGIC_RECEPTORS | 5 | 0.350 | 0.681 | 0.873 | 1.000 | 1 | 948 |
| REACTOME_INFLUENZA_INFECTION | 151 | 0.168 | 0.680 | 0.991 | 1.000 | 1 | 5337 |
| REACTOME_SIGNALING_BY_ROBO_RECEPTORS | 191 | 0.164 | 0.680 | 0.999 | 1.000 | 1 | 5544 |
| REACTOME_ORGANIC_ANION_TRANSPORTERS | 3 | 0.416 | 0.677 | 0.859 | 1.000 | 1 | 1172 |
| REACTOME_ACTIVATED_NOTCH1_TRANSMITS_SIGNAL_TO_THE_NUCLEUS | 26 | 0.233 | 0.677 | 0.903 | 1.000 | 1 | 1273 |
| REACTOME_REGULATION_OF_GLUCOKINASE_BY_GLUCOKINASE_REGULATORY_PROTEIN | 30 | 0.224 | 0.674 | 0.933 | 1.000 | 1 | 2107 |
| REACTOME_DISEASES_ASSOCIATED_WITH_GLYCOSYLATION_PRECURSOR_BIOSYNTHESIS | 18 | 0.245 | 0.667 | 0.906 | 1.000 | 1 | 2649 |
| REACTOME_CROSS_PRESENTATION_OF_SOLUBLE_EXOGENOUS_ANTIGENS_ENDOSOMES_ | 45 | 0.202 | 0.667 | 0.952 | 1.000 | 1 | 45 |
| REACTOME_CREB3_FACTORS_ACTIVATE_GENES | 8 | 0.310 | 0.666 | 0.865 | 1.000 | 1 | 1248 |
| REACTOME_PRC2_METHYLATES_HISTONES_AND_DNA | 23 | 0.234 | 0.665 | 0.924 | 1.000 | 1 | 85 |
| REACTOME_RELEASE_OF_APOPTOTIC_FACTORS_FROM_THE_MITOCHONDRIA | 5 | 0.352 | 0.663 | 0.844 | 1.000 | 1 | 8232 |
| REACTOME_MTOR_SIGNALLING | 39 | 0.206 | 0.662 | 0.947 | 1.000 | 1 | 3484 |
| REACTOME_VITAMIN_D_CALCIFEROL_METABOLISM | 8 | 0.304 | 0.661 | 0.880 | 1.000 | 1 | 3128 |
| REACTOME_FATTY_ACYL_COA_BIOSYNTHESIS | 26 | 0.221 | 0.658 | 0.928 | 1.000 | 1 | 2450 |
| REACTOME_REGULATION_OF_HSF1_MEDIATED_HEAT_SHOCK_RESPONSE | 76 | 0.181 | 0.658 | 0.985 | 1.000 | 1 | 3995 |
| REACTOME_NUCLEAR_ENVELOPE_BREAKDOWN | 52 | 0.195 | 0.656 | 0.969 | 1.000 | 1 | 4409 |
| REACTOME_APOPTOTIC_FACTOR_MEDIATED_RESPONSE | 18 | 0.240 | 0.655 | 0.927 | 1.000 | 1 | 2887 |
| REACTOME_REGULATION_OF_GENE_EXPRESSION_IN_LATE_STAGE_BRANCHING_MORPHOGENESIS_PANCREATIC_BUD_PRECURSOR_CELLS | 12 | 0.264 | 0.651 | 0.919 | 1.000 | 1 | 4374 |
| REACTOME_ATF6_ATF6_ALPHA_ACTIVATES_CHAPERONES | 12 | 0.269 | 0.651 | 0.897 | 1.000 | 1 | 4215 |
| REACTOME_TYSND1_CLEAVES_PEROXISOMAL_PROTEINS | 7 | 0.310 | 0.649 | 0.871 | 1.000 | 1 | 8768 |
| REACTOME_TRAFFICKING_OF_MYRISTOYLATED_PROTEINS_TO_THE_CILIUM | 4 | 0.367 | 0.646 | 0.879 | 1.000 | 1 | 3116 |

| | | | | | | | |
|---|---|---|---|---|---|---|---|
| REACTOME_C_TYPE_LECTIN_RECEPTORS_CLRS_ | 112 | 0.167 | 0.643 | 0.994 | 1.000 | 1 | 4340 |
| REACTOME_DEX_H_BOX_HELICASES_ACTIVATE_TYPE_I_IFN_AND_INFLAMMATORY_CYTOKINES_PRODUCTION_ | 7 | 0.310 | 0.640 | 0.878 | 1.000 | 1 | 5070 |
| REACTOME_INITIATION_OF_NUCLEAR_ENVELOPE_NE_REFORMATION | 19 | 0.234 | 0.635 | 0.927 | 1.000 | 1 | 9743 |
| REACTOME_PROCESSING_OF_CAPPED_INTRON_CONTAINING_PRE_MRNA | 236 | 0.149 | 0.634 | 1.000 | 1.000 | 1 | 4177 |
| REACTOME_PHOSPHORYLATION_OF_THE_APC_C | 20 | 0.226 | 0.631 | 0.941 | 1.000 | 1 | 5013 |
| REACTOME_CONSTITUTIVE_SIGNALING_BY_EGFRVIII | 12 | 0.252 | 0.625 | 0.910 | 1.000 | 1 | 4118 |
| REACTOME_APC_C_CDC20_MEDIATED_DEGRADATION_OF_CYCLIN_B | 24 | 0.218 | 0.623 | 0.951 | 1.000 | 1 | 5013 |
| REACTOME_TRANSCRIPTIONAL_REGULATION_BY_SMALL_RNAS | 57 | 0.180 | 0.622 | 0.984 | 1.000 | 1 | 2107 |
| REACTOME_TRANSCRIPTIONAL_REGULATION_BY_VENTX | 36 | 0.197 | 0.619 | 0.976 | 1.000 | 1 | 1433 |
| REACTOME_LDL_CLEARANCE | 18 | 0.230 | 0.619 | 0.948 | 1.000 | 1 | 1372 |
| REACTOME_DEFENSINS | 3 | 0.381 | 0.618 | 0.911 | 1.000 | 1 | 1251 |
| REACTOME_ALPHA_DEFENSINS | 3 | 0.381 | 0.617 | 0.909 | 1.000 | 1 | 1251 |
| REACTOME_EUKARYOTIC_TRANSLATION_INITIATION | 113 | 0.158 | 0.615 | 0.999 | 1.000 | 1 | 5937 |
| REACTOME_ARACHIDONATE_PRODUCTION_FROM_DAG | 4 | 0.351 | 0.615 | 0.887 | 1.000 | 1 | 4067 |
| REACTOME_MRNA_SPLICING | 186 | 0.149 | 0.612 | 1.000 | 1.000 | 1 | 4161 |
| REACTOME_AQUAPORIN_MEDIATED_TRANSPORT | 24 | 0.212 | 0.612 | 0.963 | 1.000 | 1 | 2618 |
| REACTOME_NONSENSE_MEDIATED_DECAY_NMD_ | 110 | 0.161 | 0.610 | 0.997 | 1.000 | 1 | 6007 |
| REACTOME_MITOTIC_TELOPHASE_CYTOKINESIS | 13 | 0.245 | 0.609 | 0.927 | 1.000 | 1 | 9605 |
| REACTOME_BETA_OXIDATION_OF_HEXANOYL_COA_TO_BUTANOYL_COA | 5 | 0.319 | 0.608 | 0.909 | 1.000 | 1 | 8660 |
| REACTOME_NUCLEAR_IMPORT_OF_REV_PROTEIN | 34 | 0.192 | 0.608 | 0.973 | 1.000 | 1 | 2107 |
| REACTOME_ALPHA_LINOLENIC_OMEGA3_AND_LINOLEIC_OMEGA6_ACID_METABOLISM | 11 | 0.252 | 0.606 | 0.925 | 1.000 | 1 | 4978 |
| REACTOME_MYOCLONIC_EPILEPSY_OF_LAFORA | 7 | 0.287 | 0.604 | 0.922 | 1.000 | 1 | 3842 |
| REACTOME_DEACTIVATION_OF_THE_BETA_CATENIN_TRANSACTIVATING_COMPLEX | 35 | 0.192 | 0.603 | 0.974 | 1.000 | 1 | 3842 |

| | | | | | | | |
|---|---|---|---|---|---|---|---|
| REACTOME_TP53_REGULATES_TRANSCRIPTION_OF_DNA_REPAIR_GENES | 60 | 0.171 | 0.603 | 0.995 | 1.000 | 1 | 3815 |
| REACTOME_RNA_POLYMERASE_II_TRANSCRIPTION_TERMINATION | 63 | 0.171 | 0.599 | 0.997 | 1.000 | 1 | 4271 |
| REACTOME_LINOLEIC_ACID_LA_METABOLISM | 6 | 0.302 | 0.598 | 0.918 | 1.000 | 1 | 4292 |
| REACTOME_PROTEIN_METHYLATION | 16 | 0.228 | 0.593 | 0.958 | 1.000 | 1 | 4224 |
| REACTOME_POSTMITOTIC_NUCLEAR_PORE_COMPLEX_NPC_REFORMATION | 27 | 0.200 | 0.591 | 0.964 | 1.000 | 1 | 2107 |
| REACTOME_CD28_DEPENDENT_PI3K_AKT_SIGNALING | 19 | 0.212 | 0.589 | 0.969 | 1.000 | 1 | 3080 |
| REACTOME_ANTIGEN_PROCESSING_UBIQUITINATION_PROTEASOME_DEGRADATION | 272 | 0.136 | 0.587 | 1.000 | 1.000 | 1 | 4026 |
| REACTOME_RNA_POLYMERASE_II_TRANSCRIBES_SNRNA_GENES | 74 | 0.162 | 0.584 | 0.995 | 1.000 | 1 | 3815 |
| REACTOME_INTERLEUKIN_1_FAMILY_SIGNALING | 108 | 0.151 | 0.584 | 1.000 | 1.000 | 1 | 3449 |
| REACTOME_MRNA_SPLICING_MINOR_PATHWAY | 52 | 0.172 | 0.581 | 0.984 | 1.000 | 1 | 2715 |
| REACTOME_DOWNSTREAM_SIGNALING_EVENTS_OF_B_CELL_RECEPTOR_BCR_ | 76 | 0.161 | 0.578 | 0.998 | 1.000 | 1 | 4991 |
| REACTOME_PKA_MEDIATED_PHOSPHORYLATION_OF_CREB | 13 | 0.231 | 0.573 | 0.954 | 1.000 | 1 | 779 |
| REACTOME_MITOTIC_METAPHASE_AND_ANAPHASE | 224 | 0.134 | 0.567 | 1.000 | 1.000 | 1 | 2240 |
| REACTOME_CLEC7A_DECTIN_1_SIGNALING | 94 | 0.151 | 0.566 | 0.998 | 1.000 | 1 | 4335 |
| REACTOME_INHIBITION_OF_THE_PROTEOLYTIC_ACTIVITY_OF_APC_C_REQUIRED_FOR_THE_ONSET_OF_ANAPHASE_BY_MITOTIC_SPINDLE_CHECKPOINT_COMPONENTS | 21 | 0.200 | 0.564 | 0.977 | 1.000 | 1 | 10167 |
| REACTOME_CONVERSION_FROM_APC_C_CDC20_TO_APC_C_CDH1_IN_LATE_ANAPHASE | 20 | 0.202 | 0.554 | 0.986 | 1.000 | 1 | 3783 |
| REACTOME_REGULATION_OF_PTEN_LOCALIZATION | 9 | 0.244 | 0.549 | 0.951 | 1.000 | 1 | 9613 |
| REACTOME_ERYTHROCYTES_TAKE_UP_OXYGEN_AND_RELEASE_CARBON_DIOXIDE | 2 | 0.362 | 0.549 | 0.959 | 1.000 | 1 | 2318 |
| REACTOME_MECP2_REGULATES_TRANSCRIPTION_OF_NEURONAL_LIGANDS | 5 | 0.288 | 0.546 | 0.960 | 1.000 | 1 | 9046 |
| REACTOME_SEPARATION_OF_SISTER_CHROMATIDS | 179 | 0.133 | 0.544 | 1.000 | 1.000 | 1 | 2240 |

| | | | | | | | |
|---|---|---|---|---|---|---|---|
| REACTOME_CHROMATIN_MODIFYING_ENZYMES | 208 | 0.129 | 0.542 | 1.000 | 1.000 | 1 | 3168 |
| REACTOME_REGULATION_OF_MECP2_EXPRESSION_AND_ACTIVITY | 29 | 0.184 | 0.541 | 0.993 | 1.000 | 1 | 1061 |
| REACTOME_B_WICH_COMPLEX_POSITIVELY_REGULATES_RRNA_EXPRESSION | 41 | 0.168 | 0.539 | 0.993 | 1.000 | 1 | 3793 |
| REACTOME_NEF_MEDIATED_CD8_DOWN_REGULATION | 7 | 0.258 | 0.539 | 0.960 | 1.000 | 1 | 2755 |
| REACTOME_DOWNREGULATION_OF_ERBB4_SIGNALING | 8 | 0.248 | 0.536 | 0.956 | 1.000 | 1 | 3842 |
| REACTOME_BETA_OXIDATION_OF_BUTANOYL_COA_TO_ACETYL_COA | 3 | 0.319 | 0.531 | 0.948 | 1.000 | 1 | 8660 |
| REACTOME_PKA_ACTIVATION_IN_GLUCAGON_SIGNALLING | 10 | 0.223 | 0.520 | 0.979 | 1.000 | 1 | 2328 |
| REACTOME_ARMS_MEDIATED_ACTIVATION | 5 | 0.268 | 0.519 | 0.964 | 1.000 | 1 | 3484 |
| REACTOME_HCMV_LATE_EVENTS | 60 | 0.148 | 0.516 | 1.000 | 1.000 | 1 | 4377 |
| REACTOME_ACYL_CHAIN_REMODELING_OF_CL | 5 | 0.269 | 0.511 | 0.970 | 1.000 | 1 | 1237 |
| REACTOME_INHIBITION_OF_DNA_RECOMBINATION_AT_TELOMERE | 30 | 0.166 | 0.510 | 1.000 | 1.000 | 1 | 2835 |
| REACTOME_TIE2_SIGNALING | 13 | 0.209 | 0.507 | 0.978 | 1.000 | 1 | 4442 |
| REACTOME_INTERACTIONS_OF_REV_WITH_HOST_CELLULAR_PROTEINS | 37 | 0.159 | 0.503 | 0.998 | 1.000 | 1 | 2107 |
| REACTOME_SIGNALING_BY_ERBB2_ECD_MUTANTS | 13 | 0.201 | 0.502 | 0.987 | 1.000 | 1 | 4118 |
| REACTOME_SNRNP_ASSEMBLY | 52 | 0.147 | 0.500 | 0.998 | 1.000 | 1 | 4377 |
| REACTOME_DEUBIQUITINATION | 226 | 0.117 | 0.498 | 1.000 | 1.000 | 1 | 3181 |
| REACTOME_ANDROGEN_BIOSYNTHESIS | 4 | 0.280 | 0.494 | 0.976 | 1.000 | 1 | 3910 |
| REACTOME_NR1H2_NR1H3_REGULATE_GENE_EXPRESSION_TO_CONTROL_BILE_ACID_HOMEOSTASIS | 7 | 0.245 | 0.493 | 0.973 | 1.000 | 1 | 5158 |
| REACTOME_CITRIC_ACID_CYCLE_TCA_CYCLE_ | 22 | 0.176 | 0.489 | 0.991 | 1.000 | 1 | 2082 |
| REACTOME_ACTIVATION_OF_ANTERIOR_HOX_GENES_IN_HINDBRAIN_DEVELOPMENT_DURING_EARLY_EMBRYOGENESIS | 66 | 0.139 | 0.486 | 1.000 | 1.000 | 1 | 3821 |
| REACTOME_ATF6_ATF6_ALPHA_ACTIVATES_CHAPERONE_GENES | 10 | 0.213 | 0.485 | 0.981 | 1.000 | 1 | 4215 |
| REACTOME_APC_CDC20_MEDIATED_DEGRADATION_OF_NEK2A | 26 | 0.165 | 0.484 | 0.995 | 1.000 | 1 | 3892 |

| | | | | | | | |
|---|---|---|---|---|---|---|---|
| REACTOME_SYNTHESIS_OF_PIPS_AT_THE_EARLY_ENDOSOME_MEMBRANE | 15 | 0.187 | 0.482 | 0.984 | 1.000 | 1 | 2243 |
| REACTOME_RNA_POLYMERASE_I_TRANSCRIPTION | 60 | 0.137 | 0.479 | 1.000 | 1.000 | 1 | 3522 |
| REACTOME_ACTIVATION_OF_RAC1_DOWNSTREAM_OF_NMDARS | 7 | 0.232 | 0.476 | 0.996 | 1.000 | 1 | 4281 |
| REACTOME_ABERRANT_REGULATION_OF_MITOTIC_EXIT_IN_CANCER_DUE_TO_RB1_DEFECTS | 20 | 0.169 | 0.469 | 0.996 | 1.000 | 1 | 3783 |
| REACTOME_REGULATION_OF_TP53_ACTIVITY_THROUGH_METHYLATION | 19 | 0.170 | 0.461 | 0.993 | 1.000 | 1 | 3170 |
| REACTOME_KSRP_KHSRP_BINDS_AND_DESTABILIZES_MRNA | 17 | 0.171 | 0.455 | 0.997 | 1.000 | 1 | 3199 |
| REACTOME_TRNA_PROCESSING_IN_THE_NUCLEUS | 58 | 0.129 | 0.448 | 1.000 | 1.000 | 1 | 4682 |
| REACTOME_NEGATIVE_EPIGENETIC_REGULATION_OF_RRNA_EXPRESSION | 58 | 0.129 | 0.445 | 1.000 | 1.000 | 1 | 11074 |
| REACTOME_RETROGRADE_NEUROTROPHIN_SIGNALLING | 11 | 0.191 | 0.445 | 0.995 | 1.000 | 1 | 10291 |
| REACTOME_NA_CL_DEPENDENT_NEUROTRANSMITTER_TRANSPORTERS | 2 | 0.296 | 0.445 | 0.994 | 1.000 | 1 | 3207 |
| REACTOME_NTRK2_ACTIVATES_RAC1 | 3 | 0.275 | 0.441 | 0.996 | 1.000 | 1 | 2419 |
| REACTOME_RECYCLING_OF_EIF2_GDP | 8 | 0.201 | 0.435 | 0.993 | 1.000 | 1 | 1783 |
| REACTOME_TP53_REGULATES_TRANSCRIPTION_OF_GENES_INVOLVED_IN_G2_CELL_CYCLE_ARREST | 18 | 0.162 | 0.434 | 0.996 | 1.000 | 1 | 2407 |
| REACTOME_SENSING_OF_DNA_DOUBLE_STRAND_BREAKS | 6 | 0.215 | 0.430 | 0.994 | 1.000 | 1 | 4604 |
| REACTOME_P2Y_RECEPTORS | 3 | 0.256 | 0.426 | 1.000 | 1.000 | 1 | 4466 |
| REACTOME_RNA_POLYMERASE_I_PROMOTER_ESCAPE | 40 | 0.129 | 0.414 | 1.000 | 1.000 | 1 | 11074 |
| REACTOME_MITOCHONDRIAL_FATTY_ACID_BETA_OXIDATION_OF_SATURATED_FATTY_ACIDS | 8 | 0.187 | 0.412 | 0.998 | 1.000 | 1 | 4992 |
| REACTOME_REGULATION_OF_RUNX3_EXPRESSION_AND_ACTIVITY | 55 | 0.115 | 0.401 | 1.000 | 1.000 | 1 | 184 |
| REACTOME_RESPONSE_OF_EIF2AK4_GCN2_TO_AMINO_ACID_DEFICIENCY | 97 | 0.092 | 0.352 | 1.000 | 1.000 | 1 | 5937 |
| REACTOME_RETROGRADE_TRANSPORT_AT_THE_TRANS_GOLGI_NETWORK | 47 | 0.103 | 0.348 | 1.000 | 1.000 | 1 | 1985 |
| REACTOME_FCERI_MEDIATED_NF_KB_ACTIVATION | 73 | 0.097 | 0.347 | 1.000 | 1.000 | 1 | 4991 |

| HS_siLonp vs siScramble | | | | | | | |
|---|---|---|---|---|---|---|---|
| NAME | SIZE | ES | NES | NOM p-val | FDR q-val | FWER p-val | RANK AT MAX |
| REACTOME_NUCLEAR_EVENTS_KINASE_AND_TRANSCRIPTION_FACTOR_ACTIVATION_ | 54 | 0.649 | 2.320 | 0.000 | 0.000 | 0.000 | 2088 |
| REACTOME_NGF_STIMULATED_TRANSCRIPTION | 33 | 0.721 | 2.298 | 0.000 | 0.000 | 0.000 | 1589 |
| REACTOME_SRP_DEPENDENT_COTRANSLATIONAL_PROTEIN_TARGETING_TO_MEMBRANE | 108 | 0.552 | 2.250 | 0.000 | 0.000 | 0.000 | 4689 |
| REACTOME_EUKARYOTIC_TRANSLATION_ELONGATION | 89 | 0.568 | 2.201 | 0.000 | 0.000 | 0.000 | 4689 |
| REACTOME_KERATINIZATION | 38 | 0.655 | 2.195 | 0.000 | 0.000 | 0.000 | 1499 |
| REACTOME_ELASTIC_FIBRE_FORMATION | 25 | 0.722 | 2.192 | 0.000 | 0.000 | 0.000 | 1821 |
| REACTOME_EXTRACELLULAR_MATRIX_ORGANIZATION | 164 | 0.511 | 2.188 | 0.000 | 0.000 | 0.000 | 1289 |
| REACTOME_ASSEMBLY_AND_CELL_SURFACE_PRESENTATION_OF_NMDA_RECEPTORS | 24 | 0.696 | 2.109 | 0.000 | 0.002 | 0.010 | 2861 |
| REACTOME_FORMATION_OF_THE_CORNIFIED_ENVELOPE | 34 | 0.657 | 2.108 | 0.000 | 0.001 | 0.010 | 1741 |
| REACTOME_RESPONSE_OF_EIF2AK4_GCN2_TO_AMINO_ACID_DEFICIENCY | 97 | 0.524 | 2.094 | 0.000 | 0.001 | 0.010 | 4689 |
| REACTOME_FORMATION_OF_TUBULIN_FOLDING_INTERMEDIATES_BY_CCT_TRIC | 19 | 0.745 | 2.088 | 0.000 | 0.001 | 0.013 | 1710 |
| REACTOME_MOLECULES_ASSOCIATED_WITH_ELASTIC_FIBRES | 19 | 0.734 | 2.072 | 0.000 | 0.002 | 0.015 | 1821 |
| REACTOME_INTEGRIN_CELL_SURFACE_INTERACTIONS | 45 | 0.578 | 2.024 | 0.000 | 0.004 | 0.043 | 1119 |
| REACTOME_A_TETRASACCHARIDE_LINKER_SEQUENCE_IS_REQUIRED_FOR_GAG_SYNTHESIS | 15 | 0.772 | 2.012 | 0.000 | 0.005 | 0.058 | 2177 |
| REACTOME_DEGRADATION_OF_THE_EXTRACELLULAR_MATRIX | 70 | 0.541 | 2.011 | 0.000 | 0.005 | 0.060 | 1119 |
| REACTOME_RESPONSE_TO_METAL_IONS | 10 | 0.829 | 2.010 | 0.000 | 0.005 | 0.061 | 1424 |
| REACTOME_EUKARYOTIC_TRANSLATION_INITIATION | 113 | 0.486 | 1.986 | 0.000 | 0.006 | 0.087 | 4732 |
| REACTOME_METALLOTHIONEINS_BIND_METALS | 8 | 0.874 | 1.983 | 0.000 | 0.007 | 0.092 | 1066 |
| REACTOME_CHONDROITIN_SULFATE_DERMATAN_SULFATE_METABOLISM | 36 | 0.598 | 1.977 | 0.000 | 0.007 | 0.103 | 1682 |
| REACTOME_ASSEMBLY_OF_COLLAGEN_FIBRILS_AND_OTHER_MULTIMERIC_STRUCTURES | 32 | 0.615 | 1.974 | 0.000 | 0.007 | 0.108 | 1289 |

| | | | | | | | |
|---|---|---|---|---|---|---|---|
| REACTOME_DEFECTIVE_B4GALT7_CAUSES_EDS_PROGEROID_TYPE | 11 | 0.804 | 1.971 | 0.000 | 0.007 | 0.110 | 2177 |
| REACTOME_SYNDECAN_INTERACTIONS | 18 | 0.710 | 1.971 | 0.002 | 0.007 | 0.112 | 2065 |
| REACTOME_SIGNALING_BY_NTRKS | 113 | 0.484 | 1.966 | 0.000 | 0.007 | 0.122 | 2088 |
| REACTOME_DISSOLUTION_OF_FIBRIN_CLOT | 10 | 0.819 | 1.962 | 0.000 | 0.007 | 0.134 | 2250 |
| REACTOME_RESPONSE_TO_ELEVATED_PLATELET_CYTOSOLIC_CA2_ | 85 | 0.499 | 1.939 | 0.000 | 0.009 | 0.177 | 2642 |
| REACTOME_COOPERATION_OF_PREFOLDIN_AND_TRIC_CCT_IN_ACTIN_AND_TUBULIN_FOLDING | 26 | 0.633 | 1.934 | 0.000 | 0.010 | 0.190 | 1710 |
| REACTOME_CONSTITUTIVE_SIGNALING_BY_ABERRANT_PI3K_IN_CANCER | 39 | 0.579 | 1.934 | 0.000 | 0.009 | 0.190 | 1771 |
| REACTOME_CELL_EXTRACELLULAR_MATRIX_INTERACTIONS | 17 | 0.703 | 1.924 | 0.000 | 0.011 | 0.223 | 2504 |
| REACTOME_NONSENSE_MEDIATED_DECAY_NMD_ | 110 | 0.481 | 1.920 | 0.000 | 0.011 | 0.236 | 4689 |
| REACTOME_COLLAGEN_CHAIN_TRIMERIZATION | 15 | 0.712 | 1.918 | 0.000 | 0.011 | 0.240 | 1119 |
| REACTOME_ECM_PROTEOGLYCANS | 37 | 0.577 | 1.901 | 0.000 | 0.014 | 0.306 | 2186 |
| REACTOME_ROS_AND_RNS_PRODUCTION_IN_PHAGOCYTES | 21 | 0.641 | 1.898 | 0.002 | 0.014 | 0.312 | 3485 |
| REACTOME_COLLAGEN_FORMATION | 50 | 0.528 | 1.884 | 0.000 | 0.017 | 0.362 | 1289 |
| REACTOME_COLLAGEN_DEGRADATION | 29 | 0.595 | 1.881 | 0.000 | 0.017 | 0.379 | 708 |
| REACTOME_SHC1_EVENTS_IN_EGFR_SIGNALING | 10 | 0.793 | 1.880 | 0.002 | 0.017 | 0.383 | 922 |
| REACTOME_NEUREXINS_AND_NEUROLIGINS | 34 | 0.582 | 1.879 | 0.000 | 0.016 | 0.387 | 2063 |
| REACTOME_CHEMOKINE_RECEPTORS_BIND_CHEMOKINES | 10 | 0.792 | 1.875 | 0.000 | 0.017 | 0.408 | 1549 |
| REACTOME_PLASMA_LIPOPROTEIN_ASSEMBLY | 9 | 0.804 | 1.871 | 0.002 | 0.018 | 0.427 | 406 |
| REACTOME_RECYCLING_PATHWAY_OF_L1 | 39 | 0.560 | 1.867 | 0.002 | 0.018 | 0.441 | 1810 |
| REACTOME_NON_INTEGRIN_MEMBRANE_ECM_INTERACTIONS | 37 | 0.559 | 1.858 | 0.000 | 0.020 | 0.479 | 2264 |
| REACTOME_ACTIVATION_OF_AMPK_DOWNSTREAM_OF_NMDARS | 20 | 0.650 | 1.856 | 0.002 | 0.020 | 0.492 | 1710 |
| REACTOME_HEPARAN_SULFATE_HEPARIN_HS_GAG_METABOLISM | 34 | 0.564 | 1.837 | 0.004 | 0.027 | 0.597 | 2418 |
| REACTOME_SIGNALING_BY_RECEPTOR_TYROSINE_KINASES | 377 | 0.387 | 1.813 | 0.000 | 0.036 | 0.719 | 2147 |

| | | | | | | | |
|---|---|---|---|---|---|---|---|
| REACTOME_GAP_JUNCTION_ASSEMBLY | 18 | 0.647 | 1.808 | 0.000 | 0.037 | 0.739 | 1710 |
| REACTOME_REGULATION_OF_EXPRESSION_OF_SLITS_AND_ROBOS | 156 | 0.424 | 1.807 | 0.000 | 0.037 | 0.740 | 4025 |
| REACTOME_COLLAGEN_BIOSYNTHESIS_AND_MODIFYING_ENZYMES | 33 | 0.557 | 1.807 | 0.004 | 0.036 | 0.741 | 1728 |
| REACTOME_UPTAKE_AND_ACTIONS_OF_BACTERIAL_TOXINS | 23 | 0.594 | 1.806 | 0.002 | 0.036 | 0.750 | 2848 |
| REACTOME_NEGATIVE_REGULATION_OF_MAPK_PATHWAY | 42 | 0.527 | 1.796 | 0.000 | 0.039 | 0.789 | 4650 |
| REACTOME_RHO_GTPASES_ACTIVATE_IQGAPS | 23 | 0.598 | 1.795 | 0.000 | 0.039 | 0.789 | 1751 |
| REACTOME_TOLL_LIKE_RECEPTOR_TLR1_TLR2_CASCADE | 82 | 0.456 | 1.790 | 0.000 | 0.041 | 0.815 | 2936 |
| REACTOME_SELENOAMINO_ACID_METABOLISM | 107 | 0.445 | 1.785 | 0.002 | 0.043 | 0.832 | 4689 |
| REACTOME_SEALING_OF_THE_NUCLEAR_ENVELOPE_NE_BY_ESCRT_III | 24 | 0.603 | 1.782 | 0.004 | 0.044 | 0.844 | 3118 |
| REACTOME_ACTIVATION_OF_THE_MRNA_UPON_BINDING_OF_THE_CAP_BINDING_COMPLEX_AND_EIFS_AND_SUBSEQUENT_BINDING_TO_43S | 56 | 0.483 | 1.756 | 0.002 | 0.058 | 0.908 | 4732 |
| REACTOME_NEGATIVE_REGULATION_OF_FGFR2_SIGNALING | 20 | 0.613 | 1.753 | 0.007 | 0.059 | 0.914 | 2088 |
| REACTOME_GAP_JUNCTION_TRAFFICKING_AND_REGULATION | 31 | 0.555 | 1.753 | 0.002 | 0.058 | 0.915 | 1857 |
| REACTOME_NEGATIVE_REGULATION_OF_FGFR4_SIGNALING | 21 | 0.604 | 1.750 | 0.004 | 0.059 | 0.923 | 2088 |
| REACTOME_GRB2_SOS_PROVIDES_LINKAGE_TO_MAPK_SIGNALING_FOR_INTEGRINS_ | 11 | 0.692 | 1.748 | 0.013 | 0.059 | 0.926 | 897 |
| REACTOME_RESPONSE_OF_EIF2AK1_HRI_TO_HEME_DEFICIENCY | 15 | 0.655 | 1.748 | 0.011 | 0.058 | 0.926 | 2415 |
| REACTOME_PI3K_AKT_SIGNALING_IN_CANCER | 66 | 0.473 | 1.747 | 0.002 | 0.058 | 0.931 | 1771 |
| REACTOME_DEFECTIVE_EXT2_CAUSES_EXOSTOSES_2 | 10 | 0.748 | 1.745 | 0.007 | 0.059 | 0.935 | 2212 |
| REACTOME_TRANSPORT_OF_CONNEXONS_TO_THE_PLASMA_MEMBRANE | 12 | 0.707 | 1.744 | 0.002 | 0.058 | 0.937 | 1710 |
| REACTOME_SIGNALING_BY_ROBO_RECEPTORS | 191 | 0.400 | 1.743 | 0.000 | 0.058 | 0.940 | 4025 |
| REACTOME_HSP90_CHAPERONE_CYCLE_FOR_STEROID_HORMONE_RECEPTORS_SHR_ | 44 | 0.507 | 1.741 | 0.005 | 0.059 | 0.944 | 2078 |
| REACTOME_ACTIVATION_OF_NMDA_RECEPTORS_AND_POSTSYNAPTIC_EVENTS | 63 | 0.459 | 1.723 | 0.002 | 0.070 | 0.973 | 2486 |

| | | | | | | | |
|---|---|---|---|---|---|---|---|
| REACTOME_NEGATIVE_REGULATION_OF_THE_PI3K_AKT_NETWORK | 70 | 0.464 | 1.722 | 0.000 | 0.070 | 0.975 | 2759 |
| REACTOME_ACETYLCHOLINE_BINDING_AND_DOWNSTREAM_EVENTS | 5 | 0.888 | 1.721 | 0.009 | 0.070 | 0.977 | 123 |
| REACTOME_NEGATIVE_REGULATION_OF_FGFR3_SIGNALING | 20 | 0.608 | 1.718 | 0.009 | 0.071 | 0.981 | 2088 |
| REACTOME_POST_TRANSLATIONAL_MODIFICATION_SYNTHESIS_OF_GPI_ANCHORED_PROTEINS | 40 | 0.509 | 1.713 | 0.007 | 0.075 | 0.984 | 1207 |
| REACTOME_COPI_INDEPENDENT_GOLGI_TO_ER_RETROGRADE_TRAFFIC | 41 | 0.506 | 1.710 | 0.005 | 0.076 | 0.986 | 2360 |
| REACTOME_FLT3_SIGNALING | 217 | 0.385 | 1.707 | 0.000 | 0.077 | 0.989 | 3989 |
| REACTOME_INTERLEUKIN_6_FAMILY_SIGNALING | 14 | 0.646 | 1.707 | 0.011 | 0.075 | 0.989 | 3707 |
| REACTOME_CTLA4_INHIBITORY_SIGNALING | 18 | 0.609 | 1.704 | 0.011 | 0.077 | 0.990 | 2441 |
| REACTOME_NERVOUS_SYSTEM_DEVELOPMENT | 448 | 0.359 | 1.704 | 0.000 | 0.076 | 0.990 | 4044 |
| REACTOME_CELL_SURFACE_INTERACTIONS_AT_THE_VASCULAR_WALL | 75 | 0.445 | 1.703 | 0.000 | 0.076 | 0.990 | 1657 |
| REACTOME_P75NTR_NEGATIVELY_REGULATES_CELL_CYCLE_VIA_SC1 | 5 | 0.859 | 1.697 | 0.004 | 0.079 | 0.992 | 6 |
| REACTOME_CARGO_CONCENTRATION_IN_THE_ER | 31 | 0.531 | 1.696 | 0.009 | 0.079 | 0.992 | 2582 |
| REACTOME_PLATELET_ACTIVATION_SIGNALING_AND_AGGREGATION | 167 | 0.400 | 1.695 | 0.000 | 0.079 | 0.992 | 2675 |
| REACTOME_INSULIN_RECEPTOR_RECYCLING | 21 | 0.589 | 1.695 | 0.009 | 0.078 | 0.992 | 3485 |
| REACTOME_MAPK_FAMILY_SIGNALING_CASCADES | 245 | 0.379 | 1.693 | 0.000 | 0.078 | 0.992 | 3989 |
| REACTOME_SIGNALING_BY_EGFR_IN_CANCER | 20 | 0.584 | 1.692 | 0.018 | 0.078 | 0.992 | 2078 |
| REACTOME_HEMOSTASIS | 385 | 0.359 | 1.690 | 0.000 | 0.079 | 0.993 | 2383 |
| REACTOME_NEUTROPHIL_DEGRANULATION | 342 | 0.359 | 1.687 | 0.000 | 0.081 | 0.994 | 2892 |
| REACTOME_VOLTAGE_GATED_POTASSIUM_CHANNELS | 12 | 0.654 | 1.682 | 0.019 | 0.083 | 0.995 | 513 |
| REACTOME_NEURONAL_SYSTEM | 196 | 0.387 | 1.680 | 0.000 | 0.084 | 0.996 | 2209 |
| REACTOME_ESTROGEN_DEPENDENT_NUCLEAR_EVENTS_DOWNSTREAM_OF_ESR_MEMBRANE_SIGNALING | 20 | 0.584 | 1.678 | 0.014 | 0.085 | 0.996 | 2026 |
| REACTOME_MAPK_TARGETS_NUCLEAR_EVENTS_MEDIATED_BY_MAP_KINASES | 29 | 0.533 | 1.676 | 0.005 | 0.086 | 0.996 | 2088 |

| | | | | | | | |
|---|---|---|---|---|---|---|---|
| REACTOME_DERMATAN_SULFATE_BIOSYNTHESIS | 5 | 0.861 | 1.670 | 0.002 | 0.090 | 0.997 | 850 |
| REACTOME_ATTENUATION_PHASE | 23 | 0.566 | 1.669 | 0.013 | 0.089 | 0.997 | 4503 |
| REACTOME_TOLL_LIKE_RECEPTOR_4_TLR4_CASCADE | 106 | 0.410 | 1.669 | 0.000 | 0.088 | 0.997 | 2936 |
| REACTOME_RHO_GTPASES_ACTIVATE_CIT | 18 | 0.586 | 1.669 | 0.007 | 0.088 | 0.999 | 3725 |
| REACTOME_PEPTIDE_LIGAND_BINDING_RECEPTORS | 37 | 0.502 | 1.668 | 0.012 | 0.088 | 0.999 | 1549 |
| REACTOME_CHONDROITIN_SULFATE_BIOSYNTHESIS | 13 | 0.664 | 1.668 | 0.011 | 0.087 | 0.999 | 1010 |
| REACTOME_BASIGIN_INTERACTIONS | 19 | 0.588 | 1.665 | 0.013 | 0.089 | 0.999 | 4186 |
| REACTOME_SIGNAL_ATTENUATION | 9 | 0.717 | 1.664 | 0.010 | 0.089 | 0.999 | 1544 |
| REACTOME_YAP1_AND_WWTR1_TAZ_STIMULATED_GENE_EXPRESSION | 12 | 0.671 | 1.663 | 0.013 | 0.089 | 0.999 | 2187 |
| REACTOME_CHYLOMICRON_ASSEMBLY | 3 | 0.979 | 1.662 | 0.000 | 0.089 | 0.999 | 191 |
| REACTOME_MUSCLE_CONTRACTION | 99 | 0.421 | 1.661 | 0.003 | 0.089 | 0.999 | 2515 |
| REACTOME_LGI_ADAM_INTERACTIONS | 6 | 0.822 | 1.660 | 0.010 | 0.089 | 0.999 | 1110 |
| REACTOME_ER_TO_GOLGI_ANTEROGRADE_TRANSPORT | 139 | 0.396 | 1.660 | 0.002 | 0.088 | 0.999 | 2468 |
| REACTOME_NCAM_SIGNALING_FOR_NEURITE_OUT_GROWTH | 34 | 0.507 | 1.658 | 0.014 | 0.088 | 0.999 | 579 |
| REACTOME_LOSS_OF_FUNCTION_OF_SMAD2_3_IN_CANCER | 7 | 0.765 | 1.657 | 0.011 | 0.089 | 0.999 | 2988 |
| REACTOME_TYPE_I_HEMIDESMOSOME_ASSEMBLY | 9 | 0.715 | 1.656 | 0.017 | 0.089 | 0.999 | 2065 |
| REACTOME_PROTEIN_PROTEIN_INTERACTIONS_AT_SYNAPSES | 50 | 0.468 | 1.653 | 0.005 | 0.090 | 1.000 | 2063 |
| REACTOME_RAB_GERANYLGERANYLATION | 48 | 0.470 | 1.653 | 0.003 | 0.090 | 1.000 | 4020 |
| REACTOME_SIGNALING_BY_INTERLEUKINS | 302 | 0.360 | 1.648 | 0.000 | 0.093 | 1.000 | 3243 |
| REACTOME_GPCR_LIGAND_BINDING | 106 | 0.410 | 1.648 | 0.000 | 0.093 | 1.000 | 1689 |
| REACTOME_CELL_JUNCTION_ORGANIZATION | 59 | 0.461 | 1.648 | 0.002 | 0.092 | 1.000 | 1751 |
| REACTOME_POST_CHAPERONIN_TUBULIN_FOLDING_PATHWAY | 17 | 0.600 | 1.645 | 0.017 | 0.094 | 1.000 | 2421 |
| REACTOME_SIGNALING_BY_INSULIN_RECEPTOR | 54 | 0.461 | 1.643 | 0.004 | 0.095 | 1.000 | 3485 |
| REACTOME_HS_GAG_DEGRADATION | 17 | 0.601 | 1.637 | 0.025 | 0.099 | 1.000 | 2177 |
| REACTOME_INTERLEUKIN_17_SIGNALING | 65 | 0.439 | 1.636 | 0.008 | 0.099 | 1.000 | 2911 |
| REACTOME_RAF_INDEPENDENT_MAPK1_3_ACTIVATION | 22 | 0.560 | 1.634 | 0.017 | 0.101 | 1.000 | 4416 |

| | | | | | | | |
|---|---|---|---|---|---|---|---|
| REACTOME_RHO_GTPASES_ACTIVATE_WASPS_AND_WAVES | 33 | 0.517 | 1.633 | 0.007 | 0.100 | 1.000 | 3912 |
| REACTOME_SIGNALING_BY_TGF_BETA_RECEPTOR_COMPLEX_IN_CANCER | 8 | 0.729 | 1.632 | 0.015 | 0.100 | 1.000 | 3446 |
| REACTOME_NEGATIVE_REGULATION_OF_FGFR1_SIGNALING | 20 | 0.574 | 1.631 | 0.016 | 0.101 | 1.000 | 2088 |
| REACTOME_INTEGRIN_SIGNALING | 21 | 0.550 | 1.628 | 0.019 | 0.102 | 1.000 | 2038 |
| REACTOME_INFLUENZA_INFECTION | 151 | 0.381 | 1.625 | 0.000 | 0.106 | 1.000 | 4025 |
| REACTOME_EGFR_DOWNREGULATION | 25 | 0.534 | 1.621 | 0.014 | 0.108 | 1.000 | 1986 |
| REACTOME_TOLL_LIKE_RECEPTOR_9_TLR9_CASCADE | 85 | 0.418 | 1.620 | 0.005 | 0.108 | 1.000 | 2936 |
| REACTOME_TRANSLOCATION_OF_SLC2A4_GLUT4_TO_THE_PLASMA_MEMBRANE | 63 | 0.441 | 1.620 | 0.009 | 0.108 | 1.000 | 3691 |
| REACTOME_GROWTH_HORMONE_RECEPTOR_SIGNALING | 19 | 0.575 | 1.619 | 0.018 | 0.108 | 1.000 | 3480 |
| REACTOME_SIGNALING_BY_PDGF | 40 | 0.486 | 1.616 | 0.006 | 0.110 | 1.000 | 846 |
| REACTOME_CELLULAR_RESPONSES_TO_EXTERNAL_STIMULI | 499 | 0.338 | 1.615 | 0.000 | 0.110 | 1.000 | 3576 |
| REACTOME_RHO_GTPASES_ACTIVATE_PKNS | 39 | 0.469 | 1.613 | 0.010 | 0.111 | 1.000 | 3507 |
| REACTOME_REGULATION_OF_TLR_BY_ENDOGENOUS_LIGAND | 5 | 0.822 | 1.613 | 0.010 | 0.111 | 1.000 | 355 |
| REACTOME_REGULATION_OF_INSULIN_LIKE_GROWTH_FACTOR_IGF_TRANSPORT_AND_UPTAKE_BY_INSULIN_LIKE_GROWTH_FACTOR_BINDING_PROTEINS_IGFBPS_ | 65 | 0.434 | 1.611 | 0.005 | 0.111 | 1.000 | 2249 |
| REACTOME_O_GLYCOSYLATION_OF_TSR_DOMAIN_CONTAINING_PROTEINS | 20 | 0.565 | 1.611 | 0.023 | 0.111 | 1.000 | 1450 |
| REACTOME_PLASMA_LIPOPROTEIN_REMODELING | 15 | 0.603 | 1.606 | 0.026 | 0.115 | 1.000 | 1700 |
| REACTOME_TRAF6_MEDIATED_INDUCTION_OF_TAK1_COMPLEX_WITHIN_TLR4_COMPLEX | 14 | 0.619 | 1.604 | 0.031 | 0.117 | 1.000 | 1569 |
| REACTOME_CLASS_A_1_RHODOPSIN_LIKE_RECEPTORS_ | 65 | 0.428 | 1.603 | 0.007 | 0.117 | 1.000 | 1549 |
| REACTOME_ACTIVATION_OF_IRF3_IRF7_MEDIATED_BY_TBK1_IKK_EPSILON | 15 | 0.608 | 1.602 | 0.016 | 0.117 | 1.000 | 1755 |
| REACTOME_TRANSFERRIN_ENDOCYTOSIS_AND_RECYCLING | 26 | 0.531 | 1.602 | 0.019 | 0.116 | 1.000 | 3485 |
| REACTOME_HS_GAG_BIOSYNTHESIS | 18 | 0.579 | 1.602 | 0.021 | 0.116 | 1.000 | 2820 |
| REACTOME_SPRY_REGULATION_OF_FGF_SIGNALING | 16 | 0.600 | 1.600 | 0.027 | 0.116 | 1.000 | 2088 |

| | | | | | | | |
|---|---|---|---|---|---|---|---|
| REACTOME_EGFR_INTERACTS_WITH_PHOSPHOLIPASE_C_GAMMA | 5 | 0.831 | 1.599 | 0.020 | 0.117 | 1.000 | 922 |
| REACTOME_ACTIVATION_OF_BAD_AND_TRANSLOCATION_TO_MITOCHONDRIA_ | 15 | 0.596 | 1.599 | 0.039 | 0.116 | 1.000 | 3691 |
| REACTOME_ADVANCED_GLYCOSYLATION_ENDPRODUCT_RECEPTOR_SIGNALING | 10 | 0.672 | 1.599 | 0.029 | 0.115 | 1.000 | 2936 |
| REACTOME_ION_TRANSPORT_BY_P_TYPE_ATPASES | 36 | 0.487 | 1.598 | 0.020 | 0.115 | 1.000 | 1427 |
| REACTOME_TOLL_LIKE_RECEPTOR_10_TLR10_CASCADE | 77 | 0.429 | 1.597 | 0.007 | 0.115 | 1.000 | 2936 |
| REACTOME_DEFECTIVE_CHST14_CAUSES_EDS_MUSCULOCONTRACTURAL_TYPE | 3 | 0.933 | 1.595 | 0.004 | 0.116 | 1.000 | 850 |
| REACTOME_CREB1_PHOSPHORYLATION_THROUGH_NMDA_RECEPTOR_MEDIATED_ACTIVATION_OF_RAS_SIGNALING | 22 | 0.535 | 1.593 | 0.009 | 0.118 | 1.000 | 649 |
| REACTOME_REGULATION_OF_LOCALIZATION_OF_FOXO_TRANSCRIPTION_FACTORS | 12 | 0.639 | 1.590 | 0.033 | 0.121 | 1.000 | 3691 |
| REACTOME_SIGNALLING_TO_ERKS | 30 | 0.498 | 1.585 | 0.021 | 0.125 | 1.000 | 4322 |
| REACTOME_NEUROTRANSMITTER_RECEPTORS_AND_POSTSYNAPTIC_SIGNAL_TRANSMISSION | 109 | 0.393 | 1.585 | 0.002 | 0.125 | 1.000 | 2182 |
| REACTOME_MYD88_INDEPENDENT_TLR4_CASCADE_ | 90 | 0.400 | 1.584 | 0.007 | 0.125 | 1.000 | 2936 |
| REACTOME_COPII_MEDIATED_VESICLE_TRANSPORT | 66 | 0.426 | 1.578 | 0.003 | 0.131 | 1.000 | 2582 |
| REACTOME_COPI_MEDIATED_ANTEROGRADE_TRANSPORT | 88 | 0.399 | 1.575 | 0.008 | 0.133 | 1.000 | 3023 |
| REACTOME_P130CAS_LINKAGE_TO_MAPK_SIGNALING_FOR_INTEGRINS | 11 | 0.647 | 1.574 | 0.037 | 0.133 | 1.000 | 897 |
| REACTOME_TRANSMISSION_ACROSS_CHEMICAL_SYNAPSES | 139 | 0.375 | 1.574 | 0.003 | 0.133 | 1.000 | 2182 |
| REACTOME_GENERATION_OF_SECOND_MESSENGER_MOLECULES | 13 | 0.621 | 1.572 | 0.034 | 0.134 | 1.000 | 1595 |
| REACTOME_OLFACTORY_SIGNALING_PATHWAY | 8 | 0.712 | 1.569 | 0.024 | 0.136 | 1.000 | 2916 |
| REACTOME_CELL_CELL_COMMUNICATION | 84 | 0.401 | 1.569 | 0.005 | 0.136 | 1.000 | 1751 |
| REACTOME_DISEASES_OF_GLYCOSYLATION | 97 | 0.395 | 1.567 | 0.010 | 0.137 | 1.000 | 1630 |
| REACTOME_PLASMA_LIPOPROTEIN_ASSEMBLY_REMODELING_AND_CLEARANCE | 47 | 0.450 | 1.564 | 0.004 | 0.140 | 1.000 | 817 |
| REACTOME_SIGNAL_TRANSDUCTION_BY_L1 | 17 | 0.570 | 1.555 | 0.023 | 0.150 | 1.000 | 3355 |

| | | | | | | | |
|---|---|---|---|---|---|---|---|
| REACTOME_PARASITE_INFECTION | 48 | 0.444 | 1.554 | 0.017 | 0.151 | 1.000 | 3912 |
| REACTOME_PYRIMIDINE_SALVAGE | 8 | 0.682 | 1.551 | 0.041 | 0.153 | 1.000 | 2256 |
| REACTOME_AGGREPHAGY | 32 | 0.485 | 1.549 | 0.029 | 0.154 | 1.000 | 2078 |
| REACTOME_WNT_LIGAND_BIOGENESIS_AND_TRAFFICKING | 13 | 0.591 | 1.545 | 0.036 | 0.159 | 1.000 | 617 |
| REACTOME_DEFECTIVE_CHST3_CAUSES_SEDCJD | 3 | 0.921 | 1.543 | 0.008 | 0.161 | 1.000 | 1010 |
| REACTOME_IMMUNOREGULATORY_INTERACTIONS_BETWEEN_A_LYMPHOID_AND_A_NON_LYMPHOID_CELL | 29 | 0.484 | 1.543 | 0.026 | 0.161 | 1.000 | 1740 |
| REACTOME_ACTIVATION_OF_THE_AP_1_FAMILY_OF_TRANSCRIPTION_FACTORS | 9 | 0.680 | 1.539 | 0.047 | 0.165 | 1.000 | 1028 |
| REACTOME_REVERSIBLE_HYDRATION_OF_CARBON_DIOXIDE | 7 | 0.715 | 1.529 | 0.031 | 0.177 | 1.000 | 2164 |
| REACTOME_TP53_REGULATES_METABOLIC_GENES | 81 | 0.397 | 1.528 | 0.012 | 0.177 | 1.000 | 3381 |
| REACTOME_TGF_BETA_RECEPTOR_SIGNALING_ACTIVATES_SMADS | 32 | 0.473 | 1.527 | 0.034 | 0.178 | 1.000 | 3779 |
| REACTOME_CLASS_C_3_METABOTROPIC_GLUTAMATE_PHEROMONE_RECEPTORS_ | 3 | 0.908 | 1.526 | 0.012 | 0.178 | 1.000 | 782 |
| REACTOME_MAP2K_AND_MAPK_ACTIVATION | 35 | 0.456 | 1.524 | 0.035 | 0.180 | 1.000 | 1751 |
| REACTOME_COMPLEMENT_CASCADE | 15 | 0.575 | 1.522 | 0.037 | 0.182 | 1.000 | 2284 |
| REACTOME_DISEASES_OF_SIGNAL_TRANSDUCTION_BY_GROWTH_FACTOR_RECEPTORS_AND_SECOND_MESSENGERS | 329 | 0.328 | 1.520 | 0.000 | 0.184 | 1.000 | 3989 |
| REACTOME_REGULATION_OF_GLYCOLYSIS_BY_FRUCTOSE_2_6_BISPHOSPHATE_METABOLISM | 10 | 0.641 | 1.520 | 0.043 | 0.183 | 1.000 | 2666 |
| REACTOME_TP53_REGULATES_TRANSCRIPTION_OF_GENES_INVOLVED_IN_CYTOCHROME_C_RELEASE | 18 | 0.543 | 1.520 | 0.034 | 0.182 | 1.000 | 2991 |
| REACTOME_SYNAPTIC_ADHESION_LIKE_MOLECULES | 14 | 0.575 | 1.520 | 0.043 | 0.182 | 1.000 | 3181 |
| REACTOME_IL_6_TYPE_CYTOKINE_RECEPTOR_LIGAND_INTERACTIONS | 8 | 0.686 | 1.516 | 0.046 | 0.186 | 1.000 | 1206 |
| REACTOME_ACTIVATION_OF_C3_AND_C5 | 5 | 0.773 | 1.512 | 0.045 | 0.191 | 1.000 | 2284 |
| REACTOME_HDL_REMODELING | 4 | 0.833 | 1.511 | 0.032 | 0.191 | 1.000 | 1370 |
| REACTOME_GAB1_SIGNALOSOME | 12 | 0.617 | 1.510 | 0.060 | 0.192 | 1.000 | 922 |

| | | | | | | | |
|---|---|---|---|---|---|---|---|
| REACTOME_CREB_PHOSPHORYLATION | 7 | 0.698 | 1.509 | 0.058 | 0.191 | 1.000 | 1445 |
| REACTOME_HSF1_DEPENDENT_TRANSACTIVATION | 31 | 0.470 | 1.507 | 0.031 | 0.194 | 1.000 | 4731 |
| REACTOME_RUNX1_REGULATES_TRANSCRIPTION_OF_GENES_INVOLVED_IN_DIFFERENTIATION_OF_MYELOID_CELLS | 5 | 0.762 | 1.506 | 0.049 | 0.194 | 1.000 | 2775 |
| REACTOME_PHYSIOLOGICAL_FACTORS | 7 | 0.692 | 1.506 | 0.055 | 0.193 | 1.000 | 2187 |
| REACTOME_MATURATION_OF_SPIKE_PROTEIN | 5 | 0.784 | 1.504 | 0.044 | 0.196 | 1.000 | 229 |
| REACTOME_NUCLEOTIDE_SALVAGE | 19 | 0.538 | 1.502 | 0.039 | 0.198 | 1.000 | 2256 |
| REACTOME_INTERLEUKIN_4_AND_INTERLEUKIN_13_SIGNALING | 62 | 0.413 | 1.501 | 0.024 | 0.198 | 1.000 | 1678 |
| REACTOME_ION_CHANNEL_TRANSPORT | 111 | 0.370 | 1.499 | 0.005 | 0.199 | 1.000 | 2641 |
| REACTOME_REGULATION_OF_GENE_EXPRESSION_BY_HYPOXIA_INDUCIBLE_FACTOR | 9 | 0.652 | 1.495 | 0.045 | 0.204 | 1.000 | 3280 |
| REACTOME_HIGHLY_CALCIUM_PERMEABLE_NICOTINIC_ACETYLCHOLINE_RECEPTORS | 4 | 0.811 | 1.493 | 0.049 | 0.206 | 1.000 | 99 |
| REACTOME_INTERLEUKIN_12_FAMILY_SIGNALING | 42 | 0.431 | 1.492 | 0.037 | 0.207 | 1.000 | 4097 |
| REACTOME_VITAMIN_C_ASCORBATE_METABOLISM | 7 | 0.700 | 1.491 | 0.045 | 0.208 | 1.000 | 3079 |
| REACTOME_FOXO_MEDIATED_TRANSCRIPTION_OF_CELL_CYCLE_GENES | 16 | 0.539 | 1.488 | 0.058 | 0.211 | 1.000 | 1909 |
| REACTOME_SIGNALING_BY_MRAS_COMPLEX_MUTANTS | 8 | 0.662 | 1.487 | 0.055 | 0.211 | 1.000 | 3789 |
| REACTOME_INTERLEUKIN_10_SIGNALING | 18 | 0.539 | 1.486 | 0.049 | 0.212 | 1.000 | 1229 |
| REACTOME_SCAVENGING_BY_CLASS_F_RECEPTORS | 4 | 0.806 | 1.482 | 0.048 | 0.217 | 1.000 | 2078 |
| REACTOME_UPTAKE_AND_FUNCTION_OF_DIPHTHERIA_TOXIN | 6 | 0.734 | 1.482 | 0.050 | 0.216 | 1.000 | 2078 |
| REACTOME_NR1H3_NR1H2_REGULATE_GENE_EXPRESSION_LINKED_TO_CHOLESTEROL_TRANSPORT_AND_EFFLUX | 31 | 0.460 | 1.482 | 0.036 | 0.216 | 1.000 | 2974 |
| REACTOME_PLATELET_AGGREGATION_PLUG_FORMATION_ | 24 | 0.491 | 1.481 | 0.058 | 0.216 | 1.000 | 2038 |
| REACTOME_HIGHLY_CALCIUM_PERMEABLE_POSTSYNAPTIC_NICOTINIC_ACETYLCHOLINE_RECEPTORS | 4 | 0.811 | 1.481 | 0.045 | 0.215 | 1.000 | 99 |
| REACTOME_PEPTIDE_HORMONE_BIOSYNTHESIS | 3 | 0.905 | 1.480 | 0.021 | 0.214 | 1.000 | 562 |
| REACTOME_P75NTR_REGULATES_AXONOGENESIS | 5 | 0.751 | 1.479 | 0.061 | 0.215 | 1.000 | 6 |

| | | | | | | | |
|---|---|---|---|---|---|---|---|
| REACTOME_INFECTION_WITH_MYCOBACTERIUM_TUBERCULOSIS | 23 | 0.499 | 1.477 | 0.052 | 0.217 | 1.000 | 2576 |
| REACTOME_UREA_CYCLE | 5 | 0.761 | 1.476 | 0.042 | 0.219 | 1.000 | 572 |
| REACTOME_DEFECTIVE_CHSY1_CAUSES_TPBS | 3 | 0.879 | 1.475 | 0.022 | 0.219 | 1.000 | 354 |
| REACTOME_PECAM1_INTERACTIONS | 10 | 0.615 | 1.475 | 0.072 | 0.218 | 1.000 | 862 |
| REACTOME_GOLGI_TO_ER_RETROGRADE_TRANSPORT | 118 | 0.360 | 1.472 | 0.017 | 0.222 | 1.000 | 2582 |
| REACTOME_COPI_DEPENDENT_GOLGI_TO_ER_RETROGRADE_TRAFFIC | 88 | 0.377 | 1.469 | 0.018 | 0.226 | 1.000 | 2582 |
| REACTOME_AKT_PHOSPHORYLATES_TARGETS_IN_THE_NUCLEUS | 10 | 0.625 | 1.467 | 0.072 | 0.228 | 1.000 | 2117 |
| REACTOME_RESPONSE_OF_MTB_TO_PHAGOCYTOSIS | 21 | 0.511 | 1.467 | 0.068 | 0.228 | 1.000 | 2576 |
| REACTOME_ERK_MAPK_TARGETS | 21 | 0.503 | 1.465 | 0.048 | 0.230 | 1.000 | 2574 |
| REACTOME_GASTRIN_CREB_SIGNALLING_PATHWAY_VIA_PKC_AND_MAPK | 14 | 0.558 | 1.464 | 0.059 | 0.231 | 1.000 | 649 |
| REACTOME_NEGATIVE_FEEDBACK_REGULATION_OF_MAPK_PATHWAY | 6 | 0.704 | 1.462 | 0.055 | 0.231 | 1.000 | 1476 |
| REACTOME_REGULATION_OF_IFNG_SIGNALING | 13 | 0.579 | 1.461 | 0.055 | 0.232 | 1.000 | 4357 |
| REACTOME_TRANSPORT_TO_THE_GOLGI_AND_SUBSEQUENT_MODIFICATION | 159 | 0.341 | 1.461 | 0.013 | 0.231 | 1.000 | 2582 |
| REACTOME_EPHRIN_SIGNALING | 17 | 0.532 | 1.461 | 0.063 | 0.231 | 1.000 | 2694 |
| REACTOME_ATF6_ATF6_ALPHA_ACTIVATES_CHAPERONES | 12 | 0.580 | 1.459 | 0.070 | 0.232 | 1.000 | 4029 |
| REACTOME_REGULATION_OF_COMMISSURAL_AXON_PATHFINDING_BY_SLIT_AND_ROBO | 4 | 0.802 | 1.459 | 0.044 | 0.231 | 1.000 | 1185 |
| REACTOME_IKK_COMPLEX_RECRUITMENT_MEDIATED_BY_RIP1 | 21 | 0.500 | 1.458 | 0.066 | 0.232 | 1.000 | 1722 |
| REACTOME_DISEASES_ASSOCIATED_WITH_GLYCOSAMINOGLYCAN_METABOLISM | 27 | 0.466 | 1.457 | 0.049 | 0.232 | 1.000 | 2212 |
| REACTOME_L1CAM_INTERACTIONS | 84 | 0.383 | 1.455 | 0.025 | 0.235 | 1.000 | 1959 |
| REACTOME_PTK6_REGULATES_RHO_GTPASES_RAS_GTPASE_AND_MAP_KINASES | 13 | 0.562 | 1.452 | 0.088 | 0.238 | 1.000 | 4150 |
| REACTOME_SHC_MEDIATED_CASCADE_FGFR4 | 10 | 0.598 | 1.452 | 0.065 | 0.238 | 1.000 | 1020 |
| REACTOME_DEFECTIVE_C1GALT1C1_CAUSES_TN_POLYAGGLUTINATION_SYNDROME_TNPS_ | 5 | 0.753 | 1.451 | 0.054 | 0.239 | 1.000 | 3026 |

| | | | | | | | |
|---|---|---|---|---|---|---|---|
| REACTOME_DISEASES_OF_METABOLISM | 165 | 0.340 | 1.450 | 0.009 | 0.238 | 1.000 | 2234 |
| REACTOME_PRESYNAPTIC_DEPOLARIZATION_AND_CALCIUM_CHANNEL_OPENING | 4 | 0.789 | 1.450 | 0.053 | 0.238 | 1.000 | 635 |
| REACTOME_NEGATIVE_REGULATION_OF_TCF_DEPENDENT_SIGNALING_BY_WNT_LIGAND_ANTAGONISTS | 5 | 0.748 | 1.450 | 0.079 | 0.238 | 1.000 | 27 |
| REACTOME_RSK_ACTIVATION | 7 | 0.670 | 1.449 | 0.056 | 0.237 | 1.000 | 649 |
| REACTOME_INSULIN_RECEPTOR_SIGNALLING_CASCADE | 34 | 0.449 | 1.448 | 0.047 | 0.238 | 1.000 | 1544 |
| REACTOME_IONOTROPIC_ACTIVITY_OF_KAINATE_RECEPTORS | 6 | 0.711 | 1.447 | 0.060 | 0.239 | 1.000 | 2486 |
| REACTOME_VISUAL_PHOTOTRANSDUCTION | 47 | 0.418 | 1.447 | 0.042 | 0.239 | 1.000 | 2414 |
| REACTOME_DOWNREGULATION_OF_TGF_BETA_RECEPTOR_SIGNALING | 26 | 0.473 | 1.446 | 0.049 | 0.239 | 1.000 | 3229 |
| REACTOME_EXTRA_NUCLEAR_ESTROGEN_SIGNALING | 52 | 0.412 | 1.446 | 0.033 | 0.238 | 1.000 | 2180 |
| REACTOME_CD28_DEPENDENT_VAV1_PATHWAY | 8 | 0.640 | 1.444 | 0.075 | 0.240 | 1.000 | 2378 |
| REACTOME_VLDL_ASSEMBLY | 2 | 0.951 | 1.441 | 0.012 | 0.244 | 1.000 | 406 |
| REACTOME_DISEASES_ASSOCIATED_WITH_O_GLYCOSYLATION_OF_PROTEINS | 36 | 0.440 | 1.438 | 0.060 | 0.247 | 1.000 | 1630 |
| REACTOME_THE_ROLE_OF_GTSE1_IN_G2_M_PROGRESSION_AFTER_G2_CHECKPOINT | 69 | 0.381 | 1.438 | 0.031 | 0.247 | 1.000 | 4312 |
| REACTOME_TP53_REGULATES_TRANSCRIPTION_OF_SEVERAL_ADDITIONAL_CELL_DEATH_GENES_WHOSE_SPECIFIC_ROLES_IN_P53_DEPENDENT_APOPTOSIS_REMAIN_UNCERTAIN | 12 | 0.574 | 1.435 | 0.088 | 0.251 | 1.000 | 2991 |
| REACTOME_NCAM1_INTERACTIONS | 15 | 0.540 | 1.434 | 0.092 | 0.251 | 1.000 | 579 |
| REACTOME_STRIATED_MUSCLE_CONTRACTION | 15 | 0.538 | 1.433 | 0.077 | 0.252 | 1.000 | 1318 |
| REACTOME_IRS_ACTIVATION | 4 | 0.772 | 1.432 | 0.068 | 0.252 | 1.000 | 1544 |
| REACTOME_RAS_ACTIVATION_UPON_CA2_INFLUX_THROUGH_NMDA_RECEPTOR | 14 | 0.538 | 1.431 | 0.068 | 0.253 | 1.000 | 3953 |
| REACTOME_SIGNALING_BY_FGFR1 | 33 | 0.446 | 1.429 | 0.068 | 0.256 | 1.000 | 2125 |
| REACTOME_FORMATION_OF_FIBRIN_CLOT_CLOTTING_CASCADE_ | 18 | 0.513 | 1.429 | 0.086 | 0.254 | 1.000 | 1233 |
| REACTOME_TOLL_LIKE_RECEPTOR_CASCADES | 121 | 0.348 | 1.428 | 0.020 | 0.255 | 1.000 | 2936 |

| | | | | | | | |
|---|---|---|---|---|---|---|---|
| REACTOME_HIGHLY_SODIUM_PERMEABLE_POSTSYNAPTIC_ACETYLCHOLINE_NICOTINIC_RECEPTORS | 2 | 0.947 | 1.427 | 0.012 | 0.255 | 1.000 | 123 |
| REACTOME_GPVI_MEDIATED_ACTIVATION_CASCADE | 24 | 0.466 | 1.425 | 0.043 | 0.258 | 1.000 | 3621 |
| REACTOME_RAF_ACTIVATION | 32 | 0.450 | 1.424 | 0.034 | 0.259 | 1.000 | 4331 |
| REACTOME_NADE_MODULATES_DEATH_SIGNALLING | 5 | 0.744 | 1.423 | 0.079 | 0.259 | 1.000 | 6 |
| REACTOME_PHENYLALANINE_AND_TYROSINE_METABOLISM | 7 | 0.662 | 1.423 | 0.111 | 0.259 | 1.000 | 3191 |
| REACTOME_SIGNALLING_TO_P38_VIA_RIT_AND_RIN | 2 | 0.920 | 1.422 | 0.031 | 0.260 | 1.000 | 490 |
| REACTOME_MET_RECEPTOR_ACTIVATION | 4 | 0.781 | 1.421 | 0.074 | 0.261 | 1.000 | 2790 |
| REACTOME_NOTCH2_ACTIVATION_AND_TRANSMISSION_OF_SIGNAL_TO_THE_NUCLEUS | 20 | 0.486 | 1.420 | 0.080 | 0.260 | 1.000 | 1569 |
| REACTOME_INTERLEUKIN_7_SIGNALING | 16 | 0.528 | 1.416 | 0.070 | 0.266 | 1.000 | 56 |
| REACTOME_CARDIAC_CONDUCTION | 65 | 0.387 | 1.415 | 0.031 | 0.268 | 1.000 | 1376 |
| REACTOME_RESPIRATORY_ELECTRON_TRANSPORT | 90 | 0.363 | 1.412 | 0.041 | 0.271 | 1.000 | 4134 |
| REACTOME_ESR_MEDIATED_SIGNALING | 142 | 0.334 | 1.412 | 0.016 | 0.270 | 1.000 | 2236 |
| REACTOME_SIGNALING_BY_FGFR4 | 30 | 0.453 | 1.411 | 0.059 | 0.272 | 1.000 | 2088 |
| REACTOME_SIGNALING_BY_GPCR | 314 | 0.305 | 1.411 | 0.003 | 0.271 | 1.000 | 1715 |
| REACTOME_INITIAL_TRIGGERING_OF_COMPLEMENT | 7 | 0.662 | 1.409 | 0.089 | 0.272 | 1.000 | 2284 |
| REACTOME_IRAK4_DEFICIENCY_TLR2_4_ | 5 | 0.717 | 1.407 | 0.082 | 0.275 | 1.000 | 317 |
| REACTOME_DEFECTIVE_FACTOR_IX_CAUSES_HEMOPHILIA_B | 3 | 0.830 | 1.406 | 0.066 | 0.276 | 1.000 | 333 |
| REACTOME_MET_ACTIVATES_PTK2_SIGNALING | 20 | 0.499 | 1.406 | 0.073 | 0.275 | 1.000 | 1771 |
| REACTOME_GLYCOSAMINOGLYCAN_METABOLISM | 85 | 0.366 | 1.404 | 0.045 | 0.277 | 1.000 | 2212 |
| REACTOME_FGFR3B_LIGAND_BINDING_AND_ACTIVATION | 3 | 0.820 | 1.401 | 0.088 | 0.281 | 1.000 | 1136 |
| REACTOME_POTASSIUM_CHANNELS | 33 | 0.441 | 1.399 | 0.069 | 0.284 | 1.000 | 2318 |
| REACTOME_CREATION_OF_C4_AND_C2_ACTIVATORS | 2 | 0.933 | 1.399 | 0.048 | 0.283 | 1.000 | 848 |
| REACTOME_PTK6_PROMOTES_HIF1A_STABILIZATION | 3 | 0.845 | 1.396 | 0.070 | 0.287 | 1.000 | 1421 |
| REACTOME_METABOLISM_OF_FAT_SOLUBLE_VITAMINS | 27 | 0.460 | 1.395 | 0.091 | 0.287 | 1.000 | 2414 |
| REACTOME_EXTRINSIC_PATHWAY_OF_FIBRIN_CLOT_FORMATION | 4 | 0.760 | 1.393 | 0.095 | 0.291 | 1.000 | 889 |

| | | | | | | | |
|---|---|---|---|---|---|---|---|
| REACTOME_RUNX2_REGULATES_GENES_INVOLVED_IN_CELL_MIGRATION | 6 | 0.681 | 1.392 | 0.107 | 0.291 | 1.000 | 3691 |
| REACTOME_LAMININ_INTERACTIONS | 22 | 0.465 | 1.390 | 0.086 | 0.294 | 1.000 | 1289 |
| REACTOME_SELECTIVE_AUTOPHAGY | 69 | 0.371 | 1.389 | 0.049 | 0.294 | 1.000 | 2103 |
| REACTOME_INTERLEUKIN_6_SIGNALING | 10 | 0.570 | 1.388 | 0.104 | 0.296 | 1.000 | 3707 |
| REACTOME_CROSS_PRESENTATION_OF_PARTICULATE_EXOGENOUS_ANTIGENS_PHAGOSOMES_ | 3 | 0.810 | 1.387 | 0.098 | 0.296 | 1.000 | 1792 |
| REACTOME_ABC_TRANSPORTERS_IN_LIPID_HOMEOSTASIS | 9 | 0.590 | 1.387 | 0.114 | 0.295 | 1.000 | 2740 |
| REACTOME_SHC_MEDIATED_CASCADE_FGFR3 | 9 | 0.595 | 1.386 | 0.110 | 0.296 | 1.000 | 1136 |
| REACTOME_SIGNALING_BY_LEPTIN | 10 | 0.577 | 1.386 | 0.117 | 0.295 | 1.000 | 3480 |
| REACTOME_DEFECTS_OF_CONTACT_ACTIVATION_SYSTEM_CAS_AND_KALLIKREIN_KININ_SYSTEM_KKS_ | 8 | 0.626 | 1.386 | 0.117 | 0.294 | 1.000 | 1170 |
| REACTOME_COMMON_PATHWAY_OF_FIBRIN_CLOT_FORMATION | 11 | 0.556 | 1.381 | 0.119 | 0.302 | 1.000 | 1233 |
| REACTOME_RHO_GTPASES_ACTIVATE_ROCKS | 18 | 0.501 | 1.380 | 0.084 | 0.302 | 1.000 | 3725 |
| REACTOME_NEGATIVE_REGULATION_OF_NMDA_RECEPTOR_MEDIATED_NEURONAL_TRANSMISSION | 12 | 0.547 | 1.379 | 0.097 | 0.303 | 1.000 | 2486 |
| REACTOME_FGFRL1_MODULATION_OF_FGFR1_SIGNALING | 5 | 0.699 | 1.379 | 0.106 | 0.303 | 1.000 | 2125 |
| REACTOME_SMOOTH_MUSCLE_CONTRACTION | 25 | 0.456 | 1.378 | 0.088 | 0.303 | 1.000 | 1792 |
| REACTOME_COSTIMULATION_BY_THE_CD28_FAMILY | 44 | 0.395 | 1.377 | 0.069 | 0.304 | 1.000 | 2441 |
| REACTOME_INTERLEUKIN_2_SIGNALING | 7 | 0.648 | 1.377 | 0.113 | 0.303 | 1.000 | 1162 |
| REACTOME_HSF1_ACTIVATION | 26 | 0.450 | 1.376 | 0.092 | 0.303 | 1.000 | 3861 |
| REACTOME_RHO_GTPASE_EFFECTORS | 241 | 0.307 | 1.376 | 0.013 | 0.303 | 1.000 | 3639 |
| REACTOME_MISSPLICED_LRP5_MUTANTS_HAVE_ENHANCED_BETA_CATENIN_DEPENDENT_SIGNALING | 4 | 0.753 | 1.376 | 0.112 | 0.302 | 1.000 | 27 |
| REACTOME_RHO_GTPASES_ACTIVATE_PAKS | 19 | 0.484 | 1.375 | 0.111 | 0.302 | 1.000 | 3943 |
| REACTOME_SPERM_MOTILITY_AND_TAXES | 2 | 0.920 | 1.374 | 0.040 | 0.303 | 1.000 | 152 |
| REACTOME_RESPIRATORY_ELECTRON_TRANSPORT_ATP_SYNTHESIS_BY_CHEMIOSMOTIC_COUPLI | 110 | 0.338 | 1.373 | 0.029 | 0.304 | 1.000 | 4134 |

| | | | | | | | |
|---|---|---|---|---|---|---|---|
| NG_AND_HEAT_PRODUCTION_BY_UNCOUPLING_PROTEINS_ | | | | | | | |
| REACTOME_TCR_SIGNALING | 94 | 0.344 | 1.372 | 0.034 | 0.304 | 1.000 | 4312 |
| REACTOME_UPTAKE_AND_FUNCTION_OF_ANTHRAX_TOXINS | 11 | 0.564 | 1.370 | 0.106 | 0.307 | 1.000 | 3943 |
| REACTOME_SYNTHESIS_OF_ACTIVE_UBIQUITIN_ROLES_OF_E1_AND_E2_ENZYMES | 30 | 0.439 | 1.370 | 0.084 | 0.307 | 1.000 | 4884 |
| REACTOME_NOSTRIN_MEDIATED_ENOS_TRAFFICKING | 3 | 0.809 | 1.368 | 0.111 | 0.308 | 1.000 | 206 |
| REACTOME_INTERLEUKIN_3_INTERLEUKIN_5_AND_GM_CSF_SIGNALING | 34 | 0.415 | 1.366 | 0.063 | 0.311 | 1.000 | 3779 |
| REACTOME_DEFECTIVE_FACTOR_VIII_CAUSES_HEMOPHILIA_A | 6 | 0.661 | 1.365 | 0.118 | 0.311 | 1.000 | 1170 |
| REACTOME_ACTIVATION_OF_THE_PHOTOTRANSDUCTION_CASCADE | 2 | 0.878 | 1.365 | 0.086 | 0.312 | 1.000 | 232 |
| REACTOME_RECEPTOR_MEDIATED_MITOPHAGY | 11 | 0.561 | 1.364 | 0.114 | 0.312 | 1.000 | 3677 |
| REACTOME_PLATELET_SENSITIZATION_BY_LDL | 13 | 0.535 | 1.363 | 0.135 | 0.313 | 1.000 | 3243 |
| REACTOME_UNFOLDED_PROTEIN_RESPONSE_UPR_ | 87 | 0.344 | 1.360 | 0.051 | 0.317 | 1.000 | 2571 |
| REACTOME_PLASMA_LIPOPROTEIN_CLEARANCE | 27 | 0.445 | 1.360 | 0.099 | 0.316 | 1.000 | 817 |
| REACTOME_IRON_UPTAKE_AND_TRANSPORT | 47 | 0.394 | 1.359 | 0.073 | 0.318 | 1.000 | 3808 |
| REACTOME_SEMAPHORIN_INTERACTIONS | 51 | 0.381 | 1.358 | 0.064 | 0.317 | 1.000 | 2416 |
| REACTOME_MYOGENESIS | 16 | 0.503 | 1.358 | 0.098 | 0.317 | 1.000 | 3680 |
| REACTOME_P38MAPK_EVENTS | 13 | 0.532 | 1.357 | 0.104 | 0.317 | 1.000 | 4269 |
| REACTOME_CELLULAR_RESPONSE_TO_HYPOXIA | 70 | 0.361 | 1.357 | 0.048 | 0.316 | 1.000 | 4312 |
| REACTOME_ACTIVATED_TAK1_MEDIATES_P38_MAPK_ACTIVATION | 23 | 0.454 | 1.355 | 0.095 | 0.320 | 1.000 | 1873 |
| REACTOME_ERYTHROPOIETIN_ACTIVATES_RAS | 13 | 0.523 | 1.354 | 0.120 | 0.320 | 1.000 | 3953 |
| REACTOME_CLATHRIN_MEDIATED_ENDOCYTOSIS | 116 | 0.333 | 1.352 | 0.035 | 0.323 | 1.000 | 3973 |
| REACTOME_EGR2_AND_SOX10_MEDIATED_INITIATION_OF_SCHWANN_CELL_MYELINATION | 24 | 0.450 | 1.351 | 0.106 | 0.323 | 1.000 | 1124 |
| REACTOME_CHYLOMICRON_REMODELING | 1 | 0.993 | 1.351 | 0.020 | 0.323 | 1.000 | 88 |
| REACTOME_SIGNALING_BY_MET | 63 | 0.363 | 1.351 | 0.073 | 0.322 | 1.000 | 3953 |
| REACTOME_METABOLISM_OF_ANGIOTENSINOGEN_TO_ANGIOTENSINS | 7 | 0.636 | 1.348 | 0.141 | 0.326 | 1.000 | 2450 |
| REACTOME_IRE1ALPHA_ACTIVATES_CHAPERONES | 47 | 0.384 | 1.347 | 0.081 | 0.327 | 1.000 | 2571 |

| | | | | | | | |
|---|---|---|---|---|---|---|---|
| REACTOME_ATF6_ATF6_ALPHA_ACTIVATES_CHAPERONE_GENES | 10 | 0.570 | 1.347 | 0.140 | 0.326 | 1.000 | 4029 |
| REACTOME_SIGNALING_BY_ERBB4 | 38 | 0.406 | 1.345 | 0.080 | 0.328 | 1.000 | 1569 |
| REACTOME_PTK6_REGULATES_RTKS_AND_THEIR_EFFECTORS_AKT1_AND_DOK1 | 9 | 0.574 | 1.345 | 0.129 | 0.328 | 1.000 | 3779 |
| REACTOME_FGFR2_MUTANT_RECEPTOR_ACTIVATION | 19 | 0.471 | 1.344 | 0.115 | 0.328 | 1.000 | 2357 |
| REACTOME_PROLONGED_ERK_ACTIVATION_EVENTS | 12 | 0.534 | 1.343 | 0.121 | 0.330 | 1.000 | 4322 |
| REACTOME_EPHA_MEDIATED_GROWTH_CONE_COLLAPSE | 23 | 0.448 | 1.342 | 0.113 | 0.332 | 1.000 | 1596 |
| REACTOME_GLUCURONIDATION | 4 | 0.734 | 1.340 | 0.147 | 0.334 | 1.000 | 1516 |
| REACTOME_SIGNALING_BY_VEGF | 86 | 0.340 | 1.339 | 0.058 | 0.334 | 1.000 | 3953 |
| REACTOME_SURFACTANT_METABOLISM | 11 | 0.553 | 1.338 | 0.119 | 0.335 | 1.000 | 2740 |
| REACTOME_CHYLOMICRON_CLEARANCE | 4 | 0.724 | 1.336 | 0.138 | 0.338 | 1.000 | 692 |
| REACTOME_ONCOGENE_INDUCED_SENESCENCE | 33 | 0.416 | 1.330 | 0.115 | 0.348 | 1.000 | 1769 |
| REACTOME_RAS_SIGNALING_DOWNSTREAM_OF_NF1_LOSS_OF_FUNCTION_VARIANTS | 7 | 0.636 | 1.330 | 0.139 | 0.348 | 1.000 | 3989 |
| REACTOME_PD_1_SIGNALING | 6 | 0.637 | 1.328 | 0.161 | 0.352 | 1.000 | 1036 |
| REACTOME_PROLACTIN_RECEPTOR_SIGNALING | 10 | 0.562 | 1.327 | 0.142 | 0.352 | 1.000 | 3480 |
| REACTOME_GLYCOGEN_SYNTHESIS | 13 | 0.526 | 1.325 | 0.124 | 0.355 | 1.000 | 1569 |
| REACTOME_FCERI_MEDIATED_MAPK_ACTIVATION | 25 | 0.431 | 1.324 | 0.112 | 0.355 | 1.000 | 3355 |
| REACTOME_SIGNALING_BY_EGFR | 42 | 0.389 | 1.320 | 0.099 | 0.364 | 1.000 | 1986 |
| REACTOME_MHC_CLASS_II_ANTIGEN_PRESENTATION | 95 | 0.337 | 1.319 | 0.065 | 0.364 | 1.000 | 2543 |
| REACTOME_TP53_REGULATES_TRANSCRIPTION_OF_CELL_DEATH_GENES | 37 | 0.398 | 1.318 | 0.094 | 0.365 | 1.000 | 2446 |
| REACTOME_RUNX3_REGULATES_CDKN1A_TRANSCRIPTION | 7 | 0.633 | 1.316 | 0.159 | 0.368 | 1.000 | 2991 |
| REACTOME_INTRINSIC_PATHWAY_OF_FIBRIN_CLOT_FORMATION | 10 | 0.552 | 1.313 | 0.162 | 0.372 | 1.000 | 333 |
| REACTOME_CS_DS_DEGRADATION | 9 | 0.574 | 1.313 | 0.138 | 0.371 | 1.000 | 461 |
| REACTOME_ERYTHROCYTES_TAKE_UP_CARBON_DIOXIDE_AND_RELEASE_OXYGEN | 6 | 0.632 | 1.313 | 0.161 | 0.371 | 1.000 | 1529 |
| REACTOME_APOBEC3G_MEDIATED_RESISTANCE_TO_HIV_1_INFECTION | 5 | 0.669 | 1.312 | 0.177 | 0.373 | 1.000 | 4215 |

| | | | | | | | |
|---|---|---|---|---|---|---|---|
| REACTOME_CD163_MEDIATING_AN_ANTI_INFLAMMATORY_RESPONSE | 7 | 0.609 | 1.311 | 0.161 | 0.372 | 1.000 | 3309 |
| REACTOME_TRANSLATION | 284 | 0.289 | 1.310 | 0.029 | 0.373 | 1.000 | 4231 |
| REACTOME_FERTILIZATION | 6 | 0.643 | 1.310 | 0.152 | 0.373 | 1.000 | 830 |
| REACTOME_RET_SIGNALING | 29 | 0.418 | 1.309 | 0.131 | 0.373 | 1.000 | 1544 |
| REACTOME_SIGNALING_BY_NUCLEAR_RECEPTORS | 195 | 0.300 | 1.307 | 0.039 | 0.377 | 1.000 | 2357 |
| REACTOME_BMAL1_CLOCK_NPAS2_ACTIVATES_CIRCADIAN_GENE_EXPRESSION | 26 | 0.434 | 1.307 | 0.125 | 0.376 | 1.000 | 1886 |
| REACTOME_SIGNALING_BY_PTK6 | 42 | 0.388 | 1.304 | 0.114 | 0.381 | 1.000 | 4357 |
| REACTOME_MAPK3_ERK1_ACTIVATION | 9 | 0.560 | 1.303 | 0.175 | 0.381 | 1.000 | 3707 |
| REACTOME_PLATELET_HOMEOSTASIS | 45 | 0.377 | 1.303 | 0.118 | 0.381 | 1.000 | 2441 |
| REACTOME_SYNTHESIS_OF_PE | 10 | 0.543 | 1.303 | 0.144 | 0.380 | 1.000 | 1456 |
| REACTOME_TOXICITY_OF_BOTULINUM_TOXIN_TYPE_D_BONT_D_ | 3 | 0.776 | 1.302 | 0.124 | 0.379 | 1.000 | 2848 |
| REACTOME_AUTOPHAGY | 134 | 0.310 | 1.301 | 0.063 | 0.381 | 1.000 | 2768 |
| REACTOME_SUPPRESSION_OF_PHAGOSOMAL_MATURATION | 12 | 0.523 | 1.301 | 0.161 | 0.381 | 1.000 | 2576 |
| REACTOME_NETRIN_MEDIATED_REPULSION_SIGNALS | 4 | 0.721 | 1.300 | 0.155 | 0.382 | 1.000 | 400 |
| REACTOME_EGFR_TRANSACTIVATION_BY_GASTRIN | 7 | 0.604 | 1.298 | 0.173 | 0.385 | 1.000 | 176 |
| REACTOME_KINESINS | 50 | 0.368 | 1.297 | 0.085 | 0.385 | 1.000 | 1710 |
| REACTOME_SHC1_EVENTS_IN_ERBB2_SIGNALING | 15 | 0.492 | 1.296 | 0.181 | 0.385 | 1.000 | 1398 |
| REACTOME_SHC1_EVENTS_IN_ERBB4_SIGNALING | 9 | 0.577 | 1.295 | 0.178 | 0.387 | 1.000 | 816 |
| REACTOME_THYROXINE_BIOSYNTHESIS | 1 | 0.963 | 1.294 | 0.065 | 0.388 | 1.000 | 464 |
| REACTOME_PREVENTION_OF_PHAGOSOMAL_LYSOSOMAL_FUSION | 9 | 0.562 | 1.294 | 0.181 | 0.387 | 1.000 | 1977 |
| REACTOME_SIGNALING_BY_MAPK_MUTANTS | 7 | 0.601 | 1.294 | 0.178 | 0.386 | 1.000 | 4650 |
| REACTOME_EPHB_MEDIATED_FORWARD_SIGNALING | 39 | 0.382 | 1.292 | 0.125 | 0.389 | 1.000 | 3390 |
| REACTOME_CROSSLINKING_OF_COLLAGEN_FIBRILS | 9 | 0.551 | 1.291 | 0.150 | 0.391 | 1.000 | 1231 |
| REACTOME_REGULATED_PROTEOLYSIS_OF_P75NTR | 11 | 0.522 | 1.289 | 0.186 | 0.393 | 1.000 | 6 |
| REACTOME_ADRENOCEPTORS | 2 | 0.850 | 1.288 | 0.142 | 0.394 | 1.000 | 320 |
| REACTOME_INTEGRATION_OF_PROVIRUS | 9 | 0.555 | 1.287 | 0.200 | 0.397 | 1.000 | 4837 |
| REACTOME_NRIF_SIGNALS_CELL_DEATH_FROM_THE_NUCLEUS | 15 | 0.492 | 1.286 | 0.177 | 0.396 | 1.000 | 1569 |

| | | | | | | | |
|---|---|---|---|---|---|---|---|
| REACTOME_INTERLEUKIN_23_SIGNALING | 5 | 0.665 | 1.286 | 0.182 | 0.396 | 1.000 | 40 |
| REACTOME_DOWNREGULATION_OF_SMAD2_3_SMAD4_TRANSCRIPTIONAL_ACTIVITY | 23 | 0.431 | 1.286 | 0.142 | 0.395 | 1.000 | 3779 |
| REACTOME_DARPP_32_EVENTS | 19 | 0.450 | 1.283 | 0.152 | 0.399 | 1.000 | 2088 |
| REACTOME_CARBOXYTERMINAL_POST_TRANSLATIONAL_MODIFICATIONS_OF_TUBULIN | 26 | 0.419 | 1.281 | 0.136 | 0.403 | 1.000 | 1710 |
| REACTOME_SERINE_BIOSYNTHESIS | 9 | 0.560 | 1.280 | 0.181 | 0.403 | 1.000 | 1648 |
| REACTOME_ASPARAGINE_N_LINKED_GLYCOSYLATION | 262 | 0.284 | 1.280 | 0.036 | 0.403 | 1.000 | 2647 |
| REACTOME_CLASS_B_2_SECRETIN_FAMILY_RECEPTORS_ | 38 | 0.379 | 1.280 | 0.144 | 0.402 | 1.000 | 2180 |
| REACTOME_INTERLEUKIN_2_FAMILY_SIGNALING | 26 | 0.412 | 1.280 | 0.165 | 0.401 | 1.000 | 3707 |
| REACTOME_SEMA4D_IN_SEMAPHORIN_SIGNALING | 23 | 0.437 | 1.278 | 0.147 | 0.404 | 1.000 | 3949 |
| REACTOME_RUNX1_AND_FOXP3_CONTROL_THE_DEVELOPMENT_OF_REGULATORY_T_LYMPHOCYTES_TREGS_ | 5 | 0.665 | 1.278 | 0.178 | 0.403 | 1.000 | 2832 |
| REACTOME_SEMA4D_MEDIATED_INHIBITION_OF_CELL_ATTACHMENT_AND_MIGRATION | 8 | 0.564 | 1.275 | 0.182 | 0.407 | 1.000 | 3390 |
| REACTOME_PHASE_3_RAPID_REPOLARISATION | 4 | 0.701 | 1.274 | 0.205 | 0.408 | 1.000 | 513 |
| REACTOME_FC_EPSILON_RECEPTOR_FCERI_SIGNALING | 117 | 0.311 | 1.272 | 0.076 | 0.413 | 1.000 | 3985 |
| REACTOME_SIGNALING_BY_FGFR3 | 30 | 0.402 | 1.271 | 0.137 | 0.413 | 1.000 | 2088 |
| REACTOME_SHC_MEDIATED_CASCADE_FGFR1 | 9 | 0.550 | 1.269 | 0.175 | 0.416 | 1.000 | 4782 |
| REACTOME_RHO_GTPASES_ACTIVATE_NADPH_OXIDASES | 14 | 0.488 | 1.269 | 0.170 | 0.416 | 1.000 | 3243 |
| REACTOME_EPH_EPHRIN_SIGNALING | 79 | 0.330 | 1.267 | 0.096 | 0.418 | 1.000 | 1751 |
| REACTOME_VEGF_LIGAND_RECEPTOR_INTERACTIONS | 3 | 0.752 | 1.266 | 0.209 | 0.419 | 1.000 | 1649 |
| REACTOME_MET_ACTIVATES_RAP1_AND_RAC1 | 9 | 0.550 | 1.266 | 0.189 | 0.419 | 1.000 | 4574 |
| REACTOME_ALPHA_PROTEIN_KINASE_1_SIGNALING_PATHWAY | 11 | 0.516 | 1.264 | 0.221 | 0.422 | 1.000 | 1569 |
| REACTOME_SIGNALING_BY_FGFR | 65 | 0.344 | 1.264 | 0.115 | 0.421 | 1.000 | 2125 |
| REACTOME_CALCINEURIN_ACTIVATES_NFAT | 9 | 0.558 | 1.263 | 0.205 | 0.421 | 1.000 | 4551 |
| REACTOME_SIGNALING_BY_MODERATE_KINASE_ACTIVITY_BRAF_MUTANTS | 38 | 0.380 | 1.262 | 0.154 | 0.423 | 1.000 | 1751 |

| | | | | | | | |
|---|---|---|---|---|---|---|---|
| REACTOME_UNBLOCKING_OF_NMDA_RECEPTORS_GLUTAMATE_BINDING_AND_ACTIVATION | 10 | 0.526 | 1.260 | 0.194 | 0.426 | 1.000 | 3066 |
| REACTOME_RUNX1_REGULATES_TRANSCRIPTION_OF_GENES_INVOLVED_IN_DIFFERENTIATION_OF_KERATINOCYTES | 7 | 0.584 | 1.260 | 0.198 | 0.425 | 1.000 | 1908 |
| REACTOME_RUNX1_REGULATES_GENES_INVOLVED_IN_MEGAKARYOCYTE_DIFFERENTIATION_AND_PLATELET_FUNCTION | 44 | 0.360 | 1.257 | 0.141 | 0.429 | 1.000 | 2672 |
| REACTOME_PHOSPHORYLATION_SITE_MUTANTS_OF_CTNNB1_ARE_NOT_TARGETED_TO_THE_PROTEASOME_BY_THE_DESTRUCTION_COMPLEX | 15 | 0.466 | 1.257 | 0.191 | 0.430 | 1.000 | 3597 |
| REACTOME_INTERLEUKIN_21_SIGNALING | 6 | 0.618 | 1.256 | 0.203 | 0.430 | 1.000 | 3707 |
| REACTOME_PHASE_0_RAPID_DEPOLARISATION | 16 | 0.463 | 1.254 | 0.183 | 0.433 | 1.000 | 2854 |
| REACTOME_SIGNALING_BY_MST1 | 5 | 0.634 | 1.254 | 0.203 | 0.433 | 1.000 | 2790 |
| REACTOME_PURINE_SALVAGE | 12 | 0.502 | 1.254 | 0.207 | 0.432 | 1.000 | 1328 |
| REACTOME_ONCOGENIC_MAPK_SIGNALING | 74 | 0.332 | 1.253 | 0.117 | 0.431 | 1.000 | 4416 |
| REACTOME_FOXO_MEDIATED_TRANSCRIPTION | 55 | 0.344 | 1.253 | 0.115 | 0.431 | 1.000 | 3691 |
| REACTOME_ION_HOMEOSTASIS | 32 | 0.388 | 1.252 | 0.164 | 0.432 | 1.000 | 1278 |
| REACTOME_ACROSOME_REACTION_AND_SPERM_OOCYTE_MEMBRANE_BINDING | 1 | 0.935 | 1.251 | 0.128 | 0.433 | 1.000 | 830 |
| REACTOME_G_ALPHA_I_SIGNALLING_EVENTS | 145 | 0.296 | 1.247 | 0.093 | 0.441 | 1.000 | 2186 |
| REACTOME_METABOLISM_OF_STEROID_HORMONES | 17 | 0.456 | 1.246 | 0.197 | 0.442 | 1.000 | 4118 |
| REACTOME_METABOLISM_OF_CARBOHYDRATES | 221 | 0.279 | 1.246 | 0.065 | 0.441 | 1.000 | 2581 |
| REACTOME_COMPETING_ENDOGENOUS_RNAS_CERNAS_REGULATE_PTEN_TRANSLATION | 8 | 0.546 | 1.244 | 0.228 | 0.443 | 1.000 | 431 |
| REACTOME_RUNX2_REGULATES_CHONDROCYTE_MATURATION | 3 | 0.736 | 1.243 | 0.224 | 0.445 | 1.000 | 1908 |
| REACTOME_HORMONE_LIGAND_BINDING_RECEPTORS | 1 | 0.939 | 1.243 | 0.121 | 0.445 | 1.000 | 770 |
| REACTOME_FCERI_MEDIATED_CA_2_MOBILIZATION | 23 | 0.414 | 1.238 | 0.188 | 0.454 | 1.000 | 1713 |
| REACTOME_DOWNREGULATION_OF_ERBB2_SIGNALING | 21 | 0.421 | 1.236 | 0.192 | 0.457 | 1.000 | 2078 |
| REACTOME_SIGNALING_BY_FGFR2 | 57 | 0.342 | 1.236 | 0.143 | 0.457 | 1.000 | 2357 |
| REACTOME_EARLY_PHASE_OF_HIV_LIFE_CYCLE | 13 | 0.492 | 1.234 | 0.214 | 0.459 | 1.000 | 1800 |

| | | | | | | | |
|---|---|---|---|---|---|---|---|
| REACTOME_ENOS_ACTIVATION | 10 | 0.521 | 1.233 | 0.215 | 0.460 | 1.000 | 2078 |
| REACTOME_REGULATION_OF_SIGNALING_BY_CBL | 20 | 0.422 | 1.233 | 0.191 | 0.459 | 1.000 | 1569 |
| REACTOME_INLA_MEDIATED_ENTRY_OF_LISTERIA_MONOCYTOGENES_INTO_HOST_CELLS | 9 | 0.538 | 1.229 | 0.238 | 0.468 | 1.000 | 3779 |
| REACTOME_NR1H2_AND_NR1H3_MEDIATED_SIGNALING | 37 | 0.373 | 1.228 | 0.173 | 0.468 | 1.000 | 602 |
| REACTOME_DISEASES_OF_CARBOHYDRATE_METABOLISM | 27 | 0.393 | 1.226 | 0.182 | 0.471 | 1.000 | 1984 |
| REACTOME_TBC_RABGAPS | 39 | 0.358 | 1.226 | 0.151 | 0.470 | 1.000 | 2519 |
| REACTOME_CHAPERONE_MEDIATED_AUTOPHAGY | 17 | 0.443 | 1.224 | 0.196 | 0.474 | 1.000 | 2078 |
| REACTOME_RAP1_SIGNALLING | 13 | 0.474 | 1.223 | 0.217 | 0.475 | 1.000 | 3393 |
| REACTOME_RUNX2_REGULATES_BONE_DEVELOPMENT | 23 | 0.407 | 1.223 | 0.223 | 0.474 | 1.000 | 1908 |
| REACTOME_LONG_TERM_POTENTIATION | 11 | 0.489 | 1.221 | 0.225 | 0.477 | 1.000 | 2486 |
| REACTOME_THE_CITRIC_ACID_TCA_CYCLE_AND_RESPIRATORY_ELECTRON_TRANSPORT | 155 | 0.287 | 1.221 | 0.099 | 0.477 | 1.000 | 4134 |
| REACTOME_SIGNALING_BY_RHO_GTPASES | 348 | 0.263 | 1.220 | 0.050 | 0.477 | 1.000 | 2523 |
| REACTOME_SIGNALLING_TO_RAS | 17 | 0.452 | 1.219 | 0.228 | 0.478 | 1.000 | 4574 |
| REACTOME_METABOLISM_OF_NITRIC_OXIDE_NOS3_ACTIVATION_AND_REGULATION | 13 | 0.482 | 1.218 | 0.229 | 0.481 | 1.000 | 2078 |
| REACTOME_TYROSINE_CATABOLISM | 3 | 0.749 | 1.217 | 0.226 | 0.480 | 1.000 | 3191 |
| REACTOME_EICOSANOIDS | 1 | 0.914 | 1.216 | 0.173 | 0.482 | 1.000 | 1097 |
| REACTOME_GLYCOGEN_METABOLISM | 24 | 0.411 | 1.216 | 0.184 | 0.481 | 1.000 | 1569 |
| REACTOME_LEISHMANIA_INFECTION | 122 | 0.295 | 1.214 | 0.122 | 0.484 | 1.000 | 2269 |
| REACTOME_FCGAMMA_RECEPTOR_FCGR_DEPENDENT_PHAGOCYTOSIS | 71 | 0.321 | 1.211 | 0.156 | 0.490 | 1.000 | 3912 |
| REACTOME_ERKS_ARE_INACTIVATED | 13 | 0.465 | 1.210 | 0.237 | 0.490 | 1.000 | 2574 |
| REACTOME_NEUROTOXICITY_OF_CLOSTRIDIUM_TOXINS | 6 | 0.597 | 1.209 | 0.276 | 0.491 | 1.000 | 2848 |
| REACTOME_TRAFFICKING_OF_AMPA_RECEPTORS | 18 | 0.441 | 1.209 | 0.218 | 0.492 | 1.000 | 3318 |
| REACTOME_SENESCENCE_ASSOCIATED_SECRETORY_PHENOTYPE_SASP_ | 57 | 0.340 | 1.209 | 0.175 | 0.491 | 1.000 | 3185 |
| REACTOME_RNA_POLYMERASE_III_CHAIN_ELONGATION | 18 | 0.429 | 1.208 | 0.224 | 0.491 | 1.000 | 803 |
| REACTOME_RHO_GTPASES_ACTIVATE_RHOTEKIN_AND_RHOPHILINS | 8 | 0.527 | 1.208 | 0.240 | 0.490 | 1.000 | 3484 |

| | | | | | | | |
|---|---|---|---|---|---|---|---|
| REACTOME_BIOSYNTHESIS_OF_MARESIN_LIKE_SPMS | 1 | 0.906 | 1.206 | 0.216 | 0.494 | 1.000 | 1194 |
| REACTOME_INSERTION_OF_TAIL_ANCHORED_PROTEINS_INTO_THE_ENDOPLASMIC_RETICULUM_MEMBRANE | 21 | 0.413 | 1.205 | 0.208 | 0.494 | 1.000 | 2319 |
| REACTOME_PI_3K_CASCADE_FGFR2 | 8 | 0.526 | 1.200 | 0.247 | 0.504 | 1.000 | 1020 |
| REACTOME_INTRAFLAGELLAR_TRANSPORT | 48 | 0.341 | 1.200 | 0.202 | 0.503 | 1.000 | 1710 |
| REACTOME_MET_PROMOTES_CELL_MOTILITY | 30 | 0.377 | 1.200 | 0.227 | 0.502 | 1.000 | 3654 |
| REACTOME_TRANSPORT_OF_SMALL_MOLECULES | 455 | 0.250 | 1.198 | 0.069 | 0.505 | 1.000 | 2807 |
| REACTOME_TRANSPORT_OF_NUCLEOTIDE_SUGARS | 9 | 0.509 | 1.198 | 0.239 | 0.504 | 1.000 | 3600 |
| REACTOME_AMINO_ACIDS_REGULATE_MTORC1 | 50 | 0.339 | 1.198 | 0.196 | 0.504 | 1.000 | 2587 |
| REACTOME_ANTIGEN_PROCESSING_CROSS_PRESENTATION | 82 | 0.313 | 1.197 | 0.176 | 0.505 | 1.000 | 4312 |
| REACTOME_DECTIN_2_FAMILY | 7 | 0.567 | 1.194 | 0.265 | 0.510 | 1.000 | 1022 |
| REACTOME_COMPLEX_I_BIOGENESIS | 49 | 0.339 | 1.193 | 0.206 | 0.511 | 1.000 | 4134 |
| REACTOME_NETRIN_1_SIGNALING | 36 | 0.363 | 1.193 | 0.205 | 0.510 | 1.000 | 1773 |
| REACTOME_OXIDATIVE_STRESS_INDUCED_SENESCENCE | 72 | 0.316 | 1.190 | 0.170 | 0.515 | 1.000 | 1815 |
| REACTOME_IRAK2_MEDIATED_ACTIVATION_OF_TAK1_COMPLEX | 10 | 0.503 | 1.190 | 0.254 | 0.515 | 1.000 | 1569 |
| REACTOME_REGULATION_OF_CYTOSKELETAL_REMODELING_AND_CELL_SPREADING_BY_IPP_COMPLEX_COMPONENTS | 7 | 0.549 | 1.188 | 0.269 | 0.519 | 1.000 | 4170 |
| REACTOME_INTRA_GOLGI_AND_RETROGRADE_GOLGI_TO_ER_TRAFFIC | 183 | 0.272 | 1.184 | 0.125 | 0.527 | 1.000 | 3376 |
| REACTOME_NEF_AND_SIGNAL_TRANSDUCTION | 4 | 0.648 | 1.183 | 0.282 | 0.527 | 1.000 | 3874 |
| REACTOME_NUCLEAR_SIGNALING_BY_ERBB4 | 20 | 0.419 | 1.183 | 0.236 | 0.526 | 1.000 | 3271 |
| REACTOME_ASSEMBLY_OF_ACTIVE_LPL_AND_LIPC_LIPASE_COMPLEXES | 11 | 0.492 | 1.182 | 0.271 | 0.528 | 1.000 | 1700 |
| REACTOME_CREATINE_METABOLISM | 5 | 0.608 | 1.179 | 0.292 | 0.533 | 1.000 | 774 |
| REACTOME_ERBB2_ACTIVATES_PTK6_SIGNALING | 6 | 0.578 | 1.178 | 0.294 | 0.533 | 1.000 | 606 |
| REACTOME_LYSOSPHINGOLIPID_AND_LPA_RECEPTORS | 6 | 0.574 | 1.178 | 0.269 | 0.533 | 1.000 | 1058 |
| REACTOME_REGULATION_OF_PTEN_MRNA_TRANSLATION | 9 | 0.516 | 1.178 | 0.293 | 0.532 | 1.000 | 431 |
| REACTOME_DSCAM_INTERACTIONS | 7 | 0.548 | 1.178 | 0.266 | 0.531 | 1.000 | 4122 |

| | | | | | | | |
|---|---|---|---|---|---|---|---|
| REACTOME_NUCLEAR_ENVELOPE_NE_REASSEMBLY | 68 | 0.312 | 1.175 | 0.177 | 0.537 | 1.000 | 3569 |
| REACTOME_GENE_AND_PROTEIN_EXPRESSION_BY_JAK_STAT_SIGNALING_AFTER_INTERLEUKIN_12_STIMULATION | 31 | 0.368 | 1.174 | 0.255 | 0.538 | 1.000 | 4097 |
| REACTOME_CHK1_CHK2_CDS1_MEDIATED_INACTIVATION_OF_CYCLIN_B_CDK1_COMPLEX | 13 | 0.457 | 1.173 | 0.261 | 0.538 | 1.000 | 3550 |
| REACTOME_RUNX3_REGULATES_YAP1_MEDIATED_TRANSCRIPTION | 8 | 0.517 | 1.173 | 0.275 | 0.539 | 1.000 | 1386 |
| REACTOME_MET_ACTIVATES_RAS_SIGNALING | 9 | 0.508 | 1.171 | 0.281 | 0.541 | 1.000 | 4574 |
| REACTOME_PERK_REGULATES_GENE_EXPRESSION | 31 | 0.364 | 1.169 | 0.254 | 0.545 | 1.000 | 2229 |
| REACTOME_SUMO_IS_PROTEOLYTICALLY_PROCESSED | 6 | 0.561 | 1.168 | 0.306 | 0.546 | 1.000 | 4031 |
| REACTOME_P75NTR_RECRUITS_SIGNALLING_COMPLEXES | 12 | 0.454 | 1.168 | 0.258 | 0.545 | 1.000 | 1569 |
| REACTOME_ACTIVATION_OF_MATRIX_METALLOPROTEINASES | 14 | 0.451 | 1.168 | 0.268 | 0.544 | 1.000 | 1119 |
| REACTOME_NF_KB_IS_ACTIVATED_AND_SIGNALS_SURVIVAL | 12 | 0.467 | 1.167 | 0.285 | 0.546 | 1.000 | 1569 |
| REACTOME_PROTEIN_FOLDING | 75 | 0.309 | 1.166 | 0.198 | 0.547 | 1.000 | 2421 |
| REACTOME_SIGNALING_BY_WNT_IN_CANCER | 30 | 0.371 | 1.163 | 0.243 | 0.553 | 1.000 | 3626 |
| REACTOME_ATTACHMENT_OF_GPI_ANCHOR_TO_UPAR | 7 | 0.547 | 1.162 | 0.287 | 0.553 | 1.000 | 837 |
| REACTOME_FGFR2_LIGAND_BINDING_AND_ACTIVATION | 5 | 0.598 | 1.161 | 0.306 | 0.554 | 1.000 | 1506 |
| REACTOME_ACTIVATED_NTRK3_SIGNALS_THROUGH_RAS | 7 | 0.543 | 1.160 | 0.268 | 0.555 | 1.000 | 4574 |
| REACTOME_ARYL_HYDROCARBON_RECEPTOR_SIGNALLING | 6 | 0.556 | 1.159 | 0.291 | 0.556 | 1.000 | 1690 |
| REACTOME_ESTROGEN_STIMULATED_SIGNALING_THROUGH_PRKCZ | 6 | 0.569 | 1.159 | 0.300 | 0.556 | 1.000 | 4234 |
| REACTOME_TRANSPORT_OF_VITAMINS_NUCLEOSIDES_AND_RELATED_MOLECULES | 28 | 0.378 | 1.159 | 0.249 | 0.555 | 1.000 | 1623 |
| REACTOME_CARGO_RECOGNITION_FOR_CLATHRIN_MEDIATED_ENDOCYTOSIS | 78 | 0.303 | 1.159 | 0.194 | 0.554 | 1.000 | 3583 |
| REACTOME_SIGNALING_BY_NTRK3_TRKC_ | 15 | 0.437 | 1.158 | 0.280 | 0.554 | 1.000 | 1901 |
| REACTOME_SIGNALING_BY_TGF_BETA_RECEPTOR_COMPLEX | 72 | 0.309 | 1.157 | 0.211 | 0.555 | 1.000 | 3642 |
| REACTOME_SUPPRESSION_OF_APOPTOSIS | 6 | 0.569 | 1.156 | 0.310 | 0.558 | 1.000 | 4771 |
| REACTOME_THROMBIN_SIGNALLING_THROUGH_PROTEINASE_ACTIVATED_RECEPTORS_PARS_ | 19 | 0.415 | 1.152 | 0.277 | 0.566 | 1.000 | 272 |

| | | | | | | | |
|---|---|---|---|---|---|---|---|
| REACTOME_GLUCOCORTICOID_BIOSYNTHESIS | 2 | 0.756 | 1.148 | 0.319 | 0.574 | 1.000 | 2234 |
| REACTOME_MUCOPOLYSACCHARIDOSES | 11 | 0.463 | 1.147 | 0.294 | 0.575 | 1.000 | 1984 |
| REACTOME_DOWNREGULATION_OF_ERBB2_ERBB3_SIGNALING | 11 | 0.463 | 1.147 | 0.291 | 0.574 | 1.000 | 3779 |
| REACTOME_RHO_GTPASES_ACTIVATE_KTN1 | 11 | 0.469 | 1.146 | 0.284 | 0.575 | 1.000 | 3390 |
| REACTOME_INTERLEUKIN_12_SIGNALING | 36 | 0.347 | 1.146 | 0.242 | 0.574 | 1.000 | 4097 |
| REACTOME_SEMA4D_INDUCED_CELL_MIGRATION_AND_GROWTH_CONE_COLLAPSE | 19 | 0.400 | 1.141 | 0.293 | 0.585 | 1.000 | 3949 |
| REACTOME_PI3K_EVENTS_IN_ERBB4_SIGNALING | 5 | 0.588 | 1.138 | 0.349 | 0.589 | 1.000 | 606 |
| REACTOME_PHOSPHOLIPASE_C_MEDIATED_CASCADE_FGFR4 | 5 | 0.592 | 1.138 | 0.331 | 0.590 | 1.000 | 1020 |
| REACTOME_MODULATION_BY_MTB_OF_HOST_IMMUNE_SYSTEM | 6 | 0.563 | 1.136 | 0.338 | 0.593 | 1.000 | 5168 |
| REACTOME_2_LTR_CIRCLE_FORMATION | 7 | 0.530 | 1.136 | 0.327 | 0.592 | 1.000 | 4837 |
| REACTOME_PI_3K_CASCADE_FGFR4 | 9 | 0.496 | 1.135 | 0.325 | 0.593 | 1.000 | 1020 |
| REACTOME_SIGNALING_BY_NOTCH2 | 28 | 0.368 | 1.134 | 0.290 | 0.592 | 1.000 | 1569 |
| REACTOME_PI3K_EVENTS_IN_ERBB2_SIGNALING | 8 | 0.501 | 1.133 | 0.301 | 0.594 | 1.000 | 606 |
| REACTOME_GOLGI_CISTERNAE_PERICENTRIOLAR_STACK_REORGANIZATION | 14 | 0.446 | 1.133 | 0.286 | 0.593 | 1.000 | 4246 |
| REACTOME_SIGNALING_BY_ERBB2 | 41 | 0.340 | 1.133 | 0.260 | 0.592 | 1.000 | 2100 |
| REACTOME_SMAD2_SMAD3_SMAD4_HETEROTRIMER_REGULATES_TRANSCRIPTION | 31 | 0.355 | 1.133 | 0.272 | 0.592 | 1.000 | 1569 |
| REACTOME_TRANSPORT_OF_FATTY_ACIDS | 2 | 0.759 | 1.129 | 0.330 | 0.598 | 1.000 | 257 |
| REACTOME_FGFR1B_LIGAND_BINDING_AND_ACTIVATION | 2 | 0.766 | 1.128 | 0.341 | 0.599 | 1.000 | 720 |
| REACTOME_FGFR2C_LIGAND_BINDING_AND_ACTIVATION | 2 | 0.757 | 1.127 | 0.326 | 0.601 | 1.000 | 1020 |
| REACTOME_BIOSYNTHESIS_OF_MARESINS | 2 | 0.750 | 1.124 | 0.325 | 0.607 | 1.000 | 3178 |
| REACTOME_FGFR1_LIGAND_BINDING_AND_ACTIVATION | 3 | 0.669 | 1.124 | 0.371 | 0.606 | 1.000 | 720 |
| REACTOME_TFAP2_AP_2_FAMILY_REGULATES_TRANSCRIPTION_OF_CELL_CYCLE_FACTORS | 5 | 0.571 | 1.123 | 0.364 | 0.607 | 1.000 | 1059 |
| REACTOME_ACTIVATION_OF_RAC1 | 10 | 0.477 | 1.123 | 0.333 | 0.607 | 1.000 | 3874 |
| REACTOME_PHOSPHOLIPASE_C_MEDIATED_CASCADE_FGFR2 | 4 | 0.625 | 1.121 | 0.352 | 0.609 | 1.000 | 1020 |

| | | | | | | | |
|---|---|---|---|---|---|---|---|
| REACTOME_PROTEIN_REPAIR | 6 | 0.560 | 1.119 | 0.368 | 0.613 | 1.000 | 5417 |
| REACTOME_CIRCADIAN_CLOCK | 67 | 0.300 | 1.119 | 0.260 | 0.613 | 1.000 | 1886 |
| REACTOME_SIGNALING_BY_TYPE_1_INSULIN_LIKE_GROWTH_FACTOR_1_RECEPTOR_IGF1R_ | 31 | 0.348 | 1.118 | 0.300 | 0.614 | 1.000 | 1544 |
| REACTOME_INTERLEUKIN_9_SIGNALING | 5 | 0.586 | 1.117 | 0.357 | 0.615 | 1.000 | 3707 |
| REACTOME_P2Y_RECEPTORS | 3 | 0.657 | 1.117 | 0.367 | 0.615 | 1.000 | 456 |
| REACTOME_ACTIVATION_OF_BH3_ONLY_PROTEINS | 29 | 0.355 | 1.114 | 0.336 | 0.621 | 1.000 | 1715 |
| REACTOME_RUNX2_REGULATES_OSTEOBLAST_DIFFERENTIATION | 18 | 0.399 | 1.113 | 0.309 | 0.621 | 1.000 | 1908 |
| REACTOME_DOWNSTREAM_SIGNALING_EVENTS_OF_B_CELL_RECEPTOR_BCR_ | 76 | 0.295 | 1.113 | 0.275 | 0.620 | 1.000 | 4312 |
| REACTOME_STAT5_ACTIVATION | 6 | 0.546 | 1.111 | 0.357 | 0.623 | 1.000 | 2908 |
| REACTOME_BETA_OXIDATION_OF_HEXANOYL_COA_TO_BUTANOYL_COA | 5 | 0.570 | 1.110 | 0.373 | 0.625 | 1.000 | 4043 |
| REACTOME_ATF4_ACTIVATES_GENES_IN_RESPONSE_TO_ENDOPLASMIC_RETICULUM_STRESS | 26 | 0.365 | 1.109 | 0.320 | 0.627 | 1.000 | 1951 |
| REACTOME_NOTCH1_INTRACELLULAR_DOMAIN_REGULATES_TRANSCRIPTION | 45 | 0.322 | 1.108 | 0.313 | 0.628 | 1.000 | 3349 |
| REACTOME_INTRINSIC_PATHWAY_FOR_APOPTOSIS | 51 | 0.313 | 1.106 | 0.289 | 0.631 | 1.000 | 1851 |
| REACTOME_METABOLIC_DISORDERS_OF_BIOLOGICAL_OXIDATION_ENZYMES | 21 | 0.376 | 1.106 | 0.323 | 0.630 | 1.000 | 1516 |
| REACTOME_OTHER_INTERLEUKIN_SIGNALING | 16 | 0.409 | 1.104 | 0.326 | 0.632 | 1.000 | 1545 |
| REACTOME_REDUCTION_OF_CYTOSOLIC_CA_LEVELS | 7 | 0.511 | 1.103 | 0.357 | 0.634 | 1.000 | 852 |
| REACTOME_THE_PHOTOTRANSDUCTION_CASCADE | 15 | 0.413 | 1.103 | 0.307 | 0.633 | 1.000 | 1611 |
| REACTOME_ESTROGEN_DEPENDENT_GENE_EXPRESSION | 93 | 0.282 | 1.102 | 0.262 | 0.635 | 1.000 | 2911 |
| REACTOME_TERMINATION_OF_O_GLYCAN_BIOSYNTHESIS | 8 | 0.499 | 1.100 | 0.365 | 0.637 | 1.000 | 3303 |
| REACTOME_SIGNALING_BY_FGFR_IN_DISEASE | 48 | 0.312 | 1.100 | 0.318 | 0.637 | 1.000 | 4894 |
| REACTOME_SPHINGOLIPID_DE_NOVO_BIOSYNTHESIS | 35 | 0.337 | 1.098 | 0.332 | 0.640 | 1.000 | 3892 |
| REACTOME_NEF_MEDIATED_CD4_DOWN_REGULATION | 9 | 0.475 | 1.097 | 0.363 | 0.641 | 1.000 | 2954 |
| REACTOME_SIGNALING_BY_ACTIVIN | 10 | 0.457 | 1.097 | 0.361 | 0.641 | 1.000 | 1604 |
| REACTOME_CA2_ACTIVATED_K_CHANNELS | 3 | 0.641 | 1.096 | 0.414 | 0.643 | 1.000 | 2209 |

| | | | | | | | |
|---|---|---|---|---|---|---|---|
| REACTOME_CALNEXIN_CALRETICULIN_CYCLE | 26 | 0.353 | 1.096 | 0.308 | 0.642 | 1.000 | 4705 |
| REACTOME_GRB2_EVENTS_IN_ERBB2_SIGNALING | 9 | 0.477 | 1.095 | 0.341 | 0.642 | 1.000 | 606 |
| REACTOME_CELLULAR_SENESCENCE | 134 | 0.260 | 1.095 | 0.269 | 0.641 | 1.000 | 1815 |
| REACTOME_ANDROGEN_BIOSYNTHESIS | 4 | 0.606 | 1.095 | 0.404 | 0.640 | 1.000 | 3601 |
| REACTOME_MAP3K8_TPL2_DEPENDENT_MAPK1_3_ACTIVATION | 16 | 0.403 | 1.092 | 0.336 | 0.647 | 1.000 | 2850 |
| REACTOME_SIGNALING_BY_ERYTHROPOIETIN | 21 | 0.380 | 1.088 | 0.345 | 0.653 | 1.000 | 3480 |
| REACTOME_VITAMINS | 3 | 0.639 | 1.088 | 0.402 | 0.653 | 1.000 | 1273 |
| REACTOME_IRAK1_RECRUITS_IKK_COMPLEX | 14 | 0.426 | 1.088 | 0.384 | 0.651 | 1.000 | 1722 |
| REACTOME_ER_QUALITY_CONTROL_COMPARTMENT_ERQC_ | 21 | 0.381 | 1.088 | 0.359 | 0.651 | 1.000 | 4133 |
| REACTOME_PREGNENOLONE_BIOSYNTHESIS | 9 | 0.480 | 1.087 | 0.385 | 0.652 | 1.000 | 4118 |
| REACTOME_PEXOPHAGY | 11 | 0.450 | 1.086 | 0.378 | 0.652 | 1.000 | 1569 |
| REACTOME_INTERLEUKIN_27_SIGNALING | 8 | 0.489 | 1.086 | 0.381 | 0.651 | 1.000 | 3877 |
| REACTOME_INHIBITION_OF_DNA_RECOMBINATION_AT_TELOMERE | 30 | 0.339 | 1.085 | 0.321 | 0.653 | 1.000 | 3185 |
| REACTOME_EPH_EPHRIN_MEDIATED_REPULSION_OF_CELLS | 42 | 0.322 | 1.085 | 0.347 | 0.652 | 1.000 | 1115 |
| REACTOME_TRANSLATION_OF_REPLICASE_AND_ASSEMBLY_OF_THE_REPLICATION_TRANSCRIPTION_COMPLEX | 13 | 0.430 | 1.084 | 0.385 | 0.654 | 1.000 | 2473 |
| REACTOME_INTERLEUKIN_35_SIGNALLING | 8 | 0.489 | 1.082 | 0.381 | 0.656 | 1.000 | 3877 |
| REACTOME_CASPASE_ACTIVATION_VIA_DEPENDENCE_RECEPTORS_IN_THE_ABSENCE_OF_LIGAND | 7 | 0.503 | 1.082 | 0.402 | 0.655 | 1.000 | 4049 |
| REACTOME_SIGNALING_BY_TGFB_FAMILY_MEMBERS | 86 | 0.277 | 1.081 | 0.313 | 0.656 | 1.000 | 1858 |
| REACTOME_PHENYLALANINE_METABOLISM | 4 | 0.595 | 1.081 | 0.423 | 0.655 | 1.000 | 1805 |
| REACTOME_P75NTR_SIGNALS_VIA_NF_KB | 15 | 0.404 | 1.080 | 0.373 | 0.656 | 1.000 | 1569 |
| REACTOME_PHASE_II_CONJUGATION_OF_COMPOUNDS | 51 | 0.304 | 1.080 | 0.324 | 0.657 | 1.000 | 2923 |
| REACTOME_STIMULI_SENSING_CHANNELS | 57 | 0.297 | 1.078 | 0.346 | 0.660 | 1.000 | 1721 |
| REACTOME_MET_INTERACTS_WITH_TNS_PROTEINS | 4 | 0.595 | 1.078 | 0.398 | 0.659 | 1.000 | 1771 |
| REACTOME_PI_3K_CASCADE_FGFR3 | 8 | 0.487 | 1.075 | 0.375 | 0.664 | 1.000 | 1136 |

| | | | | | | | |
|---|---|---|---|---|---|---|---|
| REACTOME_ACTIVATION_OF_RAS_IN_B_CELLS | 3 | 0.633 | 1.075 | 0.425 | 0.664 | 1.000 | 3953 |
| REACTOME_DETOXIFICATION_OF_REACTIVE_OXYGEN_SPECIES | 27 | 0.346 | 1.074 | 0.336 | 0.663 | 1.000 | 4653 |
| REACTOME_RUNX3_REGULATES_P14_ARF | 10 | 0.455 | 1.074 | 0.386 | 0.662 | 1.000 | 3322 |
| REACTOME_HOST_INTERACTIONS_OF_HIV_FACTORS | 123 | 0.259 | 1.074 | 0.320 | 0.662 | 1.000 | 4312 |
| REACTOME_MITOCHONDRIAL_FATTY_ACID_BETA_OXIDATION_OF_SATURATED_FATTY_ACIDS | 8 | 0.471 | 1.073 | 0.397 | 0.662 | 1.000 | 4043 |
| REACTOME_SIGNALING_BY_KIT_IN_DISEASE | 19 | 0.382 | 1.073 | 0.385 | 0.662 | 1.000 | 3953 |
| REACTOME_SIGNALING_BY_NOTCH1_HD_DOMAIN_MUTANTS_IN_CANCER | 14 | 0.417 | 1.073 | 0.379 | 0.661 | 1.000 | 1569 |
| REACTOME_DEFENSINS | 3 | 0.646 | 1.072 | 0.426 | 0.662 | 1.000 | 1036 |
| REACTOME_PLATELET_CALCIUM_HOMEOSTASIS | 17 | 0.380 | 1.072 | 0.352 | 0.662 | 1.000 | 1975 |
| REACTOME_LISTERIA_MONOCYTOGENES_ENTRY_INTO_HOST_CELLS | 18 | 0.384 | 1.069 | 0.360 | 0.667 | 1.000 | 3779 |
| REACTOME_TRANSCRIPTIONAL_REGULATION_BY_THE_AP_2_TFAP2_FAMILY_OF_TRANSCRIPTION_FACTORS | 28 | 0.348 | 1.069 | 0.367 | 0.666 | 1.000 | 2775 |
| REACTOME_INTRACELLULAR_SIGNALING_BY_SECOND_MESSENGERS | 247 | 0.238 | 1.067 | 0.288 | 0.669 | 1.000 | 1351 |
| REACTOME_METABOLISM_OF_AMINO_ACIDS_AND_DERIVATIVES | 292 | 0.236 | 1.066 | 0.286 | 0.670 | 1.000 | 4025 |
| REACTOME_VEGFR2_MEDIATED_VASCULAR_PERMEABILITY | 23 | 0.358 | 1.066 | 0.371 | 0.669 | 1.000 | 3943 |
| REACTOME_ALPHA_DEFENSINS | 3 | 0.646 | 1.065 | 0.441 | 0.671 | 1.000 | 1036 |
| REACTOME_TRANSCRIPTIONAL_REGULATION_BY_RUNX1 | 162 | 0.249 | 1.064 | 0.320 | 0.671 | 1.000 | 3525 |
| REACTOME_THE_ROLE_OF_NEF_IN_HIV_1_REPLICATION_AND_DISEASE_PATHOGENESIS | 22 | 0.369 | 1.063 | 0.389 | 0.674 | 1.000 | 2954 |
| REACTOME_INTERLEUKIN_RECEPTOR_SHC_SIGNALING | 15 | 0.395 | 1.063 | 0.372 | 0.673 | 1.000 | 3707 |
| REACTOME_BUDDING_AND_MATURATION_OF_HIV_VIRION | 28 | 0.342 | 1.062 | 0.366 | 0.672 | 1.000 | 4066 |
| REACTOME_MITOCHONDRIAL_FATTY_ACID_BETA_OXIDATION_OF_UNSATURATED_FATTY_ACIDS | 5 | 0.541 | 1.062 | 0.410 | 0.671 | 1.000 | 5531 |
| REACTOME_LYSOSOME_VESICLE_BIOGENESIS | 31 | 0.334 | 1.062 | 0.372 | 0.670 | 1.000 | 2475 |
| REACTOME_TRIF_MEDIATED_PROGRAMMED_CELL_DEATH | 7 | 0.498 | 1.062 | 0.390 | 0.669 | 1.000 | 317 |
| REACTOME_NEGATIVE_REGULATION_OF_MET_ACTIVITY | 18 | 0.371 | 1.059 | 0.353 | 0.676 | 1.000 | 4574 |

| | | | | | | | |
|---|---|---|---|---|---|---|---|
| REACTOME_G_ALPHA_Q_SIGNALLING_EVENTS | 87 | 0.273 | 1.058 | 0.348 | 0.676 | 1.000 | 1412 |
| REACTOME_PI5P_REGULATES_TP53_ACETYLATION | 9 | 0.458 | 1.057 | 0.413 | 0.677 | 1.000 | 4119 |
| REACTOME_TRP_CHANNELS | 13 | 0.416 | 1.056 | 0.410 | 0.679 | 1.000 | 2000 |
| REACTOME_ERYTHROCYTES_TAKE_UP_OXYGEN_AND_RELEASE_CARBON_DIOXIDE | 2 | 0.687 | 1.056 | 0.431 | 0.678 | 1.000 | 1529 |
| REACTOME_TRANSCRIPTIONAL_REGULATION_BY_RUNX3 | 92 | 0.265 | 1.055 | 0.337 | 0.680 | 1.000 | 4024 |
| REACTOME_RHO_GTPASE_CYCLE | 117 | 0.259 | 1.054 | 0.354 | 0.681 | 1.000 | 1663 |
| REACTOME_TRANSCRIPTIONAL_ACTIVITY_OF_SMAD2_SMAD3_SMAD4_HETEROTRIMER | 43 | 0.303 | 1.053 | 0.369 | 0.681 | 1.000 | 1799 |
| REACTOME_PROSTACYCLIN_SIGNALLING_THROUGH_PROSTACYCLIN_RECEPTOR | 8 | 0.474 | 1.053 | 0.404 | 0.680 | 1.000 | 2180 |
| REACTOME_ACTIVATION_OF_KAINATE_RECEPTORS_UPON_GLUTAMATE_BINDING | 15 | 0.400 | 1.052 | 0.382 | 0.681 | 1.000 | 2486 |
| REACTOME_GLUCAGON_LIKE_PEPTIDE_1_GLP1_REGULATES_INSULIN_SECRETION | 22 | 0.361 | 1.052 | 0.369 | 0.681 | 1.000 | 2180 |
| REACTOME_ERBB2_REGULATES_CELL_MOTILITY | 8 | 0.479 | 1.051 | 0.430 | 0.681 | 1.000 | 606 |
| REACTOME_INSULIN_PROCESSING | 22 | 0.353 | 1.050 | 0.404 | 0.682 | 1.000 | 3910 |
| REACTOME_ACYL_CHAIN_REMODELLING_OF_PG | 6 | 0.508 | 1.050 | 0.432 | 0.682 | 1.000 | 231 |
| REACTOME_AMYLOID_FIBER_FORMATION | 42 | 0.312 | 1.049 | 0.376 | 0.682 | 1.000 | 2748 |
| REACTOME_ACTIVATED_NTRK2_SIGNALS_THROUGH_CDK5 | 5 | 0.522 | 1.049 | 0.444 | 0.682 | 1.000 | 2378 |
| REACTOME_SIGNALING_BY_THE_B_CELL_RECEPTOR_BCR_ | 97 | 0.262 | 1.046 | 0.354 | 0.687 | 1.000 | 4312 |
| REACTOME_CELL_CELL_JUNCTION_ORGANIZATION | 35 | 0.318 | 1.046 | 0.380 | 0.686 | 1.000 | 964 |
| REACTOME_NEF_MEDIATES_DOWN_MODULATION_OF_CELL_SURFACE_RECEPTORS_BY_RECRUITING_THEM_TO_CLATHRIN_ADAPTERS | 19 | 0.373 | 1.045 | 0.392 | 0.687 | 1.000 | 2954 |
| REACTOME_DEFECTIVE_GALNT3_CAUSES_FAMILIAL_HYPERPHOSPHATEMIC_TUMORAL_CALCINOSIS_HFTC_ | 4 | 0.583 | 1.045 | 0.462 | 0.687 | 1.000 | 1022 |
| REACTOME_ABACAVIR_METABOLISM | 4 | 0.573 | 1.045 | 0.454 | 0.686 | 1.000 | 3240 |
| REACTOME_INTERLEUKIN_20_FAMILY_SIGNALING | 16 | 0.376 | 1.045 | 0.385 | 0.685 | 1.000 | 414 |
| REACTOME_MTORC1_MEDIATED_SIGNALLING | 23 | 0.354 | 1.041 | 0.403 | 0.692 | 1.000 | 2367 |

| | | | | | | | |
|---|---|---|---|---|---|---|---|
| REACTOME_E3_UBIQUITIN_LIGASES_UBIQUITINATE_TARGET_PROTEINS | 45 | 0.297 | 1.039 | 0.371 | 0.695 | 1.000 | 2672 |
| REACTOME_VASOPRESSIN_REGULATES_RENAL_WATER_HOMEOSTASIS_VIA_AQUAPORINS | 21 | 0.358 | 1.039 | 0.400 | 0.694 | 1.000 | 2182 |
| REACTOME_ADORA2B_MEDIATED_ANTI_INFLAMMATORY_CYTOKINES_PRODUCTION | 37 | 0.311 | 1.039 | 0.404 | 0.693 | 1.000 | 2269 |
| REACTOME_VXPX_CARGO_TARGETING_TO_CILIUM | 17 | 0.378 | 1.038 | 0.411 | 0.695 | 1.000 | 3223 |
| REACTOME_PHASE_1_INACTIVATION_OF_FAST_NA_CHANNELS | 3 | 0.624 | 1.037 | 0.475 | 0.696 | 1.000 | 332 |
| REACTOME_O_LINKED_GLYCOSYLATION | 61 | 0.281 | 1.034 | 0.378 | 0.703 | 1.000 | 1630 |
| REACTOME_DIGESTION_OF_DIETARY_LIPID | 1 | 0.776 | 1.034 | 0.447 | 0.702 | 1.000 | 2844 |
| REACTOME_DISEASES_ASSOCIATED_WITH_SURFACTANT_METABOLISM | 1 | 0.784 | 1.032 | 0.454 | 0.705 | 1.000 | 2740 |
| REACTOME_MECP2_REGULATES_TRANSCRIPTION_FACTORS | 3 | 0.615 | 1.032 | 0.489 | 0.705 | 1.000 | 4899 |
| REACTOME_ROBO_RECEPTORS_BIND_AKAP5 | 7 | 0.480 | 1.030 | 0.438 | 0.707 | 1.000 | 1610 |
| REACTOME_REGULATION_OF_RUNX1_EXPRESSION_AND_ACTIVITY | 16 | 0.384 | 1.029 | 0.422 | 0.708 | 1.000 | 1908 |
| REACTOME_ROLE_OF_LAT2_NTAL_LAB_ON_CALCIUM_MOBILIZATION | 13 | 0.411 | 1.029 | 0.433 | 0.708 | 1.000 | 816 |
| REACTOME_RHO_GTPASES_ACTIVATE_FORMINS | 129 | 0.248 | 1.029 | 0.396 | 0.707 | 1.000 | 2493 |
| REACTOME_DOPAMINE_NEUROTRANSMITTER_RELEASE_CYCLE | 14 | 0.393 | 1.027 | 0.424 | 0.709 | 1.000 | 3096 |
| REACTOME_IRS_MEDIATED_SIGNALLING | 29 | 0.324 | 1.022 | 0.408 | 0.720 | 1.000 | 1544 |
| REACTOME_ARMS_MEDIATED_ACTIVATION | 5 | 0.515 | 1.022 | 0.453 | 0.719 | 1.000 | 4322 |
| REACTOME_CYTOCHROME_C_MEDIATED_APOPTOTIC_RESPONSE | 13 | 0.400 | 1.021 | 0.415 | 0.721 | 1.000 | 1851 |
| REACTOME_CELLULAR_RESPONSE_TO_HEAT_STRESS | 93 | 0.259 | 1.021 | 0.401 | 0.720 | 1.000 | 3960 |
| REACTOME_ACTIVATION_OF_NIMA_KINASES_NEK9_NEK6_NEK7 | 7 | 0.474 | 1.021 | 0.451 | 0.719 | 1.000 | 3550 |
| REACTOME_TERMINAL_PATHWAY_OF_COMPLEMENT | 2 | 0.663 | 1.019 | 0.483 | 0.721 | 1.000 | 1785 |
| REACTOME_G_ALPHA_12_13_SIGNALLING_EVENTS | 60 | 0.278 | 1.019 | 0.413 | 0.720 | 1.000 | 2238 |
| REACTOME_TETRAHYDROBIOPTERIN_BH4_SYNTHESIS_RECYCLING_SALVAGE_AND_REGULATION | 9 | 0.448 | 1.018 | 0.442 | 0.723 | 1.000 | 2078 |

| | | | | | | | |
|---|---|---|---|---|---|---|---|
| REACTOME_PHOSPHATE_BOND_HYDROLYSIS_BY_NTPDASE_PROTEINS | 6 | 0.480 | 1.016 | 0.448 | 0.725 | 1.000 | 1855 |
| REACTOME_DOWNSTREAM_SIGNAL_TRANSDUCTION | 28 | 0.329 | 1.016 | 0.438 | 0.724 | 1.000 | 4150 |
| REACTOME_SIGNALING_BY_BRAF_AND_RAF_FUSIONS | 57 | 0.279 | 1.016 | 0.409 | 0.723 | 1.000 | 897 |
| REACTOME_C_TYPE_LECTIN_RECEPTORS_CLRS_ | 112 | 0.250 | 1.014 | 0.414 | 0.726 | 1.000 | 3985 |
| REACTOME_WAX_AND_PLASMALOGEN_BIOSYNTHESIS | 4 | 0.547 | 1.012 | 0.492 | 0.729 | 1.000 | 793 |
| REACTOME_GLYCOLYSIS | 62 | 0.276 | 1.012 | 0.412 | 0.729 | 1.000 | 2666 |
| REACTOME_GLYCOGEN_BREAKDOWN_GLYCOGENOLYSIS_ | 15 | 0.384 | 1.012 | 0.417 | 0.729 | 1.000 | 407 |
| REACTOME_G_BETA_GAMMA_SIGNALLING_THROUGH_CDC42 | 9 | 0.441 | 1.011 | 0.434 | 0.728 | 1.000 | 2180 |
| REACTOME_SIGNALING_BY_NOTCH1_PEST_DOMAIN_MUTANTS_IN_CANCER | 56 | 0.278 | 1.011 | 0.430 | 0.728 | 1.000 | 3349 |
| REACTOME_RAS_PROCESSING | 23 | 0.339 | 1.011 | 0.435 | 0.728 | 1.000 | 4173 |
| REACTOME_LRR_FLII_INTERACTING_PROTEIN_1_LRRFIP1_ACTIVATES_TYPE_I_IFN_PRODUCTION | 5 | 0.512 | 1.010 | 0.473 | 0.727 | 1.000 | 5361 |
| REACTOME_GABA_B_RECEPTOR_ACTIVATION | 19 | 0.361 | 1.010 | 0.451 | 0.726 | 1.000 | 2182 |
| REACTOME_CARGO_TRAFFICKING_TO_THE_PERICILIARY_MEMBRANE | 43 | 0.290 | 1.007 | 0.451 | 0.734 | 1.000 | 3223 |
| REACTOME_RNA_POLYMERASE_III_TRANSCRIPTION_INITIATION_FROM_TYPE_3_PROMOTER | 28 | 0.321 | 1.006 | 0.437 | 0.733 | 1.000 | 1350 |
| REACTOME_PROSTANOID_LIGAND_RECEPTORS | 1 | 0.755 | 1.005 | 0.486 | 0.736 | 1.000 | 3119 |
| REACTOME_GLUCAGON_TYPE_LIGAND_RECEPTORS | 11 | 0.399 | 1.002 | 0.471 | 0.740 | 1.000 | 2180 |
| REACTOME_HCMV_EARLY_EVENTS | 72 | 0.271 | 1.002 | 0.457 | 0.740 | 1.000 | 2166 |
| REACTOME_SIGNALING_BY_NODAL | 12 | 0.408 | 1.002 | 0.466 | 0.739 | 1.000 | 1248 |
| REACTOME_CONSTITUTIVE_SIGNALING_BY_LIGAND_RESPONSIVE_EGFR_CANCER_VARIANTS | 16 | 0.368 | 1.001 | 0.453 | 0.739 | 1.000 | 2078 |
| REACTOME_PHOSPHATE_BOND_HYDROLYSIS_BY_NUDT_PROTEINS | 7 | 0.463 | 1.000 | 0.472 | 0.740 | 1.000 | 361 |
| REACTOME_GLYCOGEN_STORAGE_DISEASES | 12 | 0.411 | 1.000 | 0.470 | 0.740 | 1.000 | 1569 |
| REACTOME_SIGNALING_BY_ERBB2_IN_CANCER | 18 | 0.367 | 1.000 | 0.454 | 0.740 | 1.000 | 2078 |
| REACTOME_MITOPHAGY | 28 | 0.326 | 0.999 | 0.483 | 0.741 | 1.000 | 3779 |
| REACTOME_PEPTIDE_HORMONE_METABOLISM | 44 | 0.288 | 0.997 | 0.445 | 0.744 | 1.000 | 3910 |

| | | | | | | | |
|---|---|---|---|---|---|---|---|
| REACTOME_G_BETA_GAMMA_SIGNALLING_THROUGH_PI3KGAMMA | 12 | 0.406 | 0.996 | 0.467 | 0.745 | 1.000 | 3691 |
| REACTOME_TRAFFICKING_OF_MYRISTOYLATED_PROTEINS_TO_THE_CILIUM | 4 | 0.556 | 0.995 | 0.511 | 0.746 | 1.000 | 2792 |
| REACTOME_ASPARTATE_AND_ASPARAGINE_METABOLISM | 6 | 0.482 | 0.995 | 0.481 | 0.745 | 1.000 | 104 |
| REACTOME_SYNTHESIS_OF_IP2_IP_AND_INS_IN_THE_CYTOSOL | 11 | 0.414 | 0.995 | 0.438 | 0.745 | 1.000 | 3126 |
| REACTOME_SIGNALING_BY_SCF_KIT | 38 | 0.299 | 0.993 | 0.454 | 0.747 | 1.000 | 3953 |
| REACTOME_FCERI_MEDIATED_NF_KB_ACTIVATION | 73 | 0.264 | 0.992 | 0.461 | 0.749 | 1.000 | 4312 |
| REACTOME_PROTEIN_UBIQUITINATION | 65 | 0.269 | 0.991 | 0.470 | 0.749 | 1.000 | 4677 |
| REACTOME_RUNX1_REGULATES_TRANSCRIPTION_OF_GENES_INVOLVED_IN_INTERLEUKIN_SIGNALING | 3 | 0.594 | 0.991 | 0.530 | 0.749 | 1.000 | 1908 |
| REACTOME_PROTON_COUPLED_MONOCARBOXYLATE_TRANSPORT | 3 | 0.586 | 0.990 | 0.534 | 0.750 | 1.000 | 4862 |
| REACTOME_INTERACTIONS_OF_VPR_WITH_HOST_CELLULAR_PROTEINS | 37 | 0.298 | 0.987 | 0.490 | 0.757 | 1.000 | 2493 |
| REACTOME_SIGNALING_BY_FGFR2_IIIA_TM | 16 | 0.363 | 0.984 | 0.480 | 0.761 | 1.000 | 2357 |
| REACTOME_PTK6_REGULATES_PROTEINS_INVOLVED_IN_RNA_PROCESSING | 4 | 0.535 | 0.984 | 0.518 | 0.760 | 1.000 | 5907 |
| REACTOME_TGF_BETA_RECEPTOR_SIGNALING_IN_EMT_EPITHELIAL_TO_MESENCHYMAL_TRANSITION_ | 16 | 0.370 | 0.984 | 0.496 | 0.760 | 1.000 | 4568 |
| REACTOME_HIV_INFECTION | 218 | 0.220 | 0.984 | 0.504 | 0.759 | 1.000 | 4215 |
| REACTOME_GLUTATHIONE_CONJUGATION | 22 | 0.334 | 0.982 | 0.480 | 0.762 | 1.000 | 2897 |
| REACTOME_NOD1_2_SIGNALING_PATHWAY | 35 | 0.300 | 0.982 | 0.488 | 0.761 | 1.000 | 1873 |
| REACTOME_TRANSPORT_OF_NUCLEOSIDES_AND_FREE_PURINE_AND_PYRIMIDINE_BASES_ACROSS_THE_PLASMA_MEMBRANE | 9 | 0.420 | 0.982 | 0.509 | 0.761 | 1.000 | 1623 |
| REACTOME_FACTORS_INVOLVED_IN_MEGAKARYOCYTE_DEVELOPMENT_AND_PLATELET_PRODUCTION | 117 | 0.237 | 0.975 | 0.524 | 0.774 | 1.000 | 2543 |
| REACTOME_CHOLINE_CATABOLISM | 4 | 0.537 | 0.975 | 0.535 | 0.774 | 1.000 | 588 |
| REACTOME_HDMS_DEMETHYLATE_HISTONES | 21 | 0.341 | 0.974 | 0.508 | 0.775 | 1.000 | 3618 |

| | | | | | | | |
|---|---|---|---|---|---|---|---|
| REACTOME_SOS_MEDIATED_SIGNALLING | 7 | 0.456 | 0.970 | 0.513 | 0.782 | 1.000 | 5166 |
| REACTOME_REGULATION_OF_HSF1_MEDIATED_HEAT_SHOCK_RESPONSE | 76 | 0.254 | 0.970 | 0.508 | 0.782 | 1.000 | 3960 |
| REACTOME_INACTIVATION_OF_CDC42_AND_RAC1 | 6 | 0.472 | 0.969 | 0.496 | 0.782 | 1.000 | 2378 |
| REACTOME_TRANSCRIPTIONAL_REGULATION_BY_RUNX2 | 101 | 0.242 | 0.968 | 0.505 | 0.783 | 1.000 | 4312 |
| REACTOME_NRAGE_SIGNALS_DEATH_THROUGH_JNK | 52 | 0.273 | 0.968 | 0.523 | 0.784 | 1.000 | 977 |
| REACTOME_SULFIDE_OXIDATION_TO_SULFATE | 6 | 0.473 | 0.967 | 0.496 | 0.784 | 1.000 | 1093 |
| REACTOME_INTERFERON_SIGNALING | 142 | 0.231 | 0.967 | 0.559 | 0.783 | 1.000 | 2948 |
| REACTOME_PROCESSING_AND_ACTIVATION_OF_SUMO | 10 | 0.400 | 0.966 | 0.494 | 0.785 | 1.000 | 2915 |
| REACTOME_NEUROTRANSMITTER_RELEASE_CYCLE | 25 | 0.321 | 0.965 | 0.494 | 0.785 | 1.000 | 3186 |
| REACTOME_HCMV_INFECTION | 96 | 0.241 | 0.964 | 0.550 | 0.786 | 1.000 | 2703 |
| REACTOME_AQUAPORIN_MEDIATED_TRANSPORT | 24 | 0.321 | 0.963 | 0.518 | 0.786 | 1.000 | 2333 |
| REACTOME_ABORTIVE_ELONGATION_OF_HIV_1_TRANSCRIPT_IN_THE_ABSENCE_OF_TAT | 23 | 0.328 | 0.962 | 0.527 | 0.788 | 1.000 | 2357 |
| REACTOME_DISEASES_ASSOCIATED_WITH_GLYCOSYLATION_PRECURSOR_BIOSYNTHESIS | 18 | 0.342 | 0.961 | 0.508 | 0.789 | 1.000 | 2566 |
| REACTOME_INWARDLY_RECTIFYING_K_CHANNELS | 13 | 0.378 | 0.960 | 0.518 | 0.792 | 1.000 | 782 |
| REACTOME_INLB_MEDIATED_ENTRY_OF_LISTERIA_MONOCYTOGENES_INTO_HOST_CELL | 13 | 0.377 | 0.959 | 0.512 | 0.791 | 1.000 | 1771 |
| REACTOME_NEDDYLATION | 208 | 0.214 | 0.953 | 0.571 | 0.805 | 1.000 | 3531 |
| REACTOME_REMOVAL_OF_AMINOTERMINAL_PROPEPTIDES_FROM_GAMMA_CARBOXYLATED_PROTEINS | 7 | 0.436 | 0.952 | 0.521 | 0.805 | 1.000 | 1464 |
| REACTOME_DOWNSTREAM_SIGNALING_OF_ACTIVATED_FGFR2 | 15 | 0.355 | 0.951 | 0.532 | 0.806 | 1.000 | 1020 |
| REACTOME_BETA_OXIDATION_OF_BUTANOYL_COA_TO_ACETYL_COA | 3 | 0.558 | 0.951 | 0.587 | 0.807 | 1.000 | 2693 |
| REACTOME_GAMMA_CARBOXYLATION_TRANSPORT_AND_AMINO_TERMINAL_CLEAVAGE_OF_PROTEINS | 8 | 0.434 | 0.951 | 0.515 | 0.806 | 1.000 | 1464 |
| REACTOME_REGULATION_OF_KIT_SIGNALING | 15 | 0.370 | 0.951 | 0.519 | 0.805 | 1.000 | 1443 |
| REACTOME_FGFR1C_LIGAND_BINDING_AND_ACTIVATION | 2 | 0.624 | 0.949 | 0.598 | 0.807 | 1.000 | 4782 |

| | | | | | | | |
|---|---|---|---|---|---|---|---|
| REACTOME_TRANSCRIPTIONAL_REGULATION_BY_TP53 | 343 | 0.204 | 0.946 | 0.620 | 0.812 | 1.000 | 2789 |
| REACTOME_SIGNAL_REGULATORY_PROTEIN_FAMILY_INTERACTIONS | 10 | 0.396 | 0.945 | 0.559 | 0.814 | 1.000 | 5160 |
| REACTOME_DOWNSTREAM_SIGNALING_OF_ACTIVATED_FGFR4 | 16 | 0.340 | 0.944 | 0.541 | 0.814 | 1.000 | 1020 |
| REACTOME_NUCLEOTIDE_LIKE_PURINERGIC_RECEPTORS | 5 | 0.473 | 0.944 | 0.542 | 0.814 | 1.000 | 2269 |
| REACTOME_FGFR1_MUTANT_RECEPTOR_ACTIVATION | 22 | 0.327 | 0.941 | 0.559 | 0.819 | 1.000 | 4894 |
| REACTOME_REGULATION_OF_RAS_BY_GAPS | 64 | 0.258 | 0.941 | 0.566 | 0.818 | 1.000 | 4312 |
| REACTOME_MET_ACTIVATES_PTPN11 | 3 | 0.563 | 0.941 | 0.590 | 0.817 | 1.000 | 1771 |
| REACTOME_CYP2E1_REACTIONS | 3 | 0.567 | 0.939 | 0.610 | 0.819 | 1.000 | 1194 |
| REACTOME_SLC_MEDIATED_TRANSMEMBRANE_TRANSPORT | 132 | 0.229 | 0.938 | 0.611 | 0.821 | 1.000 | 2055 |
| REACTOME_DECTIN_1_MEDIATED_NONCANONICAL_NF_KB_SIGNALING | 58 | 0.257 | 0.934 | 0.579 | 0.828 | 1.000 | 4312 |
| REACTOME_HEDGEHOG_OFF_STATE | 97 | 0.236 | 0.934 | 0.596 | 0.828 | 1.000 | 3985 |
| REACTOME_CELL_DEATH_SIGNALLING_VIA_NRAGE_NRIF_AND_NADE | 69 | 0.248 | 0.931 | 0.599 | 0.833 | 1.000 | 2765 |
| REACTOME_TP53_REGULATES_TRANSCRIPTION_OF_GENES_INVOLVED_IN_G1_CELL_CYCLE_ARREST | 13 | 0.357 | 0.931 | 0.564 | 0.832 | 1.000 | 1059 |
| REACTOME_OPSINS | 1 | 0.701 | 0.930 | 0.618 | 0.833 | 1.000 | 3803 |
| REACTOME_FOXO_MEDIATED_TRANSCRIPTION_OF_OXIDATIVE_STRESS_METABOLIC_AND_NEURONAL_GENES | 20 | 0.320 | 0.928 | 0.555 | 0.837 | 1.000 | 3687 |
| REACTOME_INTERLEUKIN_1_FAMILY_SIGNALING | 108 | 0.227 | 0.928 | 0.600 | 0.836 | 1.000 | 3985 |
| REACTOME_DIGESTION | 2 | 0.613 | 0.927 | 0.597 | 0.837 | 1.000 | 2844 |
| REACTOME_CD22_MEDIATED_BCR_REGULATION | 2 | 0.613 | 0.925 | 0.619 | 0.839 | 1.000 | 4915 |
| REACTOME_RAB_REGULATION_OF_TRAFFICKING | 111 | 0.226 | 0.924 | 0.625 | 0.841 | 1.000 | 3691 |
| REACTOME_DISEASES_OF_IMMUNE_SYSTEM | 16 | 0.339 | 0.923 | 0.569 | 0.842 | 1.000 | 317 |
| REACTOME_N_GLYCAN_TRIMMING_IN_THE_ER_AND_CALNEXIN_CALRETICULIN_CYCLE | 35 | 0.281 | 0.921 | 0.559 | 0.845 | 1.000 | 4151 |
| REACTOME_CRMPS_IN_SEMA3A_SIGNALING | 12 | 0.367 | 0.921 | 0.566 | 0.845 | 1.000 | 1589 |
| REACTOME_BETA_OXIDATION_OF_OCTANOYL_COA_TO_HEXANOYL_COA | 5 | 0.479 | 0.920 | 0.595 | 0.847 | 1.000 | 4043 |

| | | | | | | | |
|---|---|---|---|---|---|---|---|
| REACTOME_SIGNALING_BY_WNT | 221 | 0.206 | 0.918 | 0.668 | 0.850 | 1.000 | 3642 |
| REACTOME_G_ALPHA_S_SIGNALLING_EVENTS | 51 | 0.260 | 0.917 | 0.609 | 0.849 | 1.000 | 3387 |
| REACTOME_INTERLEUKIN_37_SIGNALING | 14 | 0.349 | 0.916 | 0.606 | 0.850 | 1.000 | 3101 |
| REACTOME_HIV_ELONGATION_ARREST_AND_RECOVERY | 32 | 0.289 | 0.916 | 0.596 | 0.849 | 1.000 | 2789 |
| REACTOME_ANTI_INFLAMMATORY_RESPONSE_FAVOURING_LEISHMANIA_PARASITE_INFECTION | 58 | 0.251 | 0.915 | 0.595 | 0.850 | 1.000 | 2269 |
| REACTOME_BETA_OXIDATION_OF_LAUROYL_COA_TO_DECANOYL_COA_COA | 4 | 0.503 | 0.915 | 0.596 | 0.849 | 1.000 | 4043 |
| REACTOME_DEGRADATION_OF_GLI1_BY_THE_PROTEASOME | 56 | 0.257 | 0.915 | 0.622 | 0.848 | 1.000 | 4312 |
| REACTOME_NUCLEOTIDE_BINDING_DOMAIN_LEUCINE_RICH_REPEAT_CONTAINING_RECEPTOR_NLR_SIGNALING_PATHWAYS | 47 | 0.264 | 0.915 | 0.606 | 0.847 | 1.000 | 2186 |
| REACTOME_SIGNALING_BY_FGFR2_IN_DISEASE | 28 | 0.300 | 0.915 | 0.601 | 0.847 | 1.000 | 2357 |
| REACTOME_FGFR2B_LIGAND_BINDING_AND_ACTIVATION | 3 | 0.550 | 0.915 | 0.627 | 0.846 | 1.000 | 1506 |
| REACTOME_CASPASE_ACTIVATION_VIA_EXTRINSIC_APOPTOTIC_SIGNALLING_PATHWAY | 21 | 0.312 | 0.912 | 0.599 | 0.850 | 1.000 | 400 |
| REACTOME_SODIUM_PROTON_EXCHANGERS | 5 | 0.462 | 0.911 | 0.583 | 0.851 | 1.000 | 1807 |
| REACTOME_GABA_RECEPTOR_ACTIVATION | 24 | 0.300 | 0.911 | 0.632 | 0.851 | 1.000 | 2182 |
| REACTOME_CYTOSOLIC_SULFONATION_OF_SMALL_MOLECULES | 15 | 0.338 | 0.910 | 0.545 | 0.851 | 1.000 | 1170 |
| REACTOME_PINK1_PRKN_MEDIATED_MITOPHAGY | 21 | 0.313 | 0.910 | 0.586 | 0.850 | 1.000 | 2103 |
| REACTOME_REGULATION_OF_RUNX3_EXPRESSION_AND_ACTIVITY | 55 | 0.250 | 0.910 | 0.637 | 0.850 | 1.000 | 5361 |
| REACTOME_JNK_C_JUN_KINASES_PHOSPHORYLATION_AND_ACTIVATION_MEDIATED_BY_ACTIVATED_HUMAN_TAK1 | 21 | 0.306 | 0.907 | 0.577 | 0.855 | 1.000 | 2841 |
| REACTOME_GAP_JUNCTION_DEGRADATION | 11 | 0.372 | 0.907 | 0.581 | 0.854 | 1.000 | 3583 |
| REACTOME_TRYPTOPHAN_CATABOLISM | 8 | 0.407 | 0.905 | 0.581 | 0.856 | 1.000 | 1870 |
| REACTOME_ROLE_OF_ABL_IN_ROBO_SLIT_SIGNALING | 7 | 0.414 | 0.902 | 0.586 | 0.861 | 1.000 | 2310 |
| REACTOME_METABOLISM_OF_COFACTORS | 18 | 0.322 | 0.902 | 0.583 | 0.861 | 1.000 | 2338 |
| REACTOME_APC_C_CDC20_MEDIATED_DEGRADATION_OF_CYCLIN_B | 24 | 0.301 | 0.900 | 0.601 | 0.864 | 1.000 | 3779 |

| | | | | | | | |
|---|---|---|---|---|---|---|---|
| REACTOME_THROMBOXANE_SIGNALLING_THROUGH_TP_RECEPTOR | 11 | 0.367 | 0.900 | 0.605 | 0.863 | 1.000 | 2180 |
| REACTOME_SARS_COV_1_INFECTION | 46 | 0.258 | 0.899 | 0.646 | 0.864 | 1.000 | 2628 |
| REACTOME_HUR_ELAVL1_BINDS_AND_STABILIZES_MRNA | 8 | 0.401 | 0.898 | 0.601 | 0.866 | 1.000 | 619 |
| REACTOME_CD28_CO_STIMULATION | 28 | 0.293 | 0.897 | 0.619 | 0.867 | 1.000 | 2415 |
| REACTOME_NOTCH3_ACTIVATION_AND_TRANSMISSION_OF_SIGNAL_TO_THE_NUCLEUS | 21 | 0.305 | 0.893 | 0.622 | 0.874 | 1.000 | 1569 |
| REACTOME_RUNX1_REGULATES_TRANSCRIPTION_OF_GENES_INVOLVED_IN_DIFFERENTIATION_OF_HSCS | 74 | 0.235 | 0.892 | 0.670 | 0.874 | 1.000 | 4339 |
| REACTOME_FCGR_ACTIVATION | 5 | 0.467 | 0.891 | 0.616 | 0.875 | 1.000 | 576 |
| REACTOME_DEGRADATION_OF_BETA_CATENIN_BY_THE_DESTRUCTION_COMPLEX | 80 | 0.230 | 0.890 | 0.666 | 0.876 | 1.000 | 4312 |
| REACTOME_SUMOYLATION_OF_TRANSCRIPTION_COFACTORS | 43 | 0.254 | 0.888 | 0.648 | 0.879 | 1.000 | 2977 |
| REACTOME_METHIONINE_SALVAGE_PATHWAY | 6 | 0.429 | 0.887 | 0.611 | 0.880 | 1.000 | 2706 |
| REACTOME_FORMATION_OF_ATP_BY_CHEMIOSMOTIC_COUPLING | 16 | 0.326 | 0.886 | 0.631 | 0.881 | 1.000 | 5312 |
| REACTOME_SULFUR_AMINO_ACID_METABOLISM | 22 | 0.300 | 0.884 | 0.626 | 0.886 | 1.000 | 1967 |
| REACTOME_APOPTOSIS_INDUCED_DNA_FRAGMENTATION | 9 | 0.384 | 0.882 | 0.615 | 0.888 | 1.000 | 2155 |
| REACTOME_MYOCLONIC_EPILEPSY_OF_LAFORA | 7 | 0.406 | 0.880 | 0.627 | 0.892 | 1.000 | 1569 |
| REACTOME_TAK1_ACTIVATES_NFKB_BY_PHOSPHORYLATION_AND_ACTIVATION_OF_IKKS_COMPLEX | 29 | 0.281 | 0.879 | 0.638 | 0.893 | 1.000 | 2936 |
| REACTOME_ACYL_CHAIN_REMODELLING_OF_PI | 6 | 0.419 | 0.874 | 0.639 | 0.902 | 1.000 | 231 |
| REACTOME_SEMA3A_PLEXIN_REPULSION_SIGNALING_BY_INHIBITING_INTEGRIN_ADHESION | 12 | 0.346 | 0.874 | 0.639 | 0.901 | 1.000 | 2938 |
| REACTOME_NOREPINEPHRINE_NEUROTRANSMITTER_RELEASE_CYCLE | 10 | 0.357 | 0.873 | 0.640 | 0.901 | 1.000 | 1496 |
| REACTOME_PP2A_MEDIATED_DEPHOSPHORYLATION_OF_KEY_METABOLIC_FACTORS | 6 | 0.421 | 0.872 | 0.651 | 0.903 | 1.000 | 2088 |
| REACTOME_SYNTHESIS_OF_PIPS_AT_THE_PLASMA_MEMBRANE | 45 | 0.251 | 0.870 | 0.691 | 0.905 | 1.000 | 3736 |
| REACTOME_METABOLISM_OF_INGESTED_SEMET_SEC_MESEC_INTO_H2SE | 4 | 0.472 | 0.870 | 0.635 | 0.904 | 1.000 | 312 |

| | | | | | | | |
|---|---|---|---|---|---|---|---|
| REACTOME_VLDL_CLEARANCE | 3 | 0.517 | 0.870 | 0.646 | 0.903 | 1.000 | 406 |
| REACTOME_MAPK6_MAPK4_SIGNALING | 82 | 0.229 | 0.869 | 0.700 | 0.903 | 1.000 | 3985 |
| REACTOME_MRNA_CAPPING | 28 | 0.285 | 0.869 | 0.654 | 0.903 | 1.000 | 2549 |
| REACTOME_SUMO_IS_TRANSFERRED_FROM_E1_TO_E2_UBE2I_UBC9_ | 7 | 0.403 | 0.869 | 0.644 | 0.902 | 1.000 | 4031 |
| REACTOME_FOLDING_OF_ACTIN_BY_CCT_TRIC | 9 | 0.380 | 0.868 | 0.626 | 0.903 | 1.000 | 3065 |
| REACTOME_APC_CDC20_MEDIATED_DEGRADATION_OF_NEK2A | 26 | 0.283 | 0.866 | 0.660 | 0.905 | 1.000 | 3290 |
| REACTOME_ALTERNATIVE_COMPLEMENT_ACTIVATION | 4 | 0.471 | 0.866 | 0.642 | 0.904 | 1.000 | 2284 |
| REACTOME_INTERLEUKIN_1_SIGNALING | 92 | 0.222 | 0.866 | 0.740 | 0.903 | 1.000 | 4312 |
| REACTOME_NRCAM_INTERACTIONS | 6 | 0.422 | 0.866 | 0.652 | 0.902 | 1.000 | 2486 |
| REACTOME_PHASE_2_PLATEAU_PHASE | 9 | 0.375 | 0.864 | 0.611 | 0.904 | 1.000 | 2515 |
| REACTOME_RUNX1_REGULATES_EXPRESSION_OF_COMPONENTS_OF_TIGHT_JUNCTIONS | 4 | 0.474 | 0.864 | 0.632 | 0.903 | 1.000 | 3465 |
| REACTOME_DOWNSTREAM_SIGNALING_OF_ACTIVATED_FGFR3 | 15 | 0.326 | 0.864 | 0.673 | 0.902 | 1.000 | 1136 |
| REACTOME_TIGHT_JUNCTION_INTERACTIONS | 17 | 0.320 | 0.862 | 0.655 | 0.904 | 1.000 | 1401 |
| REACTOME_TIE2_SIGNALING | 13 | 0.343 | 0.861 | 0.659 | 0.905 | 1.000 | 816 |
| REACTOME_NOTCH_HLH_TRANSCRIPTION_PATHWAY | 27 | 0.280 | 0.859 | 0.663 | 0.908 | 1.000 | 3349 |
| REACTOME_APOPTOTIC_FACTOR_MEDIATED_RESPONSE | 18 | 0.303 | 0.859 | 0.670 | 0.908 | 1.000 | 1851 |
| REACTOME_SYNTHESIS_OF_GLYCOSYLPHOSPHATIDYLINOSITOL_GPI_ | 16 | 0.314 | 0.858 | 0.634 | 0.908 | 1.000 | 2009 |
| REACTOME_FRS_MEDIATED_FGFR2_SIGNALING | 11 | 0.346 | 0.856 | 0.644 | 0.912 | 1.000 | 1020 |
| REACTOME_SIGNALING_BY_NOTCH | 166 | 0.198 | 0.853 | 0.843 | 0.917 | 1.000 | 4196 |
| REACTOME_SODIUM_COUPLED_PHOSPHATE_COTRANSPORTERS | 2 | 0.572 | 0.852 | 0.746 | 0.917 | 1.000 | 1486 |
| REACTOME_DISEASES_OF_PROGRAMMED_CELL_DEATH | 23 | 0.287 | 0.852 | 0.666 | 0.916 | 1.000 | 2215 |
| REACTOME_REGULATION_OF_FOXO_TRANSCRIPTIONAL_ACTIVITY_BY_ACETYLATION | 10 | 0.361 | 0.850 | 0.660 | 0.919 | 1.000 | 2775 |
| REACTOME_GLUTATHIONE_SYNTHESIS_AND_RECYCLING | 10 | 0.356 | 0.849 | 0.647 | 0.919 | 1.000 | 1552 |
| REACTOME_CONSTITUTIVE_SIGNALING_BY_OVEREXPRESSED_ERBB2 | 11 | 0.355 | 0.848 | 0.663 | 0.920 | 1.000 | 4750 |

| | | | | | | | |
|---|---|---|---|---|---|---|---|
| REACTOME_ACETYLCHOLINE_NEUROTRANSMITTER_RELEASE_CYCLE | 10 | 0.357 | 0.846 | 0.651 | 0.923 | 1.000 | 1496 |
| REACTOME_GLUCOSE_METABOLISM | 77 | 0.226 | 0.846 | 0.755 | 0.922 | 1.000 | 2706 |
| REACTOME_FORMATION_OF_THE_EARLY_ELONGATION_COMPLEX | 32 | 0.258 | 0.843 | 0.725 | 0.927 | 1.000 | 2549 |
| REACTOME_HIV_LIFE_CYCLE | 144 | 0.200 | 0.842 | 0.833 | 0.927 | 1.000 | 3118 |
| REACTOME_TFAP2_AP_2_FAMILY_REGULATES_TRANSCRIPTION_OF_GROWTH_FACTORS_AND_THEIR_RECEPTORS | 7 | 0.392 | 0.841 | 0.656 | 0.928 | 1.000 | 1649 |
| REACTOME_CLEC7A_DECTIN_1_SIGNALING | 94 | 0.213 | 0.841 | 0.790 | 0.928 | 1.000 | 4312 |
| REACTOME_CONSTITUTIVE_SIGNALING_BY_AKT1_E17K_IN_CANCER | 26 | 0.273 | 0.838 | 0.699 | 0.931 | 1.000 | 2117 |
| REACTOME_RAB_GEFS_EXCHANGE_GTP_FOR_GDP_ON_RABS | 82 | 0.216 | 0.837 | 0.768 | 0.933 | 1.000 | 3691 |
| REACTOME_STABILIZATION_OF_P53 | 55 | 0.231 | 0.836 | 0.741 | 0.933 | 1.000 | 4568 |
| REACTOME_SIGNALING_BY_HEDGEHOG | 123 | 0.204 | 0.836 | 0.841 | 0.932 | 1.000 | 3985 |
| REACTOME_ORGANIC_CATION_ANION_ZWITTERION_TRANSPORT | 6 | 0.401 | 0.836 | 0.663 | 0.931 | 1.000 | 155 |
| REACTOME_DEGRADATION_OF_CYSTEINE_AND_HOMOCYSTEINE | 12 | 0.336 | 0.836 | 0.677 | 0.930 | 1.000 | 1093 |
| REACTOME_RNA_POLYMERASE_III_TRANSCRIPTION_INITIATION_FROM_TYPE_1_PROMOTER | 28 | 0.271 | 0.836 | 0.724 | 0.930 | 1.000 | 803 |
| REACTOME_AUF1_HNRNP_D0_BINDS_AND_DESTABILIZES_MRNA | 53 | 0.233 | 0.834 | 0.763 | 0.931 | 1.000 | 4312 |
| REACTOME_RUNX1_REGULATES_TRANSCRIPTION_OF_GENES_INVOLVED_IN_WNT_SIGNALING | 4 | 0.453 | 0.833 | 0.682 | 0.931 | 1.000 | 1908 |
| REACTOME_TRAF6_MEDIATED_IRF7_ACTIVATION_IN_TLR7_8_OR_9_SIGNALING | 11 | 0.336 | 0.831 | 0.693 | 0.934 | 1.000 | 3779 |
| REACTOME_HIV_TRANSCRIPTION_ELONGATION | 41 | 0.247 | 0.830 | 0.754 | 0.936 | 1.000 | 2789 |
| REACTOME_MISCELLANEOUS_TRANSPORT_AND_BINDING_EVENTS | 21 | 0.281 | 0.830 | 0.707 | 0.935 | 1.000 | 2392 |
| REACTOME_ORGANIC_CATION_TRANSPORT | 6 | 0.401 | 0.828 | 0.690 | 0.937 | 1.000 | 155 |
| REACTOME_UCH_PROTEINASES | 83 | 0.212 | 0.828 | 0.820 | 0.937 | 1.000 | 4908 |
| REACTOME_DEFECTIVE_CFTR_CAUSES_CYSTIC_FIBROSIS | 58 | 0.229 | 0.825 | 0.775 | 0.940 | 1.000 | 4312 |
| REACTOME_FRS_MEDIATED_FGFR4_SIGNALING | 12 | 0.324 | 0.823 | 0.675 | 0.943 | 1.000 | 1020 |

| | | | | | | | |
|---|---|---|---|---|---|---|---|
| REACTOME_BETA_CATENIN_PHOSPHORYLATION_CASCADE | 16 | 0.300 | 0.822 | 0.716 | 0.944 | 1.000 | 3597 |
| REACTOME_SIGNALING_BY_MEMBRANE_TETHERED_FUSIONS_OF_PDGFRA_OR_PDGFRB | 3 | 0.480 | 0.820 | 0.709 | 0.946 | 1.000 | 484 |
| REACTOME_ACTIVATION_OF_PPARGC1A_PGC_1ALPHA_BY_PHOSPHORYLATION | 9 | 0.360 | 0.820 | 0.705 | 0.945 | 1.000 | 568 |
| REACTOME_ENDOSOMAL_SORTING_COMPLEX_REQUIRED_FOR_TRANSPORT_ESCRT_ | 31 | 0.260 | 0.820 | 0.760 | 0.944 | 1.000 | 2703 |
| REACTOME_METABOLISM_OF_POLYAMINES | 57 | 0.225 | 0.819 | 0.778 | 0.944 | 1.000 | 4312 |
| REACTOME_MTOR_SIGNALLING | 39 | 0.244 | 0.819 | 0.776 | 0.944 | 1.000 | 2367 |
| REACTOME_TRIGLYCERIDE_BIOSYNTHESIS | 10 | 0.345 | 0.818 | 0.727 | 0.944 | 1.000 | 280 |
| REACTOME_REACTIONS_SPECIFIC_TO_THE_COMPLEX_N_GLYCAN_SYNTHESIS_PATHWAY | 5 | 0.418 | 0.817 | 0.694 | 0.945 | 1.000 | 2451 |
| REACTOME_TP53_REGULATES_TRANSCRIPTION_OF_DEATH_RECEPTORS_AND_LIGANDS | 10 | 0.348 | 0.815 | 0.702 | 0.946 | 1.000 | 1526 |
| REACTOME_PHOSPHORYLATION_OF_THE_APC_C | 20 | 0.280 | 0.814 | 0.718 | 0.947 | 1.000 | 3550 |
| REACTOME_REGULATION_OF_PTEN_GENE_TRANSCRIPTION | 60 | 0.223 | 0.814 | 0.802 | 0.947 | 1.000 | 1263 |
| REACTOME_FGFR3_LIGAND_BINDING_AND_ACTIVATION | 4 | 0.449 | 0.813 | 0.702 | 0.946 | 1.000 | 1136 |
| REACTOME_SIGNALING_BY_CYTOSOLIC_FGFR1_FUSION_MUTANTS | 18 | 0.288 | 0.813 | 0.710 | 0.946 | 1.000 | 4894 |
| REACTOME_INTERFERON_ALPHA_BETA_SIGNALING | 47 | 0.231 | 0.809 | 0.793 | 0.950 | 1.000 | 1175 |
| REACTOME_SIGNALING_BY_NOTCH1 | 66 | 0.218 | 0.808 | 0.828 | 0.951 | 1.000 | 3349 |
| REACTOME_CROSS_PRESENTATION_OF_SOLUBLE_EXOGENOUS_ANTIGENS_ENDOSOMES_ | 45 | 0.234 | 0.807 | 0.815 | 0.952 | 1.000 | 4312 |
| REACTOME_DISASSEMBLY_OF_THE_DESTRUCTION_COMPLEX_AND_RECRUITMENT_OF_AXIN_TO_THE_MEMBRANE | 26 | 0.265 | 0.807 | 0.751 | 0.951 | 1.000 | 629 |
| REACTOME_TP53_REGULATES_TRANSCRIPTION_OF_CELL_CYCLE_GENES | 48 | 0.227 | 0.807 | 0.789 | 0.950 | 1.000 | 1282 |
| REACTOME_RNA_POLYMERASE_II_TRANSCRIPTION_TERMINATION | 63 | 0.219 | 0.807 | 0.791 | 0.949 | 1.000 | 4896 |
| REACTOME_GLUCAGON_SIGNALING_IN_METABOLIC_REGULATION | 16 | 0.295 | 0.806 | 0.727 | 0.949 | 1.000 | 2182 |
| REACTOME_CATION_COUPLED_CHLORIDE_COTRANSPORTERS | 4 | 0.449 | 0.804 | 0.715 | 0.951 | 1.000 | 2994 |

| | | | | | | | |
|---|---|---|---|---|---|---|---|
| REACTOME_GLUCONEOGENESIS | 25 | 0.268 | 0.803 | 0.771 | 0.952 | 1.000 | 2706 |
| REACTOME_LATE_ENDOSOMAL_MICROAUTOPHAGY | 30 | 0.257 | 0.801 | 0.784 | 0.954 | 1.000 | 2703 |
| REACTOME_PI_3K_CASCADE_FGFR1 | 8 | 0.359 | 0.800 | 0.741 | 0.954 | 1.000 | 720 |
| REACTOME_GABA_SYNTHESIS_RELEASE_REUPTAKE_AND_DEGRADATION | 10 | 0.341 | 0.800 | 0.739 | 0.954 | 1.000 | 1678 |
| REACTOME_CELLULAR_HEXOSE_TRANSPORT | 12 | 0.323 | 0.800 | 0.715 | 0.953 | 1.000 | 3564 |
| REACTOME_ANTIVIRAL_MECHANISM_BY_IFN_STIMULATED_GENES | 74 | 0.210 | 0.797 | 0.835 | 0.956 | 1.000 | 3779 |
| REACTOME_DNA_DAMAGE_RECOGNITION_IN_GG_NER | 37 | 0.240 | 0.795 | 0.809 | 0.959 | 1.000 | 3779 |
| REACTOME_CD209_DC_SIGN_SIGNALING | 18 | 0.288 | 0.795 | 0.756 | 0.958 | 1.000 | 3953 |
| REACTOME_TCF_DEPENDENT_SIGNALING_IN_RESPONSE_TO_WNT | 150 | 0.188 | 0.793 | 0.916 | 0.960 | 1.000 | 3985 |
| REACTOME_KILLING_MECHANISMS | 9 | 0.342 | 0.791 | 0.738 | 0.961 | 1.000 | 2378 |
| REACTOME_CYCLIN_D_ASSOCIATED_EVENTS_IN_G1 | 46 | 0.229 | 0.789 | 0.826 | 0.963 | 1.000 | 3480 |
| REACTOME_REGULATION_OF_MECP2_EXPRESSION_AND_ACTIVITY | 29 | 0.258 | 0.788 | 0.790 | 0.964 | 1.000 | 1249 |
| REACTOME_FORMATION_OF_RNA_POL_II_ELONGATION_COMPLEX_ | 56 | 0.220 | 0.788 | 0.804 | 0.963 | 1.000 | 2789 |
| REACTOME_ROLE_OF_SECOND_MESSENGERS_IN_NETRIN_1_SIGNALING | 3 | 0.479 | 0.787 | 0.764 | 0.963 | 1.000 | 2494 |
| REACTOME_PROTEIN_METHYLATION | 16 | 0.291 | 0.787 | 0.765 | 0.962 | 1.000 | 2175 |
| REACTOME_SEROTONIN_NEUROTRANSMITTER_RELEASE_CYCLE | 11 | 0.326 | 0.784 | 0.726 | 0.965 | 1.000 | 1496 |
| REACTOME_INTERLEUKIN_15_SIGNALING | 10 | 0.326 | 0.781 | 0.754 | 0.970 | 1.000 | 3707 |
| REACTOME_KERATAN_SULFATE_KERATIN_METABOLISM | 23 | 0.261 | 0.781 | 0.790 | 0.969 | 1.000 | 3610 |
| REACTOME_ACTIVATED_PKN1_STIMULATES_TRANSCRIPTION_OF_AR_ANDROGEN_RECEPTOR_REGULATED_GENES_KLK2_AND_KLK3 | 14 | 0.293 | 0.779 | 0.737 | 0.970 | 1.000 | 4687 |
| REACTOME_ADP_SIGNALLING_THROUGH_P2Y_PURINOCEPTOR_12 | 10 | 0.323 | 0.778 | 0.724 | 0.970 | 1.000 | 2180 |
| REACTOME_SUMO_IS_CONJUGATED_TO_E1_UBA2_SAE1_ | 5 | 0.397 | 0.773 | 0.732 | 0.977 | 1.000 | 4031 |
| REACTOME_PEROXISOMAL_PROTEIN_IMPORT | 55 | 0.215 | 0.772 | 0.858 | 0.977 | 1.000 | 1569 |

| | | | | | | | |
|---|---|---|---|---|---|---|---|
| REACTOME_TRANSCRIPTIONAL_REGULATION_BY_SMALL_RNAS | 57 | 0.212 | 0.766 | 0.868 | 0.985 | 1.000 | 2672 |
| REACTOME_DISEASES_ASSOCIATED_WITH_N_GLYCOSYLATION_OF_PROTEINS | 17 | 0.274 | 0.766 | 0.788 | 0.984 | 1.000 | 1559 |
| REACTOME_RNA_POLYMERASE_I_TRANSCRIPTION_TERMINATION | 30 | 0.242 | 0.766 | 0.823 | 0.983 | 1.000 | 2549 |
| REACTOME_MAPK1_ERK2_ACTIVATION | 8 | 0.340 | 0.766 | 0.761 | 0.982 | 1.000 | 5263 |
| REACTOME_SIGNALING_BY_HIPPO | 19 | 0.274 | 0.765 | 0.814 | 0.982 | 1.000 | 4364 |
| REACTOME_PROTEIN_LOCALIZATION | 151 | 0.180 | 0.764 | 0.941 | 0.982 | 1.000 | 2951 |
| REACTOME_MASTL_FACILITATES_MITOTIC_PROGRESSION | 10 | 0.322 | 0.758 | 0.772 | 0.989 | 1.000 | 3550 |
| REACTOME_APOPTOTIC_CLEAVAGE_OF_CELL_ADHESION_PROTEINS | 8 | 0.338 | 0.757 | 0.764 | 0.989 | 1.000 | 3597 |
| REACTOME_PRE_NOTCH_EXPRESSION_AND_PROCESSING | 57 | 0.211 | 0.757 | 0.897 | 0.988 | 1.000 | 3326 |
| REACTOME_FORMATION_OF_XYLULOSE_5_PHOSPHATE | 5 | 0.386 | 0.757 | 0.744 | 0.988 | 1.000 | 577 |
| REACTOME_INHIBITION_OF_THE_PROTEOLYTIC_ACTIVITY_OF_APC_C_REQUIRED_FOR_THE_ONSET_OF_ANAPHASE_BY_MITOTIC_SPINDLE_CHECKPOINT_COMPONENTS | 21 | 0.259 | 0.756 | 0.827 | 0.988 | 1.000 | 3290 |
| REACTOME_PROGRAMMED_CELL_DEATH | 163 | 0.176 | 0.756 | 0.970 | 0.987 | 1.000 | 1851 |
| REACTOME_ACYL_CHAIN_REMODELLING_OF_PS | 11 | 0.306 | 0.755 | 0.781 | 0.987 | 1.000 | 2769 |
| REACTOME_NEGATIVE_REGULATION_OF_NOTCH4_SIGNALING | 54 | 0.208 | 0.753 | 0.888 | 0.989 | 1.000 | 4312 |
| REACTOME_SYNTHESIS_OF_PROSTAGLANDINS_PG_AND_THROMBOXANES_TX_ | 9 | 0.322 | 0.748 | 0.784 | 0.995 | 1.000 | 1097 |
| REACTOME_RNA_POLYMERASE_I_TRANSCRIPTION | 60 | 0.203 | 0.746 | 0.894 | 0.996 | 1.000 | 3350 |
| REACTOME_SHC_RELATED_EVENTS_TRIGGERED_BY_IGF1R | 7 | 0.348 | 0.745 | 0.801 | 0.996 | 1.000 | 4574 |
| REACTOME_RESOLUTION_OF_SISTER_CHROMATID_COHESION | 116 | 0.182 | 0.745 | 0.953 | 0.995 | 1.000 | 2493 |
| REACTOME_RECOGNITION_AND_ASSOCIATION_OF_DNA_GLYCOSYLASE_WITH_SITE_CONTAINING_AN_AFFECTED_PURINE | 19 | 0.262 | 0.745 | 0.849 | 0.994 | 1.000 | 3185 |
| REACTOME_AMINE_LIGAND_BINDING_RECEPTORS | 6 | 0.360 | 0.745 | 0.806 | 0.993 | 1.000 | 320 |
| REACTOME_FRS_MEDIATED_FGFR3_SIGNALING | 11 | 0.307 | 0.744 | 0.794 | 0.993 | 1.000 | 1136 |

| | | | | | | | |
|---|---|---|---|---|---|---|---|
| REACTOME_P75_NTR_RECEPTOR_MEDIATED_SIGNALLING | 87 | 0.191 | 0.744 | 0.929 | 0.992 | 1.000 | 2841 |
| REACTOME_DEFECTS_IN_BIOTIN_BTN_METABOLISM | 8 | 0.339 | 0.743 | 0.776 | 0.992 | 1.000 | 2489 |
| REACTOME_TYSND1_CLEAVES_PEROXISOMAL_PROTEINS | 7 | 0.346 | 0.743 | 0.770 | 0.991 | 1.000 | 8318 |
| REACTOME_INTERFERON_GAMMA_SIGNALING | 57 | 0.205 | 0.742 | 0.908 | 0.990 | 1.000 | 3834 |
| REACTOME_SIGNALING_BY_FGFR1_IN_DISEASE | 28 | 0.239 | 0.742 | 0.864 | 0.990 | 1.000 | 4894 |
| REACTOME_SIGNAL_AMPLIFICATION | 16 | 0.268 | 0.741 | 0.817 | 0.989 | 1.000 | 2180 |
| REACTOME_MITOTIC_PROPHASE | 91 | 0.187 | 0.741 | 0.937 | 0.988 | 1.000 | 2672 |
| REACTOME_CRISTAE_FORMATION | 29 | 0.239 | 0.741 | 0.856 | 0.988 | 1.000 | 5312 |
| REACTOME_SYNTHESIS_OF_PC | 23 | 0.251 | 0.741 | 0.846 | 0.987 | 1.000 | 1217 |
| REACTOME_AKT_PHOSPHORYLATES_TARGETS_IN_THE_CYTOSOL | 14 | 0.278 | 0.740 | 0.793 | 0.987 | 1.000 | 4634 |
| REACTOME_LDL_CLEARANCE | 18 | 0.264 | 0.740 | 0.835 | 0.986 | 1.000 | 817 |
| REACTOME_ADP_SIGNALLING_THROUGH_P2Y_PURINOCEPTOR_1 | 11 | 0.303 | 0.739 | 0.787 | 0.985 | 1.000 | 3490 |
| REACTOME_TRANSCRIPTION_OF_THE_HIV_GENOME | 66 | 0.201 | 0.739 | 0.930 | 0.985 | 1.000 | 3028 |
| REACTOME_SCF_SKP2_MEDIATED_DEGRADATION_OF_P27_P21 | 59 | 0.199 | 0.737 | 0.908 | 0.985 | 1.000 | 4312 |
| REACTOME_METABOLISM_OF_NUCLEOTIDES | 84 | 0.190 | 0.737 | 0.929 | 0.985 | 1.000 | 2691 |
| REACTOME_G1_S_DNA_DAMAGE_CHECKPOINTS | 65 | 0.199 | 0.737 | 0.937 | 0.984 | 1.000 | 4312 |
| REACTOME_REGULATION_OF_RUNX2_EXPRESSION_AND_ACTIVITY | 66 | 0.196 | 0.736 | 0.918 | 0.983 | 1.000 | 4312 |
| REACTOME_GLUTAMATE_NEUROTRANSMITTER_RELEASE_CYCLE | 15 | 0.276 | 0.735 | 0.825 | 0.984 | 1.000 | 1496 |
| REACTOME_TRANSLATION_OF_STRUCTURAL_PROTEINS | 25 | 0.246 | 0.734 | 0.866 | 0.984 | 1.000 | 3303 |
| REACTOME_DOWNSTREAM_SIGNALING_OF_ACTIVATED_FGFR1 | 15 | 0.272 | 0.734 | 0.831 | 0.983 | 1.000 | 816 |
| REACTOME_GENOME_REPLICATION_AND_TRANSCRIPTION | 6 | 0.359 | 0.732 | 0.777 | 0.985 | 1.000 | 3208 |
| REACTOME_HDACS_DEACETYLATE_HISTONES | 38 | 0.217 | 0.731 | 0.884 | 0.984 | 1.000 | 4605 |
| REACTOME_FGFR2_ALTERNATIVE_SPLICING | 26 | 0.238 | 0.731 | 0.878 | 0.983 | 1.000 | 3383 |
| REACTOME_DCC_MEDIATED_ATTRACTIVE_SIGNALING | 12 | 0.294 | 0.730 | 0.822 | 0.984 | 1.000 | 2765 |
| REACTOME_CONSTITUTIVE_SIGNALING_BY_EGFRVIII | 12 | 0.285 | 0.727 | 0.815 | 0.986 | 1.000 | 2078 |
| REACTOME_TRANSCRIPTIONAL_REGULATION_BY_MECP2 | 46 | 0.211 | 0.724 | 0.898 | 0.988 | 1.000 | 1249 |

| | | | | | | | |
|---|---|---|---|---|---|---|---|
| REACTOME_LIPOPHAGY | 8 | 0.327 | 0.723 | 0.804 | 0.988 | 1.000 | 2017 |
| REACTOME_SIGNALING_BY_NOTCH3 | 40 | 0.217 | 0.723 | 0.906 | 0.987 | 1.000 | 2775 |
| REACTOME_DEGRADATION_OF_DVL | 53 | 0.201 | 0.723 | 0.935 | 0.986 | 1.000 | 4312 |
| REACTOME_TNFR2_NON_CANONICAL_NF_KB_PATHWAY | 76 | 0.188 | 0.723 | 0.956 | 0.985 | 1.000 | 4324 |
| REACTOME_LTC4_CYSLTR_MEDIATED_IL4_PRODUCTION | 2 | 0.475 | 0.721 | 0.823 | 0.986 | 1.000 | 3780 |
| REACTOME_SENSING_OF_DNA_DOUBLE_STRAND_BREAKS | 6 | 0.349 | 0.718 | 0.812 | 0.988 | 1.000 | 4402 |
| REACTOME_ASSOCIATION_OF_TRIC_CCT_WITH_TARGET_PROTEINS_DURING_BIOSYNTHESIS | 35 | 0.217 | 0.718 | 0.908 | 0.988 | 1.000 | 1271 |
| REACTOME_SMALL_INTERFERING_RNA_SIRNA_BIOGENESIS | 9 | 0.311 | 0.713 | 0.815 | 0.991 | 1.000 | 1991 |
| REACTOME_THE_RETINOID_CYCLE_IN_CONES_DAYLIGHT_VISION_ | 1 | 0.536 | 0.709 | 0.913 | 0.995 | 1.000 | 5903 |
| REACTOME_LOSS_OF_MECP2_BINDING_ABILITY_TO_THE_NCOR_SMRT_COMPLEX | 7 | 0.334 | 0.709 | 0.804 | 0.994 | 1.000 | 8463 |
| REACTOME_RNA_POLYMERASE_I_PROMOTER_ESCAPE | 40 | 0.210 | 0.706 | 0.894 | 0.996 | 1.000 | 4944 |
| REACTOME_PYRUVATE_METABOLISM | 26 | 0.231 | 0.706 | 0.868 | 0.995 | 1.000 | 4247 |
| REACTOME_CONVERSION_FROM_APC_C_CDC20_TO_APC_C_CDH1_IN_LATE_ANAPHASE | 20 | 0.246 | 0.704 | 0.875 | 0.996 | 1.000 | 3142 |
| REACTOME_DOWNREGULATION_OF_ERBB4_SIGNALING | 8 | 0.317 | 0.703 | 0.846 | 0.996 | 1.000 | 1569 |
| REACTOME_DAP12_SIGNALING | 21 | 0.240 | 0.703 | 0.889 | 0.995 | 1.000 | 816 |
| REACTOME_DEGRADATION_OF_AXIN | 53 | 0.195 | 0.700 | 0.947 | 0.997 | 1.000 | 4312 |
| REACTOME_DEACTIVATION_OF_THE_BETA_CATENIN_TRANSACTIVATING_COMPLEX | 35 | 0.214 | 0.700 | 0.905 | 0.996 | 1.000 | 3779 |
| REACTOME_MICRORNA_MIRNA_BIOGENESIS | 24 | 0.233 | 0.700 | 0.894 | 0.995 | 1.000 | 2357 |
| REACTOME_ANTIGEN_ACTIVATES_B_CELL_RECEPTOR_BCR_LEADING_TO_GENERATION_OF_SECOND_MESSENGERS | 22 | 0.237 | 0.698 | 0.873 | 0.995 | 1.000 | 3050 |
| REACTOME_RECYCLING_OF_BILE_ACIDS_AND_SALTS | 8 | 0.319 | 0.697 | 0.833 | 0.996 | 1.000 | 1886 |
| REACTOME_TRANS_GOLGI_NETWORK_VESICLE_BUDDING | 68 | 0.184 | 0.697 | 0.964 | 0.995 | 1.000 | 3171 |
| REACTOME_BBSOME_MEDIATED_CARGO_TARGETING_TO_CILIUM | 20 | 0.242 | 0.695 | 0.885 | 0.995 | 1.000 | 1347 |
| REACTOME_RUNX1_INTERACTS_WITH_CO_FACTORS_WHOSE_PR | 35 | 0.213 | 0.695 | 0.909 | 0.994 | 1.000 | 2129 |

| | | | | | | | |
|---|---|---|---|---|---|---|---|
| ECISE_EFFECT_ON_RUNX1_TARGETS_IS_NOT_KNOWN | | | | | | | |
| REACTOME_SYNTHESIS_SECRETION_AND_DEACYLATION_OF_GHRELIN | 6 | 0.337 | 0.694 | 0.824 | 0.994 | 1.000 | 3865 |
| REACTOME_DAP12_INTERACTIONS | 24 | 0.231 | 0.691 | 0.881 | 0.996 | 1.000 | 816 |
| REACTOME_CLASS_I_MHC_MEDIATED_ANTIGEN_PROCESSING_PRESENTATION | 319 | 0.150 | 0.691 | 1.000 | 0.995 | 1.000 | 4009 |
| REACTOME_REGULATION_OF_PTEN_LOCALIZATION | 9 | 0.296 | 0.690 | 0.845 | 0.995 | 1.000 | 1569 |
| REACTOME_SYNTHESIS_OF_VERY_LONG_CHAIN_FATTY_ACYL_COAS | 15 | 0.260 | 0.690 | 0.871 | 0.994 | 1.000 | 336 |
| REACTOME_MRNA_SPLICING_MINOR_PATHWAY | 52 | 0.195 | 0.688 | 0.939 | 0.994 | 1.000 | 4484 |
| REACTOME_PASSIVE_TRANSPORT_BY_AQUAPORINS | 4 | 0.365 | 0.686 | 0.826 | 0.995 | 1.000 | 2333 |
| REACTOME_HIV_TRANSCRIPTION_INITIATION | 44 | 0.199 | 0.686 | 0.950 | 0.994 | 1.000 | 3028 |
| REACTOME_BIOSYNTHESIS_OF_EPA_DERIVED_SPMS | 2 | 0.459 | 0.685 | 0.864 | 0.994 | 1.000 | 6876 |
| REACTOME_RRNA_PROCESSING | 197 | 0.156 | 0.684 | 0.992 | 0.994 | 1.000 | 4028 |
| REACTOME_JOSEPHIN_DOMAIN_DUBS | 10 | 0.293 | 0.683 | 0.877 | 0.994 | 1.000 | 1569 |
| REACTOME_VIRAL_MESSENGER_RNA_SYNTHESIS | 44 | 0.199 | 0.681 | 0.939 | 0.994 | 1.000 | 2493 |
| REACTOME_FORMATION_OF_TC_NER_PRE_INCISION_COMPLEX | 52 | 0.194 | 0.680 | 0.952 | 0.994 | 1.000 | 2789 |
| REACTOME_NUCLEOBASE_CATABOLISM | 25 | 0.224 | 0.678 | 0.905 | 0.995 | 1.000 | 3102 |
| REACTOME_INOSITOL_PHOSPHATE_METABOLISM | 36 | 0.201 | 0.674 | 0.919 | 0.997 | 1.000 | 3670 |
| REACTOME_NECTIN_NECL_TRANS_HETERODIMERIZATION | 4 | 0.368 | 0.674 | 0.838 | 0.996 | 1.000 | 683 |
| REACTOME_MRNA_SPLICING | 186 | 0.154 | 0.671 | 1.000 | 0.998 | 1.000 | 3858 |
| REACTOME_MITOCHONDRIAL_BIOGENESIS | 87 | 0.172 | 0.671 | 0.984 | 0.997 | 1.000 | 4502 |
| REACTOME_METALLOPROTEASE_DUBS | 21 | 0.230 | 0.671 | 0.918 | 0.996 | 1.000 | 2866 |
| REACTOME_TP53_REGULATES_TRANSCRIPTION_OF_DNA_REPAIR_GENES | 60 | 0.181 | 0.670 | 0.974 | 0.996 | 1.000 | 2991 |
| REACTOME_PRC2_METHYLATES_HISTONES_AND_DNA | 23 | 0.226 | 0.666 | 0.926 | 0.998 | 1.000 | 52 |
| REACTOME_SLBP_DEPENDENT_PROCESSING_OF_REPLICATION_DEPENDENT_HISTONE_PRE_MRNAS | 11 | 0.273 | 0.665 | 0.872 | 0.998 | 1.000 | 5002 |
| REACTOME_CITRIC_ACID_CYCLE_TCA_CYCLE_ | 22 | 0.230 | 0.664 | 0.912 | 0.997 | 1.000 | 1823 |

| | | | | | | | |
|---|---|---|---|---|---|---|---|
| REACTOME_REGULATION_OF_IFNA_SIGNALING | 12 | 0.271 | 0.663 | 0.888 | 0.997 | 1.000 | 2842 |
| REACTOME_NR1H2_NR1H3_REGULATE_GENE_EXPRESSION_TO_CONTROL_BILE_ACID_HOMEOSTASIS | 7 | 0.320 | 0.662 | 0.880 | 0.997 | 1.000 | 8641 |
| REACTOME_ERYTHROPOIETIN_ACTIVATES_STAT5 | 6 | 0.328 | 0.661 | 0.874 | 0.997 | 1.000 | 3480 |
| REACTOME_SIGNALING_BY_NTRK2_TRKB_ | 21 | 0.226 | 0.660 | 0.907 | 0.996 | 1.000 | 2378 |
| REACTOME_ZINC_EFFLUX_AND_COMPARTMENTALIZATION_BY_THE_SLC30_FAMILY | 6 | 0.323 | 0.659 | 0.880 | 0.996 | 1.000 | 3706 |
| REACTOME_RNA_POLYMERASE_II_TRANSCRIBES_SNRNA_GENES | 74 | 0.174 | 0.659 | 0.978 | 0.995 | 1.000 | 2559 |
| REACTOME_CLEC7A_DECTIN_1_INDUCES_NFAT_ACTIVATION | 11 | 0.267 | 0.655 | 0.894 | 0.997 | 1.000 | 1713 |
| REACTOME_PTEN_REGULATION | 135 | 0.158 | 0.655 | 0.994 | 0.996 | 1.000 | 4312 |
| REACTOME_REGULATION_OF_TP53_ACTIVITY_THROUGH_ACETYLATION | 30 | 0.208 | 0.654 | 0.944 | 0.995 | 1.000 | 3716 |
| REACTOME_PYRUVATE_METABOLISM_AND_CITRIC_ACID_TCA_CYCLE | 49 | 0.188 | 0.653 | 0.951 | 0.995 | 1.000 | 2897 |
| REACTOME_INFLAMMASOMES | 12 | 0.270 | 0.652 | 0.917 | 0.995 | 1.000 | 2831 |
| REACTOME_CALCITONIN_LIKE_LIGAND_RECEPTORS | 3 | 0.385 | 0.651 | 0.878 | 0.995 | 1.000 | 4036 |
| REACTOME_FBXW7_MUTANTS_AND_NOTCH1_IN_CANCER | 5 | 0.328 | 0.650 | 0.875 | 0.995 | 1.000 | 2850 |
| REACTOME_TRANSCRIPTIONAL_REGULATION_OF_WHITE_ADIPOCYTE_DIFFERENTIATION | 73 | 0.173 | 0.645 | 0.992 | 0.997 | 1.000 | 762 |
| REACTOME_DNA_DAMAGE_REVERSAL | 7 | 0.300 | 0.645 | 0.888 | 0.996 | 1.000 | 4294 |
| REACTOME_ALPHA_OXIDATION_OF_PHYTANATE | 6 | 0.309 | 0.645 | 0.870 | 0.995 | 1.000 | 2638 |
| REACTOME_PROCESSING_OF_CAPPED_INTRON_CONTAINING_PRE_MRNA | 236 | 0.143 | 0.644 | 1.000 | 0.994 | 1.000 | 3796 |
| REACTOME_TRANSPORT_OF_MATURE_TRANSCRIPT_TO_CYTOPLASM | 79 | 0.167 | 0.639 | 0.988 | 0.997 | 1.000 | 3768 |
| REACTOME_TRANSCRIPTIONAL_ACTIVATION_OF_MITOCHONDRIAL_BIOGENESIS | 51 | 0.179 | 0.639 | 0.970 | 0.996 | 1.000 | 3006 |
| REACTOME_METHYLATION | 10 | 0.264 | 0.631 | 0.919 | 1.000 | 1.000 | 3829 |
| REACTOME_ACTIVATED_NTRK2_SIGNALS_THROUGH_RAS | 7 | 0.297 | 0.630 | 0.901 | 1.000 | 1.000 | 4574 |
| REACTOME_SARS_COV_INFECTIONS | 74 | 0.166 | 0.628 | 0.995 | 1.000 | 1.000 | 2713 |

| | | | | | | | |
|---|---|---|---|---|---|---|---|
| REACTOME_REGULATION_OF_MRNA_STABILITY_BY_PROTEINS_THAT_BIND_AU_RICH_ELEMENTS | 85 | 0.159 | 0.622 | 0.992 | 1.000 | 1.000 | 4312 |
| REACTOME_LYSINE_CATABOLISM | 10 | 0.267 | 0.621 | 0.937 | 1.000 | 1.000 | 537 |
| REACTOME_SIGNALING_BY_NOTCH4 | 79 | 0.161 | 0.617 | 1.000 | 1.000 | 1.000 | 4312 |
| REACTOME_APC_C_CDH1_MEDIATED_DEGRADATION_OF_CDC20_AND_OTHER_APC_C_CDH1_TARGETED_PROTEINS_IN_LATE_MITOSIS_EARLY_G1 | 72 | 0.161 | 0.617 | 0.995 | 1.000 | 1.000 | 4312 |
| REACTOME_MITOCHONDRIAL_PROTEIN_IMPORT | 62 | 0.169 | 0.614 | 0.987 | 1.000 | 1.000 | 3138 |
| REACTOME_RUNX1_REGULATES_ESTROGEN_RECEPTOR_MEDIATED_TRANSCRIPTION | 5 | 0.312 | 0.612 | 0.906 | 1.000 | 1.000 | 1908 |
| REACTOME_MET_RECEPTOR_RECYCLING | 7 | 0.289 | 0.612 | 0.932 | 1.000 | 1.000 | 5052 |
| REACTOME_NS1_MEDIATED_EFFECTS_ON_HOST_PATHWAYS | 40 | 0.180 | 0.611 | 0.980 | 1.000 | 1.000 | 2493 |
| REACTOME_APC_C_MEDIATED_DEGRADATION_OF_CELL_CYCLE_PROTEINS | 85 | 0.155 | 0.608 | 0.998 | 1.000 | 1.000 | 4312 |
| REACTOME_TRANSPORT_OF_THE_SLBP_DEPENDANT_MATURE_MRNA | 36 | 0.179 | 0.604 | 0.969 | 1.000 | 1.000 | 2493 |
| REACTOME_ANTIGEN_PRESENTATION_FOLDING_ASSEMBLY_AND_PEPTIDE_LOADING_OF_CLASS_I_MHC | 24 | 0.198 | 0.603 | 0.971 | 1.000 | 1.000 | 2229 |
| REACTOME_MITOTIC_METAPHASE_AND_ANAPHASE | 224 | 0.135 | 0.600 | 1.000 | 1.000 | 1.000 | 3611 |
| REACTOME_ERYTHROPOIETIN_ACTIVATES_PHOSPHOLIPASE_C_GAMMA_PLCG_ | 6 | 0.287 | 0.598 | 0.911 | 1.000 | 1.000 | 3480 |
| REACTOME_SIALIC_ACID_METABOLISM | 20 | 0.209 | 0.597 | 0.942 | 1.000 | 1.000 | 2628 |
| REACTOME_SYNTHESIS_OF_LEUKOTRIENES_LT_AND_EOXINS_EX_ | 6 | 0.285 | 0.594 | 0.918 | 1.000 | 1.000 | 1445 |
| REACTOME_CYTOSOLIC_TRNA_AMINOACYLATION | 23 | 0.200 | 0.592 | 0.961 | 1.000 | 1.000 | 3640 |
| REACTOME_MET_ACTIVATES_PI3K_AKT_SIGNALING | 4 | 0.313 | 0.589 | 0.902 | 1.000 | 1.000 | 1771 |
| REACTOME_PRE_NOTCH_PROCESSING_IN_GOLGI | 16 | 0.214 | 0.589 | 0.949 | 1.000 | 1.000 | 3326 |
| REACTOME_SEPARATION_OF_SISTER_CHROMATIDS | 179 | 0.136 | 0.589 | 1.000 | 1.000 | 1.000 | 2493 |
| REACTOME_SIGNALING_BY_PDGFR_IN_DISEASE | 17 | 0.211 | 0.588 | 0.956 | 1.000 | 1.000 | 3322 |

| | | | | | | | |
|---|---|---|---|---|---|---|---|
| REACTOME_ALPHA_LINOLENIC_OMEGA3_AND_LINOLEIC_OMEGA6_ACID_METABOLISM | 11 | 0.234 | 0.583 | 0.944 | 1.000 | 1.000 | 4989 |
| REACTOME_TP53_REGULATES_TRANSCRIPTION_OF_ADDITIONAL_CELL_CYCLE_GENES_WHOSE_EXACT_ROLE_IN_THE_P53_PATHWAY_REMAIN_UNCERTAIN | 21 | 0.201 | 0.579 | 0.967 | 1.000 | 1.000 | 3791 |
| REACTOME_SIRT1_NEGATIVELY_REGULATES_RRNA_EXPRESSION | 18 | 0.206 | 0.578 | 0.974 | 1.000 | 1.000 | 4817 |
| REACTOME_SODIUM_CALCIUM_EXCHANGERS | 4 | 0.322 | 0.577 | 0.921 | 1.000 | 1.000 | 8625 |
| REACTOME_PURINE_CATABOLISM | 15 | 0.213 | 0.574 | 0.958 | 1.000 | 1.000 | 361 |
| REACTOME_REGULATION_OF_PTEN_STABILITY_AND_ACTIVITY | 66 | 0.155 | 0.573 | 0.997 | 1.000 | 1.000 | 4312 |
| REACTOME_RUNX3_REGULATES_IMMUNE_RESPONSE_AND_CELL_MIGRATION | 2 | 0.370 | 0.571 | 0.944 | 1.000 | 1.000 | 1908 |
| REACTOME_NA_CL_DEPENDENT_NEUROTRANSMITTER_TRANSPORTERS | 2 | 0.373 | 0.569 | 0.944 | 1.000 | 1.000 | 1892 |
| REACTOME_SIGNALING_BY_FGFR3_FUSIONS_IN_CANCER | 9 | 0.244 | 0.567 | 0.934 | 1.000 | 1.000 | 4574 |
| REACTOME_MITOCHONDRIAL_UNCOUPLING | 4 | 0.310 | 0.566 | 0.941 | 0.999 | 1.000 | 4038 |
| REACTOME_ESTROGEN_BIOSYNTHESIS | 3 | 0.337 | 0.565 | 0.951 | 0.999 | 1.000 | 8434 |
| REACTOME_SLC_TRANSPORTER_DISORDERS | 54 | 0.156 | 0.564 | 0.995 | 0.998 | 1.000 | 2493 |
| REACTOME_REGULATION_OF_TP53_ACTIVITY_THROUGH_METHYLATION | 19 | 0.199 | 0.559 | 0.976 | 0.999 | 1.000 | 4568 |
| REACTOME_THE_FATTY_ACID_CYCLING_MODEL | 4 | 0.310 | 0.559 | 0.943 | 0.998 | 1.000 | 4038 |
| REACTOME_BASE_EXCISION_REPAIR_AP_SITE_FORMATION | 26 | 0.182 | 0.557 | 0.986 | 0.998 | 1.000 | 3185 |
| REACTOME_ABC_FAMILY_PROTEINS_MEDIATED_TRANSPORT | 85 | 0.144 | 0.555 | 0.998 | 0.997 | 1.000 | 4312 |
| REACTOME_HCMV_LATE_EVENTS | 60 | 0.152 | 0.555 | 0.998 | 0.997 | 1.000 | 3295 |
| REACTOME_BETA_OXIDATION_OF_DECANOYL_COA_TO_OCTANOYL_COA_COA | 6 | 0.271 | 0.553 | 0.960 | 0.996 | 1.000 | 9273 |
| REACTOME_NEF_MEDIATED_CD8_DOWN_REGULATION | 7 | 0.256 | 0.549 | 0.953 | 0.996 | 1.000 | 2576 |
| REACTOME_REGULATION_OF_PYRUVATE_DEHYDROGENASE_PDH_COMPLEX | 15 | 0.204 | 0.549 | 0.978 | 0.996 | 1.000 | 4247 |
| REACTOME_SUMOYLATION_OF_RNA_BINDING_PROTEINS | 47 | 0.158 | 0.543 | 0.996 | 0.996 | 1.000 | 2720 |

| | | | | | | | |
|---|---|---|---|---|---|---|---|
| REACTOME_B_WICH_COMPLEX_POSITIVELY_REGULATES_RRNA_EXPRESSION | 41 | 0.158 | 0.528 | 0.991 | 1.000 | 1.000 | 3796 |
| REACTOME_CREB1_PHOSPHORYLATION_THROUGH_THE_ACTIVATION_OF_ADENYLATE_CYCLASE | 8 | 0.234 | 0.528 | 0.969 | 0.999 | 1.000 | 910 |
| REACTOME_ERYTHROPOIETIN_ACTIVATES_PHOSPHOINOSITIDE_3_KINASE_PI3K_ | 8 | 0.236 | 0.523 | 0.970 | 0.999 | 1.000 | 3480 |
| REACTOME_ABERRANT_REGULATION_OF_MITOTIC_G1_S_TRANSITION_IN_CANCER_DUE_TO_RB1_DEFECTS | 16 | 0.194 | 0.522 | 0.980 | 0.998 | 1.000 | 1769 |
| REACTOME_ACTIVATED_NTRK3_SIGNALS_THROUGH_PI3K | 5 | 0.274 | 0.520 | 0.958 | 0.998 | 1.000 | 1814 |
| REACTOME_HDL_ASSEMBLY | 5 | 0.260 | 0.505 | 0.967 | 1.000 | 1.000 | 9401 |
| REACTOME_PHOSPHORYLATION_OF_EMI1 | 6 | 0.244 | 0.497 | 0.974 | 1.000 | 1.000 | 9608 |
| REACTOME_BIOTIN_TRANSPORT_AND_METABOLISM | 11 | 0.207 | 0.497 | 0.974 | 1.000 | 1.000 | 2489 |
| REACTOME_NEF_MEDIATED_DOWNREGULATION_OF_MHC_CLASS_I_COMPLEX_CELL_SURFACE_EXPRESSION | 9 | 0.221 | 0.495 | 0.971 | 0.999 | 1.000 | 4136 |
| REACTOME_SUMOYLATION_OF_IMMUNE_RESPONSE_PROTEINS | 11 | 0.196 | 0.478 | 0.987 | 1.000 | 1.000 | 2498 |
| REACTOME_NEPHRIN_FAMILY_INTERACTIONS | 16 | 0.176 | 0.478 | 0.995 | 1.000 | 1.000 | 4350 |
| REACTOME_NUCLEAR_PORE_COMPLEX_NPC_DISASSEMBLY | 36 | 0.143 | 0.470 | 1.000 | 1.000 | 1.000 | 2493 |
| REACTOME_PROCESSING_OF_SMDT1 | 15 | 0.175 | 0.464 | 0.995 | 1.000 | 1.000 | 10485 |
| REACTOME_CONDENSATION_OF_PROPHASE_CHROMOSOMES | 23 | 0.153 | 0.452 | 1.000 | 1.000 | 1.000 | 3550 |
| REACTOME_PI3K_AKT_ACTIVATION | 7 | 0.205 | 0.438 | 0.992 | 1.000 | 1.000 | 10107 |
| REACTOME_SUMOYLATION_OF_DNA_METHYLATION_PROTEINS | 16 | 0.156 | 0.426 | 1.000 | 1.000 | 1.000 | 4102 |
| REACTOME_ACTIVATION_OF_CASPASES_THROUGH_APOPTOSOME_MEDIATED_CLEAVAGE | 6 | 0.203 | 0.412 | 1.000 | 1.000 | 1.000 | 10138 |
| REACTOME_NEGATIVE_EPIGENETIC_REGULATION_OF_RRNA_EXPRESSION | 58 | 0.111 | 0.399 | 1.000 | 1.000 | 1.000 | 3548 |
| REACTOME_GALACTOSE_CATABOLISM | 5 | 0.205 | 0.395 | 1.000 | 0.999 | 1.000 | 4508 |
